# In Situ Root Dataset Expansion Strategy Based on an Improved CycleGAN Generator

**DOI:** 10.34133/plantphenomics.0148

**Published:** 2024-02-12

**Authors:** Qiushi Yu, Nan Wang, Hui Tang, JiaXi Zhang, Rui Xu, Liantao Liu

**Affiliations:** ^1^ College of Mechanical and Electrical Engineering, Hebei Agricultural University, 071000 Baoding, China.; ^2^ College of Foreign Languages, Hebei Agricultural University, 071000 Baoding, China.; ^3^ College of Agronomy, Hebei Agricultural University, 071000 Baoding, China.

## Abstract

The root system plays a vital role in plants' ability to absorb water and nutrients. In situ root research offers an intuitive approach to exploring root phenotypes and their dynamics. Deep-learning-based root segmentation methods have gained popularity, but they require large labeled datasets for training. This paper presents an expansion method for in situ root datasets using an improved CycleGAN generator. In addition, spatial-coordinate-based target background separation method is proposed, which solves the issue of background pixel variations caused by generator errors. Compared to traditional threshold segmentation methods, this approach demonstrates superior speed, accuracy, and stability. Moreover, through time-division soil image acquisition, diverse culture medium can be replaced in in situ root images, thereby enhancing dataset versatility. After validating the performance of the Improved_UNet network on the augmented dataset, the optimal results show a 0.63% increase in mean intersection over union, 0.41% in F1, and 0.04% in accuracy. In terms of generalization performance, the optimal results show a 33.6% increase in mean intersection over union, 28.11% in F1, and 2.62% in accuracy. The experimental results confirm the feasibility and practicality of the proposed dataset augmentation strategy. In the future, we plan to combine normal mapping with rendering software to achieve more accurate shading simulations of in situ roots. In addition, we aim to create a broader range of images that encompass various crop varieties and soil types.

## Introduction

The root system architecture is an important organ for plants to absorb water and nutrients and plays an important role in plant growth and productivity [1–3]. The root system architecture directly contacts the soil, first perceives the changes under soil environmental conditions, affects how well crops absorb water and nutrients, and is strongly correlated with yield [4,5]. Accurate detection and analysis of root phenotype are the keys to analyzing crop response to environmental conditions.

In situ root observation is an important method for the study of dynamic root phenotype, which consists of in situ cultivation and in situ imaging. In situ cultivation can be divided into hydroponics [6], soil culture, gel culture [7], and paper culture [8] according to the different culture substrates. Among them, the soil culture method is difficult to observe the root system architecture through the soil, while the hydroponics, gel culture, and paper culture methods can directly observe the plant root system architecture; however, because of the different culture medium, the root morphology will be different from the natural growth, and the root hair is difficult to form. The gel culture method and paper medium culture method are restricted by the supporting ability of the culture medium and the content of the nutrient solution, which can only support the growth of plant seedling morphology and cannot achieve the observation of plant roots in the whole growth period. In situ imaging methods include minirhizotron [9], x-ray tomography technology [10], magnetic resonance imaging [11], ground penetrating radar (GPR) [12], and digital equipment imaging method [13]. Among them, the minirhizotron technique is a nondestructive method for direct observation and study of plant root systems at specific points. Combining the minirhizotron tube with the shooting device enables nondestructive observation of root systems. X-ray tomography technology collects root morphology through the different absorption levels of x-rays by soil and roots. Magnetic resonance imaging obtains encoded nuclear magnetic resonance information by detecting resonance signals from objects at different positions in a magnetic field, providing a modern tomographic imaging technique for reconstructing internal images of objects using computers. GPR utilizes high-frequency electromagnetic waves to reflect in a medium with uneven dielectric properties to obtain a cross-sectional image. Through interpretation, the nature and structure of underground objects can be determined. Digital equipment imaging method combines digital equipment with growth devices to dynamically collect high-resolution in situ root images without changing the soil environment and affecting root growth status, which is beneficial to improving the efficiency of root system segmentation and quantitative analysis. However, x-ray tomography technology and magnetic resonance imaging have the disadvantages of high cost, long collection time, and low resolution and cannot achieve root collection in the field. Although GPR can collect root system architecture in the field, its acquisition resolution is easily affected by the soil environment. At the same time, the equipment cost is high, so it is difficult to achieve batch collection in the field. Minirhizotron is widely used in field root collection, which has the advantages of low cost and easy installation, but the minirhizotron has low imaging resolution and cannot observe the complete root configuration. The RhizoPot platform developed by our laboratory in the early stage has been reported [14], which can realize the nondestructive acquisition of complete root image through a scanner and the RhizoPot growth device. In this experiment, the in situ root image collected by RhizoPot device is used as the dataset.

The extensive application of minirhizotron brings the problem of in situ root image processing, and the key is to identify and segment root system architecture. The traditional recognition methods of in situ root images include manual depiction, semiautomatic interactive recognition, and fully automatic threshold segmentation. Among them, the manual depiction method is highly dependent on human subjectivity, with large discrepancies and low efficiency [15,16]. Semiautomatic interactive identification methods, such as WinRhizo [17], although the efficiency is improved, still rely too much on the operator’s subjective ability to distinguish roots and their own experience. The automatic threshold segmentation method has obvious improvement in efficiency and objectivity; however, it still suffers from large identification errors and soil noise interference.

Compared with traditional methods, in situ root image recognition based on deep learning is more conducive to mining the deep features of the target. In recent years, this kind of method is often used in the recognition and segmentation of in situ root image. For example, SegRoot [18] based on SegNet [19] can distinguish dark soil from roots, but in the image with a ruler, the ruler will be mistaken for roots; SNAP (soybean nodule acquisition pipeline) [20], based on UNet [21] and RetinaNet [22], can accurately detect root nodules on soybean roots and segment main roots; ChronoRoot integrates UNet, ResUNet, DSResUNet, SegNet, and DeeplabV3+ [23]. Its model principle is to average the segmentation results of the integrated model, which can effectively realize the architecture and integration of multiple models [24]. Deep learning can effectively avoid the impact of threshold and soil noise in the process of image segmentation.

Root segmentation methods based on deep learning are becoming more and more popular, but they need a large number of manually labeled datasets to support training. The manual annotation process is time-consuming and labor-consuming. For example, it takes about 4.5 h to annotate a 1,200-dpi in situ root image (10,200 × 14,039 pixels) using the Adobe Photoshop lasso tool. RootPainter [25] is one of the most commonly used tools by root ecologists. Its interactive annotation method substantially reduces labeling time, and it also performs well on datasets with smaller sample sizes. More importantly, different culture substrates have great differences in color or compactness, and the change of image features further affects the training results. It is urgent to change this situation. The generative adversarial network CycleGAN [26] has advantages in domain adaptation. On the one hand, CycleGAN can be trained without paired data. On the other hand, CycleGAN’s unique training strategy can make the 2 styles of data migrate to each other without retraining. Therefore, CycleGAN is widely used in dataset generation. Chen et al. [27] used pixel2pixel and CycleGAN networks to expand the outdoor green space data. Zhang et al. [28] used CycleGAN to transform the source fruit images into the target fruit images, reducing the imbalance between the types of datasets, to enhance the datasets. However, there are few reports on the dataset generation method of in situ images [29]. At the same time, the output will become diversified because of the generative adversarial network. Although this is not a disadvantage, in the actual operation process, the background pixels often change, which restricts the effect of in situ root dataset enhancement. The aforementioned issues must be resolved to accomplish the dataset enhancement objective. The goal of this study is to achieve texture transfer from mask images to root structures, extracting in situ root structures to enhance the dataset and expand the root architecture. The migration of root texture is achieved through training existing in situ root datasets using CycleGAN. The extraction of in situ root structure is distinguished from the background and roots by a postprocessing method based on spatial coordinates. The enhancement of the dataset is achieved by incorporating the extracted root system structures into shadows and soil backgrounds. Ultimately, by enhancing the in situ root dataset, the root architecture is expanded, and the generalization performance of the network is improved.

## Materials and Methods

### Experimental pipeline

**Fig. 1.**
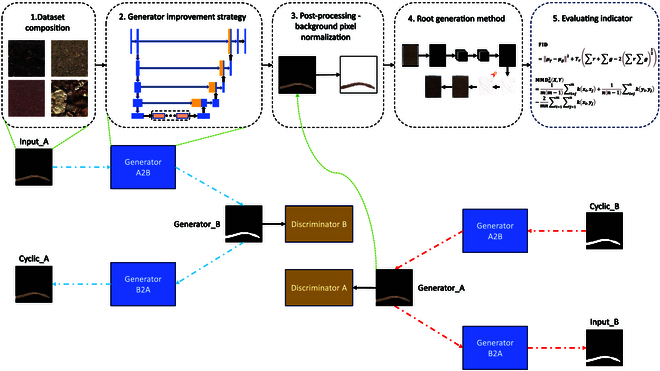
Experimental pipeline. (1) Collect pictures of different soils through the RhizoPot platform. (2) The improved generator structure can obtain a root texture that closely resembles reality. (3) Extract the generated root system from the background through postprocessing methods. (4) The mask images undergo segmentation; CycleGAN generates root systems, followed by stitching, postprocessing, and the addition of shadows and background to obtain the generated in situ root images. Removing the stitching operation enables the generation of an enhanced dataset using CycleGAN. (5) FID and KID perform performance verification.

The experimental pipelines carried out in this experiment are shown in Fig. 1. The purpose is to train the existing in situ root datasets through CycleGAN so that it can generate root architectures, which can be used to enhance the datasets and broaden the root architectures. By improving the structure of the generator, it can generate a more realistic root configuration. At the same time, through the postprocessing method based on spatial coordinates, the background pixel value is unified, and then the dataset is expanded by adding shadows, soil background, etc.

### Collection and labeling of root data

This experiment was conducted at the experimental station of Hebei Agricultural University in Baoding, Hebei Province in 2020–2022 (38.85°N, 115.30°E). The principle of image acquisition equipment and the method of cotton seed cultivation were described in the previous articles of this experimental group [14,30,31]. The planting varieties are “Guoxin No.9” [32] and “JiNongda 36” [33]. For the cotton planted in 8 groups of RhizoPot devices, the Epson scanner V39 (Epson Inc., Suwa Shi, Nagano, Japan) was used to collect images of cotton seedling roots for 110 consecutive days. The scanning accuracy was set to 1,200 dpi, and the image resolution was 10,200 × 14,039 pixels (saved in BMP format). According to the time and group, the sequential images taken continuously can be obtained. Selecting results with clear shooting, no noise, and consistent resolution, we obtained 100 images of the variety “Guoxin No.9” and 25 images of the variety “JiNongda 36”. After annotation processing, the former was used for training, and the latter was used for testing.

Image annotation was completed by experienced agronomic experts using the lasso tool of Adobe Photoshop CC (Adobe Inc., San Jose, CA, United States). All pixels considered to be the root were marked in white and stored in a new layer. Finally, the remaining pixels were marked in black. The labeling time of a single image was about 4.5 h.

In preliminary experiments, we used these 100 in situ root images and annotations to verify the performance of Improved_UNet. The results showed that Improved_UNet outperformed SegNet, DeeplabV3+, and UNet in both predictive outcomes and network performance metrics. In this experiment, Improved_UNet was used to segment and extract features from unlabeled in situ root images using existing weights. In addition, it served as a verification network to verify the effect of the enhanced dataset.

### Dataset composition

**Fig. 2.**
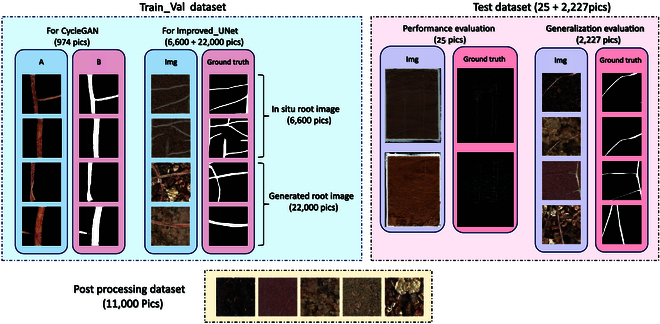
Various datasets used in this experiment. Train_Val dataset is used to train the generative adversarial network CycleGAN and the semantic segmentation network Improved_UNet. In “For CycleGAN”, we process the in situ root image as follows: We blend the original in situ root images with their masks, retaining only the roots. Subsequently, the root images are used as training set A, and the masks as training set B for CycleGAN training. In “For Improved_UNet”, 6,600 in situ root images and 22,000 in situ root images generated by CycleGAN are used as the training set and validation set for the 5-fold cross-validation of Improved_UNet. The postprocessing dataset contains 11,000 soil images collected through RhizoPot, which are used for adding soil in postprocessing. The test dataset consists of 2 parts: “Performance evaluation” contains 25 in situ root images with a resolution of 10,200 × 14,039 pixels for network performance validation. “Generalization evaluation” contains 2,227 in situ root images with a resolution of 256 × 256 pixels generated by CycleGAN for verification of generalization performance.

The annotated images were split into 256- × 256-pixel sizes, serving as training set B for the root dataset and ground truth for the validation dataset, with the same number as the training set A. Figure 2 is an example of the following datasets.

#### Train_Val dataset

• For CycleGAN: Because of the growth pattern of plant roots, a single in situ root image inevitably contains both newly formed and aging roots, exhibiting differences in color, texture, size, and other aspects. Perhaps, because of the different characteristics of the 2 roots, during the generation of images by CycleGAN, the algorithm automatically categorizes new and aging roots into separate classes. In such cases, if there is a substantial disparity in the proportion of new and aging roots in the image, it can affect the generation effectiveness of CycleGAN. To balance the dataset and enhance the stability of the CycleGAN generator, we select in situ root images collected at a later growth stage (with an aging root to new root ratio of approximately 1:1). Using annotated mask images to cover the background soil and retain only the root system after cropping, we obtain 974 images. These images are then utilized for training the generative adversarial network CycleGAN. In this process, the root images serve as data A for CycleGAN style transfer training, and the mask images serve as data B.
• For Improved_UNet: We utilized the semantic segmentation network Improved_UNet as the verification of this experiment. The Improved_UNet dataset consists of 2 parts: One is to select 3 in situ root images and divide them into small images with a resolution of 256 × 256 pixels, resulting in a total of 6,600 images. This subset is used to simulate scenarios with limited data. The second part involves in situ root images generated through CycleGAN, processed as outlined in the “Root generation methods” section, yielding 22,000 generated in situ root images. These 2 types of data together form the augmented in situ root dataset for training Improved_UNet.

#### Postprocessing dataset

Postprocessing dataset consists of a variety of soils images, and the soil images used in this experiment were collected by the scanner of RhizoPot. Among them, RhizoPot contains 6.5 l of soil, and laterite, black soil, sand, vermiculite, and ordinary soil were selected as observation objects. The relevant parameters of soil are as follows. This experiment selected 9:00 AM, 12:00 AM, 3:00 PM, and 6:00 PM of the day as the acquisition time. The scanning accuracy was set to 1,200 dpi, and 20 soil images were obtained through acquisition, with a resolution of 10,200 × 14,039 pixels. Then, through the segmentation operation, 11,000 soil images with a resolution of 256 × 256 pixels were obtained as postprocessing dataset. Postprocessing dataset was used as an image processing step after the root system architecture was generated to combine to produce more realistic in situ root system architecture images of soil culture.

Laterite is a lateritic soil collected in Xishuangbanna, Yunnan Province, China (21°N, 100°E). The soil color is red due to the presence of iron, aluminum, and other elements. The weight of the laterite used in this experiment was 9.1 kg.

Black soil is humus soil collected from Songnen Plain (45°N, 120°E) in Northeast China. The soil color is black–brown and contains a small amount of fine roots. The weight of the black soil used in this experiment was 4.33 kg.

The sand is the river sand collected from the Yellow River (40°N). The weight of the sand used in this experiment was 8.542 kg.

Vermiculite is a silicate mineral with low density and good thermal insulation performance. The weight of vermiculite used in this experiment was 1.733 kg.

Ordinary soil was collected from the experimental field of Hebei Agricultural University in Baoding, Hebei Province (39°N, 114°E). The weight of ordinary soil used in this experiment was 8.66 kg.

#### Test dataset

• Performance evaluation: Twenty-five “JiNongda 36” in situ root images with a resolution of 10,200 × 14,039 pixels are used to enhance the performance evaluation of the dataset.
• Generalization evaluation: From the masks of 25 “JiNongda 36” in situ root images, 2,227 masks containing root systems were cut out using a template with a resolution of 256 × 256. Root images were generated through CycleGAN, and after adding shadows and soil background, 2,227 generated in situ root images were obtained, which were used to verify the generalization performance of the dataset.

### CycleGAN generator improvement strategy

The original CycleGAN generation network is a U-shaped encoder–decoder structure. In the encoder part, the image is extracted by 2-layer convolution downsampling. In the bottleneck layer, 9 residual blocks are introduced to further extract the global features of the image. In the decoder part, the network receives the characteristic images of the residual block, performs upsampling through 2-layer transpose convolution, and outputs the generated images. This generator is simple in structure and easy to train. However, it is difficult to extract the deep features of the images.

**Fig. 3.**
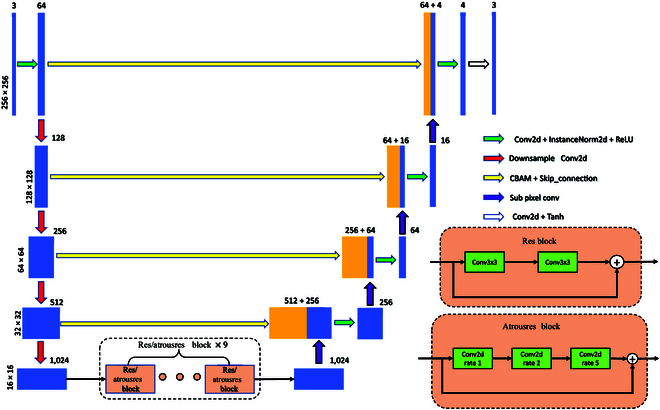
Schematic diagram of the improved generator structure. For the generator, we compared the following changes: The generator network was changed to 4 layers of downsampling, and skip connections and CBAM attention modules were added to each layer corresponding to the decoder. The Pixelshuffle algorithm was used instead of the original convolutional upsampling. In addition, a combination of dilated convolutions with dilation rates of 1, 2, and 5 replaced the convolutions in the residual blocks.

So, this paper discusses some improved methods for convolutional neural network:

1. Increase the number of samples and improve the receptive field of the network. In neural networks, as the number of convolutional downsampling layers increases, the network’s receptive field becomes larger, making it easier to capture global features and enhance overall network performance.
2. On the basis of the idea of skip connection of UNet, the Convolutional Block Attention Module (CBAM)[34] attention module is integrated. CBAM is a lightweight attention mechanism that enhances network performance by extracting features from both spatial and channel dimensions. It can combine the underlying location information with the deep semantic information, locate the information of interest at the same time, and suppress useless information.
3. Subpixel convolution is used instead of deconvolution upsampling. Compared with deconvolution upsampling, subpixel convolution eliminates invalid information, improves network efficiency, and ensures model accuracy.
4. Atrous convolution is a convolutional idea proposed to solve the problem of image semantic segmentation in which downsampling will reduce the image resolution and lose information. It is often used to reduce the number of parameters. This article intends to test the weight size produced by using atrous convolution and the changes to the generator performance. In addition, to prevent the grid effect caused by using atrous convolution, we add an extra layer of convolution to the residual block of the generator to satisfy the mixed atrous convolution with expansion rate of 1, 2, and 5.
  Among them, the simultaneous use of the CBAM attention module and subpixel convolution has been proven to improve the network accuracy in the root segmentation task [35]. The method of increasing the number of samples is also widely used in the generator. Using a skip connection can play a positive role in network training. The dilated convolution method is also considered to have an optimizing effect on network optimization [23]. The generator structure improved by these improvement strategies is shown in Fig. 3. At the same time, through the ablation experiment, this paper analyzes the effect of each improved part and its combination on the generator network and selects the part or combination with the best generation effect.

### Postprocessing

The background pixels of the root images generated by CycleGAN are different, and the quality of the generated root images is reduced by directly making the background transparent. In response to the above problems, this paper proposes 2 methods, namely, the threshold selection postprocessing strategy based on the red–green–blue (RGB) histogram and spatial-coordinate-based target background separation method.

#### Threshold selection postprocessing strategy based on the RGB histogram

The followings are the specific steps of the threshold selection postprocessing strategy based on the RGB histogram: For images without roots, use black images with all pixel values (0, 0, 0) instead. For the images containing the root system architecture, add it to the corresponding mask image. At this time, the pixel value of the background remains unchanged, and the pixel value of the root system architecture becomes (255, 255, 255). Calculate the RGB histogram of the image, and select the maximum RGB threshold whose number of pixels other than (255, 255, 255) is not 0. Finally, considering the 2 threshold ranges, the final segmentation threshold is selected.

#### Spatial-coordinate-based target background separation method

The following is the algorithm of spatial-coordinate-based target background separation method: This method is actually to remember the spatial coordinates of the background and target in the mask images and then assign the corresponding position of the generated image to realize the unification of the background pixels of the generated root images and the background target of the generated mask images.

### Root generation methods

#### Root image generation method for training

By putting the root mask into the CycleGAN generator for prediction, the mask to root conversion can be realized through the generation network. The generated image is converted to RGBA (contains red, green, blue, and alpha color spaces) type through image processing, and the black background part in the image is converted to (255, 255, 255, 0). After adding the shadow and combining it with the soil map, the generated in situ root image can be obtained. Combined with the mask image, it can be directly used for the training the of semantic segmentation network.

#### Root image generation for expanded conformational analysis

**Fig. 4.**
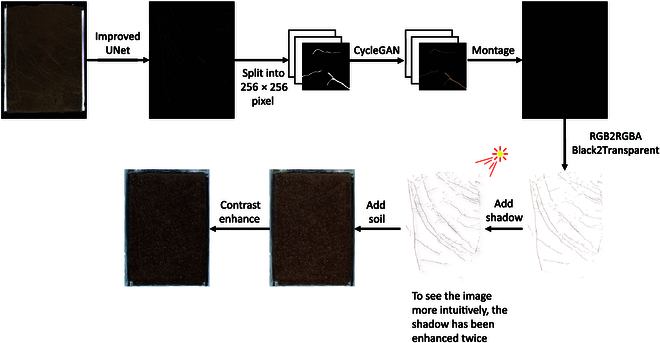
Root image generation method for extended configuration analysis. A single in situ root image is segmented and extracted for root structure using Improved_UNet. After segmentation, multiple small images with a resolution of 256 × 256 pixels are generated through cropping. Then CycleGAN is used to generate the root system texture and then spliced to obtain the entire image. The root texture is then postprocessed to become a GRT image, and after adding shadows and background, a GRS image is formed.

Put a continuous root mask into the CycleGAN generator for prediction and generate the roots with transparent background (GRT) after network generation, splicing, and color conversion. Adding shadows and combining them with soil images give the generation of roots in soil background (GRS) with a complete soil background (Fig. 4). In addition, root configuration analysis is added.

#### Batch realization strategy

**Fig. 5.**
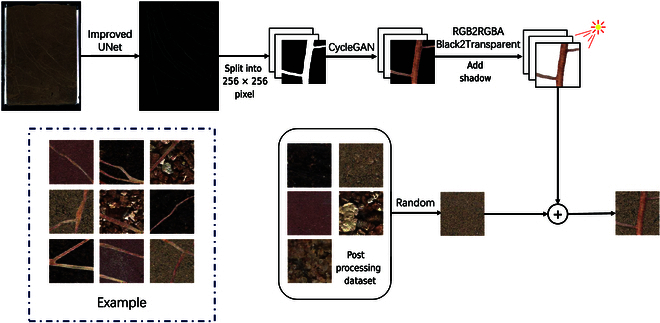
Batch implementation of root image generation method for training. A single in situ root image undergoes segmentation and extraction of root structure using Improved_UNet. Subsequently, through cropping, several small images are generated with a resolution of 256 × 256 pixels. These small images then undergo CycleGAN to generate root system textures. After postprocessing, they become GRT images. Shadows are added, and random selections of soil backgrounds are incorporated, forming GRS images. The example section showcases the generated root systems under different soil conditions. Example shows the results of root system generation under different soils.

To generate root images efficiently, this experiment corresponded GRT and postprocessing dataset by the exhaustive method. First, traverse the folder where GRT images are stored and randomly take out the GRT image; Then, traverse the folder where postprocessing dataset is stored, overlay the layer with the a GRT image taken out, and get a GRS image, which goes back and forth (Fig. 5).

### Evaluation indicators

Fréchet inception distance (FID) [36] and Kernel inception distance (KID) [37] are the 2 most popular indicators for evaluating image generation performance. A lower score means that the generated image is more similar to the original image. FID and KID are calculated as follows:

$$\begin{array}{c} \mathrm{FID}=\;∥\mu_{\gamma }-\mu_{g}{∥}^{2}+T_{r}\left(\;\sum r+\sum g-2{\left(\sum r\sum g\right)}^{\frac{1}{2}}\right) \end{array}$$ (1)

Among them, *μ_y_* and *μ_g_* represent the mean value of the inperception feature representation of the real image and the generated image, respectively, and ∑*r* and ∑*g* represent the covariance matrix of the inperception feature representation of the real image and the generated image, respectively.

KID is the maximum mean discrepancy (MMD) of the polynomial kernel function in the concept feature representation space. It is an unbiased estimation.

$$\begin{array}{c} {\mathrm{MMD}}_{u}^{2}\left(X,Y\right)=\frac{1}{m\left(m-1\right)}\sum_{i\neq j}^{m}k\left(x_{i},x_{j}\right)\\ +\frac{1}{n\left(n-1\right)}\sum_{i\neq j}^{n}k\left(y_{i},y_{j}\right)-\frac{2}{\mathrm{mn}}\sum_{i=j}^{m}\sum_{j=1}^{n}k\left(x_{i},y_{j}\right) \end{array}$$ (2)

where *m* is the sample size of the generated images, *n* is the sample size of the real images, and *k* is the kernel.

However, there are some differences between the 2 performance indicators and the subjective feeling of human eyes. Therefore, this paper introduced the subjective scoring mechanism of human eyes (Table 1), with a full score of 10 points. By comparing the following items, we can get the subjective evaluation score of human eyes.

**Table 1.** Subjective scoring mechanism

Number	Title	Score	Total
1	Generate a pixel value for whether the background stays black or not	0: The background pixel values in the generated root system architecture are not kept black.	1
1: Maintain black background pixel values in the generated root system architecture.			
2	Repeatability of root texture in a single image	0–1: There is a high degree of repetition in the root texture of a single image.	3
2–3: Low repeatability of root texture in a single image.			
3	Root texture similarity of combined pictures	0–1: The root texture similarity of the combined image is low and easy to distinguish.	3
2–3: Combination images have high texture similarity and are difficult to distinguish.			
4	Is there an observable background pixel in the transparent background image	0: There are a large number of background pixels that have not been removed in the background.	2
1: There are a small number of background pixels that have not been removed in the background.			
2: There are trace background pixels in the background that have not been removed and are not easily observed.			
5	Is the root shadow color appropriate	0: There is a substantial difference in the background color of the root system architecture compared to the soil.	1
1: The background color of the root system architecture makes it perfectly embedded in the background.			

Table 1 shows the scoring criteria for subjective scoring, and its scoring process is consistent with the CycleGAN image generation process.

1. Generate a pixel value for whether the background stays black or not. It represents whether CycleGAN during training confuses the target with the background. To some extent, this can reflect the generator training outcome.
2. To judge the repeatability of root texture in a single image. You can judge the effect of the generator prediction.
3. Root texture similarity of combined pictures is used to judge the consistency of texture in the complete root system. Using generative adversarial network to generate a dataset will inevitably lead to the diversity of output data. While diversity in output data is inevitable in generative-adversarial-network-generated datasets, excessive diversity may decrease the authenticity of the root system, potentially leading to training failures.
4. Is there an observable background pixel in the transparent background image. This part is mainly evaluated by the results of RGB threshold segmentation. Using the default threshold (127, 127, 127), values under this threshold are considered as background. If the generator’s result contains background pixel values, it indicates a relatively poor training effect.
5. Is the root shadow color appropriate. It is used to select the transparency of the root shadow.

At the same time, to prove the feasibility of CycleGAN expanding the dataset and to evaluate the performance of various networks enhanced by the dataset objectively and accurately, this paper adopts various performance metrics, such as mean intersection over union (mIOU), accuracy, and F1 score, whose formulas are as follows:

$$\text{mIOU}=\frac{1}{k+1}\sum_{i=0}^{k}\frac{P_{\mathrm{ii}}}{\sum_{j=0}^{k}P_{\mathrm{ij}}+\sum_{j=0}^{k}P_{\mathrm{ii}}}$$ (3)

$$\text{Recall}=\frac{\mathrm{TP}}{\mathrm{TP}+\mathrm{FN}}$$ (4)

$$\text{Precision}=\frac{\mathrm{TP}}{\mathrm{TP}+\mathrm{FP}}$$ (5)

$$\text{Accuracy}=\frac{\mathrm{TP}+\mathrm{TN}}{\mathrm{TP}+\mathrm{FN}+\mathrm{TN}}$$ (6)

$$F1=2\times \text{Precision}\times \frac{\text{Recall}}{\text{Precision}+\text{Recall}}$$ (7)

In Eq. 3, the subscript *i* represents the real value, and *j* represents the predicted value. In Eqs. 4 to 6, TP is the number of pixels predicted as root and actually as root, FN is the number of pixels predicted as background but actually as root, FP is the number of pixels predicted as root but actually as background, and TN is the number of pixels predicted as background and actually as background. This paper comprehensively analyzes the 5 indicators and uses the test dataset to evaluate the model.

## Results and Discussion

### Comparison of generator improvement methods

This experiment utilized an RTX 3060 12 GB + 16 GB platform for training. For the training of CycleGAN, the learning rate was set to 0.0002, batch size to 16, and epochs to 200. Learning rate decay was applied for the first 100 epochs, with the learning rate remaining constant for the subsequent 100 epochs. The optimizer selected was Adam, and the training was performed on the Train_Val dataset for CycleGAN. For the training of Improved_UNet, the learning rate was set to 0.0001, and the total number of epochs was 100. Freeze training was conducted for the first 50 epochs, with a batch size of 4, followed by unfreeze training for the next 50 epochs with a batch size of 2. The optimizer chosen was Adam. Training was carried out separately for 6,600 in situ root images and 6,600 + 22,000 in situ root images + generated root images in the Train_Val dataset for Improved_UNet.

This paper compared the original generator structure and the 8 generator structures via the combination of the first 3 types in the “CycleGAN generator improvement strategy” section improvement schemes. Compared with the generation results trained by the Train_Val dataset—for CycleGAN, the network performance is compared using FID and KID as follows.

According to Table 2, among FID indicators, model 1 has the best network performance score; in the KID index, the network performance score of model 3 is the best. Therefore, only relying on FID and KID cannot accurately quantify the generator performance of the 2 networks. Therefore, we invited 20 agricultural students as scorers to subjectively evaluate the root structure images generated by various networks using the human eye. The training method for scorers is as follows:

**Table 2.** Improvement methods of the model and generator performance (without adding a confidence interval after the numerical value represents a confidence interval less than 0.001; the best performing value is bolded)

Model name	Improvement methods			Evaluating indicators		Size (MB)
Downsampling	Skip connection + CBAM	Subpixel convolution	FID	KID		
Model 0				21.10±0.501	0.003	43.4
Model 1	✓	✓	✓	**17.74**±**0.434**	0.002	96.9
Model 2		✓		21.68±0.415	0.012	7.56
Model 3		✓	✓	17.93±0.419	**0.001**	6.02
Model 4	✓	✓		19.66±0.400	0.002	122
Model 5	✓		✓	21.13±0.332	0.002	95.9
Model 6	✓			27.36±0.900	0.005±0.001	119
Model 7			✓	23.65±0.593	0.004	**5.95**

The scorers were trained as follows: for 1, 2, and 4, 20 representative examples were selected, and agricultural experts assigned score ranges for each image. In 3, the scorers identified distinguishable areas in the stitched images, mainly focusing on the rationality of colors. The number of unreasonable regions was then calculated, and the score range was determined on the basis of the count of these regions. In 5, without distinguishing between different generator results, various shadow opacities (10%, 25%, 50%, 75%, and 90%) were used as a comparison. The opacity with the highest score was selected as the shadow transparency. Finally, the scores from all scorers were averaged to calculate the overall score.

For each kind of network, this paper provides 3 kinds of pictures, which are respectively composed of GIS (generation of roots in splitting images), GRT, and GRS. GIS is a pseudo-root image generated by the generator for 2,200 root masks of 256 × 256 pixels, and GRT and GRS are combined root maps of 10,200 × 14,039 pixels scored against the subjective scoring mechanism in Table 1 (Table 3).

**Table 3.** Subjective evaluation of various generators (please refer to the Supplementary Materias: Subjective evaluation of various generators.pdf for details; the highest scoring value is bolded)

Model name	Scoring number (Table 1)					Total
1	2	3	4	5		
Model 0	1	2.2	2.3	2	0.7	8.2
Model 1	1	2.6	2.35	2	0.7	**8.65**
Model 2	1	2.4	2.4	2	0.7	8.5
Model 3	1	2.45	2.4	2	0.7	8.55
Model 4	1	2.5	2.35	2	0.7	8.55
Model 5	1	2.6	2.2	2	0.7	8.5
Model 6	1	2.45	2.15	1.7	0.7	8
Model 7	1	2.15	2.15	2	0.7	8

Considering the network evaluation index and subjective score, it can be found that adding any one of the 3 improvement schemes to the network will reduce the performance of the network; adding skip connection and CBAM attention mechanism while increasing downsampling will improve the network performance, and adding subpixel convolution while adding skip connection and CBAM attention mechanism will also improve the network performance, which is consistent with the conclusion of the previous experiment [35]. When the 3 improved schemes are added to the network, the network performance will be further improved.

At the same time, in the process of CycleGAN training, 4 weights will be generated, namely, 2 generator weights and 2 discriminator weights, which will greatly increase the network space. To reduce the space occupied by the network, this paper adds atrous convolution to the 3 methods of model 1, model 3, and model 4 with higher network performance than the original generator, which can theoretically reduce the network parameters. This paper tests the application of mixed atrous convolution (expansion rate is 1, 2, and 5, respectively) in the residual module. Relevant data are shown in Table 4. The results demonstrate that although theoretically, the use of hybrid atrous convolution can substantially reduce the size of the network; in practice, each residual module in the generator must be convolved one more time than the original residual module to prevent the atrous convolution from having the gridding effect. This causes the size of the network to slightly increase, and, at the same time, the use of the atrous convolution also reduces the accuracy of the generator in most of cases. In addition, the performance of the performance rises after the addition of atrous convolution to model 4 only.

**Table 4.** Generator rating under improved atrous convolution (compared to Table 2, ↓ represents an increase in value and ↑ represents a decrease in value)

Model name	Network performance		Subjective scoring (Table 1)						Size (MB)
FID	KID	1	2	3	4	5	Total		
AT_Model 1	18.59 ± 0.419↓	0.002	1	2.5↓	2.25↓	2	0.7	8.45↓	139.4↓
AT_Model 3	36.49 ± 0.361↓	0.018 ± 0.001↓	0↓	2.65	2.25↓	0↓	0.7	5.6↓	8.7↓
AT_Model 4	18.53 ± 0.307↑	0.001↑	1	2.6↑	2.15↓	2	0.7	8.45↓	166.5↓

### Generated background pixel threshold analysis

Because of the different network structures, the root background pixel values generated by the generator are different, which will make it difficult to generate GRT pictures. Therefore, this paper counts the RGB histograms of GRT images generated by various generators (please refer to the Supplementary Materials: RGB histograms of GRT images.pdf for details) and selects the threshold most suitable for separating the background and root system architecture in the histograms to reduce the impact of the network on the effect of GRT images.

From the histogram results, it can be seen that in the generated images, the background color threshold of the images without roots is different from that of the images with roots. This is because in online training, to ensure that the training results will not be affected by the black background, all images without targets are removed before training. However, when generating a full mask image, it is inevitable to generate a full-black background. Therefore, the background thresholds of the 2 images are not uniform. In the subjective evaluation, we should comprehensively consider the results of the 2 types of generated images and appropriately reduce the weight of the all-black background images.

**Fig. 6.**
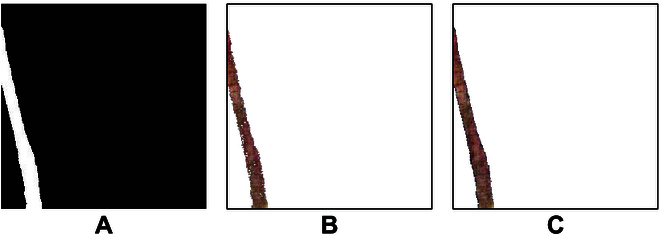
Comparison of results between 2 postprocessing methods. (A) CycleGAN generation. (B) A postprocessing method based on RGB threshold selection, with threshold selection of (127, 127, 127). (C) Postprocessing method based on spatial coordinates.

This article tested the 2 postprocessing methods mentioned in the “Postprocessing” section, and the results are shown in Fig. 6.

Figure 6 compares the effect of the 2 methods to generate the root system images, and the RGB-histogram-based threshold selection postprocessing strategy needs to determine the RGB histogram first when processing different images and then through human threshold selection to achieve a better effect of target and background segmentation. When the background pixel value is too close to the root system pixel value, this strategy will reduce the effect of the generated image (Fig. 6B). In contrast, the spatial-coordinate-based postprocessing method is fully automatic, eliminating the time for RGB threshold selection, which is faster and more effective (Fig. 6C).

At the same time, we compared the running speed of the 2 postprocessing methods, in which the threshold selection postprocessing strategy is based on the RGB histogram. The manual selection of thresholds in the RGB histogram was replaced by the maximum interclass variance method of grayscale images. We used 2,227 images to test the running speed of the 2 methods. Each method is run 3 times to reduce the impact of other factors during operation such as central processing unit temperature and memory frequency. The final result is shown in Table 5.

**Table 5.** Time comparison of 2 postprocessing methods

Method	Threshold selection postprocessing strategy based on the RGB histogram (min)	Spatial-coordinate-based target background separation method (min)
1st run	6.134	5.712
2nd run	6.095	5.677
3rd run	6.035	5.764
Average	6.088	5.718

As can be seen from Table 5, it is evident that in the time comparison between the 2 postprocessing methods, the spatial-coordinate-based target background separation method is, on average, 0.37 min faster than the threshold selection postprocessing strategy based on the RGB histogram. If the threshold selection postprocessing strategy based on the RGB histogram uses the manual threshold selection method, the time difference between the 2 methods would be further widened.

Although the effect of the threshold selection postprocessing strategy based on RGB histogram is slightly poor in root system architecture generation, the results can be used as a reference for subjective evaluation. By observing the thresholds of the target and background, it can be found that the closer the threshold interval is to the target RGB value, the worse the segmentation effect is and the lower the score will be, and vice versa.

### Root shadow, background selection results

Although there is no light effect on the underground part of the root system architecture, the scanner will rely on its light-emitting equipment to take pictures. In addition, the depth of the soil contacting the root system architecture is different from that of other parts, which will produce parts similar to the shadow of the root system architecture. The generated GRT image will therefore be more realistic if shadows are added. In contrast, the “shadow” of the original image is more closely resembled when the light source projection angle is perpendicular to the image. We compared the root shadow by selecting opacity of 0%, 10%, 25%, 50%, 75%, and 90% (see the Supplementary Materials: root shadow example.tif). We use 75% opacity after comparing it to the original image to produce a more convincing projection effect.

In this paper, different kinds of soil are used as the background for the generation of root images to have better generality in the training of the semantic segmentation network. The results of root formation under different soils as backgrounds are shown in Fig. 5. They can be found that on the basis of model 1, the root formation method mentioned in the “Root generation methods” section can make the background, and roots have a relatively good degree of chimerism.

### Lowest loss weight and latest weight results

This paper refers to the previous research, in which Shen et al. [38] used the latest results of weight and Jia et al. [39] used the results with the lowest weight. At the same time, during the training of the model, this paper found that during the training of CycleGAN, the loss value sometimes increased with the number of training iterations. Therefore, this experiment compared the 2 training strategies of the lowest loss and the latest weight. The training strategy remains consistent (see the “Comparison of generator improvement methods” section), and the relevant results are shown in the Table 6. The results show that the lowest loss value does not mean that the network effect will be good in the training of the generation adversarial network. In this experiment, the latest weight results are used for subjective evaluation and dataset generation.

**Table 6.** Performance analysis of the lowest loss model (compared to Table 2, ↓ represents an increase in numerical value, and ↑ represents a decrease in numerical value)

Model name	Improvement methods			Evaluating indicators	
Downsampling	Skip connection + CBAM	Subpixel convolution	FID	KID
Model 1	✓	✓	✓	19.56 ± 0.399↓	0.002
Model 2		✓		26.30 ± 0.551↓	0.004↑
Model 3		✓	✓	21.10 ± 0.540↓	0.003↓
Model 4	✓	✓		21.69 ± 0.635↓	0.003↓
Model 5	✓		✓	24.02 ± 0.669↓	0.004↓
Model 6	✓			44.90 ± 0.743↓	0.012 ± 0.001↓
Model 7			✓	46.14 ± 1.108↓	0.013 ± 0.001↓

### Semantic segmentation network validation

To fully prove that the generated root system architecture can be used for dataset enhancement, this paper uses the For Improved_UNet mentioned in the “Train_Val dataset” section for training, and uses the Generalization evaluation mentioned in the “Test dataset” section for testing. Including the improved model 1 of CycleGAN, a total of 22,000 generated in situ root images were generated, together with 6,600 in situ root images. At the same time, we split 2,200 in situ root images as in situ root image_small, aiming to simulate the situation of insufficient data volume. To keep the proportion of the dataset consistent, we split the generated root image into 7,334 generated root image_small. The training was conducted through the Improved_UNet [35]. The training set: validation set = 8:2. Finally, the test dataset mentioned in the “Postprocessing dataset” section is verified. The names of various weights are shown in Table 7. The comparison of the networks performance is shown in the Table 8.

**Table 7.** Weight names and datasets used

Weight	Dataset
*W_s_*	In situ root image_small (2,200 pics)
*W_n_*	In situ root image (6,600 pics)
*W_gs_*	Generated root image_small (7,334 pics)
*W_gn_*	Generated root image (22,000 pics)

**Table 8.** Comparison of network performance between 2 datasets (the number after ± represents the confidence interval at 99.5% confidence level)

Dataset	Weight	mIOU	F1	Accuracy
Performance evaluation (25 pics)	*W_s_*	83.03±0.81	90.12±0.52	98.80±0.18
*W_s_* + *W_gs_*	83.20±2.18	90.13±1.48	98.96±0.15
*W_n_*	86.08±0.95	92.05±0.62	99.08±0.16
*W_n_* + *W_gn_*	86.71±0.93	92.46±0.60	99.12±0.16
Generalization evaluation (2,227 pics)	*W_s_*	61.04±1.17	73.87±0.86	82.04±1.38
*W_s_* + *W_gs_*	92.56±0.19	95.98±0.11	99.34±0.03
*W_n_*	61.05±0.57	69.06±0.68	96.94±0.08
*W_n_* + *W_gn_*	94.65±0.12	97.17±0.07	99.56±0.01

The results show that when the original image and the enhanced image are kept at a certain ratio (in this experiment, the ratio between the 2 is 3:10), the network performance will be enhanced (in 2,200 in situ root image_small, mIOU is enhanced by 0.17 %, F1 is enhanced by 0.01%, and accuracy is enhanced by 0.16%; in 6,600 in situ root images, mIOU is enhanced by 0.63%, F1 is enhanced by 0.41%, and accuracy is enhanced by 0.04%). At the same time, the generalization performance of the enhanced dataset is also greatly enhanced (in 2,200 in situ root images_small, mIOU is enhanced by 31.52%, F1 is enhanced by 22.11%, and accuracy is enhanced by 17.3%; in 6,600 in situ root images, mIOU is enhanced by 33.6%, F1 is enhanced by 28.11%, and accuracy is enhanced by 2.62%).

The enhanced dataset improves both network performance and generalization performance. Among them, the relatively minor improvement in network performance may be attributed to the fact that the augmented dataset does not use consistent soil from the original images but rather a mixture of various soils. This is akin to adding 5 additional background categories when inputting the network. Similar to transfer learning, when transferring categories, there is not a substantial improvement in the recognition of the original categories, and, in some cases, it may even decline [40]. In addition, the size of the dataset is a crucial factor. A substantial disparity in the quantity between the original dataset and the augmented dataset can lead to class imbalance, thereby affecting network performance.

### Time analysis and comparison

The annotation of the dataset in this experiment was completed by Photoshop’s lasso tool. Constrained by the ultrahigh resolution, intricate root structures, and the annotator’s conditions, the average annotation time for in situ root images is approximately 4.5 h.

The time to generate the in situ root images through the dataset is divided into the time for CycleGAN to generate the root texture and the time for postprocessing. Among them, it takes about 3.91 min to generate 2,200 root textures with a resolution of 256 × 256 pixels (which can be spliced into a complete in situ root image). The running time of postprocessing is shown in Table 5. When converted to 2,200 pictures, it takes about 5.65 min.

If no annotations are provided in the original dataset, segmentation can be initially performed using a segmentation network. The segmented results can then be fed into CycleGAN for prediction, ultimately achieving the generation of in situ root images.

We also compared the segmentation speeds of Improved_UNet and RootPainter. The training process of Improved_UNet is as described in the “Comparison of generator improvement methods” section. RootPainter uses 219 in situ root images of 512 × 512 pixels for training, and the input image resolution was 600 × 600 pixels (as dictated by RootPainter, with a minimum of 600 × 600 pixels). Batch size was set to 4. Finally, 219 root system images were annotated. Until the end of training 60 generations, Improved_UNet took about 0.75 min to segment a picture, and RootPainter was used to segment 408 pictures with a resolution of 600 × 600pixels (which can be spliced into a complete in situ root image), which took about 2.1 min.

In general, it takes approximately 10.31 min to generate a complete in situ root image through CycleGAN, representing a substantial reduction compared to the annotation time.

### Experimental basis

The experimental idea of this paper is influenced by the method of generating simulated images on the Digital Plant Phenotyping Platform (D3P). By inputting parameters, the target plant is generated, then the light and soil are added, and, finally, the plant leaf texture is added to generate the simulation image. Put real images into the CycleGAN network for training, which can enhance the dataset of real images [41,42]. However, since there is no root simulation in D3P, this paper made changes in the experimental steps. First, the real root and mask images are trained through CycleGAN to generate root texture; second, a postprocessing step is added to unify the background pixels; finally, shadow and soil background are added to realize the generation of in situ root image.

### Future outlook

The method of generating a root extended dataset through CycleGAN has been showed to be feasible in this experiment, but there are still some defects, such as the instability of the black background pixel value generated by the network and the high degree of texture repetition in the root system architecture. One of the reasons that can be explained at present may be the asymmetric training of CycleGAN. Mapping the mask of thick roots to the fine-rooted texture will make the generated coarse-rooted texture more repetitive, while mapping the full-black background to the rooted image will make the pixel value where the full-black background should be generated unstable. At present, the method to solve this problem is to reduce the number of all-black background in the training set and increase the proportion of thick roots. This may solve the above problems slightly.

Adding shadow to the soil near the root system architecture can simulate the deeper soil displaced by the root system architecture to a certain extent, but it is still different from the real in situ root system architecture image. In the future, we will use the generated GRS image to process the root mask as a normal map. Finally, a more realistic in situ root parallax map is generated.

At present, conditional random fields (CRFs) are very popular in the field of deep learning. In terms of semantic segmentation, the UNet network [43] added to CRF improves the accuracy of semantic segmentation tasks. At the same time, the convolutional neural network with Markov random field [44] has a better effect in realizing texture migration. In the future, we will add CRF to CycleGAN for postprocessing to achieve a better root formation effect.

## Conclusion

In this paper, the expansion strategy of the in situ root dataset based on improved generator CycleGAN is proposed, and the root background unified postprocessing method based on spatial coordinates is proposed, which solves the problem that the background pixels are different because of the errors generated by the network, so it is difficult to segment the root. Compared with the traditional threshold segmentation method, this method has better accuracy and stability. At the same time, through time-division acquisition, this experiment realized the replacement of a variety of culture medium in the in situ root images and enhanced the universality of the dataset. After the verification of the Improved_UNet network, the enhanced dataset in network performance comparison is increased by 0.63% in mIOU, 0.41% in F1, and 0.04% in accuracy, and that in generalization performance comparison is increased by 33.6% in mIOU, 28.11% in F1, and 2.62% in accuracy.

## Data Availability

The model and dataset have been uploaded to Zenodo: https://doi.org/10.5281/zenodo.10460303. The code has been uploaded to GitHub: https://github.com/jiwd123/improved_cyclegan

## Supplementary Materials

Supplementary 1 The network and corresponding weights can be viewed on GitHub (https://github.com/jiwd123/improved_cyclegan) and Zenodo (https://doi.org/10.5281/zenodo.10460303).
