# Supplementary material for: In Situ Root Dataset Expansion Strategy Based on an Improved CycleGAN Generator
Source: Plant Phenomics. 2024 Feb 12;6:0148. doi: 10.34133/plantphenomics.0148 (PMC11020132; doi:10.34133/plantphenomics.0148)
Supplement: Supplementary 1 — The network and corresponding weights can be viewed on GitHub (https://github.com/jiwd123/improved_cyclegan) and Zenodo (https://doi.org/10.5281/zenodo.10460303). [file plantphenomics.0148.f1.zip › generalization evaluation_Wn+Wgn.pdf]

No.	IOU	Recall	Precision	Accuracy	F1
1	96.79	99.23	97.50	99.66	98.36
2	96.54	98.90	97.56	99.66	98.22
3	97.22	98.78	98.37	99.69	98.58
4	96.32	98.81	97.41	99.52	98.11
5	96.24	98.92	97.21	99.74	98.06
6	95.76	99.33	96.37	99.63	97.82
7	95.86	98.32	97.39	99.65	97.85
8	97.01	99.75	97.25	99.75	98.48
9	97.95	99.44	98.49	99.66	98.96
10	97.76	99.53	98.20	99.79	98.86
11	94.73	95.51	99.08	99.80	97.26
12	97.74	98.56	99.13	99.82	98.85
13	95.69	97.84	97.68	99.56	97.76
14	94.41	95.56	98.63	99.86	97.07
15	97.34	99.19	98.11	99.67	98.64
16	97.23	99.31	97.87	99.66	98.59
17	95.46	97.04	98.22	99.80	97.63
18	95.86	99.29	96.49	99.72	97.87
19	96.81	97.59	99.14	99.91	98.36
20	95.17	98.62	96.39	99.60	97.49
21	94.98	99.00	95.86	99.49	97.40
22	95.63	98.07	97.37	99.77	97.72
23	92.84	93.85	98.72	99.68	96.23
24	96.87	99.43	97.39	99.92	98.40
25	96.64	98.80	97.75	99.60	98.27
26	96.00	97.59	98.28	99.42	97.93
27	97.75	99.68	98.06	99.76	98.86
28	97.81	98.90	98.86	99.75	98.88
29	96.57	98.14	98.33	99.54	98.23
30	97.10	98.31	98.72	99.64	98.51
31	96.62	97.70	98.84	99.53	98.27
32	96.92	98.77	98.07	99.55	98.42
33	96.54	99.32	97.16	99.75	98.23
34	96.84	99.73	97.09	99.67	98.39
35	98.41	99.18	99.21	99.83	99.19
36	98.41	99.30	99.09	99.83	99.19
37	98.51	99.30	99.19	99.83	99.25
38	98.46	99.24	99.20	99.79	99.22
39	98.40	99.13	99.25	99.81	99.19
40	98.22	98.78	99.42	99.79	99.10
41	98.24	99.47	98.76	99.78	99.11
42	97.41	99.58	97.80	99.75	98.68
43	95.45	96.98	98.28	99.67	97.63
44	96.01	97.33	98.55	99.79	97.93
45	95.53	97.73	97.61	99.69	97.67
46	97.10	99.75	97.34	99.76	98.53
47	97.62	99.83	97.79	99.80	98.80
48	97.53	98.47	99.00	99.89	98.73
49	95.39	96.49	98.75	99.73	97.61
50	95.50	98.63	96.71	99.70	97.66
51	95.87	97.56	98.14	99.75	97.85
52	96.00	97.82	98.02	99.73	97.92
53	92.73	93.93	98.53	99.40	96.17
54	93.72	94.68	98.83	99.72	96.71
55	96.29	97.23	98.95	99.89	98.08
56	93.62	94.83	98.53	99.72	96.65
57	95.18	96.38	98.62	99.77	97.49
58	96.27	97.80	98.33	99.82	98.07
59	96.41	97.77	98.52	99.80	98.14
60	95.04	96.80	98.02	99.71	97.40
61	96.90	99.51	97.35	99.85	98.42

62	97.22	99.43	97.75	99.71	98.59
63	98.38	99.20	99.16	99.82	99.18
64	97.20	99.64	97.54	99.68	98.58
65	97.74	99.79	97.95	99.70	98.86
66	96.03	99.06	96.87	99.77	97.95
67	98.09	99.43	98.63	99.77	99.03
68	98.51	99.27	99.22	99.83	99.24
69	98.16	99.41	98.73	99.77	99.07
70	97.46	98.40	99.00	99.75	98.70
71	95.96	96.88	98.97	99.73	97.92
72	95.53	98.06	97.27	99.71	97.67
73	97.71	98.71	98.96	99.91	98.83
74	96.50	98.59	97.80	99.85	98.19
75	93.98	95.97	97.66	99.84	96.81
76	94.67	95.94	98.53	99.67	97.22
77	95.66	97.28	98.20	99.69	97.74
78	94.75	96.14	98.38	99.81	97.25
79	95.75	97.30	98.28	99.81	97.79
80	95.08	97.38	97.46	99.75	97.42
81	93.58	95.21	98.03	99.73	96.60
82	92.43	93.70	98.38	99.67	95.98
83	95.81	96.32	99.42	99.88	97.84
84	93.35	97.16	95.74	99.82	96.45
85	95.57	96.07	99.43	99.82	97.72
86	96.28	97.38	98.79	99.69	98.08
87	96.17	97.78	98.25	99.82	98.01
88	96.12	97.55	98.42	99.84	97.99
89	96.97	98.81	98.07	99.93	98.44
90	96.82	97.87	98.86	99.86	98.36
91	97.96	99.26	98.66	99.92	98.96
92	97.00	98.82	98.10	99.78	98.46
93	97.39	98.56	98.77	99.91	98.66
94	94.40	95.59	98.58	99.82	97.06
95	93.92	95.34	98.29	99.80	96.80
96	95.17	97.36	97.57	99.77	97.47
97	96.53	97.21	99.25	99.88	98.22
98	94.43	96.48	97.65	99.67	97.07
99	96.01	97.37	98.50	99.73	97.93
100	95.40	96.36	98.91	99.67	97.62
101	95.35	96.36	98.85	99.75	97.59
102	95.81	98.01	97.64	99.70	97.82
103	97.02	99.01	97.94	99.78	98.47
104	97.50	98.80	98.63	99.83	98.72
105	94.48	94.87	99.54	99.80	97.15
106	95.24	96.05	99.06	99.83	97.53
107	93.68	95.25	98.11	99.80	96.66
108	95.47	96.26	99.09	99.86	97.65
109	96.32	99.35	96.90	99.90	98.11
110	95.49	97.03	98.28	99.67	97.65
111	93.03	93.68	99.18	99.78	96.35
112	96.84	99.21	97.57	99.61	98.38
113	97.03	98.96	98.01	99.63	98.48
114	97.75	99.09	98.62	99.86	98.85
115	98.08	99.11	98.94	99.93	99.02
116	94.63	98.76	95.68	99.79	97.19
117	92.50	94.82	97.16	99.65	95.98
118	95.66	99.22	96.35	99.62	97.77
119	96.81	99.01	97.73	99.58	98.37
120	97.46	98.55	98.85	99.74	98.70
121	96.92	99.22	97.64	99.68	98.42
122	97.61	99.09	98.47	99.80	98.78
123	96.89	99.51	97.34	99.71	98.41

124	96.34	99.56	96.74	99.77	98.13
125	97.81	99.22	98.55	99.92	98.88
126	96.68	98.62	97.95	99.92	98.29
127	95.29	97.84	97.24	99.77	97.54
128	96.37	97.78	98.46	99.91	98.12
129	94.73	98.09	96.39	99.74	97.23
130	95.57	97.46	97.92	99.78	97.69
131	97.61	98.33	99.23	99.93	98.78
132	96.73	99.45	97.23	99.71	98.33
133	95.90	99.28	96.54	99.69	97.89
134	97.38	99.61	97.75	99.69	98.67
135	97.32	99.26	98.01	99.66	98.63
136	96.86	99.56	97.27	99.63	98.40
137	96.77	99.55	97.19	99.65	98.35
138	96.72	99.56	97.14	99.54	98.33
139	96.70	99.01	97.62	99.50	98.31
140	96.91	99.22	97.64	99.47	98.43
141	96.79	98.09	98.61	99.56	98.35
142	96.26	97.27	98.88	99.87	98.07
143	94.97	98.77	96.03	99.71	97.38
144	95.67	96.89	98.64	99.75	97.75
145	95.44	99.11	96.22	99.74	97.64
146	96.80	99.08	97.65	99.76	98.36
147	95.06	97.50	97.34	99.25	97.42
148	96.88	99.49	97.35	99.51	98.41
149	97.66	99.65	97.99	99.72	98.82
150	98.46	99.34	99.10	99.91	99.22
151	95.90	98.86	96.93	99.66	97.88
152	96.09	98.34	97.61	99.49	97.98
153	98.49	99.30	99.17	99.83	99.24
154	98.03	99.34	98.66	99.76	99.00
155	93.84	95.92	97.58	99.58	96.74
156	97.47	98.91	98.50	99.60	98.71
157	97.22	99.62	97.58	99.55	98.59
158	96.97	99.68	97.28	99.64	98.46
159	97.49	99.42	98.04	99.73	98.72
160	96.62	99.24	97.32	99.54	98.27
161	96.11	98.59	97.40	99.59	97.99
162	96.53	98.50	97.92	99.75	98.21
163	97.58	98.74	98.79	99.76	98.76
164	97.86	98.99	98.83	99.85	98.91
165	96.49	99.45	96.99	99.69	98.21
166	94.16	97.09	96.73	99.73	96.91
167	97.74	98.71	98.98	99.80	98.85
168	97.35	98.73	98.56	99.80	98.64
169	95.80	99.04	96.67	99.42	97.84
170	96.97	99.42	97.51	99.58	98.46
171	97.82	99.63	98.17	99.70	98.89
172	98.31	99.52	98.77	99.80	99.15
173	98.77	99.41	99.35	99.93	99.38
174	95.61	97.97	97.45	99.59	97.71
175	95.26	98.19	96.89	99.30	97.53
176	96.84	99.04	97.72	99.67	98.38
177	95.70	96.64	98.93	99.84	97.77
178	97.02	97.60	99.37	99.86	98.47
179	96.68	98.37	98.20	99.75	98.29
180	96.17	96.77	99.34	99.71	98.04
181	96.92	98.89	97.95	99.74	98.42
182	96.36	99.13	97.14	99.73	98.13
183	97.01	97.64	99.31	99.89	98.47
184	95.87	98.07	97.64	99.71	97.85
185	95.84	96.48	99.29	99.71	97.86

186	95.55	95.97	99.51	99.82	97.71
187	98.17	98.99	99.15	99.88	99.07
188	95.41	98.39	96.82	99.85	97.60
189	96.59	99.28	97.25	99.71	98.25
190	95.48	96.28	99.08	99.88	97.66
191	96.93	98.69	98.15	99.88	98.42
192	97.06	99.13	97.87	99.69	98.50
193	97.03	98.53	98.42	99.69	98.48
194	96.72	97.55	99.09	99.87	98.31
195	95.54	96.82	98.56	99.70	97.68
196	95.88	99.08	96.71	99.62	97.88
197	95.71	96.84	98.73	99.80	97.77
198	96.79	98.23	98.46	99.80	98.34
199	92.90	94.35	98.22	99.51	96.25
200	96.44	97.43	98.91	99.77	98.17
201	97.47	99.05	98.38	99.77	98.71
202	97.55	98.15	99.36	99.77	98.75
203	92.01	92.61	99.23	99.69	95.80
204	94.46	95.32	98.98	99.59	97.12
205	94.79	96.14	98.45	99.68	97.28
206	92.86	94.69	97.75	99.67	96.20
207	86.21	89.68	94.73	99.50	92.13
208	96.81	97.52	99.22	99.87	98.36
209	96.41	98.73	97.57	99.57	98.15
210	96.75	97.75	98.91	99.84	98.33
211	96.01	98.65	97.23	99.67	97.93
212	96.45	99.03	97.33	99.87	98.17
213	97.58	98.58	98.95	99.88	98.76
214	96.48	98.11	98.24	99.77	98.18
215	96.47	98.26	98.09	99.75	98.18
216	98.02	98.72	99.26	99.95	98.99
217	97.18	98.63	98.48	99.77	98.55
218	96.99	98.35	98.55	99.75	98.45
219	97.37	98.80	98.51	99.77	98.65
220	97.77	98.79	98.93	99.80	98.86
221	95.96	97.87	97.93	99.73	97.90
222	93.47	95.98	97.06	99.79	96.52
223	94.16	95.90	97.98	99.61	96.93
224	95.29	96.97	98.12	99.62	97.54
225	94.25	97.15	96.78	99.69	96.96
226	93.88	96.03	97.52	99.40	96.77
227	93.95	96.39	97.22	99.35	96.81
228	90.79	94.54	95.37	99.66	94.95
229	95.37	97.28	97.89	99.74	97.58
230	95.31	96.98	98.14	99.49	97.56
231	95.95	97.57	98.23	99.75	97.90
232	93.19	94.72	98.13	99.64	96.39
233	94.98	96.93	97.81	99.66	97.37
234	94.87	97.39	97.20	99.84	97.30
235	97.08	98.68	98.32	99.77	98.50
236	96.71	99.12	97.52	99.74	98.31
237	97.05	98.72	98.24	99.87	98.48
238	97.79	98.74	99.00	99.83	98.87
239	95.70	98.25	97.29	99.69	97.76
240	94.05	95.44	98.36	99.56	96.88
241	95.85	96.67	99.08	99.74	97.86
242	94.97	98.04	96.70	99.66	97.37
243	94.23	97.00	96.92	99.28	96.96
244	96.90	98.64	98.16	99.91	98.40
245	90.48	91.54	98.75	98.74	95.01
246	94.02	95.02	98.79	99.80	96.87
247	96.41	98.35	97.92	99.85	98.14

248	93.09	96.18	96.45	99.47	96.31
249	90.07	95.92	93.19	99.61	94.53
250	89.55	94.35	94.04	99.61	94.19
251	94.59	95.85	98.51	99.80	97.16
252	93.92	97.47	96.09	99.77	96.77
253	93.11	94.62	98.13	99.78	96.34
254	93.54	94.64	98.66	99.69	96.61
255	95.61	96.62	98.85	99.87	97.72
256	94.72	95.78	98.76	99.68	97.25
257	94.45	97.49	96.66	99.68	97.08
258	91.47	94.88	95.86	99.47	95.37
259	94.64	99.06	95.43	99.78	97.21
260	93.26	93.77	99.42	99.43	96.51
261	94.47	95.69	98.56	99.77	97.10
262	95.60	96.02	99.51	99.88	97.74
263	90.75	96.25	93.74	98.99	94.98
264	94.17	96.56	97.30	99.17	96.93
265	94.45	96.22	97.96	99.59	97.08
266	98.07	99.18	98.85	99.88	99.02
267	95.78	97.47	98.14	99.72	97.80
268	90.65	93.74	96.12	99.00	94.92
269	87.17	90.58	95.06	99.36	92.76
270	97.21	97.62	99.56	99.91	98.58
271	95.55	98.64	96.75	99.86	97.68
272	94.46	96.32	97.85	99.75	97.08
273	94.52	96.44	97.81	99.50	97.12
274	93.10	96.15	96.48	99.53	96.31
275	93.30	93.82	99.36	99.76	96.51
276	94.41	94.85	99.48	99.80	97.11
277	96.46	99.56	96.86	99.67	98.19
278	91.13	93.25	97.23	99.67	95.20
279	93.53	96.74	96.37	99.69	96.55
280	95.44	96.61	98.68	99.66	97.63
281	94.63	97.25	97.10	99.50	97.18
282	92.84	94.79	97.61	99.64	96.18
283	89.21	91.72	96.52	99.51	94.06
284	93.94	94.61	99.19	99.77	96.84
285	92.89	94.69	97.79	99.69	96.21
286	93.35	95.11	97.87	99.76	96.47
287	96.29	96.95	99.27	99.80	98.10
288	95.52	98.60	96.77	99.56	97.68
289	95.19	96.50	98.52	99.40	97.50
290	94.01	95.57	98.18	99.39	96.86
291	94.94	95.60	99.22	99.85	97.38
292	97.19	98.31	98.80	99.90	98.56
293	94.99	98.52	96.28	99.66	97.39
294	91.56	94.43	96.48	99.15	95.45
295	90.53	91.93	98.15	99.29	94.94
296	86.08	87.54	97.87	98.80	92.42
297	97.11	98.01	99.03	99.85	98.52
298	96.19	99.48	96.65	99.88	98.04
299	96.06	97.94	97.96	99.72	97.95
300	97.54	98.22	99.28	99.91	98.74
301	95.31	96.43	98.70	99.85	97.56
302	95.51	97.37	97.94	99.86	97.65
303	90.38	90.88	99.34	99.64	94.92
304	91.09	95.19	95.06	99.69	95.12
305	95.15	96.89	98.05	99.58	97.47
306	95.34	97.00	98.15	99.64	97.57
307	84.14	85.53	97.69	99.08	91.21
308	92.34	95.01	96.78	99.52	95.88
309	92.26	95.76	95.88	99.71	95.82

310	94.21	99.10	94.95	99.76	96.98
311	94.17	96.85	96.98	99.61	96.91
312	95.29	96.87	98.24	99.47	97.55
313	93.92	98.97	94.79	99.51	96.84
314	90.70	92.43	97.68	99.61	94.98
315	92.42	97.30	94.62	99.55	95.94
316	92.61	97.82	94.41	99.20	96.08
317	94.00	97.14	96.52	99.46	96.83
318	93.59	98.59	94.77	99.45	96.64
319	93.54	98.61	94.70	99.36	96.62
320	92.58	96.31	95.73	99.41	96.02
321	96.49	98.35	98.03	99.53	98.19
322	90.52	95.15	94.44	99.47	94.79
323	96.50	97.71	98.69	99.74	98.20
324	95.02	97.27	97.52	99.43	97.39
325	89.09	94.51	93.32	99.52	93.91
326	96.30	99.01	97.20	99.74	98.10
327	94.81	98.30	96.30	99.49	97.29
328	93.53	97.02	96.12	99.32	96.57
329	94.48	96.31	97.92	99.27	97.11
330	95.07	96.84	98.03	99.28	97.43
331	91.68	97.63	93.63	98.71	95.59
332	95.94	97.87	97.92	99.35	97.90
333	89.00	93.11	94.60	99.65	93.85
334	96.55	97.54	98.92	99.69	98.23
335	97.04	98.58	98.38	99.60	98.48
336	96.80	98.88	97.85	99.49	98.36
337	96.97	99.34	97.58	99.76	98.45
338	95.52	99.17	96.28	99.30	97.70
339	96.03	97.27	98.65	99.23	97.96
340	95.98	97.99	97.84	99.44	97.92
341	96.06	98.10	97.82	99.45	97.96
342	96.30	98.78	97.42	99.49	98.10
343	96.49	98.17	98.22	99.53	98.19
344	95.20	97.01	97.98	99.64	97.49
345	96.15	99.25	96.85	99.40	98.03
346	95.45	98.82	96.51	99.30	97.65
347	95.50	98.98	96.41	99.41	97.68
348	95.24	98.85	96.27	99.31	97.54
349	95.91	97.74	98.03	99.39	97.88
350	95.45	96.59	98.74	99.26	97.65
351	95.27	98.91	96.25	99.28	97.56
352	96.48	98.49	97.89	99.52	98.19
353	95.88	98.96	96.81	99.55	97.87
354	89.09	89.75	99.06	99.66	94.17
355	95.75	97.42	98.16	99.80	97.79
356	91.85	92.44	99.31	99.30	95.75
357	93.17	95.85	96.87	99.50	96.36
358	91.12	93.60	96.82	99.57	95.18
359	90.38	93.74	95.76	99.17	94.74
360	91.92	95.60	95.67	99.36	95.63
361	94.90	98.68	96.05	99.56	97.35
362	94.04	97.39	96.33	99.31	96.86
363	93.63	97.81	95.49	99.52	96.63
364	91.13	95.80	94.60	98.98	95.19
365	95.55	97.50	97.87	99.37	97.68
366	94.98	96.00	98.82	99.77	97.39
367	96.79	99.10	97.63	99.47	98.36
368	96.20	97.47	98.62	99.31	98.04
369	94.14	98.48	95.45	99.27	96.94
370	97.95	98.47	99.45	99.76	98.96
371	96.43	98.63	97.70	99.39	98.16

372	96.83	98.73	98.02	99.31	98.38
373	95.24	99.16	96.02	99.20	97.56
374	95.99	96.81	99.09	99.51	97.94
375	96.62	98.83	97.70	99.54	98.26
376	95.49	98.88	96.51	99.21	97.68
377	97.36	99.15	98.15	99.81	98.65
378	97.28	98.76	98.46	99.57	98.61
379	95.97	98.15	97.68	99.34	97.91
380	94.58	97.74	96.61	99.00	97.17
381	96.10	98.28	97.69	99.40	97.98
382	94.73	99.25	95.43	99.13	97.30
383	96.62	98.17	98.35	99.44	98.26
384	95.97	98.12	97.71	99.40	97.91
385	96.22	97.93	98.17	99.48	98.05
386	96.70	99.10	97.53	99.58	98.31
387	97.35	98.71	98.58	99.67	98.64
388	98.00	99.46	98.52	99.78	98.99
389	97.85	99.36	98.46	99.77	98.90
390	96.63	97.32	99.26	99.54	98.28
391	96.11	98.09	97.89	99.41	97.99
392	95.68	97.92	97.60	99.29	97.76
393	93.11	97.48	95.27	98.89	96.36
394	96.94	98.18	98.68	99.64	98.43
395	95.41	98.83	96.45	99.53	97.63
396	93.77	98.47	95.08	99.28	96.74
397	90.33	96.94	92.70	98.95	94.77
398	93.37	93.95	99.29	99.75	96.55
399	97.41	99.22	98.14	99.70	98.68
400	91.07	92.36	98.27	99.65	95.22
401	97.73	98.54	99.14	99.86	98.84
402	93.08	94.66	98.09	99.37	96.35
403	89.83	91.65	97.45	99.68	94.46
404	93.97	95.32	98.41	99.50	96.84
405	95.69	99.12	96.48	99.52	97.78
406	96.16	98.13	97.89	99.77	98.01
407	95.05	97.59	97.22	99.59	97.41
408	95.96	99.02	96.85	99.47	97.92
409	96.29	99.37	96.88	99.46	98.11
410	97.61	99.25	98.32	99.77	98.78
411	95.97	98.59	97.25	99.42	97.92
412	96.33	99.39	96.90	99.48	98.13
413	97.25	99.48	97.74	99.60	98.60
414	96.86	98.72	98.06	99.49	98.39
415	95.81	98.85	96.86	99.17	97.85
416	96.47	98.80	97.58	99.42	98.19
417	98.12	98.67	99.42	99.87	99.04
418	95.46	97.76	97.51	99.48	97.64
419	95.68	99.33	96.30	99.36	97.79
420	95.77	97.24	98.37	99.72	97.80
421	97.39	98.84	98.48	99.82	98.66
422	91.84	97.18	94.10	99.45	95.62
423	93.73	98.30	95.17	99.30	96.71
424	95.88	97.07	98.67	99.71	97.86
425	95.58	96.69	98.74	99.78	97.70
426	95.67	96.67	98.87	99.78	97.76
427	96.31	97.87	98.31	99.90	98.09
428	89.45	90.75	98.13	99.66	94.30
429	88.77	91.93	95.63	99.66	93.74
430	96.49	98.90	97.50	99.59	98.19
431	95.97	99.24	96.66	99.47	97.94
432	96.99	98.52	98.38	99.79	98.45
433	96.22	98.43	97.66	99.86	98.04

434	96.34	98.22	97.99	99.86	98.11
435	95.25	98.35	96.71	99.75	97.52
436	94.82	96.64	97.93	99.72	97.28
437	95.27	96.82	98.25	99.73	97.53
438	94.25	96.18	97.76	99.67	96.97
439	96.62	97.16	99.41	99.76	98.27
440	96.25	98.20	97.92	99.73	98.06
441	97.52	99.05	98.41	99.93	98.73
442	95.48	98.73	96.60	99.70	97.65
443	97.23	98.00	99.17	99.78	98.58
444	95.57	97.80	97.58	99.79	97.69
445	96.12	98.04	97.94	99.56	97.99
446	96.45	99.32	97.06	99.74	98.18
447	93.71	94.67	98.84	99.66	96.71
448	94.52	98.39	95.90	99.68	97.13
449	94.50	95.10	99.29	99.83	97.15
450	92.99	95.04	97.51	99.80	96.25
451	94.27	95.58	98.44	99.82	96.99
452	93.27	94.47	98.53	99.59	96.46
453	95.63	98.62	96.86	99.63	97.73
454	96.73	97.87	98.76	99.82	98.32
455	96.69	97.90	98.69	99.75	98.29
456	96.26	99.36	96.84	99.54	98.09
457	94.43	98.37	95.85	99.30	97.09
458	95.33	96.37	98.81	99.74	97.57
459	95.08	99.11	95.85	99.75	97.45
460	94.22	96.29	97.62	99.69	96.95
461	95.36	96.63	98.55	99.75	97.58
462	93.43	94.16	99.09	99.77	96.56
463	94.84	95.71	99.00	99.55	97.33
464	95.58	97.15	98.24	99.76	97.70
465	93.22	95.01	97.81	99.81	96.39
466	95.70	97.45	98.07	99.83	97.76
467	93.88	96.36	97.18	99.32	96.77
468	95.54	97.68	97.67	99.60	97.67
469	94.58	96.04	98.30	99.71	97.16
470	92.62	93.06	99.48	99.63	96.16
471	93.53	95.03	98.19	99.65	96.59
472	89.12	93.84	94.08	98.96	93.96
473	92.94	94.82	97.72	99.48	96.25
474	97.66	97.97	99.66	99.93	98.81
475	95.14	98.36	96.59	99.63	97.47
476	96.31	98.16	98.02	99.67	98.09
477	91.50	93.05	97.98	99.62	95.45
478	94.61	94.81	99.77	99.79	97.23
479	95.96	96.39	99.51	99.88	97.92
480	94.29	95.83	98.20	99.69	97.00
481	95.38	97.26	97.91	99.85	97.58
482	95.47	97.55	97.72	99.65	97.64
483	95.16	97.29	97.66	99.45	97.47
484	96.44	97.59	98.75	99.86	98.16
485	93.47	95.64	97.45	99.55	96.53
486	94.46	95.51	98.79	99.42	97.12
487	95.20	96.67	98.33	99.79	97.49
488	90.06	96.47	92.71	99.60	94.55
489	94.64	95.55	98.94	99.66	97.21
490	95.38	97.79	97.39	99.72	97.59
491	93.30	95.14	97.77	99.77	96.44
492	89.05	90.72	97.65	99.32	94.06
493	90.11	92.94	96.25	99.73	94.56
494	97.68	98.45	99.19	99.86	98.82
495	97.77	99.49	98.25	99.82	98.87

496	98.01	98.28	99.71	99.84	98.99
497	97.97	98.52	99.42	99.87	98.97
498	95.14	96.02	98.98	99.81	97.48
499	96.21	97.96	98.11	99.79	98.03
500	95.61	98.38	97.07	99.75	97.72
501	95.39	97.35	97.83	99.79	97.59
502	95.02	96.61	98.22	99.42	97.41
503	96.10	98.68	97.30	99.62	97.99
504	93.82	95.20	98.33	99.81	96.74
505	94.39	95.76	98.39	99.72	97.06
506	92.12	94.96	96.56	99.58	95.75
507	97.11	98.30	98.72	99.80	98.51
508	97.03	98.78	98.17	99.75	98.47
509	95.76	98.32	97.28	99.71	97.80
510	95.82	99.39	96.36	99.78	97.85
511	97.86	98.96	98.86	99.85	98.91
512	98.72	99.46	99.25	99.89	99.35
513	97.64	99.56	98.05	99.75	98.80
514	97.67	98.07	99.57	99.92	98.82
515	96.33	97.61	98.60	99.81	98.10
516	94.33	94.90	99.33	99.70	97.06
517	93.49	95.48	97.64	99.60	96.55
518	92.47	96.34	95.58	99.35	95.96
519	93.99	94.92	98.88	99.79	96.86
520	92.76	98.13	94.27	99.70	96.16
521	98.51	99.66	98.84	99.86	99.25
522	97.34	99.36	97.94	99.72	98.65
523	98.05	99.28	98.74	99.86	99.01
524	94.46	96.26	97.92	99.82	97.08
525	95.80	96.51	99.19	99.83	97.83
526	96.83	97.44	99.34	99.74	98.38
527	97.04	99.19	97.79	99.80	98.48
528	96.04	96.57	99.40	99.90	97.97
529	96.15	96.90	99.15	99.88	98.01
530	89.31	90.68	98.05	99.48	94.22
531	89.68	92.79	95.87	99.50	94.31
532	98.05	99.34	98.69	99.67	99.01
533	97.46	99.56	97.87	99.75	98.71
534	97.39	99.15	98.19	99.74	98.67
535	95.25	96.40	98.67	99.86	97.52
536	94.38	96.42	97.67	99.62	97.04
537	93.70	96.64	96.67	99.70	96.65
538	96.27	97.80	98.34	99.70	98.07
539	97.24	98.70	98.47	99.81	98.58
540	96.77	98.10	98.57	99.92	98.34
541	94.70	95.78	98.75	99.63	97.24
542	94.35	97.61	96.43	99.76	97.02
543	97.00	98.99	97.95	99.44	98.47
544	92.68	95.21	96.95	99.68	96.07
545	97.25	99.73	97.50	99.87	98.60
546	97.91	99.36	98.51	99.90	98.93
547	95.15	95.93	99.09	99.85	97.48
548	95.42	97.15	98.07	99.79	97.61
549	96.57	97.62	98.85	99.72	98.24
550	96.65	97.91	98.64	99.73	98.27
551	96.01	97.25	98.63	99.78	97.93
552	95.45	96.83	98.44	99.75	97.63
553	95.53	97.99	97.34	99.76	97.67
554	96.68	98.77	97.82	99.46	98.30
555	94.36	97.57	96.48	99.77	97.02
556	93.38	99.01	94.19	99.66	96.54
557	95.71	96.61	98.98	99.81	97.78

558	95.73	97.55	97.99	99.82	97.77
559	97.45	99.21	98.19	99.64	98.70
560	96.56	99.56	96.98	99.56	98.25
561	97.58	99.01	98.51	99.74	98.76
562	97.40	98.75	98.59	99.65	98.67
563	95.49	97.88	97.43	99.39	97.66
564	96.71	98.59	98.02	99.57	98.31
565	97.89	98.93	98.92	99.66	98.93
566	96.46	98.09	98.28	99.33	98.18
567	95.90	98.06	97.70	99.26	97.88
568	96.83	99.08	97.68	99.52	98.38
569	94.13	97.03	96.76	99.63	96.89
570	96.46	98.89	97.48	99.58	98.18
571	94.97	99.02	95.84	99.32	97.41
572	96.70	99.11	97.52	99.55	98.31
573	96.94	98.83	98.03	99.71	98.43
574	96.65	99.00	97.56	99.87	98.28
575	96.03	97.35	98.53	99.88	97.94
576	96.74	98.15	98.50	99.77	98.32
577	96.35	97.61	98.61	99.82	98.11
578	94.83	98.47	96.17	99.60	97.30
579	96.74	98.16	98.47	99.75	98.32
580	94.11	94.63	99.37	99.84	96.94
581	97.55	99.73	97.81	99.77	98.76
582	97.58	99.74	97.83	99.78	98.77
583	95.52	96.60	98.77	99.83	97.67
584	98.16	98.51	99.63	99.92	99.07
585	98.31	98.78	99.51	99.91	99.14
586	96.83	98.69	98.07	99.26	98.38
587	96.95	98.96	97.93	99.29	98.44
588	96.48	98.66	97.71	99.63	98.18
589	96.80	98.53	98.19	99.48	98.36
590	96.67	98.31	98.26	99.61	98.29
591	96.98	98.02	98.88	99.88	98.45
592	93.66	96.13	97.17	99.37	96.64
593	96.04	98.04	97.87	99.31	97.95
594	95.97	97.70	98.14	99.14	97.92
595	95.74	99.19	96.49	99.20	97.83
596	95.21	98.94	96.17	99.24	97.53
597	97.07	98.84	98.16	99.53	98.50
598	96.75	98.34	98.32	99.51	98.33
599	97.27	98.24	98.98	99.60	98.61
600	97.16	99.18	97.93	99.63	98.55
601	98.29	99.22	99.05	99.81	99.13
602	95.29	96.99	98.10	99.70	97.54
603	87.18	88.03	98.67	99.60	93.05
604	86.04	87.18	98.12	99.53	92.33
605	89.46	92.59	95.78	99.70	94.16
606	94.45	96.66	97.50	99.47	97.08
607	94.35	98.46	95.71	99.10	97.06
608	92.96	95.42	97.11	99.06	96.26
609	93.24	95.67	97.15	99.31	96.41
610	92.02	98.26	93.48	98.74	95.81
611	93.31	98.20	94.85	98.93	96.50
612	94.99	98.38	96.43	99.41	97.39
613	95.72	96.53	99.08	99.75	97.79
614	95.20	98.84	96.24	99.30	97.52
615	94.49	98.07	96.23	98.76	97.14
616	94.24	97.88	96.14	98.63	97.00
617	93.64	97.86	95.49	98.93	96.66
618	91.65	97.20	93.89	99.30	95.51
619	89.70	90.55	98.80	99.63	94.49

620	66.00	67.87	92.91	97.83	78.44
621	89.52	90.70	98.34	99.56	94.36
622	90.03	90.89	98.81	99.58	94.69
623	89.19	91.13	97.23	99.64	94.08
624	95.53	98.45	96.93	99.45	97.68
625	90.68	91.47	98.95	99.59	95.06
626	92.76	98.55	93.92	99.65	96.18
627	94.31	98.07	95.96	99.74	97.00
628	96.23	97.33	98.79	99.77	98.05
629	92.58	94.85	97.26	99.27	96.04
630	87.56	88.51	98.58	99.47	93.27
631	91.75	93.74	97.45	99.74	95.56
632	94.92	98.58	96.18	99.22	97.37
633	94.69	98.69	95.87	99.04	97.26
634	94.78	97.48	97.06	99.36	97.27
635	96.58	98.67	97.82	99.45	98.24
636	93.35	95.18	97.84	99.20	96.49
637	96.05	97.87	98.04	99.52	97.95
638	95.85	98.71	97.02	99.42	97.86
639	94.42	97.60	96.55	99.29	97.07
640	94.30	97.71	96.29	99.62	97.00
641	89.10	90.32	98.22	99.61	94.10
642	89.52	90.66	98.36	99.69	94.35
643	90.75	94.34	95.52	99.73	94.92
644	90.66	96.91	93.03	99.46	94.93
645	94.69	96.71	97.70	99.79	97.20
646	96.44	97.02	99.37	99.61	98.18
647	94.24	96.60	97.31	99.75	96.96
648	95.10	96.49	98.41	99.79	97.44
649	85.64	87.16	97.52	99.30	92.05
650	92.23	95.74	95.85	99.80	95.79
651	94.34	96.75	97.28	99.68	97.01
652	91.48	92.87	98.20	99.52	95.46
653	95.24	99.27	95.89	99.64	97.55
654	96.43	98.95	97.40	99.52	98.17
655	94.80	98.23	96.34	99.61	97.28
656	95.00	99.19	95.70	99.77	97.41
657	94.75	96.19	98.33	99.80	97.25
658	96.55	98.16	98.28	99.69	98.22
659	96.17	98.72	97.33	99.70	98.02
660	95.27	98.93	96.20	99.82	97.54
661	95.28	99.00	96.15	99.78	97.55
662	96.71	97.48	99.16	99.86	98.31
663	97.38	98.68	98.63	99.80	98.65
664	95.87	96.74	99.02	99.73	97.87
665	93.93	94.58	99.22	99.76	96.84
666	91.55	98.15	92.96	99.66	95.48
667	94.51	96.87	97.34	99.62	97.11
668	92.66	96.66	95.46	99.77	96.06
669	95.08	98.08	96.79	99.51	97.43
670	92.94	95.02	97.50	99.59	96.24
671	96.29	99.01	97.19	99.79	98.09
672	97.00	99.03	97.90	99.55	98.46
673	97.56	98.73	98.78	99.75	98.76
674	96.02	98.64	97.25	99.76	97.94
675	95.68	97.97	97.54	99.52	97.76
676	96.76	98.59	98.08	99.69	98.33
677	96.63	96.82	99.80	99.89	98.29
678	96.90	98.56	98.25	99.74	98.41
679	97.64	98.06	99.55	99.89	98.80
680	95.96	98.55	97.27	99.83	97.91
681	96.68	98.66	97.93	99.75	98.29

682	96.61	98.17	98.33	99.78	98.25
683	93.34	93.93	99.29	99.68	96.53
684	76.06	77.84	95.58	98.86	85.80
685	92.27	95.29	96.39	99.42	95.84
686	93.10	97.36	95.31	99.56	96.32
687	89.26	90.09	98.83	99.57	94.26
688	94.66	97.46	96.91	99.83	97.18
689	95.22	96.61	98.42	99.84	97.51
690	96.50	98.56	97.84	99.71	98.20
691	96.36	99.37	96.94	99.55	98.14
692	96.63	98.02	98.52	99.49	98.27
693	96.95	98.55	98.31	99.89	98.43
694	96.53	97.81	98.61	99.77	98.21
695	95.96	97.91	97.90	99.60	97.91
696	96.49	97.75	98.64	99.76	98.19
697	96.71	97.53	99.10	99.92	98.31
698	96.47	97.54	98.83	99.84	98.18
699	74.53	77.68	92.42	97.73	84.41
700	84.65	88.98	93.39	98.80	91.13
701	96.85	97.09	99.74	99.84	98.40
702	94.97	95.61	99.25	99.87	97.39
703	90.16	91.20	98.54	99.70	94.73
704	95.65	97.62	97.83	99.85	97.73
705	96.11	98.26	97.71	99.86	97.98
706	97.00	98.65	98.27	99.69	98.46
707	95.88	98.74	97.02	99.48	97.87
708	95.67	98.69	96.84	99.59	97.76
709	96.42	99.22	97.13	99.61	98.17
710	97.63	98.70	98.87	99.90	98.79
711	95.49	96.34	99.03	99.80	97.67
712	95.37	95.68	99.64	99.84	97.62
713	96.09	97.41	98.54	99.75	97.97
714	95.38	96.32	98.91	99.86	97.60
715	95.68	98.34	97.17	99.80	97.75
716	94.74	96.02	98.51	99.74	97.25
717	92.52	94.42	97.63	99.72	96.00
718	89.36	94.24	93.93	99.41	94.08
719	89.64	92.39	96.32	99.38	94.31
720	89.01	94.51	93.27	99.14	93.89
721	88.74	93.91	93.47	99.46	93.69
722	94.96	95.81	99.00	99.86	97.38
723	96.33	97.34	98.88	99.78	98.10
724	96.11	97.14	98.85	99.75	97.99
725	95.01	97.01	97.76	99.74	97.38
726	90.80	91.60	98.94	99.58	95.13
727	91.24	92.69	98.09	99.57	95.31
728	91.56	94.12	96.77	99.65	95.43
729	89.30	91.03	97.53	99.64	94.17
730	96.11	98.09	97.87	99.74	97.98
731	96.10	98.20	97.77	99.58	97.98
732	95.89	98.27	97.47	99.55	97.87
733	95.34	99.45	95.83	99.68	97.60
734	94.02	95.96	97.72	99.82	96.83
735	96.51	98.56	97.84	99.78	98.20
736	95.96	97.16	98.67	99.76	97.91
737	95.40	97.51	97.69	99.71	97.60
738	95.06	97.63	97.20	99.58	97.41
739	96.12	99.20	96.84	99.68	98.00
740	96.40	98.36	97.91	99.82	98.14
741	88.36	95.73	91.38	99.62	93.50
742	93.63	96.10	97.15	99.43	96.62
743	93.17	96.63	96.09	99.27	96.36

744	91.77	95.84	95.25	99.47	95.54
745	93.21	95.60	97.16	99.66	96.38
746	97.27	99.06	98.14	99.80	98.60
747	91.73	95.60	95.42	99.62	95.51
748	89.88	96.70	92.32	99.55	94.46
749	75.92	77.97	95.04	98.60	85.66
750	74.73	76.76	94.87	98.48	84.86
751	92.04	94.72	96.71	99.59	95.70
752	91.12	94.34	95.99	99.50	95.16
753	91.75	94.99	96.08	99.45	95.53
754	92.19	94.58	97.06	99.60	95.80
755	95.46	98.88	96.45	99.68	97.65
756	96.08	99.22	96.79	99.60	97.99
757	91.54	92.10	99.26	99.73	95.55
758	97.13	98.90	98.16	99.78	98.53
759	96.33	98.02	98.18	99.67	98.10
760	95.11	98.52	96.41	99.54	97.46
761	92.04	94.14	97.39	99.42	95.74
762	91.74	94.78	96.27	99.64	95.52
763	92.44	93.79	98.29	99.64	95.99
764	92.43	94.75	97.15	99.69	95.94
765	90.47	92.18	97.66	99.69	94.84
766	96.07	97.40	98.53	99.90	97.96
767	96.56	97.87	98.57	99.92	98.22
768	94.26	96.35	97.60	99.68	96.97
769	94.95	97.23	97.46	99.78	97.35
770	94.92	97.08	97.59	99.64	97.34
771	93.95	94.80	98.97	99.73	96.84
772	94.86	97.16	97.44	99.64	97.30
773	94.46	97.21	96.96	99.48	97.09
774	95.55	96.39	99.05	99.76	97.70
775	95.27	98.55	96.53	99.87	97.53
776	96.97	98.88	98.01	99.88	98.44
777	96.22	98.55	97.55	99.70	98.05
778	95.19	99.05	96.04	99.51	97.52
779	92.67	94.70	97.49	99.76	96.08
780	95.50	98.17	97.15	99.62	97.66
781	95.32	96.50	98.66	99.74	97.57
782	95.90	97.44	98.30	99.72	97.87
783	95.59	96.40	99.09	99.73	97.72
784	97.03	98.05	98.90	99.89	98.47
785	96.52	98.33	98.07	99.72	98.20
786	97.38	98.65	98.67	99.78	98.66
787	96.23	98.71	97.40	99.76	98.05
788	97.44	98.21	99.17	99.85	98.69
789	91.23	96.62	93.94	99.15	95.26
790	95.53	98.70	96.67	99.86	97.67
791	92.45	96.79	95.14	99.26	95.96
792	92.96	96.04	96.43	99.55	96.23
793	91.83	93.86	97.44	99.58	95.61
794	89.06	93.48	94.32	99.37	93.90
795	90.18	93.72	95.49	99.43	94.60
796	76.59	78.60	95.30	98.61	86.15
797	76.75	78.62	96.09	98.24	86.48
798	89.57	91.87	96.81	99.62	94.28
799	91.66	93.03	98.21	99.70	95.55
800	94.58	95.14	99.33	99.83	97.19
801	96.63	97.32	99.24	99.85	98.27
802	96.58	98.58	97.90	99.65	98.24
803	96.13	98.80	97.22	99.47	98.01
804	96.38	99.14	97.16	99.51	98.14
805	95.98	98.45	97.39	99.59	97.92

806	93.55	97.12	96.02	99.71	96.57
807	91.62	93.03	98.16	99.73	95.52
808	93.21	94.17	98.80	99.83	96.43
809	94.60	96.20	98.15	99.79	97.16
810	94.59	98.71	95.69	99.67	97.18
811	94.99	98.75	96.09	99.41	97.40
812	97.00	97.33	99.65	99.71	98.48
813	95.50	97.75	97.57	99.19	97.66
814	95.13	98.89	96.12	99.32	97.48
815	93.50	94.23	99.12	99.57	96.61
816	94.57	97.87	96.45	99.36	97.15
817	94.37	98.38	95.77	99.40	97.06
818	95.34	98.63	96.55	99.67	97.58
819	96.39	98.23	98.04	99.65	98.14
820	96.93	97.66	99.21	99.69	98.43
821	96.60	98.63	97.87	99.76	98.25
822	96.86	98.05	98.72	99.84	98.38
823	97.59	98.64	98.90	99.87	98.77
824	94.44	99.00	95.32	99.35	97.12
825	95.84	97.77	97.92	99.56	97.84
826	93.64	96.58	96.68	99.24	96.63
827	95.42	98.57	96.69	99.71	97.62
828	93.33	95.06	97.90	99.83	96.46
829	96.60	97.69	98.81	99.84	98.25
830	96.01	97.47	98.41	99.64	97.93
831	96.01	98.27	97.60	99.67	97.93
832	95.37	98.38	96.79	99.78	97.58
833	97.14	99.25	97.83	99.92	98.53
834	90.96	95.17	94.92	99.64	95.05
835	97.08	98.22	98.78	99.77	98.50
836	89.01	92.06	95.83	99.49	93.91
837	91.01	95.39	94.76	99.73	95.08
838	88.47	89.29	98.77	99.65	93.79
839	85.91	91.18	92.54	99.47	91.85
840	89.59	90.66	98.50	99.58	94.42
841	90.23	92.66	96.75	99.69	94.66
842	92.46	93.37	98.84	99.71	96.03
843	93.63	94.06	99.48	99.76	96.69
844	93.60	96.19	97.02	99.58	96.60
845	94.96	96.37	98.39	99.60	97.37
846	95.82	98.19	97.47	99.75	97.83
847	96.29	97.19	99.01	99.80	98.09
848	95.60	96.95	98.49	99.68	97.71
849	95.88	97.85	97.85	99.83	97.85
850	95.18	97.11	97.85	99.78	97.48
851	93.86	96.96	96.56	99.12	96.76
852	93.50	97.73	95.45	99.01	96.58
853	92.14	97.19	94.44	99.17	95.80
854	93.56	97.56	95.65	99.37	96.59
855	94.91	98.37	96.35	99.46	97.35
856	95.87	98.85	96.91	99.42	97.87
857	95.72	98.87	96.71	99.87	97.78
858	96.28	98.02	98.14	99.38	98.08
859	91.32	96.06	94.59	98.65	95.32
860	93.30	95.93	96.97	99.11	96.45
861	95.01	97.06	97.73	99.33	97.39
862	96.44	98.28	98.06	99.52	98.17
863	92.60	98.39	93.93	99.17	96.11
864	95.76	98.47	97.14	99.66	97.80
865	93.51	96.43	96.64	99.78	96.54
866	95.33	98.20	96.93	99.81	97.56
867	96.35	98.10	98.13	99.48	98.11

868	96.69	98.83	97.78	99.57	98.30
869	94.40	97.23	96.90	99.03	97.06
870	95.29	97.77	97.32	99.47	97.54
871	94.76	97.87	96.62	99.77	97.24
872	92.08	95.85	95.59	99.51	95.72
873	90.37	95.35	94.10	99.25	94.72
874	92.14	95.17	96.35	99.66	95.76
875	88.71	89.28	99.20	99.56	93.98
876	92.28	95.81	95.85	99.77	95.83
877	95.72	96.05	99.62	99.87	97.80
878	95.26	97.48	97.56	99.66	97.52
879	95.42	98.47	96.77	99.88	97.61
880	96.54	98.29	98.14	99.47	98.22
881	94.72	96.69	97.81	99.06	97.25
882	90.71	94.41	95.45	99.19	94.93
883	87.97	88.90	98.63	99.49	93.51
884	92.26	95.96	95.68	99.67	95.82
885	92.61	93.42	99.01	99.49	96.13
886	96.28	98.08	98.07	99.62	98.07
887	95.43	96.88	98.39	99.32	97.63
888	93.62	96.59	96.66	99.07	96.62
889	92.18	97.49	94.25	98.97	95.84
890	94.13	95.46	98.42	99.67	96.92
891	93.97	97.21	96.42	99.47	96.81
892	96.83	98.67	98.07	99.86	98.37
893	95.58	97.58	97.82	99.55	97.70
894	93.97	97.28	96.38	98.98	96.83
895	93.95	96.53	97.10	98.98	96.81
896	92.33	93.73	98.39	98.54	96.00
897	91.86	95.23	96.07	98.39	95.65
898	94.74	96.76	97.74	99.33	97.25
899	93.25	96.83	95.99	99.30	96.41
900	94.49	96.64	97.58	99.33	97.11
901	92.74	94.06	98.33	99.79	96.15
902	95.09	98.24	96.65	99.50	97.44
903	95.78	99.10	96.58	99.65	97.82
904	94.47	98.33	95.91	99.54	97.11
905	96.09	97.33	98.63	99.72	97.98
906	96.12	98.35	97.64	99.63	97.99
907	95.22	96.31	98.76	99.67	97.52
908	94.09	95.40	98.45	99.61	96.90
909	93.45	95.50	97.61	99.28	96.54
910	91.32	93.40	97.33	99.46	95.32
911	98.08	99.09	98.96	99.87	99.02
912	96.50	99.32	97.12	99.73	98.21
913	97.59	98.28	99.27	99.84	98.77
914	96.45	99.65	96.77	99.76	98.19
915	96.99	99.34	97.61	99.55	98.47
916	87.57	89.95	96.96	97.51	93.32
917	91.71	95.87	95.19	98.96	95.53
918	86.57	96.21	89.04	99.28	92.48
919	93.83	98.23	95.32	99.51	96.75
920	95.63	98.85	96.67	99.41	97.75
921	93.97	96.33	97.30	99.41	96.82
922	93.78	95.89	97.54	99.59	96.71
923	95.23	99.23	95.91	99.54	97.54
924	93.79	95.82	97.63	99.46	96.72
925	96.15	98.16	97.85	99.84	98.00
926	95.42	96.92	98.31	99.72	97.61
927	94.35	98.77	95.42	99.36	97.07
928	95.48	97.75	97.54	99.45	97.65
929	94.10	97.57	96.23	99.28	96.90

930	92.75	97.49	94.87	98.96	96.16
931	92.39	97.04	94.91	98.62	95.96
932	94.76	96.91	97.62	99.01	97.26
933	92.72	95.00	97.29	99.06	96.13
934	94.07	97.48	96.25	99.68	96.86
935	95.40	98.15	97.07	99.47	97.61
936	93.41	95.68	97.33	99.58	96.50
937	94.91	96.44	98.28	99.16	97.36
938	92.97	98.19	94.55	98.56	96.34
939	94.76	97.43	97.09	99.22	97.26
940	96.39	99.00	97.30	99.60	98.14
941	96.62	97.61	98.92	99.71	98.26
942	96.08	97.45	98.50	99.64	97.97
943	94.30	97.33	96.67	99.38	97.00
944	95.07	95.80	99.16	99.71	97.45
945	89.63	91.49	97.39	99.64	94.35
946	95.36	96.76	98.41	99.79	97.58
947	89.38	91.89	96.52	99.70	94.15
948	89.55	90.23	99.13	99.32	94.47
949	92.84	97.76	94.67	99.59	96.19
950	98.57	99.01	99.55	99.90	99.28
951	97.26	98.41	98.78	99.81	98.59
952	95.16	97.11	97.82	99.82	97.46
953	95.42	96.98	98.26	99.69	97.61
954	92.72	96.81	95.42	99.22	96.11
955	91.31	96.92	93.77	99.17	95.32
956	88.15	95.21	91.57	99.54	93.36
957	93.45	98.26	94.88	99.66	96.54
958	96.80	98.02	98.68	99.76	98.35
959	94.03	96.46	97.26	99.14	96.86
960	93.21	95.06	97.79	99.41	96.41
961	94.70	95.73	98.79	99.71	97.24
962	94.66	98.55	95.95	99.13	97.23
963	92.70	96.89	95.35	98.93	96.11
964	89.57	92.83	95.66	99.65	94.23
965	93.04	97.83	94.82	99.73	96.30
966	94.78	97.22	97.31	99.32	97.27
967	95.56	98.33	97.06	99.43	97.70
968	96.34	97.80	98.41	99.80	98.11
969	94.84	98.98	95.73	99.56	97.33
970	95.88	97.42	98.30	99.64	97.86
971	95.97	98.32	97.52	99.38	97.92
972	94.22	98.67	95.39	99.13	97.00
973	94.38	97.08	96.99	99.63	97.03
974	96.48	99.03	97.37	99.69	98.19
975	96.41	98.54	97.77	99.55	98.15
976	96.11	98.26	97.72	99.45	97.99
977	95.77	98.23	97.38	99.60	97.80
978	84.17	86.88	95.37	99.27	90.93
979	78.25	79.04	98.96	98.70	87.89
980	88.73	90.71	97.21	99.22	93.85
981	90.40	90.82	99.43	99.72	94.93
982	97.51	99.46	98.01	99.87	98.73
983	94.83	96.68	97.90	99.59	97.29
984	91.87	92.38	99.35	99.69	95.74
985	87.11	88.32	98.10	99.54	92.95
986	92.14	94.44	97.17	99.39	95.79
987	92.84	97.37	95.06	99.03	96.20
988	89.35	92.34	96.08	98.76	94.17
989	93.23	97.29	95.51	99.64	96.39
990	94.75	96.54	97.95	99.79	97.24
991	95.66	97.42	98.06	99.55	97.74

992	91.95	96.05	95.28	99.13	95.66
993	81.22	82.06	98.61	99.08	89.58
994	93.59	95.08	98.20	99.67	96.62
995	97.45	98.56	98.82	99.93	98.69
996	95.62	96.43	99.07	99.81	97.74
997	95.10	97.05	97.84	99.44	97.44
998	89.79	93.64	95.11	99.18	94.37
999	93.91	96.03	97.53	99.75	96.78
1000	95.16	98.41	96.56	99.57	97.48
1001	95.44	97.14	98.11	99.69	97.62
1002	94.58	97.94	96.39	99.36	97.16
1003	94.18	96.92	96.93	99.37	96.93
1004	93.22	95.62	97.16	99.67	96.38
1005	95.72	97.13	98.43	99.78	97.78
1006	95.92	99.03	96.80	99.59	97.90
1007	94.09	98.91	95.01	99.54	96.92
1008	94.53	99.46	94.98	99.87	97.17
1009	83.75	86.53	95.32	98.89	90.71
1010	90.85	95.05	94.96	99.17	95.01
1011	92.80	94.39	98.03	99.66	96.17
1012	97.09	97.81	99.21	99.84	98.51
1013	92.61	93.35	99.05	99.79	96.12
1014	92.31	92.95	99.16	99.78	95.96
1015	97.11	98.55	98.48	99.82	98.52
1016	95.52	98.21	97.13	99.62	97.67
1017	94.82	95.92	98.73	99.57	97.30
1018	93.35	95.08	97.93	99.41	96.49
1019	90.22	92.83	96.58	99.22	94.67
1020	92.10	96.44	95.07	99.28	95.75
1021	96.19	97.15	98.94	99.71	98.03
1022	96.66	98.40	98.16	99.69	98.28
1023	97.34	99.04	98.24	99.87	98.64
1024	93.39	98.36	94.80	99.06	96.55
1025	90.64	92.59	97.49	98.99	94.98
1026	95.37	96.77	98.41	99.77	97.58
1027	96.42	97.26	99.07	99.90	98.16
1028	95.83	98.40	97.27	99.75	97.84
1029	92.74	97.39	94.91	99.38	96.13
1030	93.65	94.73	98.70	99.69	96.67
1031	83.87	95.26	86.66	99.06	90.76
1032	93.16	98.55	94.40	99.11	96.43
1033	96.24	98.53	97.59	99.66	98.06
1034	96.28	98.93	97.24	99.76	98.08
1035	95.45	97.52	97.73	99.62	97.63
1036	92.73	98.09	94.31	99.16	96.17
1037	94.94	96.88	97.82	99.63	97.35
1038	92.87	96.78	95.60	99.46	96.19
1039	93.45	94.69	98.53	99.42	96.57
1040	86.85	90.02	95.35	99.04	92.61
1041	91.40	93.71	97.07	99.40	95.36
1042	95.11	96.35	98.58	99.70	97.45
1043	94.72	96.04	98.49	99.44	97.25
1044	89.35	90.34	98.65	99.38	94.31
1045	96.34	98.73	97.50	99.58	98.11
1046	96.17	97.85	98.19	99.57	98.02
1047	94.76	98.53	96.02	99.80	97.26
1048	94.81	96.99	97.55	99.80	97.27
1049	96.05	96.67	99.29	99.86	97.96
1050	95.76	96.77	98.86	99.80	97.80
1051	92.65	93.31	99.15	99.80	96.14
1052	94.56	96.37	97.94	99.52	97.15
1053	95.39	97.72	97.47	99.47	97.60

1054	92.61	95.52	96.57	99.37	96.04
1055	89.70	92.19	96.62	99.48	94.35
1056	89.24	94.27	93.74	99.53	94.00
1057	96.48	98.83	97.56	99.44	98.19
1058	96.48	98.58	97.79	99.65	98.18
1059	95.47	99.08	96.32	99.20	97.68
1060	95.16	96.97	98.02	98.90	97.49
1061	97.51	99.15	98.32	99.44	98.73
1062	97.93	99.50	98.41	99.58	98.95
1063	99.04	99.66	99.38	99.82	99.52
1064	99.16	99.67	99.48	99.86	99.57
1065	97.98	99.23	98.72	99.69	98.98
1066	97.48	99.58	97.89	99.48	98.73
1067	97.85	99.44	98.39	99.54	98.91
1068	94.15	97.62	96.21	99.75	96.91
1069	95.58	98.30	97.14	99.06	97.72
1070	95.08	97.81	97.07	98.98	97.44
1071	94.75	98.33	96.24	99.11	97.27
1072	94.57	98.19	96.17	99.08	97.17
1073	94.34	96.79	97.27	99.12	97.03
1074	94.82	97.28	97.30	99.16	97.29
1075	96.23	97.89	98.22	99.04	98.06
1076	96.94	99.12	97.76	99.33	98.44
1077	98.21	98.74	99.45	99.83	99.09
1078	97.16	98.69	98.40	99.66	98.54
1079	93.78	94.10	99.64	99.71	96.79
1080	94.80	98.53	96.11	99.03	97.31
1081	93.78	97.47	96.03	98.53	96.74
1082	93.83	97.61	95.93	98.78	96.76
1083	93.48	97.48	95.69	98.66	96.58
1084	93.84	96.56	96.98	98.66	96.77
1085	87.23	89.08	97.18	99.36	92.95
1086	90.32	96.00	93.40	99.67	94.68
1087	96.02	98.68	97.21	99.86	97.94
1088	96.79	99.24	97.49	99.75	98.36
1089	96.68	98.01	98.57	99.75	98.29
1090	94.26	97.05	96.89	99.41	96.97
1091	93.30	94.86	98.10	99.75	96.45
1092	95.59	97.12	98.29	99.82	97.70
1093	94.08	97.91	95.87	99.48	96.88
1094	94.27	95.77	98.26	99.49	97.00
1095	94.97	96.46	98.30	99.67	97.37
1096	95.48	96.69	98.63	99.69	97.65
1097	94.97	95.50	99.38	99.76	97.40
1098	95.42	97.81	97.40	99.77	97.60
1099	95.35	97.47	97.69	99.38	97.58
1100	95.91	98.06	97.71	99.22	97.88
1101	93.17	96.36	96.37	99.14	96.37
1102	95.96	98.72	97.14	99.29	97.92
1103	97.15	99.37	97.74	99.62	98.55
1104	96.95	98.82	98.06	99.60	98.43
1105	95.21	97.77	97.26	98.92	97.51
1106	96.62	98.63	97.91	99.10	98.27
1107	97.46	98.94	98.47	99.35	98.71
1108	98.23	99.21	99.01	99.61	99.11
1109	99.15	99.54	99.60	99.82	99.57
1110	97.73	99.27	98.42	99.52	98.85
1111	97.42	98.91	98.46	99.53	98.68
1112	88.62	92.80	94.46	99.46	93.62
1113	97.44	99.35	98.06	99.48	98.70
1114	97.80	99.12	98.64	99.56	98.88
1115	95.45	98.67	96.66	99.05	97.66

1116	93.74	97.50	95.95	98.66	96.72
1117	93.63	97.17	96.12	98.86	96.64
1118	95.03	97.91	96.92	99.17	97.41
1119	96.65	99.08	97.50	99.56	98.28
1120	98.17	99.31	98.83	99.78	99.07
1121	96.22	99.14	97.03	99.23	98.07
1122	97.12	99.19	97.88	99.43	98.53
1123	94.00	96.48	97.14	99.84	96.81
1124	97.75	98.60	99.11	99.79	98.85
1125	95.43	98.25	97.01	99.44	97.62
1126	94.19	97.13	96.79	98.75	96.96
1127	92.04	96.70	94.91	97.72	95.79
1128	94.60	97.86	96.54	98.66	97.19
1129	93.43	98.52	94.73	98.84	96.59
1130	91.68	97.66	93.59	98.81	95.58
1131	81.44	83.46	96.10	99.05	89.34
1132	94.19	96.84	97.00	99.84	96.92
1133	94.42	95.81	98.38	99.59	97.08
1134	95.57	98.51	96.90	99.73	97.70
1135	94.82	98.44	96.20	99.34	97.30
1136	93.77	98.29	95.23	99.15	96.74
1137	93.99	96.94	96.70	99.52	96.82
1138	95.37	96.59	98.62	99.71	97.59
1139	95.20	97.70	97.28	99.67	97.49
1140	95.74	96.73	98.87	99.85	97.79
1141	94.07	97.65	96.11	99.42	96.87
1142	96.62	98.71	97.83	99.46	98.27
1143	96.89	99.19	97.65	99.43	98.41
1144	97.35	98.63	98.66	99.64	98.64
1145	95.54	96.90	98.47	99.76	97.68
1146	96.72	97.99	98.63	99.69	98.31
1147	94.92	97.07	97.61	99.34	97.34
1148	93.78	96.51	96.90	99.26	96.71
1149	92.61	95.95	96.11	99.44	96.03
1150	93.71	97.58	95.76	99.77	96.66
1151	97.29	98.03	99.20	99.84	98.61
1152	95.46	97.05	98.24	99.62	97.64
1153	95.55	96.71	98.69	99.74	97.69
1154	96.39	99.08	97.22	99.75	98.14
1155	95.07	98.55	96.34	99.71	97.43
1156	93.46	94.27	99.01	99.74	96.58
1157	95.65	97.91	97.56	99.53	97.74
1158	94.91	97.19	97.46	99.81	97.32
1159	95.10	95.63	99.38	99.84	97.47
1160	93.79	97.94	95.52	99.74	96.71
1161	96.30	98.34	97.84	99.67	98.09
1162	96.55	98.52	97.92	99.80	98.22
1163	94.44	99.00	95.32	99.37	97.12
1164	93.87	97.74	95.85	98.82	96.79
1165	94.89	98.43	96.28	99.19	97.34
1166	96.02	98.73	97.18	99.38	97.95
1167	96.19	97.36	98.72	99.57	98.03
1168	94.81	96.46	98.13	99.64	97.28
1169	84.07	85.69	97.23	99.10	91.10
1170	83.82	85.80	96.53	99.23	90.85
1171	88.37	97.39	90.13	99.63	93.62
1172	92.84	93.61	99.04	99.61	96.25
1173	92.79	96.45	95.85	99.19	96.15
1174	93.80	96.26	97.19	99.29	96.72
1175	93.86	94.99	98.62	99.82	96.77
1176	92.81	93.38	99.30	99.69	96.25
1177	89.90	91.83	97.37	99.38	94.52

1178	94.19	95.74	98.19	99.55	96.95
1179	94.41	96.37	97.75	99.70	97.05
1180	95.04	96.24	98.61	99.68	97.41
1181	90.24	91.69	97.99	99.64	94.74
1182	88.46	89.96	97.90	99.14	93.76
1183	91.09	93.75	96.62	99.45	95.16
1184	92.62	93.78	98.52	99.78	96.09
1185	95.19	95.69	99.42	99.79	97.52
1186	93.47	93.73	99.70	99.76	96.62
1187	94.07	94.74	99.18	99.82	96.91
1188	97.15	98.50	98.57	99.87	98.54
1189	96.39	96.79	99.54	99.89	98.15
1190	94.85	98.47	96.18	99.60	97.31
1191	95.67	97.15	98.35	99.83	97.75
1192	96.17	97.94	98.08	99.76	98.01
1193	94.78	96.31	98.25	99.49	97.27
1194	91.30	94.74	95.83	99.23	95.28
1195	93.31	95.96	96.92	99.38	96.44
1196	93.73	96.37	97.03	98.77	96.70
1197	95.23	98.06	96.99	99.12	97.52
1198	92.24	98.89	93.10	99.74	95.91
1199	97.08	97.52	99.52	99.83	98.51
1200	95.46	97.63	97.65	99.42	97.64
1201	94.09	97.10	96.67	99.13	96.89
1202	93.84	96.75	96.73	99.46	96.74
1203	94.14	98.62	95.29	99.74	96.93
1204	90.31	95.55	93.81	99.49	94.67
1205	90.88	92.62	97.70	99.52	95.09
1206	89.92	92.16	96.96	99.47	94.50
1207	93.98	95.00	98.77	99.73	96.85
1208	92.85	94.45	98.06	99.41	96.22
1209	92.28	93.95	97.94	99.31	95.90
1210	94.79	96.23	98.36	99.45	97.28
1211	95.71	98.05	97.48	99.72	97.77
1212	95.49	97.53	97.77	99.50	97.65
1213	91.96	96.70	94.63	99.79	95.65
1214	91.34	93.94	96.73	99.39	95.31
1215	87.40	90.81	95.23	98.70	92.97
1216	93.20	93.88	99.17	99.68	96.45
1217	96.47	97.64	98.71	99.90	98.18
1218	94.31	96.19	97.83	99.64	97.00
1219	88.86	91.75	96.02	99.31	93.84
1220	92.43	96.66	95.21	99.67	95.93
1221	93.67	97.45	95.85	99.55	96.64
1222	93.02	96.62	95.94	99.31	96.28
1223	96.34	99.17	97.08	99.86	98.12
1224	92.45	95.35	96.60	98.86	95.97
1225	90.89	94.46	95.73	98.13	95.09
1226	94.81	98.67	96.00	99.15	97.32
1227	96.53	97.35	99.10	99.77	98.21
1228	95.85	98.23	97.47	99.71	97.84
1229	94.48	96.75	97.45	99.47	97.10
1230	94.08	95.35	98.47	99.84	96.89
1231	94.54	96.90	97.35	99.55	97.12
1232	94.71	96.45	98.01	99.59	97.22
1233	93.04	94.34	98.39	99.68	96.32
1234	94.88	96.69	97.96	99.65	97.32
1235	92.61	93.29	99.15	99.65	96.13
1236	93.33	96.08	96.81	99.57	96.44
1237	92.63	95.60	96.50	99.49	96.05
1238	95.08	97.59	97.27	99.48	97.43
1239	93.69	95.60	97.78	99.25	96.68

1240	90.06	91.57	97.90	99.62	94.63
1241	85.74	88.44	95.71	99.33	91.93
1242	86.35	88.90	96.14	98.92	92.38
1243	88.48	90.06	97.66	99.61	93.71
1244	86.73	88.24	97.61	99.46	92.69
1245	91.48	93.34	97.60	99.58	95.42
1246	89.28	90.65	98.13	99.23	94.24
1247	88.50	94.63	92.56	99.10	93.58
1248	81.87	87.41	90.83	99.11	89.08
1249	89.75	91.23	97.97	99.35	94.48
1250	93.60	95.77	97.48	99.32	96.62
1251	94.58	97.35	96.97	99.26	97.16
1252	90.35	94.86	94.64	98.43	94.75
1253	95.62	97.28	98.18	99.15	97.73
1254	94.63	98.27	96.17	99.08	97.21
1255	95.75	97.79	97.80	99.46	97.79
1256	85.51	91.02	92.12	99.60	91.56
1257	96.38	98.45	97.82	99.57	98.13
1258	95.63	98.74	96.76	99.46	97.74
1259	95.65	97.85	97.63	99.40	97.74
1260	94.66	95.26	99.30	99.61	97.24
1261	94.68	96.16	98.29	99.55	97.22
1262	91.81	92.76	98.75	99.73	95.66
1263	94.66	96.89	97.49	99.82	97.19
1264	96.26	97.14	99.02	99.91	98.07
1265	94.59	95.87	98.50	99.83	97.17
1266	91.96	93.32	98.27	99.49	95.73
1267	89.08	91.11	97.10	99.67	94.01
1268	97.40	98.94	98.40	99.80	98.67
1269	93.23	96.14	96.63	99.59	96.39
1270	93.64	97.11	96.15	99.39	96.63
1271	94.07	96.01	97.76	99.53	96.87
1272	91.47	94.47	96.27	99.62	95.37
1273	92.77	95.40	96.87	99.49	96.13
1274	96.91	98.89	97.93	99.89	98.41
1275	94.45	95.06	99.28	99.74	97.12
1276	95.90	98.02	97.72	99.65	97.87
1277	94.65	96.76	97.63	99.34	97.19
1278	86.13	92.87	91.23	99.02	92.04
1279	92.77	93.05	99.68	99.75	96.25
1280	85.86	88.29	96.08	99.47	92.02
1281	88.03	89.57	97.73	99.34	93.47
1282	93.04	95.49	97.12	99.24	96.30
1283	92.70	96.68	95.54	98.98	96.11
1284	93.66	96.37	96.93	99.09	96.65
1285	91.67	97.16	93.97	99.04	95.54
1286	91.75	97.21	94.09	98.41	95.62
1287	91.79	94.90	96.33	98.45	95.61
1288	94.98	96.78	97.99	99.43	97.38
1289	94.83	97.69	96.91	99.18	97.30
1290	95.10	96.32	98.60	99.68	97.45
1291	95.54	98.03	97.34	99.20	97.68
1292	95.27	97.49	97.59	99.18	97.54
1293	90.26	94.82	94.41	99.75	94.62
1294	90.10	93.16	96.00	99.62	94.56
1295	93.23	96.64	96.12	99.82	96.38
1296	93.99	95.06	98.72	99.76	96.85
1297	94.00	97.16	96.47	99.80	96.82
1298	95.04	96.00	98.89	99.71	97.42
1299	95.44	96.38	98.92	99.77	97.63
1300	92.75	93.54	99.01	99.64	96.20
1301	94.67	98.12	96.31	99.49	97.21

1302	93.48	95.19	97.97	99.37	96.56
1303	94.88	96.73	97.91	99.59	97.32
1304	96.39	97.18	99.13	99.76	98.14
1305	96.43	97.39	98.95	99.87	98.16
1306	95.33	96.87	98.26	99.84	97.56
1307	95.84	98.06	97.62	99.64	97.84
1308	95.49	97.35	97.94	99.74	97.64
1309	96.33	97.77	98.43	99.75	98.10
1310	94.23	96.58	97.36	99.21	96.97
1311	93.24	95.64	97.18	99.49	96.40
1312	95.61	97.43	98.00	99.57	97.71
1313	94.59	96.34	97.99	99.75	97.16
1314	92.97	94.89	97.70	99.22	96.28
1315	93.26	96.10	96.76	98.95	96.43
1316	93.46	96.68	96.41	98.81	96.54
1317	93.45	96.43	96.64	98.96	96.53
1318	93.62	98.50	94.88	99.41	96.66
1319	93.82	96.63	96.85	98.98	96.74
1320	93.20	97.79	95.11	98.72	96.43
1321	94.17	97.07	96.78	99.39	96.93
1322	94.86	98.36	96.30	99.38	97.32
1323	94.86	96.74	97.87	99.79	97.30
1324	90.73	95.89	94.09	98.54	94.98
1325	92.65	96.48	95.67	99.11	96.07
1326	90.74	91.32	99.24	99.62	95.11
1327	89.85	91.41	97.85	99.53	94.52
1328	94.10	95.83	97.98	99.66	96.89
1329	93.08	93.67	99.28	99.66	96.39
1330	95.28	97.44	97.62	99.89	97.53
1331	95.31	96.07	99.11	99.88	97.57
1332	91.74	93.39	97.87	99.69	95.58
1333	92.40	94.62	97.28	99.54	95.93
1334	94.50	96.49	97.73	99.57	97.11
1335	92.44	96.51	95.36	99.51	95.93
1336	95.81	96.47	99.26	99.71	97.85
1337	96.39	98.22	98.05	99.68	98.13
1338	97.14	98.74	98.32	99.90	98.53
1339	97.73	99.40	98.30	99.80	98.85
1340	96.35	98.88	97.38	99.57	98.12
1341	94.61	96.70	97.64	99.47	97.17
1342	93.80	95.85	97.64	99.14	96.74
1343	94.33	96.07	98.03	99.09	97.04
1344	94.66	98.94	95.61	99.17	97.25
1345	95.00	96.82	97.96	99.62	97.38
1346	94.89	97.41	97.25	99.22	97.33
1347	93.62	96.04	97.23	99.03	96.63
1348	93.81	96.23	97.26	98.97	96.74
1349	93.70	95.45	98.00	98.80	96.71
1350	93.60	96.27	96.97	99.04	96.62
1351	92.37	95.46	96.39	98.84	95.92
1352	90.56	92.27	97.92	98.36	95.01
1353	92.77	95.10	97.21	99.30	96.14
1354	91.84	96.92	94.29	99.80	95.59
1355	91.57	93.87	97.07	99.67	95.44
1356	95.81	96.80	98.87	99.89	97.83
1357	93.78	96.24	97.17	99.60	96.70
1358	92.79	94.67	97.70	99.58	96.16
1359	95.20	96.47	98.54	99.84	97.50
1360	94.88	97.44	97.16	99.84	97.30
1361	95.94	96.98	98.84	99.76	97.90
1362	95.47	98.05	97.23	99.70	97.64
1363	92.08	94.87	96.62	99.51	95.73

1364	94.43	96.51	97.62	99.83	97.06
1365	95.12	95.37	99.72	99.85	97.49
1366	95.39	99.20	96.10	99.54	97.62
1367	92.64	95.89	96.23	99.22	96.06
1368	96.64	98.25	98.28	99.75	98.26
1369	97.98	98.94	99.00	99.91	98.97
1370	97.24	98.24	98.94	99.69	98.59
1371	96.82	98.67	98.07	99.62	98.37
1372	93.86	95.76	97.80	99.31	96.77
1373	93.52	94.94	98.36	98.97	96.62
1374	94.82	97.47	97.11	99.25	97.29
1375	95.13	98.22	96.71	99.64	97.46
1376	93.67	95.78	97.54	99.43	96.65
1377	94.37	98.13	96.00	99.36	97.05
1378	93.84	98.66	95.00	99.11	96.80
1379	96.70	98.02	98.57	99.88	98.30
1380	90.31	92.51	97.20	98.68	94.80
1381	94.50	98.06	96.20	99.44	97.12
1382	94.47	97.07	97.11	99.48	97.09
1383	93.95	98.80	94.99	99.22	96.86
1384	93.10	96.67	95.99	99.19	96.32
1385	93.44	94.08	99.21	99.77	96.58
1386	95.23	98.63	96.43	99.77	97.52
1387	96.08	99.02	96.95	99.80	97.98
1388	95.94	96.84	98.99	99.70	97.90
1389	95.67	97.74	97.76	99.69	97.75
1390	95.79	98.73	96.93	99.58	97.82
1391	89.91	94.47	94.35	99.64	94.41
1392	94.36	94.71	99.59	99.73	97.09
1393	96.49	98.00	98.37	99.82	98.18
1394	94.76	96.52	97.98	99.78	97.25
1395	92.46	92.71	99.71	99.78	96.08
1396	91.19	93.99	96.44	99.75	95.20
1397	97.96	98.35	99.58	99.95	98.96
1398	94.03	97.69	96.01	99.66	96.85
1399	95.43	98.03	97.22	99.57	97.62
1400	94.51	99.09	95.28	99.66	97.15
1401	96.96	98.60	98.27	99.88	98.44
1402	95.21	96.32	98.72	99.82	97.50
1403	96.08	98.61	97.34	99.52	97.97
1404	95.61	98.07	97.36	99.51	97.72
1405	94.37	98.25	95.88	99.51	97.05
1406	90.67	95.51	94.34	98.83	94.92
1407	96.34	98.21	98.00	99.78	98.11
1408	94.47	99.02	95.29	99.85	97.12
1409	96.28	98.14	98.01	99.72	98.07
1410	95.06	96.00	98.92	99.66	97.44
1411	95.55	96.90	98.48	99.87	97.68
1412	95.25	96.66	98.40	99.72	97.52
1413	93.98	98.37	95.38	99.41	96.85
1414	91.55	96.80	94.16	98.90	95.46
1415	95.41	98.29	96.93	99.84	97.60
1416	96.19	98.20	97.85	99.84	98.02
1417	95.58	96.60	98.84	99.87	97.71
1418	94.64	96.99	97.36	99.83	97.17
1419	95.82	97.64	98.01	99.85	97.82
1420	96.20	97.42	98.66	99.61	98.04
1421	96.29	98.67	97.51	99.51	98.09
1422	94.77	96.19	98.38	99.47	97.27
1423	92.09	93.10	98.70	99.68	95.81
1424	94.29	95.47	98.61	99.66	97.02
1425	92.38	97.50	94.39	99.63	95.92

1426	94.40	95.61	98.58	99.80	97.07
1427	92.78	93.96	98.51	99.79	96.18
1428	90.46	90.81	99.56	99.71	94.98
1429	96.53	98.49	97.93	99.86	98.21
1430	95.69	97.94	97.57	99.72	97.75
1431	93.72	94.48	99.09	99.67	96.73
1432	97.77	99.64	98.11	99.88	98.87
1433	97.70	98.59	99.06	99.78	98.82
1434	85.84	88.34	96.04	99.22	92.03
1435	96.35	98.50	97.73	99.74	98.11
1436	95.29	97.14	97.94	99.78	97.54
1437	93.30	97.60	95.30	99.69	96.44
1438	95.59	97.69	97.71	99.67	97.70
1439	95.10	96.06	98.90	99.68	97.46
1440	92.96	93.72	99.07	99.51	96.32
1441	90.78	91.39	99.18	99.67	95.13
1442	89.41	90.83	98.00	99.52	94.28
1443	92.94	95.21	97.28	99.46	96.24
1444	89.70	93.79	94.83	99.30	94.30
1445	97.16	97.92	99.19	99.73	98.55
1446	96.74	98.05	98.60	99.71	98.32
1447	96.71	98.51	98.10	99.76	98.31
1448	95.63	97.73	97.71	99.71	97.72
1449	96.62	97.71	98.81	99.84	98.26
1450	97.01	98.16	98.77	99.86	98.46
1451	95.77	98.39	97.23	99.55	97.81
1452	97.25	98.05	99.14	99.82	98.59
1453	96.27	97.60	98.55	99.64	98.07
1454	93.56	96.29	96.85	99.77	96.57
1455	91.15	91.71	99.29	99.60	95.35
1456	92.83	93.65	98.98	99.66	96.24
1457	94.90	96.00	98.73	99.54	97.35
1458	94.77	96.40	98.12	99.73	97.26
1459	94.52	96.20	98.07	99.43	97.13
1460	91.40	92.32	98.81	99.56	95.45
1461	91.48	92.38	98.81	99.65	95.49
1462	95.88	97.58	98.14	99.62	97.86
1463	92.81	98.21	94.27	99.36	96.20
1464	94.12	97.45	96.37	99.10	96.91
1465	94.76	96.71	97.80	99.37	97.25
1466	95.85	97.50	98.19	99.71	97.84
1467	97.95	99.27	98.64	99.91	98.96
1468	96.94	99.15	97.72	99.88	98.43
1469	96.97	99.09	97.82	99.54	98.45
1470	96.66	98.56	98.01	99.30	98.29
1471	95.63	98.96	96.57	99.28	97.75
1472	94.69	99.09	95.47	99.63	97.25
1473	96.23	97.52	98.59	99.73	98.05
1474	89.06	90.69	97.68	99.47	94.05
1475	85.26	86.39	98.20	99.20	91.92
1476	94.15	94.28	99.86	99.85	96.99
1477	94.83	95.83	98.82	99.78	97.31
1478	94.72	96.57	97.88	99.86	97.22
1479	95.35	97.05	98.11	99.56	97.57
1480	95.51	98.08	97.26	99.29	97.67
1481	97.38	98.89	98.43	99.65	98.66
1482	95.91	98.86	96.95	99.49	97.89
1483	95.06	97.66	97.18	99.39	97.42
1484	95.52	98.62	96.76	99.48	97.68
1485	95.87	98.86	96.90	99.40	97.87
1486	97.06	98.46	98.52	99.46	98.49
1487	94.82	95.47	99.23	99.78	97.32

1488	95.56	97.33	98.04	99.81	97.68
1489	86.00	89.12	95.11	99.59	92.02
1490	86.16	88.19	96.69	99.58	92.24
1491	92.22	92.64	99.48	99.65	95.94
1492	98.16	99.54	98.60	99.85	99.07
1493	97.11	99.12	97.93	99.57	98.52
1494	94.62	98.52	95.90	99.55	97.19
1495	97.09	98.46	98.56	99.79	98.51
1496	95.28	98.29	96.80	99.69	97.54
1497	96.42	98.83	97.50	99.61	98.16
1498	97.28	99.05	98.17	99.69	98.61
1499	97.34	98.48	98.79	99.82	98.64
1500	94.39	97.21	96.85	99.84	97.03
1501	96.18	98.75	97.32	99.63	98.03
1502	96.87	99.06	97.73	99.77	98.39
1503	92.08	93.97	97.60	99.74	95.75
1504	93.72	95.09	98.33	99.80	96.69
1505	95.60	98.36	97.06	99.89	97.71
1506	97.32	97.90	99.37	99.75	98.63
1507	97.43	99.43	97.97	99.57	98.69
1508	97.59	99.30	98.26	99.60	98.77
1509	96.26	96.99	99.18	99.86	98.07
1510	97.52	98.28	99.19	99.83	98.73
1511	96.96	98.36	98.51	99.71	98.44
1512	96.77	99.08	97.61	99.70	98.34
1513	96.52	98.38	98.03	99.71	98.20
1514	93.08	93.27	99.78	99.79	96.41
1515	95.69	96.03	99.61	99.82	97.79
1516	97.58	98.52	99.01	99.82	98.76
1517	98.02	99.12	98.86	99.95	98.99
1518	97.74	99.74	97.98	99.92	98.85
1519	93.47	94.37	98.90	99.73	96.58
1520	93.36	94.89	98.13	99.74	96.48
1521	93.81	98.89	94.72	99.68	96.76
1522	96.89	99.03	97.80	99.60	98.41
1523	97.82	99.07	98.71	99.82	98.89
1524	96.53	98.62	97.80	99.82	98.21
1525	97.32	98.06	99.21	99.71	98.63
1526	97.67	98.43	99.19	99.85	98.81
1527	95.73	97.55	98.00	99.78	97.78
1528	96.95	99.00	97.87	99.82	98.43
1529	96.20	98.22	97.84	99.77	98.03
1530	94.34	95.16	99.01	99.85	97.05
1531	91.50	93.14	97.84	99.74	95.44
1532	92.61	93.97	98.27	99.79	96.07
1533	92.53	98.04	94.08	99.78	96.02
1534	96.08	97.82	98.12	99.75	97.97
1535	94.38	96.00	98.13	99.54	97.05
1536	96.04	97.03	98.90	99.65	97.96
1537	96.26	98.35	97.78	99.84	98.06
1538	91.68	92.26	99.24	99.72	95.63
1539	96.84	98.62	98.12	99.82	98.37
1540	94.38	95.58	98.59	99.59	97.06
1541	96.44	97.36	98.99	99.80	98.17
1542	93.46	94.13	99.17	99.75	96.59
1543	92.56	93.92	98.28	99.71	96.05
1544	96.73	97.97	98.66	99.84	98.32
1545	96.87	98.88	97.91	99.67	98.39
1546	96.00	98.24	97.61	99.63	97.92
1547	96.14	98.11	97.89	99.74	98.00
1548	96.46	97.40	98.97	99.77	98.18
1549	92.27	93.83	98.00	99.76	95.87

1550	96.42	96.66	99.73	99.89	98.17
1551	92.40	93.16	99.02	99.74	96.00
1552	93.85	95.12	98.47	99.70	96.77
1553	96.37	97.23	99.04	99.81	98.13
1554	93.03	93.74	99.13	99.62	96.36
1555	93.58	94.90	98.39	99.75	96.62
1556	93.83	95.91	97.57	99.72	96.73
1557	94.97	95.90	98.92	99.77	97.39
1558	95.90	96.27	99.59	99.84	97.90
1559	90.43	97.49	92.29	99.56	94.82
1560	95.98	97.90	97.93	99.67	97.91
1561	96.37	98.72	97.54	99.72	98.12
1562	94.95	98.97	95.85	99.58	97.39
1563	94.96	95.62	99.23	99.75	97.39
1564	97.42	98.41	98.94	99.90	98.68
1565	95.72	99.07	96.55	99.56	97.79
1566	94.06	96.24	97.50	99.32	96.87
1567	96.51	98.63	97.77	99.69	98.20
1568	96.58	98.52	97.96	99.68	98.24
1569	93.45	95.76	97.28	99.77	96.51
1570	92.71	93.41	99.14	99.64	96.19
1571	92.56	92.92	99.57	99.62	96.13
1572	97.46	98.68	98.72	99.82	98.70
1573	92.62	93.28	99.18	99.66	96.14
1574	92.83	95.32	97.02	99.65	96.16
1575	91.61	93.73	97.29	99.56	95.48
1576	90.88	91.33	99.45	99.54	95.22
1577	93.48	94.33	98.96	99.70	96.59
1578	97.30	98.76	98.46	99.77	98.61
1579	97.39	99.10	98.24	99.62	98.67
1580	97.76	98.92	98.79	99.77	98.86
1581	95.20	96.62	98.41	99.51	97.50
1582	93.62	97.59	95.68	99.39	96.62
1583	94.65	95.65	98.83	99.68	97.21
1584	97.27	98.53	98.67	99.88	98.60
1585	92.97	93.89	98.86	99.66	96.31
1586	89.88	91.23	98.13	99.47	94.56
1587	90.98	92.38	98.16	99.51	95.18
1588	93.05	97.46	95.16	99.65	96.30
1589	94.64	95.76	98.69	99.72	97.20
1590	97.05	98.20	98.76	99.92	98.48
1591	96.90	97.86	98.96	99.81	98.41
1592	95.62	96.85	98.64	99.41	97.74
1593	95.19	96.37	98.67	99.35	97.51
1594	93.52	96.99	96.11	99.72	96.55
1595	96.01	98.07	97.79	99.43	97.93
1596	96.77	98.52	98.15	99.74	98.34
1597	95.44	99.35	96.03	99.42	97.66
1598	95.65	98.51	96.98	99.65	97.74
1599	91.98	92.96	98.73	99.69	95.76
1600	95.22	96.78	98.24	99.72	97.50
1601	96.74	99.33	97.36	99.64	98.33
1602	97.01	99.39	97.58	99.62	98.48
1603	96.63	98.18	98.35	99.66	98.26
1604	90.24	91.59	98.25	99.05	94.80
1605	95.18	98.87	96.18	99.42	97.51
1606	98.30	98.76	99.51	99.95	99.14
1607	95.28	97.74	97.31	99.84	97.53
1608	97.02	98.23	98.71	99.72	98.47
1609	95.95	97.54	98.27	99.33	97.90
1610	95.48	97.41	97.89	99.32	97.65
1611	92.99	94.18	98.57	99.32	96.32

1612	88.77	90.89	97.02	99.27	93.85
1613	92.82	96.73	95.58	99.68	96.15
1614	96.64	98.24	98.29	99.78	98.27
1615	95.42	99.09	96.22	99.67	97.63
1616	95.26	96.33	98.78	99.76	97.54
1617	98.49	99.01	99.46	99.96	99.24
1618	95.40	96.58	98.66	99.71	97.61
1619	94.75	95.78	98.80	99.81	97.26
1620	93.11	95.01	97.68	99.78	96.33
1621	95.96	98.26	97.55	99.58	97.90
1622	96.65	98.47	98.08	99.38	98.28
1623	95.92	97.10	98.71	99.21	97.90
1624	93.00	98.82	93.99	99.28	96.34
1625	96.94	99.38	97.51	99.72	98.44
1626	95.54	99.04	96.40	99.45	97.70
1627	94.15	98.99	95.02	99.38	96.97
1628	96.66	98.48	98.06	99.91	98.27
1629	96.78	98.86	97.84	99.54	98.35
1630	97.45	98.98	98.42	99.60	98.70
1631	94.91	99.43	95.43	99.49	97.39
1632	97.96	99.18	98.74	99.90	98.96
1633	96.06	97.94	97.97	99.66	97.96
1634	95.96	98.52	97.31	99.41	97.91
1635	92.13	97.85	93.91	98.80	95.84
1636	98.46	99.70	98.75	99.85	99.22
1637	96.84	99.15	97.64	99.49	98.39
1638	95.63	97.98	97.48	99.51	97.73
1639	96.14	96.49	99.60	99.85	98.02
1640	95.68	97.92	97.57	99.81	97.75
1641	90.11	91.01	98.74	99.71	94.72
1642	91.98	92.36	99.53	99.68	95.81
1643	93.56	95.56	97.64	99.55	96.59
1644	94.44	97.27	96.89	99.26	97.08
1645	93.73	98.42	95.07	99.42	96.71
1646	94.69	95.61	98.91	99.78	97.24
1647	94.02	94.74	99.12	99.81	96.88
1648	95.71	99.72	95.95	99.87	97.80
1649	96.11	99.38	96.66	99.73	98.00
1650	96.62	97.22	99.34	99.88	98.27
1651	96.73	97.34	99.34	99.75	98.33
1652	96.11	97.72	98.24	99.78	97.98
1653	94.05	94.56	99.40	99.70	96.92
1654	96.01	98.17	97.69	99.71	97.93
1655	97.15	98.95	98.13	99.66	98.54
1656	96.51	99.09	97.35	99.51	98.21
1657	94.97	96.05	98.80	99.24	97.40
1658	94.40	97.41	96.70	99.42	97.06
1659	93.47	97.08	96.01	99.05	96.54
1660	89.09	93.59	94.43	97.99	94.01
1661	96.09	97.77	98.18	99.70	97.97
1662	97.22	98.79	98.36	99.62	98.58
1663	95.40	97.91	97.31	99.29	97.61
1664	83.53	85.37	97.15	98.31	90.88
1665	95.38	97.57	97.61	99.59	97.59
1666	94.47	98.49	95.81	99.00	97.13
1667	96.18	98.96	97.14	99.27	98.04
1668	66.89	72.82	79.67	98.26	76.09
1669	95.91	98.63	97.14	99.84	97.88
1670	97.03	99.18	97.79	99.74	98.48
1671	97.37	98.01	99.30	99.86	98.65
1672	96.63	98.56	97.96	99.78	98.26
1673	92.32	93.78	98.17	99.51	95.93

1674	95.94	97.55	98.26	99.31	97.90
1675	96.66	99.11	97.51	99.19	98.30
1676	94.63	98.52	95.96	98.99	97.22
1677	93.06	95.30	97.34	99.37	96.31
1678	96.42	97.30	99.03	99.78	98.16
1679	88.52	94.72	92.43	99.59	93.56
1680	89.93	92.39	96.70	99.41	94.50
1681	93.24	94.49	98.47	99.60	96.44
1682	96.37	99.69	96.66	99.77	98.15
1683	97.77	98.63	99.09	99.95	98.86
1684	97.27	99.32	97.90	99.77	98.61
1685	96.72	98.77	97.86	99.76	98.31
1686	94.45	94.70	99.73	99.70	97.15
1687	95.02	97.90	96.89	99.83	97.39
1688	96.61	98.98	97.55	99.53	98.26
1689	95.53	97.61	97.74	99.25	97.68
1690	95.50	97.41	97.91	99.33	97.66
1691	95.67	97.52	97.99	99.33	97.76
1692	96.49	98.08	98.31	99.37	98.19
1693	96.31	99.31	96.95	99.42	98.12
1694	97.47	99.44	97.99	99.89	98.71
1695	97.27	98.98	98.23	99.72	98.60
1696	94.00	97.03	96.63	99.35	96.83
1697	93.25	98.30	94.71	98.95	96.47
1698	84.68	87.50	95.87	97.63	91.50
1699	92.41	94.98	96.87	99.76	95.92
1700	94.98	96.15	98.65	99.80	97.38
1701	96.28	98.00	98.14	99.74	98.07
1702	95.52	98.07	97.28	99.44	97.67
1703	67.69	75.53	77.53	98.76	76.52
1704	96.67	99.20	97.40	99.71	98.29
1705	96.12	97.55	98.43	99.60	97.99
1706	97.15	98.90	98.18	99.77	98.54
1707	93.18	95.62	97.09	99.82	96.35
1708	93.77	95.89	97.51	99.83	96.69
1709	98.34	99.44	98.87	99.92	99.16
1710	97.63	99.37	98.23	99.71	98.80
1711	97.12	98.57	98.48	99.47	98.53
1712	94.08	96.41	97.37	98.99	96.89
1713	89.12	90.87	97.66	98.81	94.14
1714	96.06	96.77	99.20	99.76	97.97
1715	91.89	94.35	96.93	99.70	95.62
1716	89.98	96.14	92.91	99.51	94.50
1717	96.83	98.53	98.21	99.87	98.37
1718	93.35	95.24	97.73	99.65	96.47
1719	93.92	97.06	96.48	99.79	96.77
1720	98.17	98.90	99.23	99.96	99.07
1721	96.71	98.67	97.95	99.65	98.31
1722	95.69	98.52	97.03	99.28	97.77
1723	93.73	98.65	94.92	98.91	96.75
1724	96.42	98.09	98.21	99.53	98.15
1725	96.37	97.82	98.44	99.49	98.13
1726	96.47	98.63	97.74	99.28	98.18
1727	94.81	98.57	96.09	99.10	97.31
1728	95.21	99.01	96.06	99.87	97.51
1729	95.85	97.74	97.96	99.42	97.85
1730	95.62	98.56	96.93	99.23	97.74
1731	93.66	97.08	96.25	98.70	96.66
1732	92.51	94.76	97.31	98.98	96.02
1733	94.07	97.13	96.59	99.61	96.86
1734	94.91	98.02	96.68	99.47	97.34
1735	94.15	97.93	95.95	99.31	96.93

1736	93.86	97.35	96.18	99.14	96.76
1737	93.26	95.45	97.38	99.73	96.41
1738	96.61	98.72	97.79	99.78	98.25
1739	94.06	94.97	98.90	99.83	96.89
1740	97.70	99.53	98.15	99.68	98.83
1741	96.88	98.29	98.50	99.63	98.40
1742	94.08	94.62	99.34	99.85	96.92
1743	94.63	95.51	98.95	99.81	97.20
1744	94.20	97.44	96.44	99.58	96.94
1745	94.43	97.27	96.88	99.22	97.07
1746	93.40	94.27	99.01	99.18	96.58
1747	97.35	97.75	99.56	99.94	98.65
1748	95.92	98.58	97.22	99.48	97.89
1749	96.36	98.73	97.54	99.38	98.13
1750	90.71	94.70	95.09	99.75	94.89
1751	95.76	98.41	97.20	99.69	97.80
1752	97.12	98.13	98.92	99.77	98.52
1753	96.62	98.01	98.52	99.51	98.26
1754	95.45	98.37	96.91	99.49	97.63
1755	95.28	97.44	97.62	99.61	97.53
1756	95.32	97.68	97.44	99.44	97.56
1757	94.89	98.44	96.28	99.34	97.34
1758	92.32	94.81	96.98	99.42	95.88
1759	92.04	93.89	97.68	99.40	95.75
1760	93.62	97.03	96.23	98.95	96.63
1761	94.32	98.81	95.31	99.87	97.03
1762	94.52	96.00	98.34	98.95	97.15
1763	94.28	96.52	97.48	99.19	97.00
1764	91.31	93.07	97.73	99.45	95.34
1765	92.21	94.58	97.09	99.55	95.82
1766	96.20	99.16	96.94	99.82	98.04
1767	95.97	98.80	97.06	99.66	97.92
1768	95.11	98.83	96.15	99.41	97.47
1769	98.73	99.55	99.17	99.84	99.36
1770	98.25	99.02	99.20	99.85	99.11
1771	97.20	98.73	98.40	99.85	98.56
1772	93.90	96.16	97.39	99.60	96.77
1773	95.32	98.73	96.43	99.88	97.57
1774	94.12	95.06	98.87	99.73	96.93
1775	95.27	96.36	98.75	99.72	97.54
1776	95.65	99.19	96.38	99.54	97.76
1777	94.60	97.02	97.32	99.11	97.17
1778	94.65	97.46	96.94	99.09	97.20
1779	96.31	99.07	97.15	99.58	98.10
1780	96.92	97.67	99.19	99.81	98.42
1781	96.33	96.85	99.42	99.91	98.12
1782	96.60	97.99	98.49	99.86	98.24
1783	94.70	97.78	96.66	99.56	97.22
1784	95.85	99.42	96.35	99.86	97.86
1785	92.85	95.70	96.64	99.66	96.17
1786	95.61	96.32	99.19	99.79	97.74
1787	92.26	97.71	94.09	99.51	95.87
1788	93.53	97.89	95.34	99.20	96.59
1789	96.49	96.90	99.55	99.73	98.21
1790	89.21	90.47	98.19	99.59	94.17
1791	90.32	94.75	94.60	99.39	94.67
1792	93.44	97.73	95.39	99.00	96.55
1793	90.76	96.67	93.49	98.17	95.05
1794	94.52	99.15	95.28	99.34	97.17
1795	94.56	97.98	96.33	99.51	97.15
1796	87.66	93.33	92.68	99.41	93.00
1797	89.79	94.62	94.11	99.27	94.36

1798	87.77	90.61	95.88	99.45	93.17
1799	93.97	96.85	96.77	99.37	96.81
1800	95.76	98.84	96.80	99.52	97.81
1801	96.07	97.82	98.09	99.75	97.96
1802	98.59	99.49	99.08	99.94	99.28
1803	97.58	98.96	98.57	99.93	98.76
1804	96.73	97.63	99.01	99.87	98.31
1805	96.33	99.11	97.13	99.61	98.11
1806	97.74	99.01	98.68	99.74	98.85
1807	97.89	98.29	99.58	99.87	98.93
1808	98.23	99.18	99.03	99.90	99.10
1809	95.95	96.87	98.97	99.89	97.90
1810	96.04	98.64	97.26	99.88	97.95
1811	96.78	98.24	98.45	99.71	98.34
1812	94.03	97.44	96.29	99.10	96.86
1813	90.85	96.33	93.86	98.26	95.08
1814	93.85	97.76	95.79	99.21	96.77
1815	95.68	96.15	99.46	99.84	97.78
1816	97.09	97.86	99.16	99.79	98.51
1817	95.76	97.24	98.36	99.88	97.79
1818	97.35	99.02	98.27	99.75	98.65
1819	95.58	99.16	96.33	99.57	97.72
1820	93.19	95.14	97.66	99.57	96.38
1821	96.27	97.84	98.30	99.85	98.07
1822	95.85	98.34	97.34	99.85	97.84
1823	91.17	93.34	97.17	99.64	95.22
1824	90.46	91.63	98.40	99.56	94.90
1825	92.92	96.74	95.71	99.27	96.22
1826	94.41	97.42	96.74	98.95	97.08
1827	91.93	97.46	94.02	98.84	95.71
1828	97.17	98.70	98.39	99.77	98.55
1829	91.39	92.88	98.10	99.23	95.42
1830	92.21	97.05	94.66	99.04	95.84
1831	92.96	96.34	96.21	98.28	96.28
1832	90.40	95.79	93.95	97.18	94.86
1833	93.49	95.74	97.46	98.29	96.60
1834	85.49	92.14	91.00	99.47	91.56
1835	91.62	93.42	97.67	99.69	95.50
1836	95.08	96.23	98.70	99.49	97.45
1837	96.06	97.46	98.47	99.47	97.96
1838	94.77	95.57	99.06	99.68	97.29
1839	97.16	98.70	98.39	99.75	98.54
1840	93.39	93.88	99.40	99.71	96.56
1841	92.73	97.55	94.74	99.54	96.13
1842	95.99	97.33	98.52	99.62	97.92
1843	98.80	99.10	99.69	99.92	99.39
1844	94.73	96.11	98.40	99.63	97.24
1845	94.78	96.62	97.96	99.02	97.28
1846	95.76	98.19	97.44	98.78	97.82
1847	96.68	98.56	98.05	99.00	98.30
1848	97.90	99.35	98.53	99.51	98.94
1849	97.77	99.37	98.38	99.49	98.87
1850	95.70	98.05	97.46	99.90	97.75
1851	96.83	98.76	98.01	99.01	98.38
1852	96.57	98.81	97.71	98.86	98.25
1853	97.04	98.93	98.05	99.19	98.49
1854	97.37	99.08	98.25	99.42	98.66
1855	99.01	99.65	99.36	99.79	99.50
1856	98.35	99.21	99.12	99.62	99.16
1857	97.99	99.37	98.59	99.61	98.98
1858	96.09	97.47	98.50	99.28	97.98
1859	98.32	99.21	99.09	99.67	99.15

1860	97.97	98.83	99.10	99.59	98.97
1861	93.81	94.51	99.14	99.81	96.77
1862	97.29	99.16	98.09	99.50	98.62
1863	97.44	98.75	98.64	99.55	98.69
1864	97.61	98.47	99.09	99.54	98.78
1865	97.40	98.25	99.10	99.50	98.68
1866	97.09	98.50	98.52	99.47	98.51
1867	96.38	98.37	97.90	99.24	98.14
1868	95.73	98.24	97.36	99.01	97.80
1869	95.69	98.98	96.64	99.08	97.80
1870	95.00	98.31	96.53	98.88	97.41
1871	94.56	97.73	96.61	98.71	97.17
1872	95.05	96.43	98.41	99.86	97.41
1873	94.97	98.13	96.67	98.72	97.40
1874	92.43	98.06	94.17	97.94	96.08
1875	90.29	95.75	93.91	96.82	94.82
1876	95.72	96.92	98.66	99.77	97.78
1877	95.02	96.66	98.15	99.44	97.40
1878	95.84	98.85	96.89	99.33	97.86
1879	96.85	99.20	97.59	99.68	98.39
1880	95.88	97.79	97.92	99.86	97.85
1881	96.17	96.73	99.37	99.74	98.04
1882	95.34	96.21	99.01	99.69	97.59
1883	90.06	92.30	96.95	99.66	94.57
1884	94.94	97.15	97.54	99.71	97.34
1885	96.68	97.11	99.52	99.89	98.30
1886	95.28	96.56	98.57	99.44	97.55
1887	96.89	99.11	97.73	99.33	98.42
1888	97.37	98.85	98.47	99.28	98.66
1889	97.81	98.94	98.83	99.44	98.88
1890	98.18	99.61	98.56	99.59	99.08
1891	97.40	99.19	98.18	99.28	98.68
1892	95.43	98.54	96.79	98.62	97.66
1893	97.51	98.91	98.56	99.30	98.73
1894	89.75	93.85	94.78	99.68	94.31
1895	98.04	99.14	98.87	99.54	99.01
1896	98.35	98.86	99.47	99.64	99.16
1897	98.25	99.01	99.22	99.57	99.11
1898	97.92	99.38	98.51	99.57	98.95
1899	97.62	99.67	97.95	99.49	98.80
1900	98.42	99.54	98.86	99.64	99.20
1901	97.23	99.07	98.11	99.39	98.59
1902	96.98	98.77	98.13	99.38	98.45
1903	97.07	98.72	98.28	99.50	98.50
1904	96.20	99.03	97.10	99.35	98.05
1905	91.00	94.12	96.06	99.74	95.08
1906	98.28	99.09	99.17	99.72	99.13
1907	97.74	99.61	98.11	99.67	98.86
1908	97.90	99.13	98.74	99.60	98.93
1909	97.33	99.44	97.85	99.55	98.64
1910	97.94	99.36	98.55	99.74	98.95
1911	96.25	98.88	97.29	99.25	98.08
1912	95.01	98.48	96.40	98.79	97.43
1913	96.22	97.69	98.42	99.14	98.05
1914	95.32	98.94	96.32	98.85	97.61
1915	94.69	98.94	95.68	98.81	97.28
1916	93.91	97.17	96.39	99.44	96.78
1917	97.32	98.37	98.89	99.71	98.63
1918	97.47	98.15	99.26	99.89	98.71
1919	94.07	98.02	95.77	99.43	96.88
1920	96.23	97.24	98.90	99.36	98.07
1921	95.04	98.12	96.73	99.30	97.42

1922	94.08	96.37	97.36	99.81	96.86
1923	93.27	96.77	96.03	99.76	96.40
1924	96.50	97.95	98.43	99.79	98.19
1925	96.07	97.09	98.86	99.90	97.96
1926	91.35	91.77	99.46	99.67	95.46
1927	86.58	87.38	98.79	99.39	92.74
1928	95.16	98.74	96.27	99.44	97.49
1929	93.70	94.37	99.19	99.70	96.72
1930	94.64	98.30	96.10	99.75	97.19
1931	95.17	97.04	97.91	99.77	97.47
1932	93.83	96.28	97.20	99.39	96.74
1933	95.81	97.50	98.15	99.56	97.83
1934	95.32	98.77	96.40	99.73	97.57
1935	95.01	98.22	96.57	99.79	97.39
1936	95.12	96.44	98.50	99.67	97.46
1937	92.29	93.57	98.36	99.69	95.91
1938	96.98	99.29	97.63	99.73	98.46
1939	95.91	97.26	98.50	99.74	97.88
1940	97.55	98.85	98.64	99.78	98.75
1941	96.78	98.11	98.57	99.92	98.34
1942	95.55	96.91	98.46	99.88	97.68
1943	97.46	98.05	99.37	99.91	98.70
1944	97.21	98.37	98.76	99.90	98.57
1945	96.13	98.28	97.71	99.73	97.99
1946	96.31	98.90	97.33	99.30	98.11
1947	96.91	98.98	97.86	99.42	98.42
1948	97.43	99.01	98.36	99.58	98.69
1949	97.32	98.93	98.33	99.53	98.63
1950	96.57	98.36	98.10	99.79	98.23
1951	97.89	98.49	99.37	99.92	98.92
1952	94.65	96.62	97.77	99.59	97.19
1953	93.96	96.69	96.91	99.62	96.80
1954	93.48	95.47	97.64	99.65	96.54
1955	93.59	94.08	99.40	99.78	96.67
1956	94.73	97.64	96.82	99.76	97.23
1957	94.60	96.11	98.26	99.64	97.17
1958	95.03	95.83	99.08	99.68	97.43
1959	93.52	94.50	98.83	99.47	96.61
1960	92.91	93.71	98.99	99.73	96.28
1961	95.66	97.27	98.21	99.77	97.74
1962	91.16	92.13	98.69	99.71	95.30
1963	94.58	96.94	97.36	99.35	97.15
1964	94.49	97.72	96.50	99.33	97.11
1965	96.52	97.28	99.16	99.77	98.21
1966	95.72	98.92	96.68	99.74	97.79
1967	94.40	96.35	97.77	99.70	97.05
1968	96.38	99.52	96.81	99.84	98.15
1969	95.93	96.78	99.04	99.82	97.90
1970	94.68	97.10	97.30	99.77	97.20
1971	95.92	98.08	97.68	99.81	97.88
1972	94.76	96.08	98.48	99.62	97.27
1973	91.88	93.23	98.26	99.58	95.68
1974	93.37	94.99	98.05	99.52	96.50
1975	96.40	98.85	97.46	99.48	98.15
1976	96.07	98.75	97.24	99.11	97.98
1977	96.05	98.91	97.04	99.47	97.97
1978	96.51	98.50	97.90	99.61	98.20
1979	96.86	97.58	99.20	99.87	98.39
1980	97.25	99.57	97.66	99.80	98.60
1981	97.88	98.68	99.15	99.91	98.92
1982	95.93	98.23	97.55	99.71	97.89
1983	93.47	95.19	97.92	99.79	96.54

1984	96.88	98.04	98.75	99.67	98.39
1985	95.31	97.08	98.04	99.52	97.56
1986	92.56	95.85	96.17	99.25	96.01
1987	93.75	95.08	98.39	99.63	96.71
1988	96.31	98.37	97.82	99.75	98.09
1989	92.67	94.83	97.42	99.00	96.11
1990	94.85	98.62	96.06	99.46	97.33
1991	95.73	96.64	98.96	99.84	97.79
1992	94.86	96.51	98.11	99.82	97.30
1993	95.13	97.08	97.82	99.71	97.45
1994	89.15	92.02	96.04	99.65	93.99
1995	91.19	95.98	94.41	99.75	95.19
1996	92.71	93.89	98.54	99.47	96.16
1997	90.24	95.61	93.74	98.91	94.67
1998	94.26	96.73	97.22	99.25	96.98
1999	93.03	95.82	96.83	98.40	96.32
2000	96.06	98.95	97.04	99.04	97.99
2001	96.32	99.03	97.21	99.45	98.11
2002	97.17	98.74	98.36	99.72	98.55
2003	95.99	99.30	96.63	99.55	97.94
2004	95.20	96.44	98.58	99.82	97.50
2005	94.71	95.66	98.89	99.75	97.25
2006	94.89	96.30	98.39	99.64	97.33
2007	97.21	98.72	98.42	99.64	98.57
2008	97.86	98.75	99.07	99.74	98.91
2009	99.12	99.33	99.78	99.97	99.55
2010	96.64	97.70	98.84	99.82	98.27
2011	94.99	96.22	98.61	99.49	97.40
2012	96.18	96.97	99.13	99.73	98.03
2013	94.61	96.63	97.72	99.50	97.17
2014	93.93	95.76	97.87	99.31	96.81
2015	91.80	96.84	94.38	99.17	95.59
2016	94.17	95.88	98.00	99.71	96.93
2017	96.03	98.90	97.03	99.50	97.96
2018	94.01	95.20	98.60	99.45	96.87
2019	95.22	95.83	99.29	99.88	97.53
2020	95.62	96.07	99.48	99.89	97.75
2021	93.61	96.94	96.26	99.75	96.60
2022	93.27	94.91	98.03	99.51	96.44
2023	92.03	97.18	94.37	98.81	95.76
2024	89.45	92.55	96.08	98.05	94.28
2025	94.79	96.77	97.78	99.34	97.27
2026	94.81	97.40	97.18	98.92	97.29
2027	93.90	95.13	98.53	99.68	96.80
2028	96.43	97.85	98.46	99.79	98.15
2029	95.03	98.16	96.70	98.85	97.43
2030	96.38	99.04	97.28	99.37	98.15
2031	96.93	99.10	97.78	99.48	98.43
2032	97.35	97.94	99.37	99.51	98.65
2033	93.14	93.64	99.38	99.77	96.43
2034	94.69	95.64	98.89	99.70	97.23
2035	95.39	96.87	98.34	99.66	97.60
2036	96.22	97.69	98.41	99.54	98.05
2037	94.83	98.56	96.08	99.53	97.31
2038	95.77	96.33	99.37	99.74	97.83
2039	97.06	97.48	99.54	99.91	98.50
2040	95.76	97.32	98.27	99.78	97.79
2041	94.22	95.41	98.59	99.72	96.97
2042	93.57	95.85	97.36	99.21	96.60
2043	93.47	96.51	96.58	98.96	96.55
2044	94.95	96.95	97.77	99.31	97.36
2045	93.52	94.58	98.71	99.63	96.60

2046	96.40	98.69	97.59	99.85	98.14
2047	97.76	98.66	99.05	99.95	98.86
2048	96.19	97.27	98.80	99.78	98.03
2049	96.67	97.96	98.61	99.81	98.28
2050	94.27	96.60	97.35	99.73	96.97
2051	95.51	96.87	98.48	99.61	97.67
2052	95.59	98.18	97.25	99.36	97.71
2053	94.96	98.74	96.09	99.17	97.39
2054	94.50	96.03	98.27	99.02	97.14
2055	94.14	97.14	96.68	99.36	96.91
2056	94.88	98.66	96.09	99.01	97.36
2057	94.84	98.38	96.30	98.83	97.33
2058	93.21	97.04	95.78	98.90	96.41
2059	94.64	98.17	96.26	99.13	97.21
2060	96.31	98.61	97.59	99.33	98.10
2061	93.48	95.56	97.56	99.40	96.55
2062	94.21	95.28	98.71	99.78	96.97
2063	94.33	96.17	97.89	99.57	97.02
2064	94.19	96.16	97.73	99.60	96.94
2065	92.57	93.05	99.39	99.80	96.12
2066	92.64	93.93	98.36	99.76	96.09
2067	93.93	97.01	96.54	99.77	96.78
2068	93.56	94.50	98.84	99.81	96.62
2069	94.07	96.86	96.86	99.73	96.86
2070	91.13	92.12	98.69	99.58	95.29
2071	93.30	94.41	98.65	99.57	96.48
2072	95.37	96.63	98.59	99.40	97.60
2073	93.96	95.35	98.36	99.58	96.83
2074	93.78	94.50	99.14	99.61	96.76
2075	90.68	91.63	98.70	99.70	95.03
2076	96.36	97.52	98.73	99.80	98.12
2077	96.06	97.35	98.57	99.78	97.96
2078	97.04	99.08	97.90	99.79	98.48
2079	97.08	99.23	97.79	99.78	98.51
2080	89.02	92.02	95.86	99.66	93.90
2081	92.60	94.24	98.00	99.34	96.08
2082	94.52	97.12	97.11	99.30	97.12
2083	87.26	90.49	95.29	99.35	92.83
2084	94.16	96.79	97.07	99.07	96.93
2085	93.90	98.19	95.50	98.75	96.83
2086	94.92	98.18	96.57	98.99	97.37
2087	93.95	98.47	95.32	98.82	96.86
2088	93.31	96.15	96.78	98.66	96.47
2089	95.28	98.22	96.88	99.40	97.54
2090	93.31	93.97	99.27	99.13	96.55
2091	95.27	96.98	98.12	99.24	97.54
2092	96.33	98.73	97.49	99.79	98.10
2093	94.70	95.18	99.45	99.67	97.27
2094	90.19	94.45	94.74	99.51	94.59
2095	93.32	95.50	97.40	99.75	96.44
2096	85.58	86.83	97.96	99.37	92.06
2097	92.64	94.84	97.32	99.70	96.06
2098	79.33	79.84	99.28	99.21	88.50
2099	95.71	97.09	98.48	99.66	97.78
2100	96.69	97.48	99.14	99.78	98.30
2101	97.71	98.69	98.96	99.91	98.83
2102	97.47	99.22	98.20	99.77	98.71
2103	94.24	95.89	98.09	99.37	96.98
2104	94.03	97.07	96.62	99.49	96.85
2105	90.42	93.99	95.48	99.69	94.73
2106	94.01	98.49	95.31	99.32	96.87
2107	93.41	97.82	95.30	98.82	96.54

2108	95.50	97.85	97.48	99.16	97.67
2109	95.63	98.52	96.97	99.21	97.74
2110	93.66	94.76	98.77	98.83	96.72
2111	94.80	97.68	96.89	99.06	97.28
2112	91.57	95.56	95.38	98.55	95.47
2113	95.72	97.99	97.57	99.40	97.78
2114	94.81	97.86	96.74	98.98	97.30
2115	96.64	97.26	99.32	99.87	98.28
2116	96.57	97.91	98.55	99.88	98.23
2117	97.09	98.58	98.42	99.89	98.50
2118	97.70	98.32	99.34	99.89	98.83
2119	97.25	98.27	98.91	99.87	98.59
2120	93.17	95.33	97.40	99.78	96.35
2121	94.98	97.67	97.06	99.69	97.37
2122	95.64	97.17	98.29	99.83	97.73
2123	92.65	92.85	99.77	99.76	96.19
2124	95.93	99.34	96.52	99.68	97.91
2125	93.12	93.77	99.25	99.39	96.43
2126	97.91	98.77	99.09	99.89	98.93
2127	93.82	96.04	97.44	99.38	96.73
2128	97.06	98.74	98.25	99.53	98.49
2129	96.74	98.17	98.48	99.51	98.33
2130	94.03	96.20	97.52	99.28	96.85
2131	92.51	97.06	95.02	98.64	96.03
2132	96.02	96.61	99.35	99.53	97.96
2133	94.69	95.75	98.75	99.80	97.23
2134	95.39	96.98	98.24	99.53	97.60
2135	94.23	96.67	97.25	99.29	96.96
2136	95.71	98.26	97.29	99.54	97.77
2137	97.07	98.81	98.19	99.68	98.50
2138	93.82	96.66	96.79	99.40	96.72
2139	96.81	98.18	98.53	99.90	98.35
2140	94.78	98.28	96.32	99.06	97.29
2141	96.60	98.43	98.06	99.88	98.24
2142	94.52	96.71	97.51	99.72	97.11
2143	97.22	97.94	99.22	99.84	98.58
2144	97.48	98.57	98.85	99.77	98.71
2145	97.54	98.64	98.85	99.91	98.74
2146	96.36	97.06	99.22	99.75	98.13
2147	96.86	99.10	97.69	99.80	98.39
2148	95.22	98.31	96.71	99.69	97.50
2149	95.84	98.24	97.44	99.58	97.84
2150	96.76	98.42	98.23	99.92	98.33
2151	95.10	95.91	99.07	99.65	97.46
2152	95.28	97.00	98.07	99.78	97.53
2153	88.35	96.79	90.55	99.55	93.57
2154	91.60	92.62	98.65	99.71	95.54
2155	93.74	94.72	98.81	99.69	96.72
2156	94.55	97.21	97.04	99.77	97.13
2157	95.96	96.39	99.52	99.80	97.93
2158	96.50	97.76	98.63	99.69	98.19
2159	96.50	97.68	98.71	99.77	98.19
2160	97.78	98.98	98.76	99.82	98.87
2161	94.84	97.51	97.07	99.67	97.29
2162	97.42	99.52	97.88	99.65	98.69
2163	95.01	97.02	97.76	99.50	97.39
2164	91.90	97.69	93.77	99.14	95.69
2165	93.59	97.32	95.90	99.36	96.60
2166	96.16	96.94	99.13	99.75	98.02
2167	98.14	99.09	99.02	99.81	99.05
2168	97.54	98.91	98.58	99.72	98.74
2169	96.41	98.86	97.44	99.80	98.15

2170	93.40	96.67	96.30	99.52	96.49
2171	96.75	96.92	99.81	99.88	98.35
2172	94.73	96.77	97.72	99.23	97.24
2173	95.29	98.82	96.32	99.77	97.55
2174	98.12	99.76	98.35	99.96	99.05
2175	97.44	98.20	99.19	99.86	98.69
2176	97.07	97.87	99.14	99.89	98.50
2177	94.54	97.07	97.18	99.64	97.12
2178	95.18	97.01	97.96	99.44	97.48
2179	96.05	96.68	99.31	99.46	97.98
2180	97.22	99.12	98.04	99.69	98.58
2181	96.44	99.40	96.98	99.72	98.18
2182	97.33	98.69	98.58	99.80	98.63
2183	95.39	98.76	96.51	99.21	97.63
2184	97.54	98.30	99.19	99.91	98.74
2185	94.93	96.20	98.52	99.82	97.35
2186	94.99	96.58	98.19	99.84	97.38
2187	96.56	98.69	97.76	99.81	98.22
2188	95.97	98.52	97.30	99.73	97.91
2189	91.08	91.41	99.58	99.70	95.32
2190	96.99	98.38	98.52	99.83	98.45
2191	97.81	98.67	99.10	99.75	98.88
2192	98.08	98.82	99.22	99.85	99.02
2193	96.32	97.05	99.18	99.83	98.10
2194	87.35	92.65	92.96	99.43	92.80
2195	95.65	97.73	97.73	99.60	97.73
2196	96.55	99.09	97.39	99.53	98.23
2197	96.81	99.24	97.51	99.61	98.37
2198	96.45	97.88	98.46	99.63	98.17
2199	96.73	98.46	98.16	99.78	98.31
2200	90.36	91.54	98.39	99.52	94.84
2201	92.12	93.29	98.48	99.74	95.81
2202	90.63	91.26	99.15	99.65	95.04
2203	89.68	94.34	94.19	99.70	94.27
2204	93.44	97.83	95.27	99.37	96.53
2205	97.27	98.77	98.43	99.81	98.60
2206	95.81	98.66	97.03	99.30	97.84
2207	96.52	98.23	98.18	99.40	98.21
2208	95.63	98.64	96.85	99.55	97.74
2209	95.10	98.42	96.50	99.49	97.45
2210	93.19	98.05	94.83	99.25	96.41
2211	96.08	98.74	97.22	99.71	97.97
2212	95.47	97.03	98.28	99.30	97.65
2213	96.08	98.67	97.31	99.25	97.98
2214	97.11	98.49	98.55	99.49	98.52
2215	90.86	93.44	96.68	99.51	95.03
2216	96.51	98.71	97.70	99.78	98.20
2217	94.77	97.86	96.67	99.37	97.26
2218	95.25	98.97	96.17	99.41	97.55
2219	96.18	98.41	97.65	99.57	98.03
2220	93.36	94.13	99.14	99.12	96.57
2221	95.20	97.85	97.16	99.10	97.50
2222	95.79	98.68	97.00	99.12	97.83
2223	91.35	97.61	93.30	98.63	95.41
2224	95.16	98.07	96.91	99.06	97.49
2225	95.19	98.08	96.93	99.03	97.50
2226	95.86	98.46	97.28	99.17	97.87
2227	95.25	98.19	96.85	99.77	97.52
Average	94.65	96.84	97.55	99.56	97.17
Standard c	2.89	2.72	1.53	0.32	1.70
Confidence	0.12	0.11	0.06	0.01	0.07
