# Supplementary material for: In Situ Root Dataset Expansion Strategy Based on an Improved CycleGAN Generator
Source: Plant Phenomics. 2024 Feb 12;6:0148. doi: 10.34133/plantphenomics.0148 (PMC11020132; doi:10.34133/plantphenomics.0148)
Supplement: Supplementary 1 — The network and corresponding weights can be viewed on GitHub (https://github.com/jiwd123/improved_cyclegan) and Zenodo (https://doi.org/10.5281/zenodo.10460303). [file plantphenomics.0148.f1.zip › generalization evaluation_Wn.pdf]

No.	IOU	Recall	Precision	Accuracy	F1
1	91.24	99.13	92.00	99.06	95.43
2	85.92	98.20	87.22	98.62	92.39
3	80.95	98.27	82.29	97.91	89.58
4	76.81	98.46	78.35	97.04	87.26
5	78.77	99.25	79.52	98.54	88.30
6	72.80	98.56	74.06	97.64	84.57
7	77.77	91.24	82.09	97.96	86.42
8	63.22	98.14	64.82	96.90	78.07
9	64.58	96.92	67.66	94.05	79.69
10	53.35	97.85	55.50	95.72	70.82
11	49.13	49.13	50.00	98.26	49.56
12	52.06	98.16	53.90	96.34	69.59
13	68.36	98.24	69.96	96.91	81.72
14	49.42	49.42	50.00	98.84	49.71
15	62.44	97.61	64.83	95.32	77.91
16	52.75	65.54	57.31	92.65	61.15
17	48.93	48.93	50.00	97.86	49.46
18	48.28	48.28	50.00	96.57	49.13
19	49.35	49.35	50.00	98.71	49.67
20	47.88	47.88	50.00	95.76	48.92
21	58.95	96.41	61.12	95.83	74.81
22	48.70	48.71	49.99	97.40	49.34
23	48.99	48.99	50.00	97.99	49.49
24	88.90	99.64	89.18	99.70	94.12
25	75.97	98.40	77.47	97.16	86.69
26	67.48	97.13	69.88	95.49	81.28
27	94.34	99.22	95.03	99.39	97.08
28	89.41	99.38	90.03	98.82	94.47
29	79.83	98.64	81.19	97.39	89.07
30	78.35	98.66	79.70	97.41	88.17
31	62.59	97.43	65.16	94.97	78.09
32	51.48	96.27	55.02	92.98	70.02
33	65.19	98.29	66.52	97.45	79.34
34	93.20	99.51	93.66	99.30	96.50
35	93.21	99.17	93.93	99.26	96.48
36	93.88	99.13	94.64	99.34	96.83
37	88.55	99.31	89.24	98.71	94.00
38	55.42	94.84	58.52	93.95	72.38
39	91.50	99.41	92.07	98.98	95.60
40	92.02	99.51	92.51	99.09	95.88
41	89.48	98.48	90.71	98.68	94.44
42	92.42	99.27	93.06	99.28	96.06
43	62.81	91.13	64.83	97.31	75.76
44	60.74	98.51	61.76	98.01	75.92
45	50.43	79.75	52.11	96.67	63.03
46	56.22	98.08	58.03	96.40	72.92
47	64.08	98.40	65.63	96.95	78.74
48	48.90	48.90	50.00	97.80	49.44
49	48.57	48.57	50.00	97.13	49.27
50	48.29	48.29	50.00	96.59	49.13
51	68.20	87.63	71.57	97.89	78.79
52	61.55	94.30	63.11	97.47	75.61
53	48.11	48.11	50.00	96.22	49.04
54	48.99	48.99	50.00	97.97	49.49
55	49.82	99.29	50.53	98.57	66.97
56	51.27	57.74	53.02	97.46	55.28
57	48.85	48.85	50.00	97.69	49.42
58	48.85	48.85	50.00	97.69	49.42
59	48.63	48.63	50.00	97.26	49.30
60	48.61	48.61	50.00	97.22	49.29
61	85.28	99.65	85.64	99.30	92.11

62	93.27	99.41	93.81	99.31	96.53
63	90.02	99.44	90.58	98.93	94.80
64	91.18	99.43	91.73	99.01	95.43
65	93.83	99.52	94.30	99.17	96.84
66	48.53	48.53	50.00	97.06	49.25
67	78.32	98.61	79.68	97.41	88.14
68	87.33	92.39	93.37	98.36	92.88
69	92.73	99.51	93.21	99.10	96.26
70	91.61	98.11	93.11	99.19	95.54
71	48.38	48.38	50.00	96.77	49.18
72	52.28	71.62	54.15	96.72	61.67
73	48.81	48.98	49.82	97.63	49.40
74	64.90	97.30	65.83	98.54	78.53
75	49.38	49.38	50.00	98.77	49.69
76	63.93	78.22	69.09	97.37	73.37
77	48.23	48.23	50.00	96.45	49.10
78	62.95	69.37	73.88	97.83	71.56
79	48.92	48.92	50.00	97.84	49.45
80	48.75	48.75	50.00	97.50	49.37
81	49.02	49.02	50.00	98.04	49.50
82	49.02	49.02	50.00	98.04	49.50
83	49.35	49.35	50.00	98.70	49.67
84	49.35	49.35	50.00	98.70	49.67
85	49.07	49.07	50.00	98.14	49.53
86	53.13	97.96	54.99	96.30	70.44
87	48.88	48.88	50.00	97.75	49.43
88	48.98	48.98	50.00	97.95	49.48
89	49.40	49.40	50.00	98.81	49.70
90	48.92	48.92	50.00	97.83	49.45
91	48.96	48.96	50.00	97.92	49.48
92	61.94	98.52	63.35	97.22	77.11
93	49.15	49.15	50.00	98.29	49.57
94	65.33	85.01	68.18	98.85	75.67
95	49.23	49.23	50.00	98.45	49.61
96	48.84	48.84	50.00	97.68	49.41
97	49.19	49.19	50.00	98.37	49.59
98	48.59	48.59	50.00	97.18	49.28
99	48.35	48.35	50.00	96.71	49.16
100	52.57	94.50	54.17	96.84	68.87
101	48.69	48.69	50.00	97.38	49.34
102	48.20	48.20	50.00	96.41	49.09
103	72.42	95.24	74.32	97.96	83.49
104	71.51	94.35	73.53	98.04	82.65
105	49.16	49.16	50.00	98.33	49.58
106	49.16	49.16	50.00	98.33	49.58
107	49.29	49.29	50.00	98.57	49.64
108	49.29	49.29	50.00	98.57	49.64
109	72.29	99.01	72.78	99.28	83.89
110	48.23	48.23	50.00	96.45	49.10
111	57.75	69.26	61.29	98.45	65.03
112	69.79	96.19	72.03	96.29	82.38
113	78.30	98.12	79.86	97.30	88.05
114	67.38	98.65	68.45	97.97	80.82
115	49.13	49.13	50.00	98.25	49.56
116	49.01	49.01	50.00	98.03	49.50
117	48.93	48.93	50.00	97.87	49.46
118	56.55	98.08	58.47	96.19	73.27
119	88.70	97.76	90.41	98.50	93.94
120	75.88	81.73	88.40	96.66	84.94
121	82.20	98.19	83.53	98.13	90.27
122	79.77	93.26	83.31	98.17	88.00
123	81.34	98.70	82.40	98.25	89.82

124	48.96	98.41	50.55	96.82	66.79
125	49.13	49.13	50.00	98.25	49.56
126	49.40	49.40	50.00	98.80	49.70
127	57.94	98.85	58.92	98.06	73.83
128	49.42	49.42	50.00	98.84	49.71
129	48.76	48.76	50.00	97.52	49.37
130	48.77	48.77	50.00	97.54	49.38
131	84.91	99.77	85.14	99.54	91.88
132	85.59	99.32	86.26	98.73	92.33
133	71.99	96.13	73.70	97.83	83.43
134	94.04	99.47	94.54	99.29	96.94
135	88.21	98.87	89.20	98.49	93.79
136	82.98	98.95	84.03	97.99	90.88
137	90.24	99.34	90.86	98.94	94.91
138	84.72	98.74	85.91	97.88	91.88
139	77.36	98.16	79.17	96.58	87.65
140	90.70	98.11	92.28	98.39	95.11
141	73.59	90.39	77.96	96.12	83.71
142	51.47	61.94	52.63	98.20	56.91
143	51.87	98.63	53.24	97.27	69.15
144	62.20	79.07	66.09	97.49	72.00
145	61.21	98.89	62.32	97.80	76.46
146	78.20	98.72	79.17	98.41	87.87
147	68.75	97.52	71.14	95.41	82.27
148	85.46	97.93	87.12	97.72	92.21
149	93.51	99.41	94.06	99.22	96.66
150	90.75	99.22	91.37	99.45	95.13
151	73.53	98.86	74.64	97.84	85.06
152	78.39	98.38	79.88	97.26	88.17
153	92.10	99.53	92.57	99.11	95.93
154	91.87	99.40	92.45	99.01	95.80
155	48.36	48.36	50.00	96.73	49.17
156	67.42	97.32	70.05	94.94	81.46
157	83.07	98.33	84.64	97.23	90.97
158	90.70	98.98	91.58	98.89	95.14
159	92.01	99.10	92.78	99.13	95.84
160	78.22	98.30	79.81	97.07	88.09
161	67.89	97.34	69.73	96.66	81.25
162	63.08	98.67	64.41	97.38	77.94
163	86.78	94.59	90.61	98.62	92.56
164	86.24	92.70	91.55	98.93	92.12
165	47.74	47.74	50.00	95.48	48.84
166	48.86	48.86	50.00	97.72	49.42
167	47.74	47.74	50.00	95.48	48.84
168	67.82	78.22	76.24	96.75	77.22
169	70.53	97.76	72.64	95.97	83.35
170	46.91	96.29	50.63	92.58	66.36
171	89.74	98.47	91.00	98.60	94.59
172	92.31	99.42	92.86	99.11	96.03
173	89.07	98.15	90.38	99.39	94.10
174	68.21	98.10	69.73	97.14	81.52
175	75.82	98.00	77.69	96.52	86.67
176	82.92	99.01	83.86	98.25	90.81
177	49.09	49.09	50.00	98.17	49.54
178	79.18	99.54	79.64	99.09	88.48
179	56.89	98.44	58.45	96.90	73.35
180	48.14	48.14	50.00	96.27	49.05
181	56.18	98.16	58.02	96.35	72.93
182	48.10	48.10	50.00	96.20	49.03
183	49.11	49.11	50.00	98.21	49.55
184	83.76	89.63	91.30	98.68	90.46
185	49.62	53.95	52.19	95.15	53.06

186	49.05	49.05	50.00	98.10	49.52
187	52.56	78.38	54.29	96.84	64.15
188	85.27	97.18	86.92	99.53	91.77
189	52.62	97.96	54.66	95.94	70.16
190	49.39	49.39	50.00	98.78	49.69
191	49.00	49.00	50.00	97.99	49.49
192	81.47	98.62	82.62	98.09	89.91
193	65.15	89.33	68.24	96.20	77.37
194	49.03	49.03	50.00	98.06	49.51
195	48.36	48.36	50.00	96.72	49.17
196	48.99	97.69	51.30	95.39	67.28
197	48.85	48.85	50.00	97.71	49.42
198	48.41	48.41	50.00	96.82	49.19
199	69.32	89.87	72.28	97.91	80.12
200	48.26	48.40	49.85	96.53	49.12
201	47.65	97.61	50.03	95.23	66.16
202	47.61	47.61	50.00	95.22	48.78
203	67.95	77.17	76.55	98.46	76.86
204	74.83	88.72	79.68	98.05	83.96
205	48.75	98.54	50.21	97.09	66.52
206	51.31	76.19	52.41	97.90	62.10
207	49.25	49.25	50.00	98.50	49.62
208	49.98	52.78	51.89	96.86	52.33
209	76.66	96.40	78.78	97.21	86.70
210	71.60	99.32	72.28	98.65	83.67
211	49.63	96.90	51.67	95.93	67.40
212	49.10	49.10	50.00	98.19	49.54
213	66.88	95.36	68.19	98.41	79.52
214	59.33	93.09	60.90	97.38	73.63
215	48.21	48.21	50.00	96.42	49.09
216	49.32	49.32	50.00	98.63	49.66
217	48.88	97.96	50.92	95.92	67.01
218	48.13	97.93	50.20	95.86	66.38
219	47.77	47.77	50.00	95.54	48.86
220	77.83	97.99	79.16	98.06	87.57
221	66.59	98.91	67.68	97.85	80.37
222	48.79	49.25	49.53	97.59	49.39
223	68.05	83.81	72.84	97.66	77.94
224	48.02	48.02	50.00	96.05	48.99
225	49.86	98.71	51.16	97.42	67.39
226	48.96	97.67	51.29	95.34	67.26
227	57.13	89.61	59.65	95.51	71.62
228	49.62	99.17	50.45	98.33	66.88
229	48.61	48.61	50.00	97.23	49.30
230	47.31	47.31	50.00	94.62	48.62
231	57.13	97.71	58.44	97.44	73.13
232	48.76	48.76	50.00	97.53	49.37
233	48.34	48.34	50.00	96.68	49.16
234	49.25	49.25	50.00	98.51	49.62
235	50.85	95.81	52.77	96.17	68.06
236	62.03	96.66	63.66	97.02	76.76
237	48.88	48.88	50.00	97.76	49.43
238	48.04	48.04	50.00	96.07	49.00
239	58.35	98.51	59.84	97.04	74.46
240	48.25	48.25	50.00	96.51	49.11
241	48.31	48.48	49.82	96.62	49.14
242	53.42	96.94	54.98	96.91	70.16
243	55.34	92.17	58.14	94.63	71.31
244	49.31	49.31	50.00	98.61	49.65
245	47.02	47.02	50.00	94.04	48.47
246	49.22	49.22	50.00	98.44	49.61
247	48.99	48.99	50.00	97.98	49.49

248	48.13	48.13	50.00	96.26	49.05
249	49.07	49.07	50.00	98.13	49.53
250	53.76	89.37	54.65	98.39	67.83
251	49.03	49.13	49.90	98.07	49.51
252	49.07	49.07	50.00	98.14	49.53
253	49.27	49.27	50.00	98.55	49.63
254	48.70	48.89	49.80	97.40	49.34
255	49.31	49.31	50.00	98.61	49.65
256	48.58	48.58	50.00	97.16	49.28
257	48.60	48.60	50.00	97.20	49.29
258	48.80	52.14	50.29	96.76	51.20
259	48.98	48.98	50.00	97.96	49.48
260	48.05	48.05	50.00	96.10	49.00
261	49.04	49.04	50.00	98.08	49.51
262	49.28	49.38	49.89	98.55	49.64
263	52.00	59.11	58.53	91.78	58.82
264	54.58	90.04	57.84	93.74	70.43
265	48.16	48.23	49.92	96.31	49.06
266	69.23	90.93	71.89	97.90	80.30
267	48.39	48.39	50.00	96.78	49.18
268	47.48	47.49	49.98	94.95	48.71
269	48.94	48.94	50.00	97.88	49.46
270	49.01	49.24	49.76	98.01	49.50
271	49.24	49.24	50.00	98.49	49.62
272	53.03	59.96	56.10	97.08	57.96
273	48.56	97.80	50.75	95.61	66.83
274	48.45	98.36	50.09	96.72	66.38
275	49.20	49.20	50.00	98.40	49.60
276	49.20	49.20	50.00	98.40	49.60
277	71.94	94.63	74.22	97.29	83.19
278	49.16	49.16	50.00	98.33	49.58
279	48.85	48.85	50.00	97.70	49.42
280	48.20	48.20	50.00	96.40	49.08
281	60.36	93.13	62.48	96.38	74.78
282	48.85	48.85	50.00	97.71	49.42
283	48.46	49.07	49.44	96.86	49.26
284	49.14	49.14	50.00	98.28	49.57
285	49.00	49.00	50.00	98.00	49.49
286	49.18	49.18	50.00	98.36	49.59
287	55.26	85.23	56.72	97.61	68.11
288	51.26	97.42	53.63	95.28	69.18
289	53.38	95.16	56.14	94.55	70.61
290	53.16	70.88	56.19	94.93	62.69
291	49.30	49.30	50.00	98.60	49.65
292	49.12	49.12	50.00	98.23	49.55
293	48.31	48.31	50.00	96.61	49.14
294	48.87	96.37	51.20	95.35	66.87
295	48.35	48.36	50.00	96.70	49.16
296	69.69	94.71	71.52	97.92	81.50
297	77.70	99.38	78.28	98.88	87.58
298	49.22	49.22	50.00	98.45	49.61
299	48.23	48.23	50.00	96.46	49.10
300	49.05	49.05	50.00	98.10	49.52
301	49.26	49.26	50.00	98.52	49.63
302	49.26	49.26	50.00	98.52	49.63
303	49.61	52.90	50.46	98.20	51.65
304	51.67	99.24	52.43	98.48	68.61
305	58.01	98.16	59.76	96.54	74.29
306	49.88	98.19	51.69	96.38	67.73
307	48.92	48.92	50.00	97.85	49.46
308	49.71	98.55	51.16	97.11	67.35
309	49.25	51.57	50.17	98.04	50.86

310	69.60	98.93	70.31	98.73	82.20
311	48.39	50.42	50.03	96.47	50.23
312	51.34	95.74	53.97	94.76	69.03
313	57.11	97.76	58.85	96.56	73.47
314	54.27	78.19	55.50	98.22	64.92
315	52.74	96.52	54.09	97.32	69.33
316	66.85	96.25	68.90	96.46	80.31
317	57.31	90.97	59.43	96.22	71.89
318	52.88	96.80	54.88	96.03	70.04
319	61.13	94.86	63.28	96.14	75.92
320	60.72	93.54	62.52	96.99	74.95
321	82.18	97.29	84.07	97.62	90.20
322	56.61	97.43	57.76	97.77	72.53
323	65.41	98.73	66.67	97.50	79.60
324	63.74	97.99	65.76	96.04	78.70
325	48.99	48.99	50.00	97.98	49.49
326	65.23	85.05	68.76	97.26	76.05
327	66.28	81.17	72.11	96.05	76.37
328	51.99	97.26	54.40	95.20	69.77
329	73.16	97.94	74.96	96.65	84.92
330	84.19	98.75	85.39	97.81	91.59
331	77.85	96.85	80.09	96.60	87.67
332	69.00	97.22	71.54	95.18	82.43
333	59.59	99.43	60.16	98.86	74.96
334	49.35	97.84	51.51	95.69	67.49
335	72.29	98.10	74.17	96.38	84.48
336	79.26	98.22	81.01	96.71	88.79
337	73.97	98.83	75.06	97.92	85.32
338	65.84	95.21	68.85	94.63	79.91
339	74.38	84.51	83.29	94.15	83.90
340	80.31	95.80	82.89	97.27	88.88
341	86.35	98.69	87.51	98.14	92.76
342	84.44	97.05	86.50	97.85	91.47
343	85.83	98.23	87.23	98.13	92.40
344	56.24	98.44	57.81	96.89	72.84
345	85.30	98.57	86.63	97.73	92.21
346	76.38	97.78	78.36	96.38	87.00
347	83.83	98.09	85.32	97.89	91.26
348	91.20	98.74	92.28	98.74	95.40
349	81.42	96.97	83.54	97.25	89.75
350	47.46	95.97	51.49	91.96	67.02
351	84.27	98.07	85.86	97.60	91.56
352	83.87	90.56	90.72	97.52	90.64
353	76.16	98.64	77.51	97.41	86.81
354	49.36	49.36	50.00	98.72	49.68
355	48.86	48.86	50.00	97.72	49.42
356	48.09	48.09	50.00	96.19	49.03
357	50.54	97.36	52.21	96.67	67.97
358	55.87	93.24	56.93	98.10	70.69
359	48.31	98.00	50.30	96.01	66.48
360	55.40	97.93	57.07	96.71	72.11
361	66.28	91.60	68.76	97.00	78.55
362	61.87	97.49	64.03	95.78	77.30
363	51.19	96.90	52.98	96.44	68.51
364	61.91	97.08	64.06	95.87	77.19
365	64.44	97.51	66.93	95.14	79.38
366	48.91	48.91	50.00	97.82	49.45
367	81.77	98.22	83.43	97.03	90.22
368	48.52	94.32	53.08	90.93	67.93
369	79.55	98.60	80.88	97.49	88.87
370	66.51	98.08	68.43	96.24	80.62
371	80.11	97.90	82.01	96.64	89.25

372	74.14	96.96	77.13	94.45	85.91
373	80.09	97.92	81.99	96.64	89.25
374	69.77	98.21	71.55	96.52	82.79
375	86.41	98.79	87.50	98.19	92.80
376	76.45	97.60	78.69	95.89	87.13
377	82.08	89.83	88.70	98.53	89.26
378	78.89	98.23	80.63	96.73	88.56
379	77.95	93.62	81.60	96.26	87.19
380	75.40	97.64	77.75	95.57	86.57
381	81.55	98.53	83.02	97.24	90.11
382	86.61	98.53	87.95	97.78	92.94
383	78.74	87.57	86.66	95.90	87.11
384	86.45	95.25	89.84	97.91	92.46
385	84.21	98.71	85.41	97.87	91.58
386	74.01	83.66	82.87	95.83	83.26
387	84.49	98.90	85.54	98.11	91.73
388	53.87	97.30	56.47	94.84	71.46
389	88.88	98.72	89.91	98.81	94.11
390	60.71	97.37	63.34	94.84	76.76
391	78.04	97.59	79.99	96.72	87.92
392	80.32	97.38	82.36	96.82	89.24
393	75.64	97.18	77.87	96.17	86.46
394	87.05	97.04	89.15	98.45	92.93
395	86.37	99.01	87.23	98.63	92.75
396	83.53	98.76	84.62	98.14	91.15
397	76.24	98.53	77.57	97.53	86.81
398	49.16	49.16	50.00	98.32	49.58
399	91.19	99.34	91.80	99.00	95.42
400	49.16	49.16	50.00	98.32	49.58
401	48.39	48.39	50.00	96.79	49.18
402	57.55	98.24	59.30	96.52	73.96
403	49.32	49.32	50.00	98.64	49.66
404	59.22	91.88	61.11	96.83	73.40
405	78.43	97.69	80.04	97.58	87.99
406	48.52	48.52	50.00	97.04	49.25
407	58.28	98.35	59.93	96.72	74.48
408	74.53	98.28	76.25	96.68	85.88
409	81.69	95.44	84.57	97.25	89.68
410	62.49	98.16	64.33	96.37	77.73
411	72.57	97.89	74.62	96.09	84.69
412	80.51	97.76	82.26	97.25	89.34
413	91.34	98.92	92.30	98.74	95.50
414	77.74	95.99	80.37	96.36	87.49
415	85.89	96.71	88.41	97.16	92.37
416	63.37	96.96	66.41	94.10	78.83
417	65.23	98.83	66.39	97.69	79.43
418	74.90	97.24	76.70	97.18	85.76
419	87.06	98.86	88.15	98.08	93.20
420	72.88	85.55	78.88	97.91	82.08
421	48.22	48.22	50.00	96.44	49.09
422	69.74	98.26	70.86	98.03	82.34
423	66.41	97.22	68.40	96.32	80.30
424	70.07	98.73	71.12	98.01	82.68
425	48.80	48.80	50.00	97.60	49.39
426	48.80	48.80	50.00	97.60	49.39
427	49.34	49.34	50.00	98.67	49.67
428	49.29	49.34	49.95	98.58	49.64
429	51.67	99.37	52.30	98.73	68.53
430	48.64	97.04	51.59	94.10	67.37
431	57.79	96.79	60.61	94.46	74.54
432	91.12	97.48	93.07	99.39	95.23
433	49.06	49.06	50.00	98.12	49.53

434	49.07	49.07	50.00	98.15	49.53
435	48.71	48.71	50.00	97.42	49.35
436	48.68	48.71	49.97	97.36	49.33
437	53.78	95.22	55.07	97.49	69.78
438	48.62	48.62	50.00	97.24	49.30
439	48.28	48.28	50.00	96.57	49.13
440	50.22	98.29	51.93	96.59	67.96
441	49.25	49.25	50.00	98.50	49.62
442	48.30	48.30	50.00	96.59	49.13
443	90.18	93.80	95.43	99.18	94.61
444	48.86	48.86	50.00	97.71	49.42
445	59.06	96.76	61.39	95.46	75.12
446	70.62	97.71	71.94	97.82	82.87
447	48.76	48.76	50.00	97.52	49.37
448	48.54	48.54	50.00	97.08	49.26
449	49.27	49.27	50.00	98.55	49.63
450	49.36	49.36	50.00	98.71	49.68
451	49.28	49.28	50.00	98.56	49.64
452	48.58	48.58	50.00	97.17	49.28
453	47.86	47.86	50.00	95.73	48.91
454	61.63	98.53	62.71	97.91	76.64
455	83.92	91.53	89.63	98.68	90.57
456	52.26	97.03	55.23	94.10	70.39
457	54.94	96.46	57.77	94.42	72.26
458	48.66	48.66	50.00	97.33	49.32
459	57.29	98.90	58.38	97.82	73.42
460	48.57	48.73	49.84	97.15	49.28
461	48.70	48.70	50.00	97.41	49.34
462	49.19	49.19	50.00	98.39	49.59
463	47.90	47.90	50.00	95.81	48.93
464	59.23	65.23	70.11	96.10	67.58
465	49.34	49.37	49.97	98.69	49.67
466	66.28	99.12	66.95	98.71	79.92
467	54.40	64.92	59.80	93.37	62.26
468	48.43	55.39	50.69	95.18	52.93
469	48.73	48.73	50.00	97.46	49.36
470	48.88	48.88	50.00	97.76	49.43
471	48.74	48.74	50.00	97.49	49.36
472	47.59	47.75	49.83	95.18	48.77
473	60.08	96.48	61.54	97.29	75.15
474	49.24	49.25	49.99	98.48	49.62
475	67.84	98.12	69.15	97.58	81.13
476	47.72	47.72	50.00	95.45	48.84
477	75.46	85.78	82.32	98.82	84.02
478	49.13	49.13	50.00	98.25	49.56
479	49.31	49.31	50.00	98.61	49.65
480	49.63	83.18	50.91	97.45	63.16
481	49.24	49.24	50.00	98.47	49.62
482	49.12	96.18	50.98	96.27	66.64
483	47.12	47.12	50.00	94.24	48.52
484	65.93	73.24	76.65	97.92	74.91
485	48.27	48.35	49.93	96.55	49.12
486	47.45	47.45	50.00	94.90	48.69
487	48.94	48.95	49.99	97.88	49.46
488	49.03	49.03	50.00	98.06	49.51
489	48.03	48.49	49.51	96.06	48.99
490	48.22	48.49	49.71	96.43	49.09
491	49.20	49.20	50.00	98.40	49.60
492	48.68	48.68	50.00	97.37	49.33
493	47.81	49.38	48.40	95.62	48.88
494	48.25	48.44	49.80	96.51	49.11
495	47.91	47.91	50.00	95.81	48.93

496	52.03	98.18	53.85	96.36	69.55
497	79.91	87.48	87.81	98.48	87.64
498	49.06	49.06	50.00	98.12	49.53
499	48.65	48.65	50.00	97.29	49.31
500	48.60	48.60	50.00	97.20	49.29
501	48.90	48.90	50.00	97.79	49.44
502	50.80	84.21	53.57	94.51	65.49
503	55.18	96.82	57.38	95.65	72.06
504	67.74	94.06	69.03	99.08	79.62
505	54.08	98.93	55.15	97.86	70.82
506	48.77	48.77	50.00	97.54	49.38
507	49.66	98.35	51.31	96.70	67.43
508	54.69	98.13	56.56	96.28	71.76
509	54.70	98.49	56.21	96.99	71.58
510	48.63	48.63	50.00	97.27	49.31
511	73.07	96.92	74.53	98.09	84.27
512	91.43	97.15	93.69	99.22	95.39
513	68.93	97.89	70.69	96.69	82.09
514	50.52	77.73	51.39	98.28	61.87
515	48.72	48.72	50.00	97.43	49.35
516	53.85	88.90	55.08	97.74	68.01
517	48.48	48.55	49.92	96.96	49.23
518	47.88	47.88	50.00	95.77	48.92
519	49.18	49.18	50.00	98.37	49.59
520	48.98	48.98	50.00	97.97	49.49
521	47.54	47.54	50.00	95.08	48.74
522	47.28	47.28	50.00	94.56	48.60
523	48.11	48.11	50.00	96.23	49.04
524	49.23	49.23	50.00	98.45	49.61
525	48.43	49.44	49.55	96.47	49.49
526	56.08	86.72	58.15	96.45	69.62
527	48.29	48.29	50.00	96.58	49.13
528	49.38	49.38	50.00	98.76	49.69
529	49.23	49.23	50.00	98.47	49.61
530	48.98	48.98	50.00	97.96	49.49
531	48.93	48.93	50.00	97.86	49.46
532	46.23	95.49	50.74	90.99	66.27
533	50.21	97.53	52.68	95.08	68.41
534	62.32	97.65	64.24	96.27	77.50
535	49.28	49.28	50.00	98.56	49.64
536	74.95	86.82	81.02	98.09	83.82
537	48.84	48.84	50.00	97.68	49.41
538	58.88	98.41	60.47	96.84	74.91
539	68.47	95.25	70.16	97.80	80.80
540	49.38	49.38	50.00	98.75	49.69
541	54.50	86.62	56.20	96.94	68.17
542	48.95	48.95	50.00	97.90	49.47
543	48.89	95.03	53.66	90.53	68.59
544	48.98	48.98	50.00	97.95	49.48
545	48.84	48.84	50.00	97.68	49.41
546	48.84	48.84	50.00	97.68	49.41
547	49.28	49.28	50.00	98.56	49.64
548	67.54	97.84	68.45	98.58	80.55
549	52.38	98.16	54.22	96.33	69.85
550	74.87	97.65	76.22	98.03	85.62
551	64.45	84.67	67.57	97.87	75.16
552	57.04	98.86	58.18	97.74	73.25
553	48.68	48.68	50.00	97.36	49.33
554	71.33	91.35	75.36	95.06	82.59
555	48.99	48.99	50.00	97.98	49.49
556	48.68	48.68	50.00	97.36	49.33
557	48.95	48.95	50.00	97.89	49.47

558	48.95	48.95	50.00	97.89	49.47
559	87.65	96.68	90.09	98.19	93.27
560	81.84	98.24	83.29	97.65	90.15
561	86.02	98.48	87.19	98.49	92.49
562	59.29	97.24	62.01	94.65	75.73
563	63.49	97.50	65.93	95.24	78.67
564	78.28	98.51	79.74	97.21	88.14
565	77.46	98.03	79.39	96.40	87.73
566	61.08	96.25	64.80	92.78	77.46
567	54.60	95.89	58.67	92.02	72.80
568	86.83	97.31	88.84	97.99	92.88
569	60.98	86.30	63.22	97.45	72.98
570	48.73	96.97	51.75	93.96	67.49
571	81.73	98.09	83.28	97.53	90.08
572	83.41	97.89	85.03	97.74	91.01
573	88.36	99.20	89.07	98.91	93.86
574	86.46	96.69	88.55	99.45	92.44
575	49.28	49.28	50.00	98.55	49.64
576	65.79	93.07	67.75	97.51	78.42
577	73.94	80.13	85.91	98.17	82.92
578	54.51	98.29	56.23	96.59	71.53
579	48.09	48.09	50.00	96.17	49.02
580	56.99	79.92	58.33	98.83	67.44
581	48.21	97.61	50.60	95.22	66.65
582	61.39	98.13	63.15	96.55	76.84
583	49.12	49.12	50.00	98.23	49.55
584	48.86	48.86	50.00	97.73	49.43
585	78.04	99.42	78.62	98.86	87.80
586	57.06	94.82	62.22	90.01	75.13
587	70.33	94.34	74.43	92.99	83.21
588	74.37	98.03	75.89	97.33	85.55
589	72.08	97.52	74.42	95.57	84.42
590	79.33	98.52	80.64	97.65	88.69
591	49.05	49.05	50.00	98.09	49.52
592	69.25	98.47	70.71	97.15	82.32
593	71.44	96.73	74.12	95.13	83.93
594	44.18	44.18	50.00	88.37	46.91
595	75.18	97.32	77.71	95.33	86.42
596	88.54	97.05	90.80	98.15	93.82
597	88.91	97.86	90.63	98.21	94.10
598	74.98	97.77	76.99	96.31	86.14
599	46.12	46.12	50.00	92.23	47.98
600	84.71	98.84	85.81	98.04	91.86
601	93.71	99.05	94.54	99.29	96.74
602	64.18	85.32	67.22	97.56	75.20
603	49.40	49.40	50.00	98.80	49.70
604	49.71	51.93	50.45	98.45	51.18
605	49.38	49.38	50.00	98.76	49.69
606	47.45	47.62	49.81	94.90	48.69
607	72.94	96.85	75.38	95.74	84.77
608	58.14	95.49	60.84	94.80	74.32
609	55.65	94.77	57.84	95.78	71.83
610	54.46	80.99	58.62	92.39	68.02
611	67.06	97.01	69.78	94.80	81.17
612	56.07	86.66	59.02	94.70	70.22
613	75.67	98.92	76.46	98.67	86.25
614	78.58	97.72	80.43	96.90	88.24
615	83.61	97.09	86.03	96.35	91.23
616	78.69	96.20	81.79	94.97	88.41
617	57.79	95.34	61.38	93.03	74.68
618	47.91	47.91	50.00	95.82	48.93
619	49.26	49.26	50.00	98.52	49.63

620	48.86	51.65	56.29	94.09	53.87
621	49.00	49.11	49.89	98.01	49.50
622	50.47	99.14	51.33	98.27	67.64
623	49.29	49.29	50.00	98.57	49.64
624	67.33	82.47	73.44	95.22	77.70
625	49.07	49.07	50.00	98.13	49.53
626	50.30	98.84	51.46	97.67	67.68
627	49.31	98.88	50.44	97.75	66.80
628	48.51	48.51	50.00	97.03	49.25
629	53.42	97.91	55.50	95.84	70.85
630	49.14	49.15	49.99	98.29	49.57
631	49.30	49.30	50.00	98.60	49.65
632	53.37	95.71	56.94	92.95	71.41
633	62.20	94.74	65.88	93.12	77.71
634	60.95	86.22	64.39	95.00	73.72
635	74.23	89.00	79.64	95.39	84.06
636	47.09	47.09	50.00	94.18	48.50
637	73.59	93.16	76.69	96.74	84.13
638	72.91	96.91	75.12	96.23	84.64
639	71.46	93.24	74.46	96.32	82.80
640	55.89	98.27	57.32	97.17	72.40
641	64.47	80.52	68.37	98.74	73.95
642	49.39	49.39	50.00	98.79	49.69
643	49.32	49.32	50.00	98.65	49.66
644	48.57	48.57	50.00	97.14	49.28
645	49.04	49.04	50.00	98.09	49.52
646	47.26	47.26	50.00	94.51	48.59
647	48.97	48.97	50.00	97.93	49.48
648	48.97	48.97	50.00	97.94	49.48
649	63.10	95.46	64.10	98.61	76.70
650	49.40	49.40	50.00	98.81	49.70
651	48.62	48.62	50.00	97.25	49.30
652	48.35	48.75	49.58	96.71	49.16
653	82.51	95.85	84.89	98.60	90.04
654	54.66	96.56	57.69	94.01	72.23
655	48.12	48.12	50.00	96.24	49.04
656	48.84	48.84	50.00	97.69	49.41
657	49.35	99.10	50.25	98.20	66.69
658	69.70	89.89	73.05	97.13	80.60
659	51.99	96.25	53.86	96.28	69.07
660	51.89	99.08	52.81	98.16	68.90
661	50.34	98.87	51.47	97.74	67.70
662	64.92	79.08	69.93	98.13	74.23
663	88.99	94.12	93.62	99.11	93.87
664	79.05	98.56	79.98	98.70	88.30
665	49.09	49.09	50.00	98.19	49.54
666	48.99	48.99	50.00	97.99	49.49
667	49.91	98.35	51.57	96.70	67.66
668	49.24	49.24	50.00	98.49	49.62
669	54.19	95.45	56.45	95.56	70.94
670	50.19	92.94	51.51	97.36	66.29
671	48.94	98.57	50.37	97.15	66.67
672	78.30	97.00	80.46	96.70	87.96
673	77.20	97.04	78.97	97.66	87.07
674	48.50	48.50	50.00	96.99	49.24
675	61.65	87.47	64.77	95.56	74.43
676	47.51	47.51	50.00	95.02	48.72
677	49.21	49.21	50.00	98.42	49.60
678	67.12	98.47	68.53	97.26	80.81
679	48.85	48.85	50.00	97.71	49.42
680	60.25	99.00	61.09	98.33	75.56
681	79.02	93.76	82.12	98.34	87.55

682	73.80	99.04	74.65	98.39	85.13
683	54.96	63.78	58.61	97.27	61.09
684	49.14	49.29	49.84	98.28	49.57
685	48.21	48.21	50.00	96.42	49.09
686	65.02	87.23	67.84	97.62	76.32
687	49.17	49.17	50.00	98.34	49.58
688	49.23	49.23	50.00	98.45	49.61
689	49.23	49.23	50.00	98.45	49.61
690	75.82	94.76	78.19	97.93	85.68
691	59.62	97.44	62.14	95.05	75.89
692	52.18	96.38	55.71	92.99	70.61
693	48.79	49.08	49.70	97.58	49.39
694	52.35	93.85	53.89	96.98	68.46
695	47.48	47.48	50.00	94.95	48.71
696	48.31	48.31	50.00	96.61	49.14
697	49.43	49.43	50.00	98.86	49.71
698	48.90	48.90	50.00	97.81	49.45
699	76.86	89.96	81.49	98.58	85.51
700	61.25	94.76	62.76	97.48	75.51
701	48.77	48.77	50.00	97.54	49.38
702	49.40	49.40	50.00	98.79	49.70
703	49.37	49.37	50.00	98.74	49.68
704	48.80	49.13	49.65	97.60	49.39
705	49.14	49.14	50.00	98.28	49.57
706	61.85	98.04	63.80	96.15	77.30
707	57.83	97.36	60.47	94.79	74.60
708	47.58	47.58	50.00	95.16	48.76
709	61.69	96.60	63.86	95.87	76.89
710	48.96	48.96	50.00	97.92	49.47
711	48.97	48.97	50.00	97.94	49.48
712	49.49	56.58	50.33	98.28	53.28
713	77.14	87.52	83.63	98.34	85.53
714	49.26	49.26	50.00	98.52	49.63
715	48.82	48.82	50.00	97.65	49.41
716	48.82	48.82	50.00	97.63	49.40
717	58.73	93.79	59.65	98.57	72.92
718	62.84	92.17	64.40	98.06	75.82
719	48.66	48.68	49.98	97.31	49.32
720	81.88	92.58	86.20	98.57	89.28
721	83.35	93.64	87.11	99.21	90.26
722	84.44	99.30	84.88	99.60	91.52
723	66.23	99.05	67.18	98.12	80.06
724	48.41	48.41	50.00	96.82	49.19
725	63.00	96.65	64.12	98.14	77.10
726	48.99	49.03	49.96	97.97	49.49
727	73.15	78.59	86.45	98.27	82.33
728	49.07	49.07	50.00	98.14	49.53
729	49.27	49.29	49.98	98.54	49.63
730	49.17	98.38	50.79	96.76	66.99
731	60.24	97.86	62.32	95.88	76.15
732	47.15	47.15	50.00	94.31	48.54
733	48.24	48.24	50.00	96.48	49.10
734	49.31	49.31	50.00	98.63	49.65
735	71.42	95.39	73.12	98.18	82.79
736	48.55	48.55	50.00	97.11	49.27
737	48.46	48.46	50.00	96.93	49.22
738	61.04	93.82	62.95	96.76	75.35
739	75.46	97.27	76.97	97.96	85.94
740	53.62	98.85	54.76	97.71	70.48
741	49.22	49.22	50.00	98.45	49.61
742	63.10	93.47	65.12	96.84	76.76
743	78.06	91.26	82.57	97.52	86.70

744	81.89	90.46	87.82	98.73	89.12
745	77.13	80.31	92.90	98.39	86.14
746	68.01	97.90	69.33	97.66	81.17
747	72.79	74.24	97.62	97.68	84.34
748	88.07	95.57	91.20	99.46	93.33
749	61.59	68.71	70.81	97.80	69.74
750	53.22	96.62	54.04	98.40	69.31
751	85.14	92.62	90.15	99.21	91.37
752	76.67	83.75	86.48	98.43	85.09
753	59.81	89.72	61.59	97.44	73.04
754	51.22	85.28	52.39	97.70	64.91
755	67.60	97.98	68.85	97.76	80.87
756	79.09	98.09	80.48	97.86	88.42
757	49.31	49.31	50.00	98.62	49.65
758	89.45	99.12	90.15	99.21	94.43
759	81.02	97.68	82.48	98.32	89.44
760	70.27	83.89	76.47	96.65	80.01
761	65.38	98.12	66.61	97.72	79.35
762	48.99	48.99	50.00	97.98	49.49
763	67.27	98.44	68.08	98.61	80.49
764	49.40	99.07	50.33	98.15	66.75
765	49.29	49.29	50.00	98.59	49.64
766	49.36	49.36	50.00	98.73	49.68
767	49.43	49.43	50.00	98.86	49.71
768	48.68	48.68	50.00	97.35	49.33
769	48.95	48.95	50.00	97.91	49.47
770	59.74	83.43	62.30	96.98	71.33
771	48.99	48.99	50.00	97.98	49.49
772	48.29	48.29	50.00	96.59	49.13
773	59.56	93.05	61.69	96.26	74.19
774	48.70	48.70	50.00	97.39	49.34
775	49.35	49.35	50.00	98.70	49.67
776	48.99	48.99	50.00	97.99	49.49
777	53.90	98.04	55.70	96.42	71.04
778	47.33	47.33	50.00	94.65	48.63
779	54.59	77.79	55.73	98.53	64.94
780	68.02	84.09	73.03	96.87	78.17
781	69.90	93.53	71.84	98.33	81.26
782	78.02	98.29	79.07	98.57	87.64
783	61.76	98.89	62.87	97.80	76.87
784	49.07	49.11	49.97	98.15	49.53
785	80.62	97.45	82.12	98.46	89.13
786	80.53	99.08	81.38	98.44	89.36
787	94.64	98.43	95.99	99.66	97.19
788	83.27	86.45	94.63	98.73	90.35
789	74.99	97.56	76.51	97.67	85.77
790	49.20	49.20	50.00	98.41	49.60
791	78.64	90.88	83.46	97.76	87.01
792	84.43	88.50	93.63	98.83	91.00
793	55.47	97.29	56.52	97.95	71.51
794	68.02	93.90	69.70	98.30	80.01
795	65.89	95.62	67.20	98.20	78.93
796	69.05	80.15	76.12	98.53	78.08
797	48.75	48.84	49.91	97.49	49.37
798	49.22	49.22	50.00	98.45	49.61
799	49.20	49.20	50.00	98.39	49.60
800	49.29	49.29	50.00	98.58	49.64
801	49.67	98.93	50.74	97.86	67.07
802	86.19	94.44	90.05	98.48	92.19
803	72.49	94.30	75.37	96.12	83.78
804	64.61	97.49	67.06	95.22	79.46
805	77.22	96.99	78.99	97.68	87.07

806	48.91	48.91	50.00	97.82	49.45
807	49.12	49.46	49.83	98.15	49.65
808	49.42	49.42	50.00	98.85	49.71
809	49.08	49.08	50.00	98.16	49.53
810	78.96	97.51	80.28	98.70	88.06
811	73.43	97.88	75.14	96.90	85.02
812	47.59	47.59	50.00	95.17	48.76
813	76.06	97.51	78.35	95.81	86.89
814	84.77	97.68	86.50	97.86	91.75
815	48.49	48.49	50.00	96.97	49.23
816	46.97	46.97	50.00	93.93	48.44
817	50.32	97.39	52.93	94.80	68.58
818	68.10	98.07	69.34	97.78	81.24
819	55.85	97.90	57.95	95.83	72.81
820	47.49	47.49	50.00	94.98	48.71
821	48.09	48.20	49.88	96.18	49.03
822	48.76	48.76	50.00	97.53	49.37
823	48.26	48.59	49.65	96.52	49.12
824	46.92	46.92	50.00	93.83	48.41
825	47.48	97.32	50.16	94.65	66.20
826	64.89	93.14	67.50	95.92	78.28
827	48.40	48.40	50.00	96.81	49.19
828	49.40	49.40	50.00	98.80	49.70
829	48.86	48.86	50.00	97.71	49.42
830	64.62	98.44	66.18	96.92	79.15
831	69.97	85.28	75.13	97.15	79.88
832	75.97	90.95	79.73	98.76	84.97
833	49.29	49.29	50.00	98.59	49.64
834	48.04	49.04	48.96	96.08	49.00
835	48.00	48.00	50.00	96.00	48.98
836	48.99	48.99	50.00	97.97	49.49
837	49.29	49.29	50.00	98.58	49.64
838	49.39	49.39	50.00	98.78	49.69
839	49.17	49.19	49.98	98.33	49.58
840	49.16	49.16	50.00	98.31	49.57
841	49.30	49.30	50.00	98.59	49.65
842	49.15	49.15	50.00	98.30	49.57
843	49.15	49.15	50.00	98.30	49.57
844	59.16	98.52	60.41	97.53	74.90
845	48.67	96.47	50.59	96.16	66.37
846	48.35	48.50	49.84	96.69	49.16
847	48.71	48.71	50.00	97.42	49.35
848	48.23	48.23	50.00	96.45	49.10
849	48.98	48.98	50.00	97.97	49.49
850	64.85	95.62	65.99	98.46	78.09
851	72.46	94.14	75.40	96.10	83.73
852	60.12	95.70	63.20	94.09	76.13
853	68.58	95.78	70.64	96.75	81.31
854	87.89	96.91	90.08	98.82	93.37
855	48.47	97.34	51.14	94.68	67.05
856	85.38	97.49	87.22	97.94	92.07
857	49.52	99.22	50.29	98.45	66.75
858	72.93	96.78	75.44	95.61	84.79
859	60.89	95.07	63.97	94.20	76.48
860	59.90	91.75	62.82	94.86	74.58
861	64.00	90.72	67.20	95.18	77.21
862	57.82	96.91	60.67	94.40	74.62
863	50.15	78.12	53.00	94.30	63.16
864	60.53	97.17	62.18	96.85	75.83
865	49.18	49.18	50.00	98.36	49.59
866	54.58	97.10	55.49	98.22	70.62
867	62.67	96.26	65.42	94.78	77.90

868	72.44	97.24	74.46	96.48	84.34
869	55.57	86.65	59.70	92.30	70.70
870	64.10	92.23	66.76	95.93	77.46
871	48.91	48.91	50.00	97.82	49.45
872	61.91	93.45	63.42	97.74	75.56
873	48.13	48.13	50.00	96.26	49.05
874	48.72	48.99	49.71	97.44	49.35
875	49.22	49.22	50.00	98.44	49.61
876	49.29	49.29	50.00	98.58	49.64
877	49.28	49.28	50.00	98.56	49.64
878	70.86	98.99	71.87	98.01	83.28
879	49.00	49.33	49.66	98.00	49.49
880	82.09	98.38	83.60	97.30	90.39
881	45.36	45.36	50.00	90.73	47.57
882	61.37	97.76	62.94	96.96	76.58
883	60.55	77.17	63.67	98.41	69.77
884	64.23	96.35	65.21	98.57	77.78
885	48.43	48.43	50.00	96.87	49.20
886	84.27	98.02	85.66	98.42	91.42
887	54.14	96.07	57.42	93.54	71.88
888	74.20	94.17	77.21	96.28	84.85
889	81.25	96.87	83.32	97.56	89.58
890	46.21	49.06	48.04	91.85	48.54
891	85.24	95.08	88.43	98.68	91.63
892	51.25	98.91	52.34	97.82	68.46
893	56.57	96.69	58.76	95.70	73.10
894	53.24	96.13	57.11	92.36	71.65
895	66.41	89.74	70.49	94.25	78.96
896	46.53	95.44	51.09	90.89	66.55
897	69.56	96.19	72.65	94.36	82.78
898	56.24	82.02	59.66	94.25	69.08
899	54.63	96.60	56.91	95.50	71.62
900	53.86	94.81	56.58	94.64	70.87
901	49.24	49.72	49.91	98.29	49.82
902	51.45	97.56	53.89	95.15	69.43
903	74.99	98.48	76.17	97.92	85.90
904	57.10	97.93	58.88	96.49	73.54
905	54.59	93.90	56.23	96.86	70.34
906	48.08	52.29	50.79	93.77	51.53
907	48.29	48.34	49.95	96.57	49.13
908	49.12	98.46	50.66	96.92	66.90
909	55.01	79.70	57.94	95.06	67.10
910	48.61	48.61	50.00	97.21	49.29
911	71.78	91.18	74.79	97.98	82.18
912	54.72	97.68	56.46	96.55	71.56
913	61.95	97.35	63.31	97.48	76.72
914	65.90	98.84	67.06	97.71	79.91
915	88.05	98.22	89.50	98.20	93.66
916	74.83	95.05	77.85	95.69	85.59
917	58.77	97.18	61.24	95.17	75.13
918	49.22	98.72	50.50	97.44	66.82
919	80.12	94.72	82.88	98.38	88.40
920	81.20	98.08	82.76	97.46	89.77
921	68.82	98.54	70.28	97.13	82.05
922	73.87	98.01	74.97	98.40	84.96
923	65.67	96.67	67.54	96.68	79.52
924	77.19	97.42	78.66	98.14	87.04
925	79.58	98.14	80.53	99.15	88.47
926	62.21	98.61	63.33	97.81	77.13
927	77.87	97.32	79.63	97.49	87.59
928	52.94	97.21	55.73	94.46	70.85
929	68.02	95.43	70.39	96.13	81.02

930	70.63	96.21	73.08	95.86	83.07
931	68.76	96.29	71.76	94.50	82.23
932	53.67	95.39	57.96	91.58	72.10
933	46.85	46.85	50.00	93.71	48.38
934	48.67	48.67	50.00	97.35	49.33
935	60.60	97.65	62.86	95.56	76.48
936	50.93	95.58	52.38	97.10	67.67
937	46.71	95.83	50.88	91.66	66.47
938	52.55	94.62	57.44	90.37	71.49
939	81.42	97.06	83.48	97.29	89.76
940	85.12	97.72	86.72	98.34	91.89
941	60.59	97.59	62.26	96.79	76.02
942	55.05	94.87	57.12	95.97	71.31
943	56.69	94.99	59.06	95.43	72.84
944	49.58	63.98	50.99	97.13	56.76
945	49.26	49.26	50.00	98.51	49.63
946	62.93	79.67	66.67	98.10	72.59
947	61.52	66.90	72.89	98.26	69.77
948	48.63	48.65	49.98	97.26	49.31
949	48.57	48.57	50.00	97.14	49.27
950	48.27	48.27	50.00	96.53	49.12
951	53.43	98.43	55.00	96.88	70.57
952	49.10	49.10	50.00	98.21	49.55
953	62.26	98.37	63.49	97.62	77.17
954	52.77	95.25	55.21	95.18	69.90
955	51.87	91.10	54.11	95.58	67.89
956	49.10	49.10	50.00	98.19	49.54
957	82.39	98.60	83.26	99.11	90.28
958	83.21	98.97	84.01	98.78	90.88
959	80.48	97.72	82.23	97.31	89.30
960	75.29	98.55	76.41	98.02	86.08
961	48.70	48.70	50.00	97.41	49.34
962	61.46	96.10	64.69	93.80	77.33
963	64.15	95.02	66.99	94.89	78.58
964	49.25	49.25	50.00	98.50	49.62
965	63.55	99.31	64.24	98.62	78.01
966	63.35	97.28	65.70	95.45	78.43
967	67.21	89.55	70.85	95.59	79.11
968	83.24	87.03	93.66	98.88	90.22
969	77.71	97.48	79.19	98.11	87.38
970	78.38	89.22	84.22	97.91	86.64
971	61.48	79.63	67.09	93.12	72.82
972	52.48	96.10	56.08	92.88	70.83
973	74.55	97.86	75.71	98.41	85.37
974	79.89	98.55	80.99	98.23	88.91
975	72.48	97.09	74.50	96.55	84.31
976	83.02	95.90	85.69	97.55	90.51
977	66.70	98.27	68.28	96.93	80.58
978	49.11	49.11	50.00	98.22	49.55
979	47.25	50.66	52.26	92.09	51.45
980	59.96	94.67	61.33	97.67	74.44
981	49.39	49.39	50.00	98.77	49.69
982	48.91	98.63	50.28	97.27	66.60
983	63.22	98.55	64.60	97.27	78.05
984	48.86	49.16	49.70	97.73	49.43
985	49.25	49.30	49.95	98.50	49.62
986	48.20	48.20	50.00	96.40	49.08
987	54.81	96.89	57.79	94.12	72.39
988	60.88	96.97	62.95	96.02	76.34
989	48.69	50.29	50.10	96.59	50.20
990	60.06	70.69	65.47	97.87	67.98
991	68.10	80.07	75.71	95.95	77.83

992	73.12	97.41	74.80	97.26	84.62
993	49.19	49.19	50.00	98.37	49.59
994	80.16	93.47	83.44	98.97	88.17
995	49.31	49.31	50.00	98.61	49.65
996	48.96	48.96	50.00	97.93	49.48
997	50.78	95.48	53.48	94.63	68.56
998	56.55	98.32	58.11	96.90	73.05
999	49.80	99.03	50.77	98.05	67.13
1000	52.49	97.03	54.56	95.89	69.84
1001	48.32	48.32	50.00	96.64	49.14
1002	62.38	78.28	67.98	94.72	72.77
1003	67.32	86.41	71.53	96.22	78.27
1004	69.03	90.95	71.40	98.50	80.00
1005	52.71	95.88	53.88	97.69	68.99
1006	55.44	97.77	57.67	95.58	72.55
1007	63.07	97.24	64.61	97.17	77.63
1008	49.70	99.39	50.31	98.77	66.80
1009	55.96	67.82	59.45	97.01	63.36
1010	53.80	89.91	55.87	96.05	68.92
1011	53.15	70.10	54.69	97.76	61.45
1012	48.63	48.63	50.00	97.27	49.31
1013	49.38	49.38	50.00	98.75	49.69
1014	49.38	49.38	50.00	98.75	49.69
1015	48.47	48.47	50.00	96.95	49.23
1016	68.13	97.65	69.61	97.37	81.28
1017	49.92	98.08	51.84	96.17	67.83
1018	63.86	96.79	65.51	97.04	78.14
1019	52.89	85.94	54.68	96.62	66.83
1020	64.87	92.91	67.07	96.86	77.90
1021	61.49	98.14	62.91	97.25	76.67
1022	69.76	98.30	71.22	97.25	82.60
1023	48.82	48.82	50.00	97.63	49.40
1024	66.85	92.34	70.10	95.15	79.69
1025	67.03	95.20	69.07	96.82	80.06
1026	83.34	93.15	87.47	99.12	90.22
1027	49.31	49.31	50.00	98.63	49.65
1028	53.76	98.63	55.13	97.27	70.73
1029	49.57	97.92	51.65	95.85	67.63
1030	48.86	48.86	50.00	97.72	49.42
1031	48.60	48.60	50.00	97.20	49.29
1032	61.87	97.38	64.38	95.09	77.52
1033	62.41	98.32	64.08	96.69	77.59
1034	56.57	98.64	57.93	97.29	73.00
1035	47.90	47.90	50.00	95.79	48.92
1036	70.90	91.33	74.32	96.45	81.95
1037	52.95	98.12	54.60	96.71	70.16
1038	67.06	91.23	69.50	97.46	78.90
1039	73.86	94.07	76.31	97.79	84.26
1040	48.24	48.44	49.79	96.48	49.10
1041	48.42	50.15	50.01	96.70	50.08
1042	68.64	97.95	69.73	98.17	81.46
1043	76.52	95.56	78.79	97.58	86.37
1044	74.17	83.56	82.29	98.40	82.92
1045	58.84	94.51	61.35	95.30	74.40
1046	76.06	92.10	79.81	97.16	85.52
1047	49.07	49.07	50.00	98.15	49.53
1048	61.61	76.76	65.49	98.25	70.68
1049	49.17	49.17	50.00	98.35	49.58
1050	48.89	48.89	50.00	97.77	49.44
1051	49.40	49.40	50.00	98.79	49.70
1052	52.70	92.10	54.71	96.05	68.64
1053	72.82	96.39	74.87	96.91	84.28

1054	60.43	94.17	62.27	96.80	74.97
1055	51.53	77.31	52.69	97.80	62.67
1056	49.00	49.00	50.00	98.00	49.50
1057	74.97	97.70	77.11	96.03	86.19
1058	57.41	97.45	59.51	95.86	73.90
1059	79.38	97.89	81.37	96.37	88.87
1060	89.45	98.55	90.83	97.70	94.53
1061	91.86	98.64	93.12	98.17	95.80
1062	93.67	99.11	94.52	98.72	96.76
1063	93.67	99.10	94.52	98.79	96.76
1064	93.35	99.05	94.22	98.86	96.58
1065	89.32	98.60	90.53	98.36	94.39
1066	95.12	99.10	95.96	98.98	97.50
1067	89.59	98.51	90.99	97.77	94.60
1068	48.96	48.96	50.00	97.92	49.47
1069	80.01	97.53	82.34	95.83	89.30
1070	81.33	94.67	84.91	96.07	89.52
1071	80.72	98.01	82.54	96.77	89.61
1072	79.65	97.85	81.58	96.60	88.97
1073	85.30	98.85	86.45	97.83	92.23
1074	86.57	98.61	87.84	97.90	92.91
1075	52.90	93.97	58.93	88.26	72.43
1076	75.71	97.00	78.60	94.71	86.83
1077	90.39	99.28	91.02	99.11	94.97
1078	85.67	97.33	87.54	98.30	92.17
1079	48.93	48.93	50.00	97.86	49.46
1080	66.53	94.94	70.01	93.75	80.59
1081	67.82	95.12	71.92	92.51	81.91
1082	54.22	94.02	58.75	91.15	72.31
1083	72.26	91.83	76.63	94.13	83.55
1084	72.67	89.89	77.89	93.84	83.46
1085	50.23	53.85	51.93	97.15	52.88
1086	51.42	94.33	52.19	98.46	67.20
1087	69.51	99.37	70.05	98.96	82.17
1088	90.39	98.15	91.79	99.25	94.87
1089	78.03	89.76	83.28	98.18	86.40
1090	85.34	95.22	88.48	98.48	91.73
1091	49.13	49.13	50.00	98.26	49.56
1092	63.24	74.44	69.47	98.02	71.87
1093	56.96	95.17	58.89	96.32	72.76
1094	47.88	47.88	50.00	95.75	48.92
1095	50.00	98.46	51.54	96.92	67.66
1096	48.17	49.50	49.86	95.83	49.68
1097	48.87	48.87	50.00	97.73	49.43
1098	75.20	98.93	75.92	98.82	85.91
1099	81.96	97.79	83.60	97.66	90.14
1100	81.17	93.94	85.05	96.31	89.28
1101	80.04	89.40	86.53	97.25	87.95
1102	78.79	98.05	80.74	96.32	88.56
1103	86.03	98.45	87.31	98.13	92.55
1104	76.46	84.63	85.94	96.10	85.28
1105	87.62	96.71	90.23	97.20	93.36
1106	91.63	97.53	93.78	97.76	95.62
1107	89.21	97.13	91.63	97.20	94.30
1108	93.35	98.24	94.91	98.51	96.55
1109	65.34	96.24	69.07	92.88	80.42
1110	91.30	98.96	92.33	98.17	95.53
1111	87.71	98.69	88.97	97.79	93.58
1112	48.94	48.94	50.00	97.87	49.46
1113	89.61	98.02	91.30	97.89	94.54
1114	87.00	98.55	88.43	97.41	93.22
1115	80.84	95.13	84.20	95.89	89.33

1116	75.61	97.22	78.34	94.93	86.77
1117	81.47	91.76	86.77	96.46	89.19
1118	83.90	93.99	87.94	97.21	90.86
1119	90.30	98.37	91.62	98.72	94.87
1120	91.20	98.70	92.27	98.94	95.38
1121	79.18	97.56	81.51	95.77	88.82
1122	79.70	97.74	81.90	95.97	89.12
1123	68.77	98.26	69.37	99.21	81.32
1124	92.78	99.05	93.58	99.33	96.24
1125	73.78	94.50	76.49	96.71	84.55
1126	67.68	96.14	71.28	93.24	81.86
1127	73.45	93.34	78.23	92.41	85.12
1128	65.61	95.34	70.08	91.61	80.78
1129	68.24	96.23	71.27	94.43	81.89
1130	81.83	91.20	87.49	97.15	89.31
1131	50.30	63.75	51.26	98.13	56.82
1132	49.35	49.35	50.00	98.71	49.68
1133	70.56	84.37	76.26	97.59	80.11
1134	79.03	98.05	80.14	98.73	88.20
1135	72.43	97.72	74.32	96.54	84.43
1136	67.97	96.25	70.42	95.67	81.33
1137	48.05	48.05	50.00	96.10	49.00
1138	48.42	48.46	49.95	96.83	49.20
1139	48.27	48.27	50.00	96.54	49.12
1140	63.08	87.42	64.96	98.70	74.53
1141	73.25	98.71	74.55	97.48	84.94
1142	85.81	98.44	87.19	97.76	92.48
1143	74.19	97.39	76.69	95.32	85.81
1144	74.21	98.20	76.00	96.54	85.69
1145	78.38	98.66	79.20	98.87	87.87
1146	67.90	96.24	69.72	97.05	80.86
1147	80.84	94.77	83.91	97.50	89.01
1148	49.71	95.71	52.56	94.32	67.86
1149	59.52	97.27	61.01	97.15	74.99
1150	59.42	90.82	60.57	98.51	72.67
1151	85.32	96.54	87.46	99.13	91.78
1152	59.17	96.89	60.88	96.73	74.78
1153	59.93	95.64	61.20	97.76	74.64
1154	62.45	98.60	63.78	97.38	77.46
1155	48.54	48.54	50.00	97.07	49.26
1156	56.56	63.80	62.10	97.50	62.94
1157	54.07	96.44	56.52	95.16	71.27
1158	49.10	49.10	50.00	98.19	49.54
1159	49.23	49.23	50.00	98.47	49.61
1160	48.98	48.98	50.00	97.96	49.48
1161	47.76	47.76	50.00	95.51	48.85
1162	48.67	98.56	50.11	97.13	66.44
1163	63.12	97.53	65.26	95.87	78.19
1164	67.60	95.80	70.94	93.86	81.52
1165	76.20	97.34	78.35	96.28	86.82
1166	75.35	95.93	77.96	96.11	86.02
1167	73.65	86.51	79.84	96.66	83.04
1168	54.53	88.05	56.18	96.99	68.59
1169	48.95	48.95	50.00	97.90	49.47
1170	49.08	49.11	49.97	98.16	49.54
1171	49.23	49.23	50.00	98.46	49.61
1172	47.19	48.73	48.38	94.37	48.55
1173	66.88	95.35	69.09	96.41	80.12
1174	72.95	94.68	75.43	96.99	83.97
1175	52.54	96.83	53.18	98.74	68.65
1176	49.03	49.03	50.00	98.07	49.51
1177	48.67	48.67	50.00	97.33	49.32

1178	48.16	48.16	50.00	96.32	49.06
1179	51.46	93.35	52.69	97.57	67.36
1180	48.46	48.46	50.00	96.92	49.22
1181	65.22	78.30	70.57	98.61	74.23
1182	48.44	48.44	50.00	96.89	49.21
1183	48.59	48.59	50.00	97.18	49.28
1184	60.73	98.73	61.28	98.97	75.62
1185	48.97	48.97	50.00	97.94	49.48
1186	49.18	49.18	50.00	98.35	49.58
1187	49.33	49.33	50.00	98.65	49.66
1188	71.75	94.47	73.49	98.73	82.67
1189	49.25	49.25	50.00	98.49	49.62
1190	52.81	98.01	54.59	96.46	70.12
1191	49.07	49.07	50.00	98.13	49.53
1192	48.46	48.46	50.00	96.91	49.22
1193	64.25	89.16	67.10	96.50	76.57
1194	47.90	47.90	50.00	95.80	48.93
1195	47.74	47.74	50.00	95.48	48.84
1196	48.79	75.92	53.75	89.98	62.94
1197	71.53	94.88	74.78	94.74	83.64
1198	49.17	49.17	50.00	98.35	49.58
1199	54.65	78.14	56.43	97.30	65.53
1200	51.77	91.60	54.83	93.97	68.59
1201	59.50	93.68	62.62	94.20	75.06
1202	51.27	95.68	53.30	95.96	68.46
1203	55.90	99.05	56.85	98.12	72.24
1204	48.75	48.75	50.00	97.50	49.37
1205	48.84	48.84	50.00	97.68	49.41
1206	48.84	48.84	50.00	97.68	49.41
1207	51.44	87.40	52.45	98.02	65.55
1208	48.08	48.08	50.00	96.15	49.02
1209	62.36	70.04	73.58	95.08	71.77
1210	53.89	75.06	56.88	94.94	64.72
1211	48.39	48.39	50.00	96.77	49.18
1212	62.28	91.27	64.95	95.80	75.89
1213	49.37	49.37	50.00	98.74	49.68
1214	48.37	48.37	50.00	96.73	49.17
1215	50.62	90.24	52.79	95.68	66.61
1216	48.94	48.94	50.00	97.89	49.47
1217	49.30	49.30	50.00	98.60	49.65
1218	48.49	48.49	50.00	96.98	49.23
1219	52.56	98.75	53.81	97.50	69.66
1220	48.97	48.97	50.00	97.93	49.48
1221	48.26	48.26	50.00	96.51	49.11
1222	47.55	47.55	50.00	95.10	48.75
1223	53.63	99.10	54.53	98.21	70.35
1224	46.22	46.22	50.00	92.45	48.04
1225	50.79	95.29	55.51	90.68	70.15
1226	68.20	95.34	71.20	94.78	81.52
1227	73.02	99.03	73.91	98.27	84.65
1228	48.27	48.27	50.00	96.55	49.12
1229	48.15	97.63	50.51	95.27	66.58
1230	49.38	49.38	50.00	98.75	49.69
1231	48.21	98.00	50.21	96.00	66.40
1232	48.10	48.10	50.00	96.20	49.03
1233	48.94	48.94	50.00	97.88	49.46
1234	72.64	82.62	80.76	97.73	81.68
1235	48.94	48.94	50.00	97.88	49.46
1236	58.42	86.49	60.31	97.35	71.06
1237	48.36	48.36	50.00	96.72	49.17
1238	54.29	96.72	56.63	95.37	71.43
1239	49.29	81.79	52.12	94.32	63.67

1240	60.06	99.35	60.71	98.70	75.37
1241	49.06	49.06	50.00	98.12	49.53
1242	48.36	48.36	50.00	96.72	49.17
1243	50.80	57.98	51.77	98.39	54.70
1244	48.84	49.45	49.69	97.51	49.57
1245	48.91	48.91	50.00	97.82	49.45
1246	57.38	92.70	58.86	97.41	72.00
1247	63.97	96.97	65.43	97.39	78.14
1248	60.93	97.97	61.77	98.47	75.76
1249	48.65	48.65	50.00	97.29	49.31
1250	55.40	80.49	58.27	95.21	67.60
1251	69.09	81.36	76.95	94.85	79.09
1252	48.33	65.51	52.41	91.55	58.23
1253	44.82	44.82	50.00	89.65	47.27
1254	72.93	97.05	75.44	95.44	84.89
1255	80.69	92.99	84.76	97.42	88.69
1256	64.62	83.32	67.58	99.04	74.63
1257	63.78	97.84	65.91	95.83	78.76
1258	86.38	98.93	87.35	98.36	92.78
1259	73.10	98.01	74.99	96.40	84.97
1260	50.96	96.31	52.59	96.75	68.03
1261	56.38	88.40	58.42	96.45	70.35
1262	49.29	49.29	50.00	98.59	49.64
1263	49.18	49.18	50.00	98.36	49.59
1264	49.68	51.13	50.36	98.53	50.75
1265	49.28	49.28	50.00	98.56	49.64
1266	48.56	48.56	50.00	97.13	49.27
1267	49.37	49.37	50.00	98.73	49.68
1268	47.99	47.99	50.00	95.97	48.97
1269	68.73	94.24	70.47	98.16	80.64
1270	48.48	95.84	50.85	95.25	66.45
1271	48.24	98.08	50.16	96.16	66.37
1272	61.87	93.52	63.05	98.43	75.32
1273	62.85	76.68	68.24	96.90	72.22
1274	57.53	99.24	58.29	98.48	73.44
1275	63.52	76.57	68.84	97.99	72.50
1276	59.34	92.46	61.34	96.57	73.75
1277	53.74	97.29	56.46	94.62	71.45
1278	48.38	48.38	50.00	96.75	49.17
1279	68.42	96.50	69.36	99.03	80.71
1280	49.25	49.25	50.00	98.50	49.62
1281	54.31	82.79	55.61	97.86	66.53
1282	56.31	93.77	58.67	95.51	72.18
1283	56.38	83.75	59.96	93.78	69.89
1284	54.98	90.50	58.23	93.78	70.87
1285	47.07	47.07	50.00	94.15	48.49
1286	50.64	95.33	55.31	90.76	70.00
1287	48.84	95.50	53.34	91.07	68.45
1288	52.72	97.19	55.29	94.90	70.48
1289	63.34	96.88	66.26	94.35	78.70
1290	63.60	67.97	83.31	95.33	74.86
1291	80.15	97.87	82.11	96.51	89.30
1292	77.34	97.70	79.43	96.20	87.63
1293	49.42	49.42	50.00	98.84	49.71
1294	48.96	49.13	49.82	97.92	49.47
1295	49.38	49.38	50.00	98.77	49.69
1296	49.06	49.06	50.00	98.12	49.53
1297	45.57	49.11	46.33	91.14	47.68
1298	48.61	48.61	50.00	97.22	49.30
1299	58.40	94.09	59.57	98.02	72.95
1300	59.78	89.45	61.22	98.16	72.69
1301	83.39	95.65	86.04	98.38	90.59

1302	47.67	47.67	50.00	95.34	48.81
1303	63.43	87.02	66.24	97.00	75.22
1304	70.15	98.45	71.21	98.10	82.64
1305	64.45	97.02	65.31	98.71	78.07
1306	49.16	49.16	50.00	98.33	49.58
1307	47.82	47.82	50.00	95.64	48.89
1308	48.59	48.59	50.00	97.19	49.29
1309	57.36	87.59	59.22	97.03	70.66
1310	70.38	93.09	73.45	95.97	82.11
1311	54.99	95.16	56.63	96.83	71.00
1312	85.10	90.63	92.20	98.36	91.41
1313	75.39	98.50	76.19	98.92	85.92
1314	74.31	89.01	79.18	97.07	83.81
1315	66.54	84.78	71.83	94.38	77.77
1316	63.99	94.02	67.53	93.65	78.60
1317	66.51	95.63	69.35	94.90	80.40
1318	52.19	97.82	54.37	95.65	69.89
1319	47.89	95.23	52.00	91.81	67.27
1320	47.55	94.53	52.40	90.34	67.42
1321	59.02	97.35	61.11	95.90	75.09
1322	70.79	96.00	73.03	96.47	82.95
1323	49.00	49.00	50.00	98.01	49.50
1324	61.71	96.30	64.69	94.29	77.39
1325	62.24	89.49	65.28	95.42	75.49
1326	55.95	99.25	56.69	98.51	72.17
1327	49.00	49.00	50.00	98.01	49.50
1328	78.09	94.05	80.77	98.75	86.91
1329	48.91	48.91	50.00	97.82	49.45
1330	52.92	99.46	53.46	98.91	69.54
1331	51.81	99.43	52.38	98.87	68.61
1332	49.17	49.17	50.00	98.33	49.58
1333	54.19	77.13	55.94	97.28	64.85
1334	50.51	65.74	52.54	96.06	58.40
1335	48.44	48.44	50.00	96.89	49.21
1336	48.31	48.31	50.00	96.62	49.14
1337	83.58	89.77	90.96	98.32	90.36
1338	49.12	49.12	50.00	98.25	49.56
1339	71.26	98.17	72.66	97.44	83.51
1340	68.79	97.11	70.81	96.37	81.90
1341	70.29	91.99	73.18	97.05	81.51
1342	58.77	76.94	63.66	93.68	69.67
1343	52.98	96.41	56.58	92.89	71.31
1344	75.69	97.67	77.77	96.23	86.59
1345	52.88	98.30	54.58	96.61	70.19
1346	69.81	97.40	72.17	95.53	82.91
1347	53.60	96.09	56.95	93.38	71.51
1348	54.87	74.48	59.74	91.85	66.30
1349	62.86	88.98	67.16	93.06	76.55
1350	68.36	93.21	71.49	95.34	80.92
1351	70.08	85.51	75.97	95.10	80.46
1352	45.93	45.93	50.00	91.85	47.88
1353	57.87	69.17	63.85	94.81	66.41
1354	49.41	49.41	50.00	98.82	49.70
1355	49.11	49.11	50.00	98.21	49.55
1356	49.40	49.40	50.00	98.81	49.70
1357	72.11	77.91	85.60	97.44	81.57
1358	55.23	61.96	61.20	96.14	61.57
1359	53.58	91.41	54.37	98.53	68.19
1360	49.24	49.24	50.00	98.47	49.62
1361	50.08	61.61	51.65	96.89	56.19
1362	70.76	94.75	72.61	98.06	82.22
1363	49.89	98.60	51.30	97.19	67.48

1364	60.77	94.34	61.62	98.86	74.55
1365	49.15	49.27	49.87	98.29	49.57
1366	53.18	96.38	55.54	95.32	70.47
1367	47.43	97.40	50.03	94.80	66.10
1368	61.13	98.41	62.57	97.16	76.50
1369	48.86	48.86	50.00	97.73	49.43
1370	72.44	98.40	73.98	97.04	84.46
1371	70.25	96.57	72.37	96.45	82.73
1372	47.26	47.26	50.00	94.52	48.59
1373	46.86	57.21	50.77	91.69	53.80
1374	68.21	78.42	78.18	94.00	78.30
1375	58.60	98.48	60.12	96.99	74.66
1376	54.46	80.45	56.83	95.93	66.61
1377	83.43	94.95	86.58	98.06	90.57
1378	63.12	95.96	65.94	94.70	78.16
1379	49.09	49.09	50.00	98.19	49.54
1380	53.01	97.10	55.81	94.44	70.88
1381	73.42	97.91	74.95	97.35	84.90
1382	70.85	91.25	74.00	97.16	81.72
1383	70.46	97.78	72.45	96.22	83.23
1384	62.90	78.20	68.73	94.89	73.16
1385	49.23	49.23	50.00	98.45	49.61
1386	48.78	48.78	50.00	97.56	49.38
1387	63.03	98.03	64.04	98.13	77.47
1388	48.23	98.19	50.04	96.37	66.30
1389	53.05	96.62	54.69	96.76	69.84
1390	72.81	94.98	75.08	97.27	83.87
1391	49.17	49.17	50.00	98.34	49.58
1392	48.90	48.90	50.00	97.79	49.44
1393	80.08	86.83	88.69	98.75	87.75
1394	49.02	49.02	50.00	98.04	49.51
1395	49.37	49.37	50.00	98.74	49.68
1396	49.35	49.35	50.00	98.70	49.67
1397	49.43	49.43	50.00	98.86	49.71
1398	54.59	98.77	55.82	97.54	71.33
1399	57.94	90.83	60.21	96.01	72.42
1400	48.42	48.42	50.00	96.83	49.19
1401	84.15	95.14	87.00	99.34	90.89
1402	49.09	49.09	50.00	98.18	49.54
1403	57.57	97.12	60.18	94.88	74.31
1404	73.35	97.49	75.08	97.09	84.83
1405	63.37	88.07	66.16	96.65	75.56
1406	75.18	85.22	83.16	96.42	84.18
1407	48.51	48.51	50.00	97.02	49.24
1408	49.33	49.33	50.00	98.65	49.66
1409	66.15	96.14	67.74	97.47	79.48
1410	48.38	48.38	50.00	96.75	49.18
1411	49.33	49.33	50.00	98.66	49.66
1412	49.43	98.60	50.83	97.20	67.08
1413	62.06	97.73	63.94	96.35	77.30
1414	62.45	93.46	65.21	95.20	76.82
1415	81.21	96.65	82.86	99.33	89.23
1416	69.84	94.69	71.35	98.73	81.38
1417	49.31	49.31	50.00	98.63	49.65
1418	49.24	49.24	50.00	98.47	49.62
1419	77.74	80.41	93.92	98.83	86.64
1420	55.90	97.82	58.08	95.67	72.89
1421	80.17	97.80	81.84	97.40	89.11
1422	72.55	94.87	74.81	97.32	83.66
1423	49.11	49.11	50.00	98.23	49.55
1424	54.49	98.75	55.74	97.52	71.26
1425	52.00	98.88	53.13	97.76	69.12

1426	68.60	96.47	69.60	98.92	80.86
1427	49.35	49.35	50.00	98.69	49.67
1428	49.37	49.37	50.00	98.73	49.68
1429	49.03	49.03	50.00	98.06	49.51
1430	49.57	92.27	51.16	96.80	65.83
1431	66.55	88.95	69.03	98.28	77.73
1432	52.25	98.68	53.57	97.36	69.44
1433	48.97	96.49	51.34	95.28	67.02
1434	58.72	96.89	59.70	98.21	73.88
1435	53.90	98.39	55.51	96.78	70.98
1436	69.30	89.78	72.05	98.48	79.94
1437	48.83	48.86	49.97	97.67	49.41
1438	48.06	48.14	49.91	96.12	49.01
1439	48.42	48.42	50.00	96.84	49.20
1440	55.99	96.62	57.40	97.27	72.02
1441	52.05	74.96	52.91	98.51	62.04
1442	49.38	52.06	50.41	97.75	51.22
1443	62.41	84.38	65.42	97.06	73.70
1444	48.44	48.44	50.00	96.89	49.21
1445	73.99	98.64	75.23	97.65	85.36
1446	84.42	94.03	88.26	98.53	91.05
1447	94.78	97.46	97.06	99.62	97.26
1448	79.44	83.51	92.24	98.18	87.66
1449	78.77	84.05	89.89	98.68	86.87
1450	48.88	48.88	50.00	97.76	49.43
1451	68.91	98.08	70.61	96.75	82.11
1452	83.68	97.07	85.41	98.93	90.87
1453	65.11	74.44	75.01	95.26	74.72
1454	49.13	49.13	50.00	98.27	49.56
1455	49.04	49.04	50.00	98.09	49.52
1456	48.95	48.95	50.00	97.90	49.47
1457	47.80	47.80	50.00	95.60	48.87
1458	77.15	83.82	87.23	98.52	85.49
1459	69.37	79.20	78.72	95.95	78.96
1460	50.42	94.75	51.50	97.83	66.73
1461	78.51	88.82	84.37	99.11	86.54
1462	71.45	81.49	80.12	96.67	80.80
1463	67.20	95.50	69.11	97.07	80.19
1464	55.30	94.17	58.71	93.35	72.33
1465	54.94	79.96	58.15	94.46	67.33
1466	64.22	98.73	65.42	97.63	78.70
1467	56.73	99.01	57.72	98.03	72.92
1468	74.11	99.11	74.70	99.00	85.19
1469	83.21	95.35	86.27	97.34	90.58
1470	85.52	97.53	87.60	96.99	92.30
1471	69.95	96.79	72.63	95.08	82.98
1472	62.38	97.93	63.75	97.40	77.22
1473	48.24	48.24	50.00	96.47	49.10
1474	56.25	94.66	57.23	98.22	71.33
1475	48.98	48.98	50.00	97.96	49.48
1476	55.99	98.99	56.52	98.97	71.95
1477	55.06	99.10	55.96	98.21	71.53
1478	49.36	49.36	50.00	98.71	49.68
1479	59.65	98.18	61.47	96.40	75.61
1480	55.88	96.44	59.34	93.18	73.48
1481	56.07	96.01	59.05	94.16	73.13
1482	56.60	97.11	59.31	94.66	73.64
1483	62.71	97.25	65.03	95.52	77.94
1484	89.52	97.09	91.72	98.75	94.33
1485	82.05	96.82	84.22	97.39	90.08
1486	57.72	94.95	61.67	92.35	74.77
1487	49.03	49.03	50.00	98.07	49.51

1488	48.96	48.96	50.00	97.92	49.47
1489	49.40	49.40	50.00	98.81	49.70
1490	49.40	49.40	50.00	98.81	49.70
1491	49.01	49.01	50.00	98.02	49.50
1492	85.43	99.35	86.06	98.79	92.23
1493	85.56	98.48	86.90	97.84	92.33
1494	81.77	97.18	83.45	98.46	89.79
1495	48.19	48.21	49.98	96.38	49.08
1496	65.08	95.87	66.54	97.73	78.55
1497	82.61	96.47	84.81	98.07	90.26
1498	54.85	94.92	57.45	94.92	71.58
1499	49.98	98.34	51.64	96.68	67.72
1500	49.31	49.31	50.00	98.63	49.65
1501	74.56	91.08	78.41	97.31	84.27
1502	48.16	48.16	50.00	96.32	49.06
1503	49.72	99.26	50.46	98.52	66.91
1504	49.28	49.28	50.00	98.56	49.64
1505	49.41	49.41	50.00	98.83	49.70
1506	47.65	47.65	50.00	95.30	48.80
1507	45.49	45.49	50.00	90.97	47.64
1508	81.13	97.41	83.17	96.84	89.73
1509	49.10	49.10	50.00	98.20	49.55
1510	48.28	48.28	50.00	96.55	49.12
1511	60.43	98.16	62.27	96.37	76.20
1512	51.84	97.38	54.08	95.55	69.54
1513	72.05	89.93	75.76	97.50	82.24
1514	49.35	49.35	50.00	98.69	49.67
1515	48.99	48.99	50.00	97.98	49.49
1516	76.68	96.54	78.39	98.23	86.52
1517	73.54	99.70	73.84	99.40	84.84
1518	49.09	49.09	50.00	98.19	49.54
1519	49.07	49.07	50.00	98.14	49.53
1520	49.10	49.10	50.00	98.19	49.54
1521	48.70	48.70	50.00	97.40	49.34
1522	46.60	46.60	50.00	93.19	48.24
1523	90.70	99.07	91.46	99.24	95.11
1524	48.68	48.68	50.00	97.36	49.33
1525	47.29	47.29	50.00	94.57	48.61
1526	81.47	97.45	82.91	98.81	89.59
1527	48.76	48.76	50.00	97.52	49.37
1528	48.53	48.53	50.00	97.07	49.26
1529	48.60	51.42	50.11	96.78	50.76
1530	49.38	49.38	50.00	98.76	49.69
1531	60.86	75.12	64.58	98.68	69.45
1532	49.38	49.38	50.00	98.75	49.69
1533	49.29	49.29	50.00	98.58	49.64
1534	48.55	98.45	50.10	96.90	66.40
1535	47.99	48.03	49.97	95.99	48.98
1536	47.81	47.81	50.00	95.62	48.88
1537	48.94	48.94	50.00	97.87	49.46
1538	49.26	49.26	50.00	98.53	49.63
1539	48.55	48.55	50.00	97.09	49.26
1540	48.28	48.28	50.00	96.56	49.12
1541	48.61	48.61	50.00	97.21	49.29
1542	72.58	84.62	78.70	98.85	81.55
1543	58.16	73.43	61.03	98.21	66.66
1544	86.65	97.94	87.98	99.33	92.69
1545	88.67	98.47	89.86	98.79	93.97
1546	77.89	95.88	80.02	97.94	87.23
1547	85.47	88.43	95.42	98.79	91.79
1548	70.81	98.93	71.74	98.21	83.17
1549	49.25	49.30	49.95	98.50	49.62

1550	49.30	49.30	50.00	98.61	49.65
1551	62.44	88.13	64.06	98.79	74.19
1552	65.14	83.24	68.72	98.18	75.29
1553	83.37	85.75	95.88	98.91	90.54
1554	77.92	91.43	81.91	98.83	86.41
1555	60.14	79.66	62.61	98.39	70.11
1556	77.71	98.38	78.55	99.05	87.35
1557	48.91	48.91	50.00	97.83	49.45
1558	49.10	49.10	50.00	98.21	49.55
1559	49.40	98.89	50.51	97.78	66.87
1560	63.16	98.10	64.66	97.11	77.94
1561	49.38	98.06	51.32	96.13	67.38
1562	53.77	98.11	55.66	96.23	71.02
1563	48.84	48.84	50.00	97.67	49.41
1564	54.52	99.14	55.38	98.28	71.07
1565	49.52	73.67	52.17	94.67	61.08
1566	47.18	47.18	50.00	94.37	48.55
1567	71.64	87.97	76.13	97.24	81.63
1568	54.20	97.91	56.29	95.85	71.49
1569	48.23	49.30	49.15	96.30	49.22
1570	52.63	98.98	53.65	97.96	69.58
1571	65.55	89.50	67.69	98.36	77.08
1572	73.79	85.33	80.46	97.77	82.82
1573	72.30	91.35	75.06	98.77	82.41
1574	48.84	48.84	50.00	97.69	49.42
1575	70.03	75.94	83.39	97.79	79.49
1576	48.93	48.93	50.00	97.85	49.46
1577	78.01	86.02	86.15	98.85	86.09
1578	77.96	97.60	79.39	98.17	87.56
1579	87.37	98.60	88.59	98.17	93.33
1580	85.43	97.63	87.06	98.52	92.04
1581	73.36	93.21	76.21	97.29	83.86
1582	65.97	95.05	68.01	96.77	79.29
1583	48.61	48.61	50.00	97.21	49.29
1584	48.94	48.94	50.00	97.89	49.47
1585	48.91	48.92	50.00	97.83	49.45
1586	48.88	48.88	50.00	97.76	49.43
1587	48.80	48.81	49.99	97.60	49.39
1588	48.76	48.76	50.00	97.51	49.37
1589	79.33	98.25	80.29	99.01	88.37
1590	69.85	99.46	70.27	99.22	82.35
1591	80.01	83.73	92.94	98.34	88.10
1592	51.65	94.66	54.77	93.80	69.39
1593	72.45	92.77	75.72	96.28	83.38
1594	74.29	87.12	79.65	98.74	83.22
1595	75.35	96.70	77.57	96.53	86.08
1596	75.78	98.59	76.88	98.09	86.39
1597	64.47	97.17	66.83	95.47	79.20
1598	47.98	47.98	50.00	95.96	48.97
1599	49.15	49.15	50.00	98.31	49.57
1600	63.02	98.97	64.05	97.96	77.77
1601	88.18	99.15	88.96	98.71	93.78
1602	52.69	96.78	55.71	94.00	70.72
1603	51.48	97.61	53.86	95.24	69.42
1604	58.64	98.28	60.36	96.60	74.79
1605	78.45	97.62	80.14	97.41	88.02
1606	49.27	49.27	50.00	98.54	49.63
1607	87.66	96.20	90.21	99.57	93.11
1608	50.37	96.84	52.66	95.43	68.22
1609	72.48	96.61	75.02	95.60	84.45
1610	76.25	96.12	78.71	96.48	86.55
1611	63.36	84.38	66.91	96.43	74.63

1612	49.13	54.70	50.56	96.97	52.55
1613	48.94	48.94	50.00	97.88	49.46
1614	65.99	96.18	67.40	97.83	79.26
1615	50.56	98.23	52.33	96.47	68.28
1616	48.78	48.78	50.00	97.56	49.38
1617	53.76	99.33	54.43	98.67	70.33
1618	51.82	92.54	53.27	97.13	67.62
1619	49.15	49.15	50.00	98.30	49.57
1620	48.92	49.26	49.65	97.84	49.46
1621	76.25	96.75	78.11	97.55	86.44
1622	86.00	96.15	88.82	97.35	92.34
1623	59.24	96.04	63.12	92.46	76.17
1624	83.37	94.63	86.68	98.20	90.48
1625	47.78	97.64	50.14	95.27	66.26
1626	67.63	97.49	69.72	96.06	81.30
1627	69.89	98.20	71.54	96.84	82.77
1628	49.30	49.30	50.00	98.61	49.65
1629	87.31	97.54	89.17	98.16	93.17
1630	85.67	98.69	86.91	97.78	92.43
1631	83.90	98.45	85.07	98.37	91.28
1632	65.68	99.17	66.51	98.36	79.62
1633	58.42	93.52	60.37	96.48	73.38
1634	53.50	96.42	56.87	93.35	71.54
1635	56.00	96.52	59.34	93.43	73.49
1636	71.37	89.61	75.22	97.00	81.79
1637	58.53	95.92	61.96	93.33	75.29
1638	80.97	97.73	82.54	97.90	89.50
1639	49.07	49.07	50.00	98.14	49.53
1640	58.76	99.11	59.65	98.22	74.48
1641	49.39	49.39	50.00	98.79	49.69
1642	49.14	49.14	50.00	98.29	49.57
1643	66.64	89.67	69.23	97.70	78.14
1644	63.09	96.76	65.57	95.27	78.17
1645	48.10	82.66	50.44	95.31	62.65
1646	64.91	83.13	68.31	98.45	74.99
1647	49.28	49.28	50.00	98.56	49.64
1648	63.77	99.46	64.31	98.92	78.11
1649	89.67	93.27	95.36	99.20	94.30
1650	49.12	49.13	49.99	98.23	49.55
1651	48.15	48.15	50.00	96.29	49.06
1652	56.72	98.80	57.92	97.61	73.03
1653	49.51	96.47	50.66	97.70	66.43
1654	75.97	99.06	76.86	98.30	86.56
1655	83.09	98.14	84.50	98.01	90.81
1656	73.12	97.34	75.23	96.24	84.87
1657	46.18	46.18	50.00	92.36	48.01
1658	71.80	86.26	77.38	96.68	81.58
1659	55.44	87.06	58.93	93.53	70.29
1660	71.60	97.19	74.14	95.26	84.12
1661	47.82	48.07	49.73	95.63	48.88
1662	83.98	96.21	86.51	97.75	91.10
1663	70.11	96.93	72.58	95.50	83.01
1664	56.75	89.12	58.71	96.63	70.79
1665	60.52	96.37	62.34	96.59	75.71
1666	58.45	96.09	62.28	92.54	75.57
1667	66.41	96.19	69.82	93.57	80.91
1668	49.21	49.21	50.00	98.41	49.60
1669	55.92	99.17	56.75	98.35	72.19
1670	52.65	97.95	54.70	95.91	70.20
1671	75.15	99.36	75.79	98.74	85.99
1672	48.39	48.39	50.00	96.79	49.18
1673	80.17	97.42	81.54	98.85	88.78

1674	88.29	98.65	89.52	98.07	93.86
1675	86.82	97.84	88.81	96.79	93.11
1676	81.02	97.75	83.06	96.47	89.81
1677	78.56	92.53	82.30	98.06	87.12
1678	82.72	85.49	95.18	98.66	90.08
1679	49.62	53.71	66.53	91.73	59.44
1680	48.71	48.71	50.00	97.42	49.35
1681	67.23	74.95	78.05	97.37	76.47
1682	69.97	99.00	70.92	98.14	82.64
1683	49.44	49.44	50.00	98.89	49.72
1684	66.86	98.52	68.30	97.17	80.67
1685	92.37	96.56	95.25	99.42	95.90
1686	56.30	82.11	57.96	97.69	67.96
1687	63.11	99.38	63.74	98.76	77.66
1688	56.61	96.93	59.63	94.05	73.84
1689	75.49	97.65	77.66	96.00	86.52
1690	63.40	96.90	66.12	94.76	78.60
1691	58.22	96.76	61.42	93.72	75.14
1692	74.92	93.34	78.63	95.36	85.36
1693	67.12	97.31	69.79	94.85	81.28
1694	48.90	48.93	49.97	97.80	49.44
1695	76.87	98.61	78.12	97.68	87.18
1696	71.45	97.47	73.18	97.02	83.59
1697	64.34	96.28	67.25	94.50	79.19
1698	52.07	96.96	54.99	94.20	70.18
1699	49.26	49.26	50.00	98.53	49.63
1700	74.18	99.53	74.65	99.06	85.31
1701	70.92	97.95	72.12	98.01	83.07
1702	64.95	77.85	72.42	94.48	75.04
1703	49.36	49.36	50.00	98.71	49.68
1704	79.42	98.89	80.41	98.20	88.70
1705	59.62	97.97	61.64	96.00	75.67
1706	73.31	98.11	74.59	97.85	84.75
1707	49.37	49.37	50.00	98.74	49.68
1708	49.36	49.36	50.00	98.72	49.68
1709	85.37	99.64	85.73	99.30	92.16
1710	58.85	93.97	61.62	94.82	74.43
1711	80.13	98.00	82.09	96.38	89.34
1712	70.84	97.34	73.33	95.30	83.64
1713	64.19	97.37	65.95	96.71	78.64
1714	74.20	97.40	75.42	98.50	85.02
1715	49.16	49.16	50.00	98.33	49.58
1716	48.83	48.83	50.00	97.66	49.41
1717	48.98	48.98	50.00	97.97	49.49
1718	47.92	49.09	49.24	95.53	49.16
1719	80.88	99.68	81.20	99.36	89.49
1720	55.54	99.51	56.03	99.02	71.69
1721	80.10	97.90	81.58	97.89	89.00
1722	77.41	89.64	83.23	95.79	86.32
1723	80.75	94.98	83.98	96.54	89.14
1724	63.68	96.11	66.18	95.36	78.39
1725	71.27	79.70	82.59	94.65	81.12
1726	84.62	94.89	88.26	96.76	91.45
1727	72.40	95.46	75.38	95.18	84.24
1728	54.63	99.42	55.22	98.83	71.00
1729	77.73	93.00	81.46	96.78	86.85
1730	80.82	97.34	82.95	96.66	89.57
1731	63.48	96.14	67.25	92.76	79.14
1732	49.41	96.83	52.58	93.68	68.15
1733	74.79	88.13	79.93	98.18	83.83
1734	72.39	92.60	75.42	97.00	83.13
1735	68.28	81.61	75.22	95.50	78.28

1736	73.02	87.17	78.87	95.81	82.81
1737	49.05	49.05	50.00	98.10	49.52
1738	84.13	99.00	84.87	98.99	91.39
1739	49.33	49.33	50.00	98.66	49.66
1740	83.38	95.41	86.36	97.58	90.66
1741	56.98	96.42	59.51	95.06	73.59
1742	49.42	49.42	50.00	98.84	49.71
1743	49.17	49.17	50.00	98.34	49.58
1744	48.19	48.19	50.00	96.37	49.08
1745	65.81	91.68	69.04	95.20	78.77
1746	47.06	47.06	50.00	94.12	48.49
1747	49.44	49.44	50.00	98.89	49.72
1748	84.09	98.36	85.43	97.98	91.44
1749	76.69	97.23	78.99	96.03	87.17
1750	49.39	49.39	50.00	98.79	49.70
1751	57.46	98.25	58.99	96.97	73.72
1752	78.52	99.10	79.38	98.37	88.15
1753	82.49	96.34	84.91	97.46	90.27
1754	57.20	97.14	59.61	95.27	73.88
1755	75.83	98.58	76.94	98.07	86.43
1756	72.63	98.07	74.33	96.83	84.56
1757	63.14	96.70	65.61	95.30	78.18
1758	53.76	97.24	55.36	96.83	70.55
1759	68.92	92.62	71.17	97.81	80.49
1760	63.55	94.82	66.75	94.15	78.35
1761	49.37	49.42	49.95	98.74	49.68
1762	45.39	95.10	50.29	90.20	65.79
1763	49.13	96.38	52.54	93.19	68.01
1764	48.58	48.58	50.00	97.16	49.28
1765	48.66	48.66	50.00	97.32	49.32
1766	48.79	48.79	50.00	97.59	49.39
1767	57.06	96.96	58.88	96.45	73.27
1768	84.13	98.73	85.26	98.10	91.50
1769	94.43	99.34	95.03	99.29	97.14
1770	70.33	94.00	72.62	97.32	81.94
1771	48.69	48.69	50.00	97.38	49.34
1772	48.42	48.42	50.00	96.84	49.20
1773	49.35	49.35	50.00	98.69	49.67
1774	48.95	48.95	50.00	97.90	49.47
1775	48.59	48.59	50.00	97.18	49.29
1776	47.24	47.24	50.00	94.47	48.58
1777	64.97	97.16	67.81	94.49	79.87
1778	74.65	94.52	77.82	95.68	85.36
1779	78.01	90.77	82.87	97.25	86.64
1780	56.71	66.52	61.80	96.25	64.07
1781	50.58	79.81	51.14	98.88	62.34
1782	63.91	95.99	64.94	98.54	77.47
1783	47.93	47.93	50.00	95.85	48.94
1784	64.15	75.89	70.14	98.34	72.90
1785	60.06	93.39	61.25	98.20	73.98
1786	48.78	49.64	49.92	97.28	49.78
1787	55.24	96.38	56.66	97.24	71.36
1788	63.99	96.05	66.38	95.62	78.51
1789	48.10	48.10	50.00	96.20	49.03
1790	49.20	49.20	50.00	98.41	49.60
1791	48.51	48.51	50.00	97.02	49.24
1792	63.13	96.38	66.01	94.50	78.36
1793	59.96	93.37	64.11	92.21	76.02
1794	55.42	97.24	58.11	94.68	72.75
1795	47.74	47.74	50.00	95.47	48.84
1796	48.91	48.91	50.00	97.82	49.45
1797	48.15	48.30	49.84	96.31	49.06

1798	49.06	49.06	50.00	98.11	49.52
1799	77.77	93.06	81.22	97.63	86.74
1800	57.73	96.61	60.17	95.23	74.16
1801	75.95	87.19	82.18	98.23	84.61
1802	89.67	99.51	90.06	99.59	94.55
1803	49.13	49.25	49.89	98.27	49.56
1804	48.98	49.07	49.91	97.96	49.48
1805	74.30	98.17	75.80	97.30	85.55
1806	86.82	90.38	95.02	98.21	92.64
1807	57.59	98.42	58.91	97.38	73.70
1808	48.55	48.55	50.00	97.11	49.27
1809	49.35	49.35	50.00	98.69	49.67
1810	49.26	49.26	50.00	98.51	49.63
1811	47.69	47.69	50.00	95.38	48.82
1812	62.39	96.83	65.21	94.53	77.94
1813	76.99	97.21	79.40	95.79	87.41
1814	76.98	96.60	79.11	97.06	86.98
1815	49.00	49.10	49.89	97.99	49.49
1816	51.13	80.71	52.93	96.47	63.93
1817	64.60	96.24	65.39	99.05	77.87
1818	66.52	82.95	71.61	96.29	76.87
1819	84.85	98.50	85.98	98.54	91.82
1820	66.64	98.95	67.63	98.06	80.35
1821	65.01	69.15	82.78	97.20	75.35
1822	59.92	99.31	60.60	98.63	75.27
1823	49.08	49.08	50.00	98.16	49.53
1824	60.81	95.17	61.81	98.46	74.94
1825	49.44	97.14	51.91	95.07	67.66
1826	60.07	94.74	63.91	92.67	76.33
1827	69.70	90.15	73.67	95.42	81.08
1828	66.53	92.13	68.86	97.20	78.81
1829	47.98	47.98	50.00	95.96	48.97
1830	63.75	94.57	66.27	95.64	77.93
1831	53.82	94.21	59.37	89.18	72.84
1832	59.24	89.52	65.71	88.08	75.79
1833	58.35	92.79	63.88	89.50	75.66
1834	49.19	49.19	50.00	98.38	49.59
1835	49.17	49.17	50.00	98.34	49.58
1836	56.47	86.46	59.04	95.56	70.17
1837	67.01	93.54	69.87	95.58	79.99
1838	48.56	48.56	50.00	97.13	49.27
1839	63.28	96.02	65.11	96.75	77.60
1840	49.05	50.48	50.20	97.23	50.34
1841	60.06	67.87	68.99	96.04	68.43
1842	75.80	95.07	78.14	97.70	85.78
1843	84.63	98.93	85.42	99.02	91.68
1844	57.55	98.59	58.96	97.20	73.79
1845	80.14	93.33	84.24	96.24	88.55
1846	80.51	96.71	83.70	94.44	89.74
1847	88.28	97.28	90.74	96.48	93.89
1848	93.29	98.69	94.50	98.44	96.55
1849	90.73	98.82	91.92	97.90	95.24
1850	49.44	49.44	50.00	98.88	49.72
1851	84.28	95.98	87.72	95.02	91.66
1852	88.27	96.95	91.03	96.07	93.90
1853	87.79	96.70	90.54	96.62	93.52
1854	70.51	96.57	73.93	93.57	83.75
1855	93.77	98.47	95.13	98.67	96.77
1856	94.75	98.99	95.70	98.79	97.32
1857	91.23	98.46	92.58	98.30	95.43
1858	93.29	98.20	94.85	98.79	96.50
1859	86.03	98.55	87.49	97.33	92.69

1860	53.72	95.28	58.44	90.74	72.45
1861	49.31	49.31	50.00	98.62	49.65
1862	90.01	98.99	91.01	98.16	94.83
1863	89.31	98.89	90.38	98.13	94.45
1864	87.71	97.17	89.94	97.64	93.41
1865	74.26	97.42	76.85	95.13	85.92
1866	90.68	95.89	94.07	98.24	94.97
1867	79.87	92.56	84.56	95.52	88.38
1868	77.21	97.14	80.04	94.80	87.77
1869	81.05	97.58	83.33	95.95	89.90
1870	71.36	91.95	75.91	93.37	83.16
1871	71.95	83.62	80.91	92.12	82.24
1872	49.33	49.33	50.00	98.66	49.66
1873	57.10	94.06	62.74	89.16	75.27
1874	65.75	94.17	70.86	90.61	80.87
1875	74.16	87.62	81.79	90.92	84.60
1876	67.78	83.91	72.24	98.05	77.64
1877	47.18	47.18	50.00	94.37	48.55
1878	64.75	96.11	67.74	94.38	79.47
1879	71.66	97.66	73.31	97.10	83.75
1880	77.49	94.17	79.89	99.19	86.44
1881	48.36	48.36	50.00	96.72	49.17
1882	48.39	48.39	50.00	96.78	49.18
1883	49.26	49.26	50.00	98.51	49.63
1884	48.59	48.59	50.00	97.18	49.28
1885	49.21	49.21	50.00	98.41	49.60
1886	90.69	98.66	91.78	98.95	95.10
1887	63.12	95.88	67.24	92.09	79.05
1888	78.63	96.61	81.92	94.18	88.66
1889	86.59	95.56	90.05	96.47	92.72
1890	70.88	96.51	74.37	93.46	84.01
1891	83.29	97.23	85.99	95.34	91.27
1892	76.84	94.75	81.21	92.92	87.46
1893	86.70	97.77	88.90	96.26	93.12
1894	49.29	49.29	50.00	98.59	49.64
1895	55.63	94.48	61.03	89.53	74.16
1896	43.98	43.98	50.00	87.95	46.80
1897	92.69	98.15	94.34	98.20	96.20
1898	67.83	96.47	71.36	93.31	82.03
1899	77.16	97.27	79.83	95.09	87.69
1900	64.52	95.71	68.81	91.84	80.06
1901	92.21	98.74	93.38	98.30	95.99
1902	87.90	98.63	89.26	97.54	93.71
1903	91.13	99.02	92.06	98.50	95.41
1904	93.57	98.95	94.52	98.90	96.68
1905	49.35	49.35	50.00	98.70	49.67
1906	93.80	99.16	94.57	99.01	96.81
1907	96.32	99.49	96.80	99.46	98.13
1908	71.90	97.17	74.72	94.64	84.48
1909	86.77	98.71	88.02	97.76	93.06
1910	71.61	98.11	73.48	96.39	84.03
1911	79.37	97.73	81.59	95.92	88.93
1912	82.51	97.53	84.89	95.81	90.78
1913	53.45	94.69	58.76	89.62	72.52
1914	70.74	96.07	74.63	92.80	84.00
1915	79.10	97.30	81.71	95.30	88.82
1916	57.36	85.47	59.85	96.01	70.40
1917	70.10	92.82	72.91	96.68	81.67
1918	53.33	98.99	54.34	97.99	70.16
1919	56.43	97.87	58.47	95.96	73.20
1920	45.58	45.58	50.00	91.16	47.69
1921	55.21	96.88	58.33	93.84	72.81

1922	49.23	49.23	50.00	98.46	49.61
1923	49.14	49.14	50.00	98.28	49.57
1924	70.01	95.09	71.66	98.23	81.73
1925	49.28	49.38	49.90	98.56	49.64
1926	49.20	49.20	50.00	98.40	49.60
1927	49.12	49.12	50.00	98.25	49.56
1928	78.16	97.91	79.72	97.49	87.89
1929	48.92	48.92	50.00	97.84	49.45
1930	48.83	48.83	50.00	97.67	49.41
1931	61.58	82.39	64.25	98.00	72.20
1932	60.39	78.27	64.63	95.64	70.80
1933	71.86	98.16	73.40	97.15	83.99
1934	48.57	48.57	50.00	97.14	49.27
1935	48.96	48.96	50.00	97.91	49.47
1936	51.63	98.47	53.16	96.94	69.04
1937	49.12	49.12	50.00	98.23	49.55
1938	47.65	47.65	50.00	95.31	48.80
1939	62.46	76.71	67.45	97.13	71.78
1940	83.40	98.55	84.48	98.51	90.98
1941	49.38	49.38	50.00	98.75	49.69
1942	49.36	49.36	50.00	98.72	49.68
1943	49.10	49.10	50.00	98.21	49.55
1944	67.79	82.89	72.46	98.58	77.32
1945	50.36	98.34	52.02	96.69	68.04
1946	65.56	96.50	68.95	93.53	80.43
1947	68.61	96.76	71.70	94.15	82.37
1948	87.22	98.63	88.47	97.94	93.28
1949	54.78	96.01	58.77	92.16	72.91
1950	48.50	48.50	50.00	97.00	49.24
1951	62.59	99.30	63.29	98.61	77.30
1952	48.15	48.15	50.00	96.30	49.06
1953	48.48	48.48	50.00	96.96	49.23
1954	48.72	48.72	50.00	97.45	49.35
1955	49.21	49.24	49.97	98.42	49.60
1956	48.88	48.88	50.00	97.76	49.43
1957	48.26	48.41	49.84	96.52	49.12
1958	48.48	48.48	50.00	96.95	49.23
1959	48.08	48.08	50.00	96.16	49.02
1960	49.14	49.14	50.00	98.28	49.57
1961	57.08	98.90	58.18	97.81	73.26
1962	49.19	49.29	49.90	98.37	49.59
1963	46.98	46.99	49.99	93.96	48.44
1964	50.17	56.82	58.85	88.52	57.82
1965	48.35	48.35	50.00	96.71	49.16
1966	51.32	98.52	52.80	97.05	68.76
1967	48.71	48.71	50.00	97.43	49.35
1968	48.91	48.91	50.00	97.82	49.45
1969	48.92	48.92	50.00	97.84	49.45
1970	70.66	96.63	71.80	98.77	82.38
1971	48.83	48.83	50.00	97.65	49.41
1972	48.27	48.27	50.00	96.55	49.12
1973	48.85	48.85	50.00	97.70	49.42
1974	50.07	98.37	51.70	96.74	67.78
1975	64.23	96.93	66.89	94.90	79.15
1976	73.42	96.44	76.70	94.02	85.45
1977	89.68	98.83	90.69	98.61	94.58
1978	86.93	96.35	89.47	98.50	92.79
1979	82.52	97.51	83.84	99.32	90.16
1980	51.02	98.25	52.77	96.51	68.66
1981	71.37	99.42	71.95	98.86	83.48
1982	59.09	95.24	60.67	97.13	74.12
1983	49.26	49.26	50.00	98.51	49.63

1984	50.73	97.45	53.27	94.92	68.89
1985	59.34	90.06	61.84	95.84	73.33
1986	51.78	88.39	54.13	95.39	67.14
1987	57.22	84.37	58.98	97.52	69.43
1988	48.32	48.32	50.00	96.63	49.14
1989	50.19	96.71	53.31	93.79	68.73
1990	52.31	97.51	54.80	95.05	70.17
1991	49.10	49.10	50.00	98.20	49.54
1992	49.17	49.17	50.00	98.34	49.58
1993	48.56	48.56	50.00	97.11	49.27
1994	49.29	49.29	50.00	98.59	49.64
1995	52.45	79.49	53.20	98.71	63.74
1996	48.80	54.49	50.51	96.37	52.43
1997	65.66	85.86	69.70	95.96	76.94
1998	60.35	87.27	63.70	94.77	73.65
1999	68.91	96.02	72.54	93.25	82.64
2000	44.06	93.07	50.99	86.19	65.88
2001	85.47	98.67	86.71	97.84	92.31
2002	88.32	96.47	90.87	98.83	93.58
2003	73.88	98.36	75.40	97.11	85.37
2004	49.09	49.09	50.00	98.19	49.54
2005	48.90	48.90	50.00	97.81	49.45
2006	48.29	48.29	50.00	96.59	49.13
2007	71.21	97.65	73.20	96.30	83.68
2008	46.80	46.80	50.00	93.60	48.35
2009	49.09	49.09	50.00	98.18	49.54
2010	48.71	48.72	49.99	97.42	49.35
2011	47.53	47.53	50.00	95.06	48.73
2012	48.24	48.24	50.00	96.49	49.11
2013	68.62	80.75	75.88	96.48	78.24
2014	66.08	86.98	69.90	96.05	77.51
2015	59.92	88.97	62.50	95.94	73.42
2016	61.21	95.21	62.34	98.20	75.35
2017	67.14	96.75	69.42	95.85	80.84
2018	51.60	97.96	53.65	95.93	69.33
2019	49.42	49.42	50.00	98.84	49.71
2020	49.43	49.43	50.00	98.86	49.71
2021	52.10	64.85	53.45	97.98	58.60
2022	49.37	98.32	51.05	96.64	67.20
2023	74.89	94.76	77.79	96.25	85.44
2024	61.19	88.73	65.22	93.31	75.18
2025	46.80	46.80	50.00	93.60	48.35
2026	47.39	86.86	52.76	89.26	65.64
2027	48.77	48.77	50.00	97.54	49.38
2028	48.53	48.53	50.00	97.06	49.25
2029	58.60	94.92	63.52	90.52	76.11
2030	77.11	97.68	79.28	96.03	87.52
2031	74.26	81.10	87.25	94.03	84.07
2032	48.16	95.37	52.79	90.78	67.96
2033	49.25	49.25	50.00	98.51	49.62
2034	48.64	48.69	49.95	97.28	49.31
2035	60.51	87.89	62.68	97.11	73.17
2036	60.66	97.55	63.02	95.37	76.57
2037	61.71	97.68	63.47	96.59	76.95
2038	71.70	85.60	77.07	98.04	81.11
2039	49.27	49.27	50.00	98.55	49.63
2040	48.66	48.73	49.93	97.33	49.32
2041	48.16	48.84	49.29	96.31	49.06
2042	59.66	86.12	62.89	95.04	72.70
2043	51.07	95.22	54.82	92.56	69.58
2044	57.48	95.56	60.36	94.42	73.99
2045	50.82	98.05	52.08	97.47	68.03

2046	48.98	48.98	50.00	97.95	49.48
2047	60.47	75.39	63.75	98.88	69.09
2048	48.56	48.56	50.00	97.12	49.27
2049	48.57	48.57	50.00	97.14	49.27
2050	48.86	48.86	50.00	97.71	49.42
2051	68.19	98.45	69.54	97.40	81.51
2052	69.22	96.34	71.72	95.60	82.23
2053	53.57	95.98	57.42	92.41	71.86
2054	45.42	45.42	50.00	90.84	47.60
2055	53.71	97.47	56.13	95.20	71.24
2056	47.52	93.20	52.61	89.85	67.26
2057	51.86	94.45	57.41	89.12	71.41
2058	53.17	96.12	56.86	92.72	71.45
2059	67.44	96.19	70.29	94.79	81.22
2060	76.61	97.34	78.96	95.78	87.20
2061	60.32	93.90	62.28	96.58	74.89
2062	55.05	99.24	55.81	98.48	71.44
2063	75.78	88.81	80.93	98.00	84.69
2064	68.04	98.90	69.09	97.94	81.35
2065	66.59	85.19	69.68	99.10	76.66
2066	61.63	90.86	62.83	98.82	74.29
2067	65.79	88.31	68.08	98.67	76.88
2068	48.98	49.32	49.64	97.95	49.48
2069	48.90	48.90	50.00	97.79	49.44
2070	56.90	77.48	58.82	98.05	66.87
2071	48.53	48.53	50.00	97.05	49.25
2072	46.76	46.76	50.00	93.53	48.33
2073	70.65	76.67	84.27	97.07	80.29
2074	48.55	48.55	50.00	97.09	49.26
2075	49.31	49.31	50.00	98.62	49.65
2076	54.92	62.66	59.67	96.42	61.13
2077	48.62	48.62	50.00	97.24	49.30
2078	48.20	48.20	50.00	96.39	49.08
2079	48.09	48.09	50.00	96.19	49.03
2080	49.33	49.33	50.00	98.65	49.66
2081	66.82	98.26	68.21	97.36	80.52
2082	65.98	97.83	68.11	95.83	80.31
2083	49.24	98.93	50.32	97.85	66.71
2084	60.19	96.85	63.27	93.97	76.54
2085	60.79	94.14	65.03	91.99	76.93
2086	64.14	96.20	67.81	92.96	79.55
2087	54.42	94.92	58.92	91.17	72.71
2088	49.57	90.42	54.45	90.31	67.97
2089	63.91	93.53	66.66	95.39	77.85
2090	46.93	46.93	50.00	93.87	48.42
2091	61.45	90.11	65.11	93.84	75.60
2092	77.78	94.19	80.37	98.68	86.74
2093	81.89	98.07	83.02	98.95	89.92
2094	48.86	48.86	50.00	97.71	49.42
2095	49.07	49.14	49.93	98.14	49.53
2096	49.17	49.17	50.00	98.35	49.58
2097	49.05	49.05	50.00	98.11	49.52
2098	49.65	52.43	50.26	98.72	51.32
2099	52.71	69.15	55.17	95.92	61.37
2100	47.50	48.58	49.16	94.83	48.86
2101	49.06	49.06	50.00	98.11	49.52
2102	68.33	97.00	70.03	97.09	81.34
2103	69.13	98.16	70.77	96.86	82.25
2104	73.21	97.42	74.68	97.78	84.55
2105	49.27	49.27	50.00	98.54	49.63
2106	71.14	96.73	73.13	96.73	83.29
2107	62.96	93.94	66.58	93.42	77.93

2108	52.10	95.46	56.52	91.26	71.00
2109	61.73	96.39	65.25	93.19	77.82
2110	45.40	45.40	50.00	90.79	47.59
2111	74.96	95.35	77.96	95.51	85.78
2112	61.71	83.49	66.57	93.14	74.08
2113	72.68	97.16	74.83	96.24	84.54
2114	67.62	94.38	71.28	93.65	81.22
2115	49.09	49.09	50.00	98.18	49.54
2116	49.16	49.16	50.00	98.32	49.58
2117	49.09	49.09	50.00	98.18	49.54
2118	48.83	48.83	50.00	97.66	49.41
2119	48.83	48.83	50.00	97.66	49.41
2120	49.24	49.24	50.00	98.49	49.62
2121	53.68	98.63	55.05	97.27	70.66
2122	59.16	99.24	59.92	98.48	74.72
2123	49.30	49.30	50.00	98.60	49.65
2124	48.29	97.97	50.32	95.95	66.49
2125	47.96	47.96	50.00	95.92	48.96
2126	60.05	98.94	61.10	97.89	75.55
2127	48.52	97.59	50.93	95.18	66.93
2128	74.91	97.90	76.99	96.06	86.19
2129	78.72	97.42	80.68	96.88	88.27
2130	53.83	96.41	56.48	94.77	71.23
2131	50.37	93.85	54.77	91.28	69.17
2132	47.05	47.05	50.00	94.10	48.48
2133	49.12	49.12	50.00	98.24	49.56
2134	63.60	84.46	67.39	96.02	74.97
2135	69.45	90.99	72.84	96.21	80.91
2136	76.41	97.59	78.02	97.51	86.72
2137	81.53	97.96	82.99	97.99	89.85
2138	59.74	96.75	61.66	96.31	75.32
2139	49.26	49.26	50.00	98.52	49.63
2140	70.41	96.44	73.32	94.73	83.30
2141	78.47	81.37	93.67	98.86	87.09
2142	84.80	90.07	92.24	99.13	91.14
2143	48.56	48.56	50.00	97.12	49.27
2144	65.32	98.34	66.90	96.93	79.63
2145	81.48	93.32	85.04	99.30	88.99
2146	48.30	48.30	50.00	96.60	49.13
2147	55.20	97.55	56.63	97.18	71.66
2148	87.11	96.48	89.47	99.15	92.84
2149	83.82	91.72	89.42	98.20	90.56
2150	49.41	49.41	50.00	98.82	49.70
2151	73.81	85.06	80.66	97.85	82.80
2152	68.83	87.43	72.19	98.46	79.09
2153	49.05	49.05	50.00	98.11	49.52
2154	49.25	49.25	50.00	98.50	49.62
2155	48.84	48.84	50.00	97.69	49.42
2156	48.98	48.98	50.00	97.95	49.48
2157	48.83	48.83	50.00	97.66	49.41
2158	62.64	89.15	65.19	96.65	75.31
2159	49.06	98.41	50.64	96.83	66.87
2160	70.59	98.53	71.86	97.59	83.11
2161	48.42	48.42	50.00	96.85	49.20
2162	81.14	98.64	82.48	97.47	89.84
2163	72.72	96.92	74.47	97.35	84.22
2164	52.60	79.06	55.35	94.83	65.12
2165	56.13	87.01	58.62	95.63	70.05
2166	58.45	77.32	61.43	97.00	68.46
2167	73.11	90.53	77.00	97.01	83.22
2168	84.39	96.71	86.55	98.19	91.35
2169	49.54	98.62	50.92	97.24	67.17

2170	48.23	48.23	50.00	96.46	49.10
2171	87.57	99.45	87.98	99.56	93.37
2172	61.78	96.67	64.54	94.68	77.40
2173	85.38	96.63	87.43	99.27	91.80
2174	49.47	49.47	50.00	98.94	49.73
2175	48.59	48.59	50.00	97.18	49.29
2176	49.12	49.12	50.00	98.25	49.56
2177	83.09	89.75	90.17	98.75	89.96
2178	69.03	98.15	70.80	96.56	82.26
2179	46.57	46.57	50.00	93.14	48.23
2180	82.23	98.94	83.26	98.07	90.43
2181	89.33	96.59	91.85	99.11	94.16
2182	87.69	97.03	89.73	99.07	93.24
2183	45.33	45.33	50.00	90.67	47.55
2184	76.56	99.35	77.04	99.18	86.79
2185	49.16	49.16	50.00	98.33	49.58
2186	48.91	49.36	49.66	97.76	49.51
2187	48.63	48.63	50.00	97.26	49.31
2188	50.92	97.96	52.51	96.83	68.37
2189	49.40	99.29	50.11	98.58	66.60
2190	84.89	96.54	86.99	99.11	91.52
2191	47.08	47.08	50.00	94.16	48.50
2192	68.12	82.63	73.76	97.03	77.95
2193	52.97	68.14	54.63	97.63	60.64
2194	73.25	94.81	74.99	98.90	83.74
2195	75.88	89.46	80.81	97.57	84.92
2196	69.31	97.77	71.47	95.86	82.57
2197	82.03	98.43	83.35	97.83	90.26
2198	71.46	98.09	73.03	97.09	83.72
2199	55.17	98.55	56.62	97.11	71.92
2200	52.12	99.00	53.12	98.00	69.14
2201	69.66	87.11	73.19	98.99	79.54
2202	49.21	49.21	50.00	98.43	49.60
2203	49.33	49.33	50.00	98.67	49.67
2204	72.95	98.18	74.36	97.46	84.63
2205	48.40	68.20	50.20	96.39	57.83
2206	84.71	96.96	86.95	97.44	91.68
2207	72.84	97.46	75.27	95.42	84.94
2208	60.07	96.59	62.20	95.93	75.67
2209	62.63	90.12	65.38	95.97	75.78
2210	61.03	97.53	63.16	95.83	76.67
2211	80.73	97.38	82.23	98.58	89.16
2212	53.70	95.56	57.18	93.13	71.55
2213	81.30	95.70	84.28	96.34	89.63
2214	68.53	96.86	71.44	94.49	82.23
2215	49.00	91.66	50.19	97.61	64.86
2216	48.02	48.39	49.61	96.04	48.99
2217	75.14	97.30	76.97	97.07	85.95
2218	83.02	98.15	84.46	97.89	90.79
2219	74.18	87.59	79.92	96.67	83.58
2220	46.83	46.83	50.00	93.67	48.37
2221	78.48	95.12	81.64	95.93	87.87
2222	84.00	94.19	88.07	96.50	91.03
2223	62.93	95.90	65.98	94.22	78.18
2224	70.59	96.79	73.60	94.37	83.62
2225	77.43	94.64	80.89	95.39	87.23
2226	68.91	96.00	72.27	93.84	82.46
2227	48.82	48.82	50.00	97.64	49.40
Average	61.05	77.98	63.23	96.94	69.06
Standard c	13.69	22.35	14.24	1.83	16.33
Confidence	0.57	0.93	0.59	0.08	0.68
