# Supplementary material for: In Situ Root Dataset Expansion Strategy Based on an Improved CycleGAN Generator
Source: Plant Phenomics. 2024 Feb 12;6:0148. doi: 10.34133/plantphenomics.0148 (PMC11020132; doi:10.34133/plantphenomics.0148)
Supplement: Supplementary 1 — The network and corresponding weights can be viewed on GitHub (https://github.com/jiwd123/improved_cyclegan) and Zenodo (https://doi.org/10.5281/zenodo.10460303). [file plantphenomics.0148.f1.zip › generalization evaluation_Ws+Wgs.pdf]

No.	IOU	Recall	Precision	Accuracy	F1
1	96.34	99.14	97.13	99.61	98.12
2	96.16	98.38	97.65	99.62	98.01
3	95.57	97.95	97.44	99.50	97.69
4	95.14	97.99	96.95	99.36	97.47
5	94.91	97.58	97.07	99.63	97.33
6	95.46	98.99	96.35	99.60	97.65
7	95.18	98.46	96.54	99.59	97.49
8	97.30	99.78	97.51	99.77	98.63
9	97.72	98.97	98.71	99.61	98.84
10	97.93	99.41	98.50	99.81	98.95
11	90.49	90.98	99.37	99.61	94.99
12	96.38	97.69	98.58	99.71	98.13
13	96.06	97.39	98.54	99.60	97.96
14	92.32	94.19	97.63	99.80	95.88
15	97.53	98.61	98.86	99.69	98.74
16	97.49	99.15	98.30	99.69	98.72
17	95.53	97.73	97.60	99.80	97.67
18	95.83	98.57	97.11	99.72	97.83
19	96.30	97.51	98.67	99.90	98.09
20	94.86	98.02	96.61	99.57	97.31
21	93.72	98.26	95.20	99.35	96.71
22	91.76	96.33	94.76	99.56	95.54
23	90.01	91.15	98.42	99.53	94.65
24	95.27	99.51	95.69	99.87	97.56
25	96.36	98.41	97.84	99.56	98.12
26	95.57	97.87	97.53	99.37	97.70
27	96.87	99.64	97.21	99.66	98.41
28	97.13	98.96	98.10	99.67	98.53
29	94.79	97.81	96.75	99.30	97.28
30	96.76	98.22	98.45	99.60	98.33
31	96.40	97.83	98.46	99.50	98.15
32	96.69	98.48	98.12	99.51	98.30
33	96.40	98.45	97.83	99.73	98.14
34	96.89	99.58	97.28	99.68	98.42
35	97.85	99.26	98.55	99.77	98.90
36	97.26	99.54	97.69	99.71	98.61
37	98.05	99.35	98.68	99.78	99.01
38	98.32	99.49	98.81	99.77	99.15
39	97.46	98.50	98.91	99.69	98.70
40	97.67	99.00	98.62	99.73	98.81
41	97.21	99.60	97.59	99.66	98.59
42	96.44	99.48	96.92	99.66	98.18
43	94.33	95.61	98.50	99.58	97.04
44	91.60	92.93	98.29	99.51	95.53
45	95.20	97.25	97.72	99.67	97.49
46	97.67	99.67	97.98	99.81	98.82
47	96.91	99.60	97.28	99.74	98.43
48	96.24	97.74	98.36	99.83	98.05
49	94.55	95.54	98.85	99.67	97.16
50	95.07	96.21	98.70	99.65	97.44
51	94.59	96.31	98.02	99.66	97.16
52	95.55	97.96	97.40	99.70	97.68
53	90.88	93.02	97.24	99.24	95.08
54	90.40	92.26	97.49	99.55	94.80
55	95.19	96.48	98.52	99.85	97.49
56	92.58	94.32	97.84	99.67	96.05
57	92.49	94.34	97.71	99.62	95.99
58	96.20	98.19	97.88	99.82	98.03
59	94.71	96.23	98.25	99.70	97.23
60	94.49	96.68	97.52	99.68	97.10
61	97.24	99.24	97.94	99.87	98.59

62	96.85	99.49	97.32	99.67	98.39
63	97.34	99.20	98.09	99.71	98.64
64	97.10	99.39	97.66	99.67	98.52
65	97.69	99.71	97.96	99.69	98.83
66	95.95	98.12	97.66	99.76	97.89
67	97.48	99.30	98.14	99.70	98.72
68	98.04	98.81	99.20	99.77	99.00
69	97.36	99.49	97.84	99.67	98.66
70	97.57	98.94	98.58	99.77	98.76
71	94.88	95.66	99.09	99.65	97.35
72	94.48	96.64	97.55	99.63	97.09
73	88.60	90.46	97.32	99.45	93.76
74	95.00	98.00	96.77	99.79	97.38
75	88.70	90.17	97.81	99.66	93.84
76	92.54	93.51	98.80	99.51	96.08
77	93.80	95.42	98.08	99.54	96.73
78	92.80	94.87	97.47	99.73	96.15
79	89.48	91.31	97.45	99.47	94.28
80	95.01	97.76	97.02	99.75	97.38
81	92.86	94.66	97.79	99.69	96.20
82	89.75	90.55	98.92	99.52	94.55
83	94.88	95.24	99.58	99.86	97.36
84	90.89	97.15	93.05	99.75	95.05
85	93.43	94.09	99.18	99.73	96.57
86	94.71	95.74	98.80	99.55	97.25
87	95.04	97.36	97.43	99.77	97.40
88	93.76	96.01	97.38	99.73	96.69
89	96.48	97.73	98.63	99.91	98.18
90	95.25	95.73	99.44	99.78	97.55
91	96.37	97.73	98.52	99.85	98.12
92	97.29	98.40	98.83	99.80	98.61
93	96.10	98.62	97.34	99.87	97.98
94	92.26	93.64	98.23	99.75	95.88
95	91.81	92.52	99.07	99.71	95.69
96	95.04	96.49	98.34	99.76	97.40
97	95.98	98.38	97.44	99.87	97.91
98	92.25	93.59	98.31	99.52	95.89
99	94.35	95.63	98.50	99.61	97.04
100	93.67	95.49	97.85	99.54	96.66
101	95.33	96.54	98.62	99.75	97.57
102	93.14	95.01	97.75	99.48	96.36
103	96.75	98.34	98.30	99.76	98.32
104	97.00	98.65	98.27	99.80	98.46
105	95.04	95.89	99.01	99.82	97.42
106	94.91	96.61	98.07	99.82	97.33
107	91.36	91.76	99.48	99.71	95.47
108	94.65	95.52	98.97	99.84	97.21
109	94.24	99.11	94.98	99.85	97.00
110	94.36	96.17	97.92	99.58	97.04
111	89.08	89.58	99.32	99.63	94.20
112	95.90	97.77	97.97	99.49	97.87
113	96.85	98.57	98.19	99.60	98.38
114	96.99	98.98	97.93	99.81	98.45
115	95.50	95.91	99.53	99.83	97.69
116	90.08	97.99	91.53	99.60	94.65
117	90.12	91.97	97.48	99.51	94.65
118	94.34	98.63	95.51	99.49	97.05
119	96.07	98.76	97.20	99.48	97.98
120	96.30	97.53	98.65	99.62	98.09
121	95.33	98.66	96.52	99.51	97.58
122	96.83	98.18	98.56	99.73	98.37
123	96.58	99.50	97.03	99.68	98.25

124	96.41	99.59	96.78	99.78	98.17
125	97.82	98.93	98.85	99.92	98.89
126	96.21	97.44	98.63	99.91	98.04
127	94.75	97.83	96.65	99.75	97.24
128	94.61	95.83	98.57	99.87	97.18
129	92.50	95.84	96.09	99.61	95.96
130	92.83	94.65	97.78	99.62	96.19
131	95.78	96.88	98.76	99.86	97.81
132	94.89	99.54	95.31	99.55	97.38
133	94.79	98.70	95.91	99.60	97.29
134	96.16	99.49	96.63	99.54	98.04
135	96.98	98.86	98.04	99.61	98.45
136	96.36	98.61	97.64	99.56	98.12
137	97.44	99.53	97.89	99.72	98.70
138	96.29	99.45	96.81	99.48	98.11
139	96.04	98.69	97.24	99.39	97.96
140	96.76	99.12	97.59	99.44	98.34
141	95.03	96.25	98.63	99.29	97.43
142	92.60	93.22	99.22	99.73	96.13
143	89.64	99.44	90.10	99.41	94.54
144	94.21	95.54	98.43	99.65	96.96
145	93.54	99.16	94.24	99.63	96.64
146	95.33	97.71	97.41	99.65	97.56
147	96.35	98.22	98.01	99.45	98.12
148	96.92	99.35	97.53	99.52	98.43
149	96.83	99.61	97.20	99.62	98.39
150	97.34	99.51	97.79	99.84	98.65
151	94.51	97.75	96.49	99.53	97.11
152	92.03	95.12	96.34	98.91	95.72
153	98.33	99.21	99.10	99.81	99.16
154	97.64	99.39	98.22	99.71	98.80
155	90.36	92.30	97.43	99.29	94.80
156	97.34	98.77	98.51	99.58	98.64
157	97.38	99.55	97.81	99.57	98.67
158	97.12	99.58	97.51	99.66	98.54
159	97.08	99.24	97.79	99.68	98.51
160	96.19	99.31	96.82	99.48	98.05
161	95.83	98.72	96.98	99.56	97.84
162	96.30	97.58	98.60	99.73	98.09
163	95.92	96.90	98.91	99.58	97.89
164	96.41	96.98	99.37	99.74	98.16
165	96.48	99.40	97.03	99.69	98.20
166	91.55	93.37	97.66	99.57	95.46
167	97.58	99.04	98.49	99.79	98.77
168	92.45	97.58	94.42	99.42	95.98
169	95.13	97.08	97.84	99.30	97.46
170	96.78	99.16	97.55	99.55	98.35
171	97.43	99.42	97.98	99.65	98.69
172	98.13	99.52	98.59	99.78	99.05
173	98.31	99.13	99.15	99.91	99.14
174	94.57	96.70	97.60	99.48	97.15
175	94.47	97.74	96.48	99.18	97.10
176	95.93	98.42	97.36	99.57	97.89
177	94.23	95.61	98.37	99.77	96.97
178	94.75	95.59	99.01	99.75	97.27
179	96.58	97.66	98.82	99.74	98.24
180	97.41	97.87	99.51	99.81	98.68
181	96.72	98.88	97.76	99.72	98.32
182	96.29	98.95	97.23	99.72	98.09
183	96.37	97.26	99.01	99.87	98.13
184	94.98	97.07	97.67	99.64	97.37
185	91.24	91.87	99.26	99.32	95.42

186	92.29	92.80	99.36	99.67	95.97
187	97.11	98.76	98.27	99.81	98.51
188	91.00	98.48	92.12	99.71	95.19
189	96.45	98.01	98.32	99.69	98.17
190	94.57	95.51	98.88	99.86	97.16
191	97.42	98.82	98.53	99.90	98.68
192	97.42	98.75	98.61	99.73	98.68
193	96.11	97.55	98.42	99.59	97.99
194	96.39	96.87	99.46	99.85	98.15
195	94.87	96.24	98.43	99.65	97.32
196	95.14	98.23	96.72	99.55	97.47
197	93.52	94.59	98.70	99.68	96.60
198	96.60	97.47	99.05	99.78	98.25
199	88.21	89.63	98.01	99.09	93.63
200	96.05	97.02	98.92	99.74	97.96
201	96.79	98.84	97.87	99.70	98.35
202	97.23	98.17	99.00	99.74	98.58
203	89.76	90.40	99.13	99.58	94.56
204	91.88	92.79	98.87	99.37	95.73
205	93.68	94.20	99.39	99.60	96.72
206	90.33	91.49	98.41	99.52	94.83
207	79.79	81.92	95.48	99.10	88.18
208	94.39	94.84	99.46	99.76	97.10
209	96.64	98.08	98.46	99.60	98.27
210	89.11	92.90	95.01	99.41	93.94
211	95.89	97.80	97.93	99.66	97.87
212	96.99	98.49	98.41	99.89	98.45
213	96.94	98.23	98.62	99.85	98.42
214	94.86	95.88	98.82	99.65	97.33
215	95.34	97.08	98.06	99.66	97.57
216	96.01	96.99	98.90	99.89	97.93
217	96.64	97.73	98.82	99.72	98.27
218	97.19	98.51	98.60	99.77	98.56
219	97.07	98.36	98.63	99.74	98.49
220	96.84	98.38	98.38	99.72	98.38
221	95.84	96.68	99.05	99.71	97.85
222	87.20	93.76	91.67	99.58	92.71
223	92.98	94.14	98.58	99.52	96.31
224	93.99	95.36	98.39	99.50	96.85
225	91.61	93.69	97.36	99.51	95.49
226	90.30	92.26	97.45	98.97	94.78
227	86.17	88.00	97.47	98.21	92.49
228	81.29	93.60	84.63	99.32	88.89
229	92.74	93.89	98.57	99.56	96.17
230	94.43	95.58	98.67	99.38	97.10
231	94.55	96.39	97.89	99.65	97.14
232	91.96	93.43	98.12	99.56	95.72
233	94.52	95.85	98.45	99.62	97.13
234	92.89	95.06	97.37	99.77	96.20
235	96.24	98.07	98.04	99.70	98.05
236	96.44	99.06	97.29	99.72	98.17
237	96.90	97.82	99.00	99.86	98.41
238	97.53	98.29	99.19	99.81	98.74
239	94.67	96.75	97.66	99.60	97.20
240	91.90	93.01	98.60	99.37	95.73
241	93.51	94.24	99.12	99.57	96.62
242	94.07	96.78	96.94	99.58	96.86
243	93.31	95.11	97.89	99.14	96.48
244	95.69	97.47	98.04	99.88	97.75
245	88.52	89.50	98.92	98.40	93.98
246	85.62	87.34	97.15	99.42	91.99
247	95.32	96.82	98.31	99.80	97.56

248	89.59	91.98	96.80	99.12	94.33
249	88.85	93.60	93.92	99.54	93.76
250	83.10	92.76	87.41	99.36	90.01
251	84.84	85.98	98.13	99.30	91.65
252	93.22	95.91	96.84	99.73	96.37
253	90.34	92.62	96.94	99.68	94.73
254	91.97	93.13	98.52	99.60	95.75
255	94.39	95.10	99.15	99.83	97.08
256	92.49	93.73	98.45	99.53	96.03
257	94.06	95.96	97.78	99.65	96.86
258	82.65	84.54	96.77	98.62	90.25
259	91.22	98.00	92.73	99.64	95.29
260	91.53	92.20	99.22	99.25	95.58
261	91.33	93.44	97.28	99.63	95.32
262	94.27	94.64	99.55	99.84	97.03
263	90.09	95.33	93.82	98.90	94.57
264	92.69	94.19	98.21	98.91	96.16
265	91.73	93.61	97.63	99.36	95.57
266	98.02	99.23	98.76	99.87	98.99
267	88.97	90.37	98.08	99.16	94.06
268	82.78	84.82	96.99	97.72	90.50
269	83.52	84.94	97.57	99.04	90.82
270	95.27	95.59	99.62	99.85	97.57
271	92.97	99.55	93.33	99.79	96.34
272	90.70	92.00	98.25	99.53	95.02
273	89.67	91.91	97.03	98.97	94.40
274	88.32	91.74	95.31	99.13	93.49
275	89.38	91.36	97.21	99.60	94.20
276	93.02	96.06	96.45	99.76	96.26
277	95.37	99.17	96.11	99.57	97.61
278	88.30	89.09	98.86	99.51	93.72
279	91.06	94.90	95.33	99.56	95.11
280	93.46	94.33	98.96	99.49	96.59
281	93.94	95.66	98.00	99.42	96.81
282	89.83	91.78	97.32	99.46	94.47
283	85.02	86.86	96.95	99.23	91.63
284	87.13	88.50	97.89	99.44	92.96
285	91.19	93.37	97.18	99.61	95.24
286	91.70	94.14	96.92	99.70	95.51
287	95.58	96.43	99.02	99.76	97.71
288	94.04	97.40	96.32	99.40	96.86
289	92.31	93.43	98.68	98.99	95.98
290	91.26	92.88	97.96	99.05	95.35
291	93.80	94.98	98.56	99.81	96.74
292	95.30	97.50	97.58	99.83	97.54
293	92.17	96.46	95.11	99.46	95.78
294	84.78	86.84	96.95	98.18	91.61
295	87.08	88.70	97.62	98.96	92.95
296	85.98	87.26	98.16	98.78	92.39
297	95.93	97.36	98.42	99.78	97.89
298	95.72	99.16	96.45	99.87	97.79
299	95.93	97.65	98.12	99.71	97.89
300	97.25	97.81	99.39	99.89	98.59
301	94.69	95.84	98.64	99.83	97.22
302	93.66	96.51	96.74	99.80	96.62
303	87.28	88.98	97.38	99.50	92.99
304	88.37	90.35	97.10	99.55	93.60
305	94.98	96.07	98.75	99.55	97.39
306	94.77	96.20	98.36	99.59	97.27
307	84.58	87.04	95.89	99.15	91.25
308	88.33	90.68	96.66	99.19	93.57
309	89.14	92.15	95.88	99.55	93.98

310	93.60	98.97	94.45	99.73	96.65
311	91.58	93.64	97.38	99.39	95.48
312	93.58	95.27	98.01	99.25	96.62
313	92.52	97.59	94.49	99.38	96.02
314	88.19	90.11	97.16	99.48	93.51
315	91.24	95.71	94.76	99.47	95.23
316	92.47	96.52	95.42	99.16	95.97
317	93.76	96.15	97.25	99.43	96.70
318	91.34	98.00	92.91	99.25	95.39
319	92.78	96.77	95.52	99.26	96.14
320	91.26	94.20	96.36	99.27	95.27
321	95.42	98.59	96.69	99.39	97.63
322	86.83	90.33	94.88	99.19	92.55
323	95.64	96.96	98.52	99.67	97.73
324	91.72	94.17	97.00	99.00	95.56
325	86.24	96.96	88.13	99.43	92.33
326	95.88	97.82	97.90	99.70	97.86
327	94.46	97.38	96.78	99.45	97.08
328	89.97	95.14	93.86	98.92	94.50
329	93.02	94.72	97.99	99.04	96.32
330	93.93	96.06	97.57	99.10	96.81
331	89.92	94.90	94.08	98.36	94.49
332	75.74	79.38	95.27	94.08	86.60
333	85.07	89.48	93.26	99.49	91.33
334	96.66	97.94	98.62	99.71	98.28
335	96.76	98.28	98.39	99.57	98.33
336	96.13	97.97	98.04	99.37	98.00
337	96.92	98.92	97.92	99.75	98.42
338	93.17	98.71	94.30	98.93	96.45
339	92.21	94.10	97.78	98.40	95.91
340	95.03	97.18	97.64	99.30	97.41
341	95.76	97.90	97.70	99.41	97.80
342	95.31	97.38	97.74	99.34	97.56
343	95.08	97.97	96.91	99.34	97.43
344	91.90	94.40	96.92	99.36	95.64
345	95.01	98.54	96.32	99.22	97.42
346	94.79	97.42	97.13	99.17	97.28
347	93.94	98.29	95.41	99.20	96.83
348	94.25	98.52	95.54	99.17	97.01
349	93.65	95.71	97.63	99.01	96.66
350	94.05	95.86	97.93	99.03	96.88
351	95.35	98.77	96.46	99.29	97.60
352	95.42	97.35	97.89	99.37	97.62
353	95.37	98.05	97.14	99.48	97.59
354	69.96	71.49	95.38	98.35	81.73
355	92.89	96.92	95.47	99.67	96.19
356	91.02	92.18	98.52	99.23	95.24
357	92.36	94.37	97.53	99.43	95.92
358	84.99	87.28	96.23	99.17	91.54
359	85.41	88.18	95.76	98.57	91.82
360	88.00	98.95	88.81	99.11	93.61
361	94.65	98.16	96.25	99.54	97.20
362	94.44	96.65	97.51	99.35	97.08
363	92.62	95.91	96.17	99.42	96.04
364	89.39	92.10	96.45	98.68	94.22
365	95.29	97.22	97.88	99.33	97.55
366	92.87	94.19	98.36	99.66	96.23
367	96.35	98.78	97.48	99.40	98.12
368	95.68	96.91	98.65	99.20	97.77
369	93.75	98.19	95.31	99.22	96.73
370	96.87	98.49	98.28	99.64	98.39
371	95.00	97.96	96.84	99.13	97.39

372	95.27	97.09	98.01	98.94	97.55
373	92.83	99.00	93.74	98.79	96.30
374	96.24	97.41	98.72	99.55	98.06
375	96.15	97.44	98.60	99.47	98.01
376	94.77	98.48	96.12	99.08	97.29
377	96.04	97.66	98.24	99.71	97.95
378	95.94	98.06	97.74	99.35	97.90
379	87.12	89.99	96.18	97.52	92.98
380	88.94	93.85	94.00	97.82	93.92
381	90.37	93.66	95.93	98.39	94.78
382	93.82	99.15	94.60	98.97	96.82
383	95.06	96.80	98.06	99.16	97.43
384	94.23	97.12	96.81	99.12	96.96
385	94.77	97.14	97.38	99.26	97.26
386	96.05	98.90	97.06	99.49	97.97
387	96.81	97.98	98.75	99.60	98.36
388	96.59	98.71	97.79	99.61	98.25
389	97.37	98.83	98.48	99.72	98.65
390	95.98	96.88	99.01	99.44	97.93
391	95.48	97.10	98.21	99.30	97.65
392	72.69	77.16	93.75	92.74	84.65
393	86.30	93.10	91.46	97.64	92.27
394	94.96	97.08	97.64	99.39	97.36
395	95.14	98.70	96.29	99.50	97.48
396	93.16	99.08	93.96	99.22	96.45
397	78.28	83.64	90.25	96.95	86.82
398	92.70	94.23	98.08	99.73	96.12
399	97.38	99.09	98.23	99.70	98.66
400	90.22	92.13	97.40	99.62	94.69
401	97.05	97.57	99.44	99.81	98.49
402	91.69	92.94	98.43	99.22	95.61
403	88.39	89.56	98.25	99.61	93.70
404	92.47	93.33	98.97	99.35	96.07
405	94.77	98.72	95.89	99.41	97.28
406	95.93	98.32	97.46	99.76	97.89
407	93.98	95.73	97.95	99.49	96.83
408	95.97	97.79	98.03	99.46	97.91
409	96.21	99.03	97.09	99.45	98.05
410	96.48	99.33	97.10	99.66	98.20
411	95.49	97.98	97.34	99.34	97.66
412	96.03	98.98	96.96	99.44	97.96
413	96.33	99.49	96.80	99.47	98.13
414	95.15	97.30	97.65	99.19	97.47
415	94.56	98.72	95.72	98.92	97.19
416	96.10	98.36	97.62	99.36	97.99
417	97.20	98.25	98.87	99.81	98.56
418	95.06	97.16	97.67	99.42	97.42
419	95.27	99.10	96.09	99.29	97.57
420	88.15	89.43	98.21	99.06	93.62
421	95.67	98.74	96.79	99.70	97.76
422	87.79	95.41	91.04	99.15	93.18
423	89.44	95.38	93.03	98.77	94.19
424	94.38	96.34	97.75	99.60	97.04
425	94.40	95.57	98.61	99.71	97.07
426	95.18	96.40	98.61	99.76	97.49
427	94.31	97.23	96.75	99.84	96.99
428	88.64	90.59	97.15	99.64	93.75
429	88.61	95.05	92.24	99.67	93.62
430	95.90	97.18	98.59	99.51	97.88
431	96.27	98.90	97.28	99.51	98.08
432	95.85	98.49	97.21	99.72	97.85
433	95.68	96.85	98.68	99.83	97.76

434	95.32	97.82	97.28	99.82	97.55
435	92.99	97.47	95.09	99.63	96.26
436	93.01	95.11	97.47	99.61	96.27
437	93.88	95.96	97.57	99.64	96.76
438	94.25	96.14	97.82	99.67	96.97
439	95.85	96.52	99.25	99.71	97.86
440	95.82	98.42	97.24	99.71	97.83
441	97.15	98.46	98.60	99.91	98.53
442	95.31	97.84	97.26	99.68	97.55
443	96.62	97.18	99.39	99.73	98.27
444	93.21	95.40	97.39	99.67	96.39
445	93.41	95.00	98.13	99.20	96.54
446	95.49	99.41	96.01	99.67	97.68
447	93.04	93.38	99.62	99.61	96.40
448	92.05	95.59	95.82	99.51	95.70
449	88.82	89.94	98.35	99.60	93.96
450	90.84	92.54	97.70	99.73	95.05
451	89.79	90.55	98.93	99.65	94.55
452	92.74	93.73	98.77	99.55	96.19
453	94.67	98.25	96.20	99.55	97.21
454	96.09	98.07	97.87	99.78	97.97
455	95.49	96.10	99.31	99.65	97.68
456	95.54	98.79	96.62	99.44	97.70
457	95.27	97.13	97.95	99.39	97.54
458	94.52	95.75	98.56	99.69	97.14
459	92.27	98.61	93.36	99.60	95.92
460	92.47	93.96	98.14	99.58	96.00
461	94.80	96.53	98.02	99.72	97.27
462	91.83	92.80	98.73	99.70	95.67
463	93.39	94.44	98.75	99.41	96.55
464	81.05	82.09	98.53	98.52	89.57
465	92.11	93.16	98.63	99.77	95.82
466	95.30	96.85	98.25	99.81	97.55
467	85.03	87.84	95.81	98.01	91.65
468	89.57	91.22	97.81	98.94	94.40
469	93.34	94.85	98.17	99.64	96.48
470	92.81	94.05	98.46	99.64	96.20
471	90.61	92.21	97.86	99.46	94.95
472	86.29	91.02	93.40	98.62	92.19
473	74.14	76.70	94.79	96.96	84.79
474	95.39	97.08	98.12	99.86	97.59
475	94.40	97.28	96.82	99.56	97.05
476	95.04	96.12	98.77	99.54	97.43
477	90.37	92.84	96.74	99.57	94.75
478	94.37	94.72	99.59	99.78	97.10
479	90.24	90.99	98.97	99.68	94.81
480	92.88	94.28	98.27	99.60	96.23
481	94.27	96.74	97.20	99.82	96.97
482	87.64	89.79	96.92	98.89	93.22
483	93.85	96.07	97.44	99.28	96.75
484	95.28	96.67	98.42	99.81	97.54
485	80.69	83.18	95.47	98.22	88.90
486	86.39	88.32	97.28	98.33	92.58
487	94.52	95.43	98.91	99.75	97.14
488	85.42	91.62	91.43	99.36	91.52
489	85.27	86.96	97.37	98.87	91.87
490	95.44	97.48	97.76	99.72	97.62
491	91.49	94.23	96.56	99.70	95.38
492	78.77	80.18	97.66	98.25	88.06
493	92.91	93.87	98.78	99.81	96.26
494	97.42	97.85	99.54	99.84	98.69
495	97.10	99.32	97.73	99.77	98.52

496	98.00	98.45	99.53	99.84	98.99
497	97.22	97.57	99.63	99.82	98.59
498	94.50	95.06	99.33	99.78	97.15
499	95.91	97.22	98.54	99.77	97.88
500	95.40	97.76	97.43	99.74	97.60
501	93.29	95.07	97.84	99.68	96.44
502	88.42	89.89	98.12	98.45	93.82
503	94.41	96.87	97.24	99.43	97.06
504	95.34	97.31	97.82	99.87	97.56
505	93.41	95.16	97.89	99.66	96.51
506	88.57	90.30	97.51	99.33	93.77
507	96.39	98.19	98.07	99.76	98.13
508	97.11	98.56	98.48	99.76	98.52
509	96.08	98.82	97.14	99.73	97.97
510	95.79	99.36	96.36	99.77	97.84
511	97.28	98.50	98.71	99.81	98.61
512	98.20	99.02	99.15	99.84	99.08
513	97.37	98.77	98.53	99.71	98.65
514	96.31	96.76	99.50	99.87	98.11
515	94.43	95.69	98.51	99.70	97.08
516	94.00	94.55	99.35	99.68	96.89
517	90.74	91.85	98.53	99.39	95.08
518	85.41	91.89	91.28	98.65	91.58
519	90.06	90.84	98.93	99.61	94.71
520	90.01	99.30	90.54	99.60	94.72
521	97.96	99.46	98.47	99.81	98.97
522	97.35	98.41	98.87	99.72	98.64
523	97.43	98.35	99.03	99.81	98.69
524	91.99	93.81	97.68	99.72	95.70
525	93.66	94.58	98.87	99.73	96.68
526	95.82	96.43	99.33	99.66	97.86
527	95.72	98.29	97.27	99.71	97.78
528	96.00	96.37	99.58	99.89	97.95
529	94.16	94.61	99.46	99.80	96.97
530	77.97	79.27	97.61	98.54	87.48
531	82.22	84.54	95.73	98.96	89.79
532	97.92	98.92	98.97	99.65	98.94
533	96.85	99.46	97.35	99.69	98.39
534	97.34	98.65	98.62	99.73	98.64
535	93.51	97.66	95.47	99.81	96.55
536	90.76	92.76	97.38	99.32	95.01
537	89.53	95.17	93.25	99.48	94.20
538	95.85	96.96	98.76	99.66	97.85
539	96.67	97.73	98.85	99.77	98.28
540	95.76	97.34	98.25	99.89	97.79
541	93.16	95.19	97.57	99.51	96.36
542	94.06	97.33	96.38	99.74	96.85
543	96.08	97.82	98.13	99.26	97.98
544	85.86	87.05	98.12	99.25	92.26
545	96.62	99.62	96.97	99.85	98.28
546	97.76	99.65	98.09	99.90	98.86
547	93.62	94.73	98.65	99.80	96.65
548	94.33	96.73	97.27	99.74	97.00
549	95.73	96.64	98.98	99.65	97.80
550	95.81	96.97	98.71	99.66	97.83
551	92.40	93.14	99.08	99.54	96.02
552	94.37	95.16	99.06	99.67	97.07
553	94.08	96.41	97.33	99.67	96.87
554	96.21	97.76	98.34	99.37	98.05
555	93.60	95.53	97.71	99.72	96.61
556	93.17	97.94	94.86	99.64	96.38
557	93.68	94.76	98.68	99.71	96.68

558	93.87	95.54	98.02	99.72	96.76
559	96.75	98.79	97.88	99.53	98.33
560	96.39	99.31	97.03	99.53	98.15
561	95.72	98.62	96.97	99.53	97.79
562	96.65	98.07	98.48	99.55	98.28
563	89.68	94.62	94.06	98.54	94.33
564	96.16	97.70	98.33	99.49	98.02
565	92.08	93.73	98.05	98.58	95.84
566	95.69	97.01	98.55	99.16	97.78
567	95.30	97.74	97.38	99.15	97.56
568	96.50	98.82	97.58	99.47	98.20
569	93.70	96.06	97.26	99.59	96.66
570	95.63	98.72	96.78	99.48	97.74
571	94.97	98.51	96.30	99.31	97.39
572	96.57	98.81	97.68	99.53	98.24
573	96.53	98.67	97.76	99.67	98.21
574	95.92	99.05	96.76	99.84	97.89
575	93.13	94.26	98.59	99.78	96.38
576	94.08	95.41	98.43	99.55	96.90
577	94.63	96.66	97.70	99.72	97.18
578	94.15	97.00	96.81	99.54	96.91
579	95.85	97.02	98.70	99.68	97.85
580	86.06	87.90	96.99	99.56	92.22
581	97.51	99.54	97.95	99.77	98.74
582	97.72	99.60	98.10	99.80	98.84
583	85.83	86.71	98.65	99.34	92.30
584	97.47	97.74	99.71	99.88	98.71
585	98.29	98.70	99.56	99.91	99.13
586	96.47	97.86	98.52	99.16	98.19
587	96.74	98.53	98.13	99.23	98.33
588	95.97	98.47	97.37	99.57	97.91
589	96.00	98.60	97.28	99.36	97.94
590	96.12	97.68	98.31	99.54	97.99
591	94.13	95.08	98.86	99.76	96.93
592	89.68	96.01	92.73	98.98	94.34
593	95.33	97.00	98.16	99.17	97.58
594	95.77	97.31	98.33	99.09	97.82
595	95.55	98.68	96.75	99.15	97.71
596	96.34	98.29	97.93	99.41	98.11
597	96.92	98.65	98.18	99.50	98.42
598	97.02	98.46	98.48	99.55	98.47
599	97.09	98.02	99.01	99.57	98.51
600	96.48	98.69	97.69	99.54	98.19
601	96.74	99.27	97.41	99.63	98.33
602	93.61	94.92	98.43	99.57	96.64
603	83.41	84.83	97.39	99.45	90.68
604	81.90	83.01	97.90	99.32	89.84
605	87.36	91.04	94.72	99.63	92.84
606	92.60	93.93	98.38	99.25	96.10
607	93.74	97.37	96.04	98.99	96.70
608	90.96	93.37	97.00	98.75	95.15
609	89.21	91.07	97.52	98.79	94.19
610	89.78	96.26	92.72	98.34	94.46
611	93.18	96.07	96.70	98.86	96.38
612	92.79	96.24	96.06	99.12	96.15
613	93.73	95.16	98.29	99.63	96.70
614	94.31	97.94	96.12	99.16	97.02
615	93.59	96.80	96.45	98.53	96.63
616	93.64	96.66	96.65	98.45	96.66
617	93.01	96.74	95.85	98.80	96.29
618	89.87	94.95	93.90	99.12	94.42
619	88.54	90.06	97.76	99.59	93.75

620	60.70	62.92	89.16	96.81	73.78
621	84.37	86.11	97.01	99.26	91.23
622	90.99	92.30	98.25	99.63	95.18
623	70.90	72.38	96.06	98.27	82.56
624	95.47	97.56	97.72	99.43	97.64
625	91.42	92.47	98.61	99.63	95.44
626	88.82	96.42	91.37	99.45	93.83
627	92.51	97.03	94.96	99.65	95.98
628	94.85	95.68	99.04	99.68	97.33
629	90.73	92.92	97.19	99.05	95.01
630	85.90	87.38	97.61	99.38	92.21
631	80.26	82.04	96.30	99.18	88.60
632	94.80	98.04	96.55	99.20	97.29
633	94.21	98.25	95.75	98.94	96.98
634	88.64	91.55	96.13	98.43	93.78
635	95.38	96.97	98.25	99.23	97.61
636	90.38	94.55	94.94	98.84	94.74
637	95.58	96.75	98.70	99.46	97.71
638	95.71	98.13	97.43	99.39	97.78
639	91.18	94.56	95.92	98.81	95.23
640	92.52	96.36	95.60	99.49	95.98
641	89.29	91.11	97.41	99.63	94.16
642	84.33	86.88	95.59	99.50	91.03
643	88.10	89.90	97.30	99.61	93.45
644	92.21	93.34	98.58	99.51	95.89
645	91.69	95.22	95.75	99.66	95.49
646	95.42	96.09	99.24	99.49	97.64
647	92.19	92.82	99.20	99.63	95.90
648	92.39	93.85	98.16	99.65	95.95
649	84.30	86.08	96.96	99.22	91.19
650	92.18	95.63	95.91	99.80	95.77
651	89.64	95.05	93.50	99.40	94.27
652	86.63	88.41	97.27	99.17	92.63
653	95.86	97.87	97.83	99.67	97.85
654	95.21	97.34	97.66	99.34	97.50
655	94.61	96.15	98.22	99.58	97.17
656	92.74	98.34	94.06	99.66	96.15
657	90.94	91.95	98.65	99.62	95.18
658	94.61	97.17	97.16	99.51	97.17
659	95.31	97.77	97.33	99.62	97.55
660	93.58	97.41	95.78	99.74	96.59
661	94.44	97.88	96.28	99.74	97.07
662	94.36	94.97	99.27	99.74	97.07
663	96.27	97.96	98.18	99.72	98.07
664	95.33	96.06	99.16	99.69	97.59
665	89.65	89.89	99.77	99.54	94.57
666	90.72	95.33	94.49	99.60	94.91
667	93.24	94.89	98.02	99.51	96.43
668	90.23	93.47	95.80	99.67	94.62
669	94.03	96.35	97.35	99.39	96.85
670	91.47	92.46	98.72	99.47	95.49
671	96.27	98.90	97.27	99.79	98.08
672	96.41	98.40	97.90	99.45	98.15
673	96.47	98.39	97.96	99.64	98.18
674	94.27	96.33	97.63	99.64	96.98
675	93.28	95.08	97.87	99.21	96.46
676	95.50	96.71	98.64	99.55	97.67
677	96.40	96.80	99.56	99.88	98.16
678	96.57	97.91	98.55	99.70	98.23
679	96.13	96.45	99.65	99.81	98.02
680	95.40	97.73	97.46	99.80	97.59
681	95.52	97.05	98.30	99.65	97.67

682	96.23	97.21	98.92	99.76	98.06
683	79.55	80.67	98.16	98.62	88.56
684	72.10	73.53	96.27	98.43	83.38
685	90.96	92.71	97.74	99.28	95.16
686	93.07	95.49	97.13	99.54	96.30
687	87.66	88.73	98.39	99.48	93.31
688	90.04	93.56	95.46	99.66	94.50
689	95.22	96.65	98.37	99.84	97.50
690	95.30	98.07	97.03	99.60	97.55
691	96.17	99.22	96.88	99.53	98.04
692	96.23	97.63	98.48	99.43	98.05
693	96.05	96.92	99.02	99.85	97.96
694	94.49	96.56	97.65	99.63	97.10
695	95.34	96.85	98.32	99.53	97.58
696	95.23	96.69	98.35	99.67	97.51
697	95.58	96.49	98.95	99.89	97.71
698	96.27	97.08	99.10	99.83	98.08
699	76.66	79.05	94.92	97.96	86.26
700	79.85	84.19	91.94	98.25	87.89
701	95.33	95.76	99.51	99.76	97.60
702	94.09	95.09	98.80	99.84	96.91
703	85.67	91.60	91.75	99.58	91.68
704	93.94	95.46	98.19	99.78	96.80
705	95.10	97.08	97.78	99.82	97.43
706	96.16	97.98	98.04	99.60	98.01
707	94.56	98.08	96.25	99.31	97.16
708	95.07	97.24	97.59	99.52	97.42
709	95.73	98.82	96.79	99.54	97.79
710	97.01	98.67	98.25	99.87	98.46
711	93.37	94.23	98.94	99.70	96.53
712	93.85	94.57	99.12	99.78	96.79
713	93.03	95.52	97.05	99.54	96.28
714	93.18	93.56	99.53	99.77	96.45
715	91.05	94.88	95.34	99.55	95.11
716	92.81	93.75	98.83	99.62	96.22
717	89.42	93.14	95.13	99.59	94.12
718	87.74	92.00	94.19	99.29	93.08
719	84.65	87.18	95.86	98.97	91.31
720	88.07	94.08	92.54	99.06	93.30
721	85.46	89.26	94.16	99.23	91.65
722	94.82	96.08	98.53	99.86	97.29
723	95.85	97.12	98.58	99.76	97.85
724	94.39	95.37	98.85	99.62	97.08
725	90.44	92.34	97.46	99.45	94.83
726	83.16	83.79	99.18	99.06	90.84
727	90.40	91.70	98.23	99.52	94.86
728	87.35	88.73	97.89	99.41	93.09
729	74.06	75.70	95.70	98.66	84.53
730	95.01	96.42	98.39	99.66	97.39
731	94.66	96.07	98.38	99.41	97.21
732	94.42	96.85	97.28	99.37	97.07
733	93.86	98.89	94.79	99.58	96.80
734	92.16	93.75	97.96	99.76	95.81
735	94.38	97.17	96.90	99.64	97.04
736	93.41	94.18	99.07	99.58	96.56
737	94.74	95.62	98.98	99.66	97.27
738	94.11	95.70	98.14	99.48	96.90
739	95.41	98.43	96.80	99.62	97.61
740	94.07	98.11	95.67	99.70	96.88
741	91.07	96.24	94.04	99.71	95.13
742	91.37	93.47	97.34	99.19	95.37
743	89.91	92.40	96.76	98.82	94.53

744	92.89	95.44	96.97	99.53	96.20
745	92.22	94.71	96.94	99.60	95.81
746	96.31	98.76	97.45	99.73	98.10
747	90.35	94.84	94.53	99.55	94.69
748	89.17	95.17	92.82	99.50	93.98
749	74.59	75.91	97.28	98.38	85.28
750	72.83	75.82	91.40	98.37	82.89
751	89.67	91.34	97.69	99.42	94.41
752	88.96	93.20	94.49	99.37	93.84
753	89.95	92.58	96.52	99.30	94.51
754	89.34	91.95	96.44	99.42	94.14
755	95.69	97.76	97.76	99.69	97.76
756	95.80	98.85	96.83	99.57	97.83
757	90.54	91.22	99.08	99.69	94.99
758	96.28	97.82	98.33	99.71	98.08
759	95.54	96.66	98.74	99.59	97.69
760	94.44	97.01	97.13	99.46	97.07
761	89.87	91.60	97.68	99.22	94.54
762	88.27	90.39	96.90	99.43	93.54
763	90.23	91.53	98.23	99.51	94.76
764	91.17	95.07	95.28	99.65	95.18
765	85.35	86.33	98.39	99.45	91.96
766	93.59	97.68	95.53	99.83	96.59
767	94.56	95.01	99.47	99.86	97.19
768	91.08	92.28	98.43	99.46	95.26
769	84.10	94.94	87.09	99.30	90.84
770	93.42	94.59	98.59	99.50	96.55
771	92.67	93.66	98.76	99.67	96.14
772	92.46	95.20	96.72	99.46	95.95
773	92.54	95.36	96.65	99.27	96.00
774	92.53	93.22	99.15	99.56	96.09
775	93.56	97.84	95.35	99.83	96.58
776	96.30	97.70	98.48	99.85	98.08
777	92.01	93.49	98.15	99.30	95.76
778	95.83	98.42	97.27	99.57	97.84
779	87.86	88.66	98.82	99.54	93.46
780	95.38	97.73	97.44	99.60	97.59
781	93.54	94.19	99.23	99.62	96.64
782	94.59	95.63	98.78	99.62	97.18
783	93.07	93.76	99.17	99.55	96.39
784	96.59	97.65	98.85	99.87	98.24
785	95.99	97.92	97.92	99.68	97.92
786	96.74	98.74	97.91	99.73	98.32
787	95.67	98.52	96.99	99.73	97.75
788	95.85	97.22	98.47	99.75	97.84
789	85.33	90.77	92.37	98.42	91.56
790	91.15	97.36	93.16	99.71	95.21
791	90.49	93.43	96.27	99.00	94.83
792	93.36	95.16	97.84	99.56	96.48
793	90.42	92.22	97.58	99.49	94.82
794	88.73	90.94	96.88	99.31	93.82
795	88.33	90.59	96.76	99.28	93.58
796	75.40	77.30	95.43	98.47	85.42
797	74.75	76.66	95.85	97.96	85.18
798	85.80	87.84	96.67	99.44	92.04
799	89.62	91.05	97.98	99.60	94.39
800	94.36	94.92	99.33	99.82	97.07
801	94.59	95.46	98.98	99.75	97.19
802	93.35	95.23	97.78	99.26	96.49
803	94.55	97.74	96.56	99.24	97.15
804	95.99	98.52	97.33	99.45	97.93
805	95.06	97.41	97.42	99.49	97.42

806	89.62	96.11	92.51	99.53	94.28
807	90.84	92.21	98.14	99.70	95.08
808	91.16	91.78	99.16	99.76	95.33
809	92.33	94.03	97.85	99.69	95.90
810	94.26	98.34	95.68	99.64	96.99
811	94.28	98.07	95.96	99.32	97.00
812	95.60	96.39	99.11	99.57	97.73
813	88.13	90.51	97.00	97.54	93.64
814	94.77	98.29	96.28	99.26	97.27
815	91.27	92.22	98.79	99.40	95.39
816	94.44	97.14	97.01	99.33	97.08
817	93.12	97.47	95.26	99.25	96.35
818	95.60	97.86	97.56	99.68	97.71
819	95.19	96.84	98.16	99.52	97.49
820	94.92	95.87	98.92	99.48	97.37
821	95.55	96.96	98.42	99.67	97.68
822	95.96	98.25	97.55	99.80	97.90
823	95.93	97.70	98.08	99.77	97.89
824	93.37	97.98	95.08	99.21	96.51
825	94.22	95.44	98.59	99.36	96.99
826	88.23	91.76	95.27	98.47	93.48
827	95.13	96.97	97.94	99.68	97.45
828	83.05	86.53	93.87	99.47	90.05
829	94.79	96.05	98.54	99.75	97.28
830	95.25	97.13	97.92	99.57	97.52
831	90.53	93.19	96.58	99.14	94.86
832	93.12	97.94	94.81	99.67	96.35
833	90.37	98.13	91.72	99.72	94.82
834	80.09	99.43	80.53	99.26	88.99
835	95.59	97.02	98.41	99.64	97.71
836	89.08	92.42	95.51	99.50	93.94
837	91.01	94.40	95.77	99.72	95.08
838	82.81	83.42	99.01	99.39	90.55
839	78.21	84.66	87.92	99.08	86.26
840	88.13	89.29	98.28	99.50	93.57
841	86.95	89.73	95.77	99.55	92.65
842	86.62	88.35	97.28	99.43	92.60
843	92.01	93.25	98.39	99.69	95.75
844	90.63	93.12	96.78	99.35	94.91
845	93.14	94.53	98.33	99.43	96.39
846	91.21	92.66	98.11	99.41	95.31
847	93.95	95.20	98.50	99.67	96.82
848	95.09	96.40	98.51	99.64	97.44
849	94.54	96.90	97.35	99.77	97.12
850	93.12	94.79	97.96	99.67	96.35
851	93.54	95.63	97.59	99.05	96.60
852	93.00	96.98	95.60	98.92	96.29
853	92.55	96.21	95.81	99.20	96.01
854	93.22	97.22	95.60	99.34	96.40
855	95.69	97.94	97.58	99.54	97.76
856	95.41	98.57	96.70	99.35	97.62
857	95.84	98.59	97.10	99.87	97.84
858	95.73	97.42	98.16	99.29	97.79
859	92.16	95.73	95.87	98.77	95.80
860	88.04	90.26	97.04	98.21	93.53
861	93.59	95.53	97.75	99.12	96.63
862	95.87	97.20	98.54	99.43	97.87
863	94.22	97.05	96.86	99.33	96.96
864	94.80	98.62	96.00	99.58	97.29
865	92.57	94.60	97.49	99.73	96.02
866	93.33	96.87	96.02	99.73	96.44
867	95.58	97.45	97.96	99.36	97.70

868	96.36	97.95	98.29	99.52	98.12
869	90.82	92.54	97.94	98.26	95.17
870	93.44	95.48	97.61	99.23	96.53
871	92.48	96.61	95.30	99.66	95.95
872	90.70	95.66	94.17	99.42	94.91
873	89.22	92.96	95.13	99.12	94.03
874	89.60	91.55	97.29	99.52	94.33
875	87.45	88.43	98.50	99.50	93.19
876	88.12	90.54	96.46	99.60	93.41
877	93.35	93.90	99.32	99.79	96.53
878	93.79	95.73	97.73	99.54	96.72
879	91.15	97.11	93.37	99.76	95.20
880	89.24	91.56	97.05	98.11	94.22
881	91.78	93.88	97.49	98.46	95.65
882	86.89	94.85	90.49	98.89	92.62
883	53.88	57.92	89.54	92.18	70.34
884	90.43	92.29	97.50	99.56	94.82
885	91.53	92.51	98.75	99.40	95.53
886	96.31	97.34	98.87	99.62	98.10
887	94.50	96.07	98.22	99.17	97.13
888	93.63	95.52	97.81	99.05	96.65
889	92.63	97.29	94.91	99.02	96.08
890	92.02	93.41	98.23	99.53	95.76
891	94.39	97.40	96.69	99.51	97.04
892	95.59	97.95	97.45	99.80	97.70
893	93.91	96.21	97.36	99.36	96.78
894	91.89	93.84	97.67	98.53	95.72
895	92.28	94.29	97.61	98.63	95.92
896	90.89	92.65	97.90	98.23	95.20
897	91.12	93.20	97.52	98.16	95.31
898	89.86	91.65	97.74	98.56	94.59
899	87.27	89.54	96.81	98.46	93.04
900	94.69	96.07	98.43	99.34	97.24
901	83.42	84.58	97.90	99.39	90.76
902	94.20	96.35	97.55	99.38	96.94
903	94.87	97.76	96.87	99.56	97.31
904	93.74	96.22	97.15	99.45	96.68
905	93.44	95.20	97.90	99.51	96.53
906	94.71	96.34	98.14	99.47	97.24
907	94.31	95.74	98.33	99.60	97.02
908	90.45	91.35	98.84	99.31	94.95
909	85.47	87.79	96.61	98.12	91.99
910	83.01	85.25	96.13	98.74	90.37
911	97.98	98.73	99.21	99.86	98.97
912	96.45	98.12	98.21	99.72	98.16
913	97.25	98.32	98.86	99.81	98.59
914	96.34	98.95	97.29	99.75	98.11
915	95.98	98.89	96.99	99.39	97.93
916	84.98	87.46	96.85	96.81	91.92
917	89.39	94.83	93.49	98.65	94.15
918	72.93	98.29	73.85	98.62	84.33
919	91.44	94.18	96.61	99.27	95.38
920	94.93	98.49	96.26	99.31	97.36
921	91.80	94.57	96.62	99.17	95.59
922	93.20	94.47	98.46	99.54	96.42
923	94.19	99.13	94.94	99.44	96.99
924	94.52	96.43	97.83	99.53	97.12
925	96.67	98.06	98.50	99.86	98.28
926	92.63	93.79	98.56	99.52	96.11
927	93.18	97.54	95.27	99.21	96.39
928	81.22	83.90	95.95	96.91	89.52
929	92.46	94.99	96.99	99.02	95.98

930	88.85	91.74	96.23	98.20	93.93
931	88.60	91.91	95.75	97.71	93.79
932	93.39	95.47	97.60	98.71	96.53
933	91.12	94.13	96.30	98.84	95.21
934	94.01	96.79	96.87	99.67	96.83
935	94.07	97.28	96.47	99.30	96.87
936	75.10	76.96	96.79	97.40	85.74
937	93.37	94.83	98.31	98.87	96.54
938	92.79	96.47	95.88	98.48	96.17
939	93.72	96.98	96.39	99.06	96.68
940	95.81	98.08	97.57	99.53	97.83
941	92.97	93.98	98.79	99.36	96.32
942	93.88	96.59	96.94	99.42	96.76
943	91.62	95.19	95.76	99.05	95.47
944	92.25	92.85	99.27	99.51	95.96
945	86.07	89.98	94.14	99.50	92.01
946	92.44	94.02	98.02	99.64	95.98
947	86.68	88.92	96.47	99.59	92.54
948	81.23	82.63	97.68	98.53	89.53
949	89.31	95.61	92.61	99.36	94.09
950	96.76	97.31	99.39	99.77	98.34
951	97.34	98.08	99.20	99.82	98.64
952	93.81	95.79	97.67	99.76	96.72
953	93.56	94.69	98.63	99.55	96.62
954	91.79	95.31	95.84	99.10	95.57
955	88.85	95.05	92.62	98.91	93.82
956	86.35	93.50	90.87	99.46	92.17
957	91.26	98.03	92.76	99.55	95.32
958	96.23	97.37	98.76	99.71	98.06
959	79.13	81.89	96.37	95.78	88.54
960	90.08	91.95	97.53	99.07	94.66
961	90.35	91.49	98.47	99.42	94.85
962	93.39	97.54	95.52	98.91	96.52
963	87.15	89.66	96.64	97.81	93.02
964	72.88	74.24	96.79	98.41	84.03
965	91.04	95.67	94.54	99.63	95.10
966	93.07	95.44	97.22	99.07	96.32
967	94.47	96.09	98.16	99.26	97.11
968	70.28	72.25	96.67	96.73	82.70
969	94.14	98.13	95.74	99.50	96.92
970	93.97	95.73	97.95	99.46	96.83
971	94.66	96.46	97.98	99.14	97.22
972	93.68	97.93	95.47	99.03	96.68
973	91.58	93.95	97.02	99.42	95.46
974	96.35	98.53	97.70	99.67	98.11
975	95.02	96.14	98.74	99.34	97.42
976	95.07	96.50	98.40	99.27	97.44
977	89.60	91.85	97.03	98.88	94.37
978	82.70	85.41	95.07	99.18	89.98
979	73.02	74.29	97.70	98.10	84.40
980	86.09	88.02	97.03	98.98	92.31
981	86.26	87.02	98.79	99.55	92.53
982	96.92	97.64	99.21	99.83	98.42
983	89.13	90.50	98.16	99.02	94.18
984	86.55	87.15	99.16	99.41	92.77
985	87.60	88.93	97.96	99.57	93.22
986	89.18	90.25	98.58	99.08	94.23
987	90.67	95.98	93.93	98.71	94.94
988	87.33	90.20	95.97	98.46	93.00
989	92.03	95.00	96.41	99.56	95.70
990	93.38	94.74	98.34	99.72	96.51
991	92.74	94.85	97.46	99.22	96.14

992	88.21	92.77	94.08	98.65	93.42
993	82.13	83.51	97.46	99.17	89.94
994	87.51	88.81	98.08	99.25	93.22
995	96.29	98.00	98.15	99.89	98.08
996	94.58	95.70	98.69	99.76	97.17
997	80.17	82.45	96.77	96.83	89.04
998	92.97	95.11	97.43	99.45	96.26
999	94.26	97.14	96.78	99.77	96.96
1000	94.21	98.36	95.61	99.49	96.97
1001	95.60	96.67	98.79	99.69	97.72
1002	93.68	95.96	97.38	99.22	96.66
1003	92.06	95.00	96.48	99.11	95.73
1004	91.52	94.81	95.98	99.58	95.39
1005	94.80	96.27	98.31	99.73	97.28
1006	95.44	98.74	96.56	99.54	97.64
1007	94.25	98.15	95.84	99.55	96.98
1008	93.10	99.43	93.56	99.83	96.40
1009	80.55	82.49	96.44	98.51	88.92
1010	87.88	89.94	97.12	98.76	93.39
1011	91.25	93.00	97.71	99.57	95.29
1012	95.52	97.07	98.27	99.75	97.67
1013	92.05	93.39	98.26	99.78	95.76
1014	91.94	93.38	98.13	99.77	95.70
1015	94.53	95.49	98.87	99.65	97.15
1016	94.69	97.44	96.98	99.55	97.21
1017	93.31	94.22	98.91	99.43	96.51
1018	88.35	89.88	97.93	98.84	93.73
1019	88.01	90.25	96.81	98.99	93.42
1020	89.03	93.15	94.69	98.94	93.92
1021	93.76	94.52	99.11	99.50	96.76
1022	95.01	96.28	98.56	99.51	97.40
1023	96.34	97.27	98.96	99.82	98.11
1024	91.93	95.50	95.83	98.79	95.66
1025	83.88	85.88	97.05	97.96	91.13
1026	94.69	95.83	98.66	99.73	97.23
1027	95.71	96.83	98.73	99.88	97.77
1028	95.25	98.20	96.84	99.71	97.52
1029	92.13	95.46	96.07	99.30	95.76
1030	81.85	82.84	98.50	98.79	89.99
1031	83.30	93.95	86.87	99.01	90.27
1032	92.39	97.25	94.69	98.98	95.95
1033	95.05	97.05	97.77	99.54	97.41
1034	96.19	98.13	97.93	99.75	98.03
1035	91.38	93.36	97.49	99.21	95.38
1036	93.85	97.15	96.35	99.27	96.75
1037	94.18	96.27	97.60	99.57	96.93
1038	86.19	90.78	93.49	98.82	92.11
1039	92.44	93.80	98.33	99.32	96.01
1040	78.76	80.76	96.34	98.00	87.86
1041	87.43	89.75	96.61	99.05	93.06
1042	94.22	95.43	98.57	99.63	96.97
1043	94.95	96.27	98.50	99.46	97.37
1044	83.59	84.83	98.04	98.90	90.95
1045	95.05	96.73	98.11	99.41	97.42
1046	94.73	95.99	98.57	99.38	97.26
1047	92.76	97.54	94.77	99.73	96.13
1048	91.87	94.40	96.84	99.67	95.61
1049	94.37	94.92	99.35	99.80	97.08
1050	93.56	95.83	97.33	99.70	96.58
1051	91.25	92.60	98.19	99.76	95.32
1052	92.39	94.36	97.59	99.30	95.95
1053	94.42	96.70	97.44	99.34	97.07

1054	89.91	93.29	95.66	99.10	94.46
1055	82.50	88.32	90.78	99.06	89.53
1056	87.58	93.26	92.65	99.45	92.96
1057	95.74	97.83	97.75	99.30	97.79
1058	94.75	97.48	97.02	99.47	97.25
1059	93.62	98.90	94.61	98.87	96.71
1060	93.42	95.06	98.15	98.45	96.58
1061	96.86	98.78	98.01	99.29	98.39
1062	97.18	99.51	97.65	99.43	98.57
1063	98.94	99.75	99.18	99.80	99.47
1064	98.96	99.45	99.50	99.82	99.48
1065	97.52	98.84	98.62	99.62	98.73
1066	97.21	99.34	97.84	99.42	98.59
1067	97.17	99.17	97.96	99.39	98.56
1068	92.62	95.85	96.21	99.67	96.03
1069	94.36	97.19	96.90	98.78	97.05
1070	93.18	96.91	95.89	98.57	96.40
1071	93.31	96.43	96.48	98.82	96.46
1072	92.92	96.16	96.32	98.75	96.24
1073	93.74	95.14	98.39	98.99	96.74
1074	93.69	95.41	98.01	98.94	96.69
1075	95.33	97.56	97.61	98.81	97.58
1076	96.62	98.67	97.87	99.26	98.27
1077	97.85	98.49	99.32	99.80	98.91
1078	96.04	97.65	98.25	99.52	97.95
1079	93.61	94.59	98.80	99.70	96.65
1080	94.48	97.84	96.41	98.96	97.12
1081	93.01	96.88	95.75	98.33	96.31
1082	92.49	96.19	95.81	98.48	96.00
1083	88.85	92.37	95.57	97.48	93.94
1084	90.00	93.55	95.67	97.71	94.60
1085	78.16	79.65	97.07	98.58	87.50
1086	87.59	92.79	93.11	99.55	92.95
1087	94.54	96.64	97.61	99.80	97.12
1088	96.82	98.73	98.00	99.75	98.36
1089	95.58	96.60	98.84	99.66	97.71
1090	93.54	95.85	97.31	99.32	96.58
1091	88.47	89.33	98.76	99.50	93.81
1092	94.34	96.62	97.39	99.77	97.01
1093	94.41	97.49	96.63	99.51	97.06
1094	93.40	95.12	97.96	99.41	96.52
1095	93.99	95.44	98.28	99.60	96.84
1096	92.24	93.35	98.61	99.44	95.91
1097	93.94	94.69	99.09	99.70	96.84
1098	94.02	96.03	97.67	99.69	96.84
1099	95.02	96.83	97.98	99.33	97.40
1100	92.37	94.06	98.04	98.44	96.01
1101	92.33	95.41	96.38	99.02	95.89
1102	95.41	98.56	96.71	99.19	97.63
1103	96.48	98.90	97.50	99.53	98.19
1104	94.45	98.56	95.72	99.26	97.12
1105	95.53	97.91	97.47	98.99	97.69
1106	96.22	97.95	98.16	98.98	98.05
1107	97.30	98.71	98.53	99.30	98.62
1108	97.46	99.16	98.27	99.44	98.71
1109	98.00	98.83	99.14	99.58	98.99
1110	97.64	99.20	98.41	99.50	98.80
1111	97.10	98.74	98.29	99.47	98.52
1112	84.41	85.71	97.86	99.11	91.39
1113	97.24	98.63	98.55	99.44	98.59
1114	97.64	98.91	98.69	99.53	98.80
1115	94.27	97.97	96.07	98.79	97.01

1116	92.34	96.18	95.67	98.33	95.92
1117	92.87	96.80	95.64	98.72	96.22
1118	93.58	97.82	95.47	98.93	96.63
1119	96.50	98.94	97.47	99.54	98.20
1120	95.29	96.60	98.52	99.40	97.55
1121	93.89	97.00	96.59	98.71	96.79
1122	96.72	99.11	97.56	99.35	98.33
1123	91.09	92.64	97.92	99.74	95.21
1124	96.40	97.50	98.80	99.65	98.15
1125	93.04	95.51	97.11	99.10	96.31
1126	93.91	96.53	97.08	98.67	96.80
1127	91.23	95.44	95.20	97.44	95.32
1128	93.81	97.29	96.23	98.45	96.76
1129	93.17	98.04	94.86	98.79	96.42
1130	86.32	93.67	90.94	97.92	92.28
1131	76.59	78.13	96.66	98.59	86.41
1132	90.08	93.43	95.66	99.71	94.53
1133	90.66	92.36	97.77	99.27	94.99
1134	93.42	95.26	97.80	99.57	96.51
1135	93.63	95.71	97.59	99.14	96.64
1136	93.38	96.91	96.08	99.07	96.49
1137	93.78	95.65	97.81	99.49	96.72
1138	94.23	95.29	98.75	99.62	96.99
1139	94.71	95.72	98.82	99.62	97.25
1140	93.86	95.44	98.10	99.78	96.76
1141	93.30	96.80	96.08	99.33	96.44
1142	95.68	98.43	97.11	99.31	97.76
1143	96.23	99.03	97.12	99.31	98.07
1144	96.82	98.32	98.41	99.56	98.37
1145	94.48	97.12	97.05	99.70	97.09
1146	79.97	82.92	94.86	97.41	88.49
1147	92.71	96.47	95.76	99.05	96.11
1148	91.76	95.37	95.75	99.00	95.56
1149	91.58	96.02	94.86	99.37	95.44
1150	92.10	95.93	95.51	99.70	95.72
1151	95.90	96.52	99.31	99.75	97.89
1152	93.86	95.28	98.31	99.46	96.77
1153	93.24	95.73	97.07	99.60	96.39
1154	96.57	98.79	97.68	99.76	98.23
1155	94.61	97.47	96.85	99.68	97.16
1156	85.68	86.51	98.75	99.30	92.23
1157	95.79	97.26	98.38	99.54	97.82
1158	93.88	94.63	99.09	99.76	96.81
1159	94.91	95.57	99.23	99.83	97.36
1160	85.01	93.48	89.24	99.33	91.31
1161	95.14	97.77	97.15	99.57	97.46
1162	94.62	97.14	97.19	99.68	97.17
1163	94.15	98.67	95.30	99.34	96.96
1164	93.27	96.07	96.83	98.66	96.45
1165	94.01	98.07	95.69	99.05	96.87
1166	95.37	98.52	96.71	99.27	97.60
1167	95.00	96.08	98.77	99.42	97.41
1168	90.70	91.58	98.88	99.29	95.09
1169	81.56	83.01	97.40	98.88	89.63
1170	79.72	82.77	93.74	98.98	87.91
1171	81.71	96.14	83.64	99.42	89.45
1172	80.26	83.22	94.23	98.64	88.39
1173	91.34	93.73	97.04	98.97	95.35
1174	92.06	94.59	96.93	99.06	95.75
1175	89.56	92.70	95.80	99.68	94.22
1176	91.64	92.18	99.31	99.63	95.61
1177	90.07	94.64	94.39	99.43	94.52

1178	93.05	94.48	98.26	99.45	96.33
1179	93.50	95.24	97.92	99.65	96.56
1180	94.25	95.60	98.42	99.63	96.99
1181	85.70	87.03	97.82	99.42	92.11
1182	87.24	88.73	97.84	99.02	93.06
1183	89.96	93.02	96.00	99.37	94.49
1184	88.71	92.03	95.43	99.66	93.70
1185	93.78	94.13	99.60	99.72	96.79
1186	79.57	80.70	97.87	98.95	88.46
1187	87.94	88.58	99.06	99.59	93.53
1188	96.03	97.32	98.58	99.82	97.94
1189	94.64	95.25	99.26	99.82	97.22
1190	93.67	97.00	96.28	99.50	96.64
1191	92.56	93.96	98.24	99.69	96.05
1192	94.55	96.93	97.33	99.66	97.13
1193	90.89	92.50	97.95	99.04	95.15
1194	88.19	91.64	95.30	98.89	93.43
1195	92.56	94.17	98.03	99.28	96.06
1196	94.28	96.27	97.76	98.87	97.01
1197	93.51	96.58	96.56	98.77	96.57
1198	91.33	98.20	92.67	99.71	95.36
1199	95.11	95.94	99.03	99.71	97.46
1200	88.94	91.19	97.05	98.39	94.03
1201	92.70	95.56	96.67	98.90	96.11
1202	90.28	92.42	97.20	99.07	94.75
1203	93.45	97.43	95.61	99.71	96.51
1204	85.96	90.45	93.48	99.18	91.94
1205	81.45	82.75	97.79	98.75	89.64
1206	87.79	89.33	97.72	99.31	93.33
1207	90.81	92.62	97.59	99.57	95.04
1208	92.39	93.78	98.28	99.36	95.98
1209	87.36	88.88	97.90	98.73	93.17
1210	92.92	94.32	98.32	99.22	96.28
1211	93.74	96.19	97.18	99.58	96.68
1212	92.60	94.53	97.68	99.13	96.08
1213	89.15	94.55	93.33	99.70	93.94
1214	82.69	84.80	96.44	98.48	90.24
1215	78.82	82.93	92.29	97.42	87.36
1216	90.31	92.13	97.53	99.53	94.75
1217	95.27	96.41	98.70	99.86	97.54
1218	93.26	95.49	97.36	99.57	96.41
1219	86.61	89.27	95.98	99.13	92.50
1220	86.68	94.84	90.16	99.41	92.44
1221	92.64	95.36	96.77	99.46	96.06
1222	90.30	94.39	94.98	99.00	94.69
1223	95.41	97.84	97.37	99.82	97.60
1224	91.13	93.65	96.89	98.61	95.24
1225	89.54	92.12	96.81	97.74	94.41
1226	94.41	97.61	96.54	99.07	97.07
1227	96.11	96.67	99.37	99.73	98.00
1228	94.52	96.81	97.41	99.61	97.11
1229	91.77	93.25	98.16	99.15	95.64
1230	94.42	95.34	98.90	99.85	97.09
1231	93.97	96.73	96.89	99.51	96.81
1232	91.13	92.33	98.47	99.24	95.30
1233	83.59	84.65	98.31	99.03	90.97
1234	90.27	91.64	98.19	99.27	94.80
1235	92.87	94.58	97.90	99.67	96.21
1236	92.94	94.39	98.22	99.53	96.27
1237	89.14	90.21	98.57	99.15	94.21
1238	93.80	96.25	97.20	99.33	96.72
1239	93.07	95.15	97.54	99.17	96.33

1240	81.54	83.52	96.15	99.13	89.39
1241	77.38	78.66	97.53	98.59	87.08
1242	77.75	80.43	94.62	97.81	86.95
1243	84.62	86.03	97.57	99.42	91.43
1244	78.13	79.12	98.19	98.84	87.63
1245	89.62	90.41	98.94	99.46	94.48
1246	77.70	79.41	97.10	97.83	87.37
1247	84.66	92.02	90.14	98.75	91.07
1248	73.83	77.61	90.06	98.34	83.37
1249	85.31	86.54	98.15	98.96	91.98
1250	88.81	91.14	96.86	98.69	93.91
1251	89.61	91.60	97.46	98.39	94.44
1252	87.51	90.89	95.47	97.79	93.12
1253	94.10	95.68	98.21	98.82	96.93
1254	94.31	97.54	96.50	99.01	97.02
1255	94.39	96.36	97.76	99.26	97.06
1256	84.18	88.87	92.60	99.54	90.70
1257	95.31	97.50	97.61	99.44	97.56
1258	94.51	98.36	95.94	99.32	97.14
1259	91.50	93.41	97.66	98.72	95.49
1260	94.06	94.95	98.96	99.57	96.91
1261	94.07	95.10	98.78	99.49	96.91
1262	84.75	85.45	98.90	99.41	91.68
1263	92.66	94.38	97.86	99.73	96.09
1264	94.22	94.94	99.13	99.86	96.99
1265	92.34	94.36	97.47	99.76	95.89
1266	91.98	94.44	96.96	99.50	95.68
1267	84.67	85.88	97.89	99.47	91.49
1268	96.13	97.75	98.24	99.69	98.00
1269	91.76	94.68	96.43	99.49	95.54
1270	94.07	96.92	96.82	99.42	96.87
1271	83.24	85.32	96.66	98.30	90.64
1272	81.47	83.01	97.16	98.91	89.53
1273	80.58	83.40	94.80	98.21	88.73
1274	94.68	97.00	97.39	99.80	97.20
1275	90.81	91.70	98.83	99.54	95.13
1276	95.01	97.81	96.96	99.58	97.39
1277	84.98	87.62	96.16	97.77	91.69
1278	81.19	84.33	94.30	98.36	89.04
1279	89.34	89.63	99.65	99.60	94.38
1280	75.96	77.64	95.93	98.77	85.82
1281	85.47	86.45	98.54	99.13	92.10
1282	90.46	92.25	97.70	98.88	94.90
1283	88.17	91.97	94.99	98.19	93.45
1284	88.89	91.54	96.54	98.25	93.97
1285	90.43	93.29	96.38	98.81	94.81
1286	87.47	93.20	92.87	97.43	93.03
1287	91.32	94.73	95.94	98.36	95.33
1288	90.75	93.52	96.53	98.88	95.00
1289	94.08	96.37	97.41	99.03	96.89
1290	90.01	90.97	98.75	99.27	94.70
1291	92.99	95.28	97.34	98.67	96.30
1292	93.30	95.38	97.59	98.79	96.47
1293	86.95	90.11	95.26	99.64	92.61
1294	88.35	91.16	95.99	99.53	93.51
1295	88.18	95.64	91.23	99.69	93.38
1296	91.76	93.03	98.35	99.65	95.62
1297	91.63	94.10	96.88	99.70	95.47
1298	83.10	84.25	98.29	98.69	90.73
1299	93.02	94.03	98.75	99.63	96.33
1300	92.65	93.71	98.67	99.64	96.13
1301	93.17	96.66	96.07	99.33	96.36

1302	91.75	93.15	98.25	99.16	95.63
1303	90.80	92.34	98.00	99.20	95.08
1304	94.82	96.60	97.97	99.65	97.28
1305	94.39	94.87	99.43	99.78	97.10
1306	92.89	94.52	97.98	99.74	96.22
1307	94.90	97.02	97.64	99.55	97.33
1308	94.42	96.86	97.25	99.68	97.06
1309	93.35	95.90	97.02	99.53	96.46
1310	92.86	94.82	97.67	98.98	96.22
1311	89.94	93.39	95.57	99.21	94.47
1312	93.66	95.51	97.83	99.35	96.66
1313	92.97	94.67	97.91	99.66	96.27
1314	90.16	91.36	98.52	98.83	94.80
1315	87.99	90.05	97.38	97.87	93.57
1316	91.14	93.76	96.82	98.30	95.27
1317	90.64	93.14	96.90	98.41	94.98
1318	92.17	96.90	94.73	99.25	95.80
1319	90.01	93.19	96.03	98.24	94.59
1320	90.66	94.80	95.09	98.15	94.95
1321	92.89	94.30	98.31	99.21	96.26
1322	94.07	97.13	96.62	99.26	96.87
1323	92.53	95.51	96.46	99.68	95.98
1324	86.13	91.97	92.31	97.67	92.14
1325	89.00	91.94	96.13	98.54	93.99
1326	90.41	91.35	98.71	99.61	94.89
1327	92.13	95.93	95.56	99.67	95.74
1328	93.89	96.44	97.07	99.65	96.76
1329	91.40	92.03	99.20	99.57	95.48
1330	94.50	97.68	96.51	99.87	97.10
1331	93.02	93.80	99.02	99.81	96.34
1332	88.21	89.43	98.18	99.51	93.60
1333	87.01	89.58	96.17	99.13	92.76
1334	92.78	94.03	98.47	99.41	96.20
1335	88.93	91.53	96.42	99.21	93.91
1336	93.94	94.77	99.01	99.56	96.84
1337	95.61	97.43	98.01	99.61	97.72
1338	96.03	98.57	97.32	99.86	97.94
1339	97.09	98.97	98.05	99.74	98.51
1340	96.36	98.95	97.32	99.57	98.13
1341	93.08	94.48	98.32	99.29	96.36
1342	93.07	95.07	97.64	99.02	96.34
1343	86.21	88.35	97.27	97.38	92.60
1344	94.05	98.46	95.39	99.06	96.90
1345	81.41	83.52	96.45	98.08	89.52
1346	92.52	96.14	95.88	98.83	96.01
1347	93.05	94.64	98.14	98.91	96.36
1348	83.38	86.30	95.96	96.60	90.87
1349	83.51	87.00	95.11	96.30	90.87
1350	91.27	94.04	96.62	98.63	95.31
1351	90.00	93.69	95.42	98.44	94.54
1352	88.52	90.97	96.84	97.96	93.81
1353	88.55	91.52	95.97	98.81	93.69
1354	88.64	97.18	90.55	99.72	93.75
1355	91.26	93.98	96.56	99.66	95.25
1356	94.05	94.48	99.48	99.84	96.92
1357	91.66	94.32	96.71	99.44	95.50
1358	89.51	90.45	98.74	99.33	94.41
1359	93.91	94.85	98.85	99.79	96.81
1360	92.79	96.40	95.85	99.77	96.12
1361	93.12	93.83	99.14	99.56	96.41
1362	93.83	96.33	97.12	99.58	96.73
1363	89.48	90.52	98.60	99.28	94.39

1364	92.97	94.57	98.01	99.78	96.26
1365	92.99	93.53	99.32	99.77	96.34
1366	95.05	98.67	96.22	99.50	97.43
1367	89.12	94.00	93.94	98.81	93.97
1368	96.32	97.67	98.52	99.72	98.10
1369	97.14	98.25	98.82	99.87	98.53
1370	96.45	97.28	99.09	99.60	98.18
1371	96.09	97.91	98.04	99.52	97.98
1372	91.95	93.22	98.45	99.05	95.76
1373	88.72	90.88	97.22	98.05	93.95
1374	88.89	90.69	97.75	98.15	94.09
1375	93.75	97.59	95.81	99.53	96.69
1376	91.88	93.53	97.94	99.23	95.68
1377	93.13	96.39	96.29	99.20	96.34
1378	92.08	96.67	94.88	98.82	95.76
1379	94.82	96.23	98.37	99.80	97.29
1380	88.75	90.96	97.07	98.42	93.92
1381	94.59	97.22	97.09	99.44	97.16
1382	90.22	92.73	96.73	98.99	94.69
1383	91.51	97.35	93.66	98.88	95.47
1384	89.97	93.96	95.05	98.77	94.50
1385	90.66	91.85	98.37	99.66	95.00
1386	94.56	96.53	97.75	99.72	97.14
1387	93.75	97.20	96.17	99.67	96.68
1388	94.08	94.80	99.15	99.54	96.93
1389	94.73	97.47	97.00	99.62	97.23
1390	95.32	98.11	97.02	99.53	97.56
1391	89.14	92.79	95.14	99.59	93.95
1392	93.38	93.99	99.26	99.68	96.55
1393	94.67	96.00	98.46	99.71	97.21
1394	93.36	95.50	97.45	99.72	96.47
1395	92.75	93.17	99.48	99.79	96.22
1396	88.64	91.92	95.47	99.66	93.66
1397	97.45	97.91	99.50	99.94	98.70
1398	88.08	91.04	95.82	99.23	93.37
1399	93.76	96.30	97.09	99.39	96.69
1400	93.72	98.61	94.88	99.61	96.71
1401	95.87	98.29	97.41	99.84	97.85
1402	93.89	95.12	98.52	99.76	96.79
1403	95.73	97.75	97.81	99.47	97.78
1404	92.88	95.38	97.06	99.16	96.21
1405	89.88	95.77	93.15	99.09	94.45
1406	87.63	90.76	95.70	98.29	93.17
1407	89.65	91.48	97.50	99.29	94.39
1408	89.83	99.25	90.37	99.73	94.60
1409	94.42	95.45	98.81	99.55	97.10
1410	94.05	94.74	99.19	99.58	96.92
1411	92.30	93.93	97.92	99.77	95.88
1412	92.27	93.73	98.17	99.51	95.89
1413	82.30	96.87	84.22	98.25	90.10
1414	85.09	89.01	94.29	97.73	91.58
1415	95.59	97.74	97.65	99.84	97.69
1416	94.32	95.22	98.93	99.75	97.04
1417	93.37	94.42	98.69	99.80	96.51
1418	93.66	95.70	97.58	99.79	96.63
1419	93.84	95.58	97.92	99.77	96.74
1420	94.76	95.77	98.84	99.44	97.28
1421	95.83	97.79	97.88	99.44	97.84
1422	93.59	94.86	98.49	99.33	96.64
1423	85.97	86.73	98.86	99.34	92.40
1424	92.53	93.22	99.15	99.53	96.09
1425	92.19	93.56	98.26	99.58	95.85

1426	93.07	93.54	99.42	99.73	96.39
1427	84.04	85.19	97.98	99.42	91.14
1428	87.92	88.46	99.21	99.61	93.53
1429	94.67	97.26	97.12	99.79	97.19
1430	94.94	97.08	97.61	99.66	97.34
1431	92.46	93.58	98.58	99.60	96.02
1432	97.22	99.17	97.99	99.84	98.58
1433	96.82	97.41	99.36	99.69	98.38
1434	87.67	90.77	95.54	99.36	93.09
1435	95.19	96.12	98.94	99.64	97.51
1436	92.64	94.67	97.51	99.63	96.07
1437	89.86	93.98	94.81	99.49	94.39
1438	91.97	94.06	97.41	99.36	95.70
1439	92.51	93.61	98.63	99.48	96.06
1440	90.86	92.05	98.45	99.34	95.14
1441	88.29	88.76	99.37	99.56	93.77
1442	84.41	85.98	97.33	99.20	91.31
1443	88.09	90.26	96.92	98.99	93.47
1444	82.62	93.39	86.48	98.85	89.80
1445	96.39	97.54	98.75	99.66	98.14
1446	96.30	97.42	98.77	99.66	98.09
1447	96.34	98.12	98.09	99.73	98.10
1448	94.97	95.98	98.83	99.66	97.38
1449	95.05	95.63	99.33	99.75	97.45
1450	95.56	96.41	99.03	99.79	97.70
1451	94.11	95.75	98.10	99.34	96.91
1452	96.74	97.38	99.30	99.78	98.33
1453	92.76	94.18	98.28	99.25	96.19
1454	92.50	95.74	96.18	99.72	95.96
1455	88.87	90.51	97.63	99.49	93.94
1456	92.22	93.04	98.96	99.63	95.91
1457	93.88	94.80	98.92	99.43	96.82
1458	93.53	94.74	98.53	99.65	96.60
1459	89.93	91.31	98.24	98.84	94.65
1460	83.31	84.59	97.87	98.97	90.75
1461	89.74	90.79	98.52	99.56	94.50
1462	93.62	95.15	98.19	99.38	96.65
1463	92.99	97.01	95.53	99.36	96.27
1464	93.25	97.19	95.68	98.97	96.43
1465	93.63	94.80	98.64	99.21	96.68
1466	92.50	95.58	96.36	99.46	95.97
1467	97.72	98.56	99.12	99.89	98.84
1468	94.79	98.60	95.99	99.80	97.28
1469	95.94	98.94	96.91	99.38	97.92
1470	95.38	97.87	97.34	99.02	97.60
1471	95.51	98.57	96.81	99.26	97.68
1472	94.33	98.71	95.43	99.60	97.04
1473	92.90	94.44	98.12	99.46	96.24
1474	84.86	85.69	98.77	99.16	91.77
1475	86.33	87.33	98.50	99.28	92.58
1476	94.08	94.36	99.67	99.84	96.94
1477	91.76	92.22	99.44	99.61	95.69
1478	95.21	96.71	98.30	99.87	97.50
1479	94.63	95.89	98.55	99.48	97.20
1480	95.36	97.36	97.82	99.26	97.59
1481	95.84	98.37	97.32	99.44	97.85
1482	89.25	92.13	96.25	98.48	94.14
1483	93.70	96.69	96.65	99.20	96.67
1484	95.86	98.54	97.18	99.51	97.86
1485	95.11	98.44	96.51	99.29	97.46
1486	95.92	97.88	97.90	99.25	97.89
1487	90.19	90.76	99.27	99.55	94.82

1488	95.05	97.07	97.74	99.79	97.40
1489	80.26	82.77	94.72	99.32	88.34
1490	85.62	87.58	96.74	99.56	91.93
1491	89.89	90.96	98.51	99.53	94.59
1492	97.84	98.96	98.83	99.82	98.90
1493	96.41	98.30	97.99	99.45	98.15
1494	93.44	98.40	94.78	99.45	96.56
1495	96.18	97.43	98.62	99.72	98.03
1496	95.34	97.89	97.25	99.69	97.57
1497	95.82	98.00	97.66	99.54	97.83
1498	96.31	98.23	97.95	99.58	98.09
1499	96.24	97.89	98.22	99.74	98.05
1500	89.62	93.37	95.13	99.68	94.24
1501	94.73	98.06	96.44	99.48	97.24
1502	96.24	98.99	97.15	99.73	98.06
1503	85.93	88.77	95.51	99.48	92.02
1504	86.77	92.19	92.63	99.57	92.41
1505	93.23	98.32	94.59	99.84	96.42
1506	96.74	97.67	98.99	99.69	98.32
1507	96.92	98.98	97.88	99.49	98.43
1508	96.79	99.01	97.71	99.46	98.36
1509	96.20	97.20	98.89	99.86	98.04
1510	96.34	97.08	99.18	99.74	98.12
1511	96.58	97.87	98.61	99.67	98.24
1512	96.23	98.57	97.54	99.64	98.05
1513	96.07	97.78	98.14	99.67	97.96
1514	91.02	91.52	99.34	99.72	95.27
1515	94.47	94.89	99.51	99.76	97.14
1516	95.92	98.06	97.70	99.69	97.88
1517	96.65	98.02	98.52	99.92	98.27
1518	97.57	99.66	97.89	99.91	98.77
1519	90.19	93.29	95.97	99.59	94.61
1520	89.53	91.67	97.04	99.56	94.28
1521	92.80	98.18	94.26	99.62	96.18
1522	95.58	97.38	98.02	99.41	97.70
1523	97.15	99.45	97.66	99.77	98.55
1524	96.16	97.50	98.52	99.79	98.01
1525	96.98	97.96	98.94	99.68	98.45
1526	96.31	97.06	99.17	99.75	98.10
1527	92.79	93.74	98.81	99.60	96.21
1528	95.90	97.88	97.86	99.76	97.87
1529	93.77	95.16	98.34	99.60	96.72
1530	91.51	91.80	99.63	99.75	95.56
1531	89.28	89.89	99.15	99.65	94.29
1532	88.02	88.98	98.54	99.62	93.52
1533	92.79	95.39	96.88	99.78	96.13
1534	95.79	97.58	98.03	99.73	97.81
1535	82.34	83.79	97.98	98.07	90.33
1536	94.45	95.25	99.07	99.49	97.12
1537	94.90	96.47	98.20	99.77	97.33
1538	89.92	90.50	99.20	99.64	94.65
1539	95.51	97.49	97.83	99.73	97.66
1540	93.75	95.19	98.29	99.54	96.71
1541	94.50	95.43	98.90	99.67	97.13
1542	81.30	82.00	98.98	99.03	89.69
1543	91.19	92.09	98.80	99.64	95.33
1544	97.18	98.06	99.06	99.86	98.56
1545	95.51	97.86	97.47	99.51	97.66
1546	94.54	97.82	96.46	99.49	97.13
1547	95.70	97.86	97.66	99.70	97.76
1548	95.88	97.49	98.23	99.74	97.86
1549	90.46	91.54	98.50	99.68	94.89

1550	93.33	93.68	99.57	99.79	96.53
1551	89.21	89.91	99.00	99.60	94.24
1552	87.44	88.56	98.35	99.29	93.20
1553	93.84	94.36	99.38	99.66	96.80
1554	92.82	93.06	99.74	99.61	96.29
1555	85.01	86.05	98.33	99.29	91.78
1556	89.41	95.25	93.02	99.52	94.12
1557	93.96	94.77	99.03	99.71	96.85
1558	95.89	96.43	99.38	99.84	97.88
1559	92.80	94.74	97.62	99.65	96.16
1560	94.67	96.74	97.67	99.56	97.20
1561	94.53	96.11	98.19	99.55	97.13
1562	95.16	97.64	97.30	99.59	97.47
1563	84.57	85.87	97.91	99.04	91.50
1564	96.46	97.58	98.76	99.86	98.17
1565	96.14	98.43	97.58	99.60	98.00
1566	93.28	94.88	98.09	99.21	96.46
1567	94.78	96.24	98.33	99.52	97.28
1568	96.72	97.96	98.66	99.69	98.31
1569	92.37	93.19	98.95	99.71	95.98
1570	92.48	93.78	98.35	99.63	96.01
1571	91.94	93.32	98.23	99.60	95.71
1572	96.34	97.99	98.23	99.74	98.11
1573	91.67	92.28	99.22	99.60	95.63
1574	88.61	89.76	98.35	99.36	93.86
1575	87.41	89.51	96.87	99.28	93.04
1576	87.24	90.63	95.08	99.36	92.80
1577	89.90	90.85	98.71	99.50	94.62
1578	96.54	97.57	98.87	99.70	98.22
1579	96.54	98.13	98.31	99.49	98.22
1580	96.63	97.70	98.84	99.65	98.27
1581	92.88	93.79	98.92	99.23	96.29
1582	90.72	94.03	95.89	99.04	94.95
1583	92.58	94.11	98.09	99.55	96.06
1584	95.61	96.66	98.80	99.81	97.72
1585	89.30	90.57	98.20	99.45	94.23
1586	87.56	88.85	98.08	99.31	93.24
1587	85.33	86.88	97.54	99.09	91.90
1588	86.53	97.13	88.36	99.32	92.54
1589	93.37	94.30	98.87	99.65	96.53
1590	95.86	96.77	98.97	99.89	97.86
1591	94.91	95.76	99.01	99.67	97.36
1592	93.77	94.87	98.73	99.13	96.76
1593	92.79	94.13	98.42	98.98	96.23
1594	92.81	97.74	94.64	99.69	96.17
1595	94.74	97.01	97.48	99.24	97.24
1596	95.87	96.85	98.91	99.66	97.87
1597	93.76	99.04	94.60	99.20	96.77
1598	87.44	91.85	94.02	98.87	92.92
1599	93.11	93.71	99.25	99.74	96.40
1600	95.64	97.54	97.91	99.75	97.73
1601	95.18	99.01	96.05	99.47	97.51
1602	96.01	98.78	97.12	99.49	97.94
1603	95.51	97.03	98.31	99.54	97.67
1604	95.31	96.82	98.31	99.59	97.56
1605	94.47	98.55	95.73	99.33	97.12
1606	97.42	98.77	98.57	99.92	98.67
1607	94.49	97.19	96.98	99.81	97.09
1608	95.32	96.59	98.57	99.54	97.57
1609	95.82	97.09	98.60	99.31	97.84
1610	88.70	90.37	97.96	98.02	94.01
1611	70.39	73.51	94.66	95.00	82.76

1612	77.01	79.07	95.89	97.96	86.67
1613	91.58	94.86	96.00	99.62	95.43
1614	96.18	98.34	97.70	99.75	98.02
1615	94.45	98.22	95.99	99.59	97.09
1616	91.74	92.64	98.85	99.54	95.64
1617	97.55	99.18	98.32	99.93	98.75
1618	91.94	92.51	99.32	99.44	95.79
1619	93.54	94.32	99.04	99.76	96.62
1620	90.62	93.21	96.61	99.69	94.88
1621	94.36	95.69	98.47	99.38	97.06
1622	94.81	96.52	98.10	99.00	97.30
1623	94.63	96.05	98.41	98.94	97.22
1624	92.21	97.53	94.23	99.18	95.85
1625	95.49	98.96	96.42	99.59	97.67
1626	95.30	98.04	97.06	99.41	97.55
1627	93.89	98.21	95.42	99.34	96.79
1628	97.04	98.50	98.45	99.92	98.47
1629	96.82	98.72	98.02	99.54	98.37
1630	96.92	98.80	98.05	99.52	98.42
1631	94.14	99.40	94.68	99.41	96.98
1632	94.12	98.80	95.12	99.71	96.93
1633	95.70	97.14	98.41	99.62	97.77
1634	94.97	96.41	98.39	99.24	97.39
1635	91.23	95.17	95.36	98.59	95.26
1636	98.17	99.14	99.01	99.82	99.07
1637	96.76	99.33	97.39	99.48	98.35
1638	93.85	96.22	97.29	99.28	96.75
1639	95.30	95.80	99.42	99.81	97.58
1640	93.40	97.26	95.72	99.70	96.48
1641	89.41	89.96	99.22	99.68	94.36
1642	89.82	91.28	97.95	99.59	94.50
1643	90.71	92.86	97.20	99.32	94.98
1644	94.17	95.78	98.16	99.20	96.95
1645	92.76	98.18	94.25	99.33	96.18
1646	93.37	94.69	98.38	99.72	96.50
1647	93.46	93.97	99.37	99.79	96.59
1648	96.47	98.97	97.41	99.89	98.18
1649	94.54	98.89	95.49	99.62	97.16
1650	94.87	95.53	99.21	99.81	97.34
1651	93.77	95.17	98.34	99.51	96.73
1652	94.27	96.01	97.97	99.66	96.98
1653	89.82	90.25	99.48	99.43	94.64
1654	93.47	96.22	96.84	99.51	96.53
1655	95.69	97.63	97.89	99.48	97.76
1656	96.69	98.24	98.35	99.53	98.29
1657	93.00	94.39	98.38	98.90	96.34
1658	92.15	94.47	97.18	99.13	95.80
1659	92.06	93.76	97.96	98.76	95.82
1660	86.95	90.76	94.88	97.46	92.78
1661	94.60	95.87	98.54	99.57	97.18
1662	96.42	98.69	97.63	99.51	98.16
1663	90.51	93.21	96.65	98.40	94.90
1664	83.53	85.41	97.07	98.31	90.87
1665	92.94	93.77	99.01	99.32	96.32
1666	93.85	96.65	96.88	98.84	96.76
1667	95.63	98.35	97.13	99.15	97.74
1668	62.70	66.24	81.53	97.35	73.09
1669	94.37	96.16	97.93	99.77	97.03
1670	97.50	98.97	98.47	99.78	98.72
1671	97.32	97.86	99.41	99.86	98.63
1672	94.79	96.69	97.85	99.66	97.26
1673	92.11	93.77	97.91	99.50	95.79

1674	95.58	97.13	98.30	99.24	97.71
1675	96.69	98.84	97.79	99.19	98.31
1676	93.79	97.67	95.84	98.82	96.74
1677	83.15	85.13	96.94	98.08	90.65
1678	94.78	96.15	98.42	99.68	97.27
1679	85.73	92.37	91.11	99.46	91.73
1680	88.28	90.91	96.27	99.30	93.51
1681	92.02	93.26	98.43	99.52	95.77
1682	97.40	99.21	98.14	99.84	98.67
1683	97.96	99.02	98.89	99.95	98.96
1684	97.41	98.41	98.93	99.77	98.67
1685	96.27	98.78	97.39	99.73	98.08
1686	72.13	75.61	90.44	97.62	82.36
1687	92.98	96.23	96.23	99.75	96.23
1688	95.31	98.27	96.86	99.34	97.56
1689	93.74	95.89	97.54	98.91	96.71
1690	92.60	94.36	97.90	98.83	96.10
1691	94.00	95.60	98.17	99.03	96.87
1692	93.51	95.08	98.21	98.76	96.62
1693	95.98	98.53	97.33	99.36	97.92
1694	97.43	98.82	98.54	99.89	98.68
1695	97.19	98.59	98.52	99.71	98.56
1696	89.12	91.41	96.94	98.67	94.10
1697	90.50	97.66	92.38	98.51	94.95
1698	74.38	77.61	95.45	94.78	85.61
1699	89.06	90.36	98.12	99.61	94.08
1700	92.90	94.84	97.63	99.71	96.22
1701	95.65	97.48	97.99	99.69	97.73
1702	94.79	96.44	98.14	99.33	97.28
1703	63.81	69.80	75.36	98.39	72.47
1704	96.39	99.28	97.04	99.68	98.15
1705	95.50	97.29	98.03	99.53	97.66
1706	96.19	97.26	98.82	99.68	98.03
1707	92.49	95.26	96.67	99.80	95.96
1708	91.66	93.47	97.65	99.76	95.51
1709	97.86	99.51	98.32	99.90	98.91
1710	97.57	99.26	98.27	99.70	98.76
1711	93.87	95.60	98.04	98.80	96.80
1712	89.29	91.52	97.19	97.99	94.27
1713	82.96	85.12	96.70	97.87	90.54
1714	93.10	93.60	99.42	99.55	96.42
1715	85.90	91.54	92.14	99.46	91.84
1716	88.52	94.35	92.80	99.42	93.57
1717	94.80	96.95	97.57	99.78	97.26
1718	91.65	93.02	98.22	99.54	95.55
1719	95.03	99.15	95.75	99.83	97.42
1720	96.65	97.30	99.28	99.92	98.28
1721	96.16	97.61	98.43	99.58	98.02
1722	93.46	95.95	97.15	98.85	96.55
1723	93.04	98.37	94.45	98.79	96.37
1724	91.11	93.28	97.30	98.73	95.25
1725	95.48	96.62	98.73	99.35	97.67
1726	95.92	97.75	98.03	99.16	97.89
1727	94.03	96.87	96.86	98.93	96.86
1728	92.57	95.97	96.02	99.79	95.99
1729	94.84	96.35	98.30	99.26	97.32
1730	94.87	97.65	97.00	99.09	97.32
1731	84.11	87.11	96.22	96.00	91.44
1732	91.99	93.90	97.68	98.88	95.75
1733	92.15	93.87	97.85	99.45	95.81
1734	93.69	95.96	97.38	99.31	96.67
1735	92.94	95.33	97.19	99.13	96.25

1736	92.88	94.88	97.63	98.95	96.24
1737	90.73	93.85	96.02	99.61	94.93
1738	95.47	97.46	97.82	99.70	97.64
1739	92.83	93.28	99.44	99.78	96.26
1740	96.94	98.31	98.55	99.57	98.43
1741	95.79	98.08	97.55	99.50	97.81
1742	90.71	91.84	98.44	99.75	95.02
1743	85.36	86.13	98.81	99.35	92.04
1744	93.56	96.97	96.19	99.53	96.58
1745	92.02	94.08	97.50	98.81	95.76
1746	90.65	91.77	98.66	98.78	95.09
1747	96.37	96.77	99.55	99.91	98.14
1748	95.41	97.67	97.54	99.40	97.61
1749	96.15	97.99	98.04	99.33	98.01
1750	89.28	90.77	97.85	99.69	94.18
1751	95.26	97.94	97.12	99.65	97.52
1752	96.36	98.48	97.76	99.72	98.12
1753	96.30	97.71	98.47	99.46	98.09
1754	95.44	97.83	97.42	99.48	97.63
1755	90.94	92.31	98.24	99.16	95.18
1756	90.22	92.19	97.48	98.69	94.76
1757	94.69	97.32	97.12	99.30	97.22
1758	89.95	92.05	97.20	99.20	94.55
1759	85.06	86.84	97.26	98.69	91.76
1760	91.30	94.47	96.19	98.49	95.32
1761	95.63	98.03	97.41	99.90	97.72
1762	92.54	94.12	98.17	98.52	96.10
1763	80.87	83.40	96.61	96.36	89.52
1764	82.38	83.70	97.92	98.59	90.25
1765	88.93	91.76	96.11	99.32	93.89
1766	96.13	99.16	96.88	99.82	98.01
1767	95.48	97.78	97.51	99.61	97.64
1768	94.76	98.04	96.50	99.35	97.26
1769	98.11	99.66	98.44	99.76	99.05
1770	97.78	98.47	99.27	99.80	98.87
1771	95.58	96.41	99.05	99.76	97.71
1772	84.51	86.45	96.89	98.74	91.38
1773	94.86	98.20	96.42	99.86	97.30
1774	93.39	94.07	99.17	99.69	96.55
1775	93.52	95.03	98.19	99.61	96.58
1776	95.27	99.19	95.99	99.50	97.57
1777	92.95	94.66	97.99	98.78	96.30
1778	93.16	95.22	97.60	98.78	96.40
1779	94.87	98.84	95.89	99.42	97.34
1780	93.50	94.08	99.32	99.56	96.63
1781	95.23	95.68	99.47	99.88	97.54
1782	95.34	95.75	99.53	99.80	97.60
1783	90.60	96.23	93.57	99.21	94.88
1784	95.30	97.74	97.33	99.83	97.54
1785	89.61	93.79	94.69	99.49	94.24
1786	90.00	90.76	98.99	99.47	94.70
1787	88.94	96.61	91.38	99.29	93.92
1788	93.86	96.91	96.60	99.22	96.75
1789	96.26	97.23	98.93	99.71	98.07
1790	75.58	76.70	97.78	98.61	85.97
1791	87.55	90.42	95.85	99.13	93.06
1792	92.01	94.63	96.87	98.70	95.74
1793	83.89	93.71	88.23	96.69	90.89
1794	94.24	98.88	95.21	99.31	97.01
1795	93.41	96.65	96.35	99.40	96.50
1796	86.18	88.43	96.47	99.25	92.27
1797	86.38	89.06	95.98	98.89	92.39

1798	84.99	86.64	97.24	99.26	91.64
1799	93.35	96.35	96.57	99.30	96.46
1800	95.48	97.97	97.33	99.48	97.65
1801	95.68	97.33	98.17	99.72	97.75
1802	97.29	99.41	97.83	99.89	98.62
1803	96.03	96.97	98.94	99.87	97.95
1804	95.26	95.59	99.62	99.81	97.56
1805	95.27	98.27	96.82	99.49	97.54
1806	97.07	98.83	98.16	99.66	98.50
1807	95.66	96.02	99.60	99.71	97.78
1808	97.07	97.96	99.04	99.83	98.50
1809	91.69	93.33	97.86	99.75	95.55
1810	95.15	97.36	97.55	99.85	97.45
1811	95.63	97.00	98.48	99.59	97.73
1812	94.22	97.27	96.65	99.13	96.96
1813	87.68	92.88	93.45	97.51	93.16
1814	90.91	94.30	95.86	98.75	95.07
1815	93.33	93.72	99.53	99.73	96.54
1816	93.98	94.67	99.19	99.53	96.88
1817	95.27	96.20	98.91	99.87	97.54
1818	95.54	96.40	99.04	99.56	97.70
1819	94.16	95.83	98.07	99.40	96.94
1820	91.17	92.66	98.07	99.40	95.29
1821	94.84	97.04	97.53	99.78	97.28
1822	93.83	95.83	97.65	99.77	96.73
1823	89.14	91.68	96.47	99.54	94.01
1824	91.07	92.52	98.08	99.60	95.22
1825	89.09	92.41	95.64	98.78	94.00
1826	93.83	96.37	97.14	98.82	96.76
1827	89.70	95.25	93.49	98.46	94.36
1828	95.70	96.64	98.94	99.64	97.78
1829	87.87	92.62	93.74	98.93	93.18
1830	91.50	95.94	94.88	98.93	95.41
1831	69.87	76.82	89.72	88.98	82.77
1832	90.81	95.41	94.75	97.27	95.08
1833	89.95	92.84	96.54	97.22	94.66
1834	83.27	89.53	90.53	99.36	90.03
1835	86.32	87.80	97.62	99.42	92.45
1836	92.61	93.52	98.93	99.19	96.15
1837	95.06	96.32	98.58	99.32	97.44
1838	94.14	94.80	99.22	99.64	96.96
1839	96.48	97.86	98.51	99.68	98.18
1840	85.33	86.00	99.07	99.21	92.07
1841	91.42	96.51	94.22	99.45	95.35
1842	94.84	95.98	98.69	99.49	97.32
1843	96.12	97.03	98.99	99.74	98.00
1844	94.68	96.30	98.14	99.62	97.21
1845	93.50	95.56	97.64	98.76	96.58
1846	94.67	97.04	97.42	98.43	97.23
1847	96.30	97.84	98.36	98.87	98.10
1848	97.70	99.32	98.35	99.46	98.83
1849	98.20	99.25	98.93	99.59	99.09
1850	93.74	96.23	97.10	99.85	96.67
1851	96.04	97.90	98.03	98.74	97.97
1852	95.73	98.60	97.06	98.58	97.82
1853	96.73	98.34	98.30	99.10	98.32
1854	97.58	99.06	98.47	99.47	98.77
1855	99.06	99.38	99.68	99.80	99.53
1856	98.18	99.19	98.96	99.58	99.08
1857	98.16	99.29	98.85	99.64	99.07
1858	95.52	96.52	98.91	99.16	97.70
1859	98.09	98.77	99.29	99.63	99.03

1860	97.00	97.81	99.14	99.38	98.47
1861	91.17	91.61	99.42	99.71	95.36
1862	95.61	98.81	96.70	99.18	97.74
1863	93.07	94.81	97.99	98.68	96.37
1864	95.16	96.77	98.23	99.04	97.49
1865	95.89	97.19	98.59	99.19	97.88
1866	95.62	97.42	98.04	99.19	97.73
1867	95.47	97.70	97.59	99.03	97.65
1868	95.92	97.85	97.94	99.04	97.89
1869	94.85	98.62	96.10	98.89	97.34
1870	93.34	96.57	96.40	98.46	96.49
1871	93.75	96.11	97.35	98.47	96.72
1872	91.41	96.61	94.09	99.76	95.33
1873	94.68	97.46	97.00	98.63	97.23
1874	91.21	97.43	93.45	97.59	95.40
1875	90.62	94.19	95.85	96.82	95.01
1876	91.30	92.09	98.99	99.48	95.42
1877	94.44	95.63	98.63	99.36	97.11
1878	95.76	98.74	96.91	99.32	97.82
1879	96.68	99.03	97.58	99.66	98.30
1880	95.34	97.92	97.21	99.84	97.56
1881	90.35	91.04	99.15	99.26	94.92
1882	94.32	95.42	98.70	99.61	97.03
1883	88.78	89.95	98.28	99.59	93.93
1884	93.30	95.13	97.80	99.60	96.45
1885	94.44	94.96	99.38	99.81	97.12
1886	90.25	92.18	97.53	98.73	94.78
1887	96.74	98.95	97.73	99.30	98.34
1888	96.80	98.20	98.53	99.12	98.37
1889	97.39	98.79	98.54	99.33	98.67
1890	98.09	99.51	98.56	99.57	99.03
1891	97.32	99.12	98.16	99.25	98.64
1892	93.58	97.35	95.96	98.03	96.65
1893	96.88	98.43	98.39	99.11	98.41
1894	86.37	94.03	90.42	99.58	92.19
1895	97.52	98.85	98.62	99.41	98.74
1896	98.26	99.03	99.20	99.62	99.12
1897	97.22	98.36	98.80	99.31	98.58
1898	97.44	99.15	98.25	99.47	98.70
1899	97.61	99.49	98.10	99.49	98.79
1900	97.89	99.21	98.65	99.51	98.93
1901	96.62	98.82	97.73	99.26	98.27
1902	95.86	98.68	97.07	99.15	97.87
1903	95.01	98.08	96.74	99.13	97.40
1904	97.04	99.00	97.98	99.49	98.48
1905	89.67	95.88	92.75	99.71	94.29
1906	97.63	98.97	98.61	99.62	98.79
1907	97.67	99.70	97.97	99.66	98.82
1908	96.63	99.00	97.56	99.35	98.28
1909	96.73	99.48	97.23	99.44	98.34
1910	97.13	99.46	97.64	99.63	98.54
1911	96.05	98.65	97.30	99.21	97.97
1912	95.14	98.11	96.86	98.82	97.48
1913	95.18	96.82	98.20	98.88	97.50
1914	95.10	98.36	96.60	98.78	97.47
1915	94.09	98.60	95.36	98.66	96.95
1916	91.53	93.35	97.71	99.15	95.48
1917	94.99	96.27	98.55	99.44	97.40
1918	96.42	97.04	99.31	99.84	98.16
1919	92.21	95.05	96.60	99.21	95.82
1920	94.98	96.44	98.37	99.14	97.39
1921	92.94	95.34	97.19	98.95	96.26

1922	90.45	92.58	97.14	99.66	94.81
1923	93.86	96.18	97.31	99.78	96.74
1924	95.99	97.48	98.37	99.76	97.92
1925	94.32	95.50	98.59	99.85	97.02
1926	88.83	90.42	97.67	99.57	93.91
1927	89.56	91.02	97.94	99.57	94.35
1928	94.72	97.74	96.73	99.38	97.23
1929	83.99	84.91	98.61	99.04	91.25
1930	93.62	97.28	95.95	99.69	96.61
1931	93.10	95.47	97.18	99.66	96.32
1932	84.31	86.44	96.82	98.08	91.34
1933	94.80	96.49	98.08	99.44	97.28
1934	94.80	99.15	95.53	99.71	97.31
1935	93.86	96.94	96.54	99.73	96.74
1936	93.91	95.31	98.34	99.58	96.80
1937	88.79	91.91	95.70	99.54	93.77
1938	95.80	99.02	96.68	99.62	97.83
1939	95.00	96.38	98.42	99.68	97.39
1940	97.04	98.55	98.41	99.73	98.48
1941	96.01	97.30	98.56	99.90	97.93
1942	94.99	95.35	99.58	99.86	97.42
1943	94.82	95.48	99.23	99.80	97.32
1944	96.40	97.09	99.24	99.86	98.15
1945	91.73	93.77	97.44	99.38	95.57
1946	96.31	98.27	97.93	99.29	98.10
1947	93.82	95.95	97.58	98.77	96.76
1948	97.26	98.80	98.40	99.55	98.60
1949	96.74	98.28	98.37	99.42	98.33
1950	96.29	98.02	98.14	99.78	98.08
1951	96.53	96.85	99.65	99.86	98.23
1952	94.02	95.95	97.76	99.54	96.85
1953	92.59	95.27	96.79	99.52	96.02
1954	93.15	95.38	97.34	99.63	96.35
1955	88.87	89.27	99.49	99.58	94.10
1956	90.58	95.52	94.15	99.55	94.83
1957	93.11	94.63	98.15	99.53	96.36
1958	89.61	90.81	98.38	99.26	94.44
1959	92.42	93.39	98.81	99.36	96.03
1960	91.21	92.93	97.74	99.66	95.27
1961	94.34	96.09	97.97	99.69	97.02
1962	89.03	90.85	97.38	99.63	94.00
1963	93.22	94.96	97.94	99.16	96.43
1964	88.59	91.50	96.12	98.43	93.75
1965	96.26	97.42	98.73	99.75	98.07
1966	95.51	98.77	96.60	99.72	97.67
1967	94.92	96.62	98.07	99.73	97.34
1968	96.34	98.52	97.69	99.84	98.11
1969	95.57	96.95	98.45	99.80	97.69
1970	94.38	97.20	96.86	99.76	97.03
1971	94.91	97.28	97.37	99.75	97.33
1972	93.62	95.32	97.99	99.53	96.63
1973	90.43	91.19	98.99	99.48	94.93
1974	89.89	91.33	98.06	99.21	94.58
1975	95.78	98.08	97.55	99.38	97.81
1976	95.59	97.96	97.48	98.99	97.72
1977	94.71	97.06	97.40	99.26	97.23
1978	96.37	98.39	97.85	99.59	98.12
1979	95.40	96.36	98.90	99.81	97.61
1980	97.28	99.11	98.11	99.80	98.61
1981	96.30	97.79	98.38	99.85	98.08
1982	94.53	95.93	98.37	99.59	97.14
1983	89.67	90.38	99.00	99.63	94.50

1984	95.26	96.36	98.76	99.48	97.54
1985	90.20	91.57	98.27	98.86	94.80
1986	89.33	91.04	97.74	98.80	94.27
1987	90.94	92.40	98.08	99.43	95.16
1988	95.24	97.61	97.41	99.68	97.51
1989	87.79	89.91	97.18	98.15	93.40
1990	93.73	97.95	95.48	99.33	96.70
1991	95.13	95.85	99.16	99.81	97.47
1992	91.92	93.62	97.82	99.70	95.67
1993	91.05	92.73	97.80	99.42	95.20
1994	85.87	87.58	97.18	99.49	92.13
1995	87.70	93.04	92.99	99.64	93.01
1996	88.87	90.59	97.62	99.13	93.97
1997	87.31	93.71	92.01	98.53	92.86
1998	86.72	88.92	97.02	97.96	92.79
1999	91.21	94.36	96.27	97.94	95.31
2000	95.09	98.72	96.28	98.80	97.48
2001	96.42	98.74	97.59	99.46	98.16
2002	95.82	98.77	96.93	99.59	97.84
2003	94.72	98.51	96.03	99.40	97.25
2004	93.75	94.47	99.13	99.75	96.74
2005	91.18	91.98	98.95	99.55	95.34
2006	93.53	94.79	98.48	99.53	96.60
2007	96.08	97.19	98.78	99.47	97.98
2008	97.61	98.78	98.78	99.71	98.78
2009	97.00	99.43	97.52	99.89	98.47
2010	94.65	95.40	99.13	99.71	97.23
2011	93.64	94.40	99.12	99.33	96.71
2012	94.10	94.78	99.21	99.56	96.94
2013	93.23	94.49	98.50	99.34	96.45
2014	91.41	93.43	97.48	98.98	95.41
2015	89.70	94.17	94.48	98.91	94.33
2016	91.24	92.49	98.35	99.54	95.33
2017	94.16	96.90	96.94	99.23	96.92
2018	93.02	94.62	98.07	99.35	96.32
2019	95.11	95.71	99.29	99.88	97.47
2020	95.21	95.88	99.21	99.88	97.52
2021	92.16	94.53	97.07	99.68	95.78
2022	90.54	92.48	97.46	99.27	94.90
2023	89.61	94.34	94.26	98.37	94.30
2024	86.68	89.53	96.20	97.36	92.74
2025	88.36	90.08	97.79	98.30	93.78
2026	93.38	95.64	97.42	98.58	96.52
2027	92.45	93.32	98.91	99.58	96.03
2028	92.49	94.56	97.47	99.52	95.99
2029	91.00	93.97	96.47	97.76	95.21
2030	94.96	98.45	96.35	99.11	97.39
2031	96.31	98.29	97.91	99.37	98.10
2032	96.58	97.91	98.57	99.38	98.24
2033	91.27	92.06	98.94	99.70	95.38
2034	92.29	93.13	98.95	99.55	95.95
2035	94.16	95.28	98.68	99.56	96.95
2036	96.03	97.40	98.50	99.51	97.95
2037	87.34	97.22	89.27	98.84	93.07
2038	93.06	93.90	98.97	99.55	96.37
2039	96.77	97.96	98.71	99.90	98.34
2040	92.69	94.97	97.24	99.60	96.09
2041	92.28	93.01	99.08	99.60	95.95
2042	84.76	87.24	96.41	97.75	91.60
2043	91.44	93.80	97.13	98.56	95.44
2044	92.86	94.60	97.94	98.97	96.24
2045	92.71	94.24	98.10	99.58	96.13

2046	92.25	98.41	93.49	99.68	95.89
2047	96.74	98.07	98.57	99.92	98.32
2048	94.97	96.53	98.22	99.70	97.37
2049	96.33	97.84	98.36	99.79	98.10
2050	93.41	94.86	98.24	99.67	96.52
2051	85.99	87.46	97.95	98.51	92.41
2052	94.04	96.18	97.57	99.10	96.87
2053	94.75	97.98	96.56	99.12	97.26
2054	92.94	94.78	97.85	98.71	96.29
2055	92.13	94.95	96.62	99.11	95.78
2056	94.04	98.15	95.67	98.83	96.90
2057	94.74	97.71	96.82	98.79	97.26
2058	92.73	96.27	95.99	98.81	96.13
2059	93.30	96.94	95.98	98.89	96.46
2060	95.33	97.12	98.03	99.12	97.58
2061	91.71	93.31	98.00	99.21	95.60
2062	91.34	92.01	99.13	99.65	95.43
2063	93.97	95.30	98.43	99.53	96.84
2064	93.69	95.46	97.91	99.56	96.67
2065	92.37	93.45	98.62	99.80	95.96
2066	91.59	92.66	98.59	99.71	95.53
2067	93.46	94.21	99.07	99.74	96.58
2068	83.77	84.87	98.07	99.39	90.99
2069	91.93	92.98	98.65	99.60	95.73
2070	86.07	86.92	98.74	99.26	92.46
2071	89.25	91.16	97.36	99.26	94.16
2072	94.46	95.94	98.31	99.28	97.11
2073	88.08	89.06	98.70	99.04	93.63
2074	90.94	92.85	97.51	99.41	95.12
2075	78.25	82.80	90.51	99.16	86.49
2076	77.07	78.32	98.31	97.95	87.18
2077	93.86	94.93	98.71	99.64	96.79
2078	96.63	98.77	97.76	99.76	98.26
2079	96.57	99.23	97.27	99.75	98.24
2080	87.68	92.79	93.22	99.63	93.00
2081	91.03	92.22	98.49	99.17	95.25
2082	93.87	95.38	98.25	99.18	96.79
2083	81.01	83.19	95.82	98.83	89.06
2084	90.90	92.90	97.56	98.44	95.17
2085	93.53	96.53	96.65	98.63	96.59
2086	95.04	97.26	97.58	98.99	97.42
2087	93.13	98.10	94.78	98.65	96.41
2088	87.65	90.76	95.97	97.25	93.30
2089	94.29	96.65	97.35	99.25	97.00
2090	91.03	92.02	98.85	98.79	95.32
2091	94.07	95.29	98.62	99.02	96.93
2092	95.11	97.20	97.68	99.71	97.44
2093	94.00	94.57	99.32	99.62	96.89
2094	86.58	88.97	96.33	99.25	92.50
2095	88.97	91.80	96.08	99.56	93.89
2096	79.30	80.81	96.88	98.94	88.12
2097	88.86	90.61	97.49	99.50	93.92
2098	74.12	75.53	96.22	98.88	84.63
2099	89.89	90.91	98.70	99.08	94.65
2100	95.18	95.72	99.38	99.67	97.52
2101	97.46	98.83	98.56	99.90	98.70
2102	95.81	97.84	97.81	99.61	97.82
2103	94.48	95.79	98.48	99.39	97.12
2104	93.06	96.85	95.75	99.40	96.30
2105	91.85	94.34	96.89	99.74	95.60
2106	94.37	97.09	96.98	99.34	97.03
2107	92.09	95.94	95.60	98.54	95.77

2108	94.96	97.50	97.25	99.05	97.37
2109	95.16	97.17	97.80	99.10	97.48
2110	92.03	93.07	98.87	98.48	95.88
2111	91.23	96.76	93.90	98.41	95.31
2112	83.81	88.98	92.55	96.88	90.73
2113	94.06	95.72	98.10	99.13	96.89
2114	93.85	97.03	96.50	98.77	96.77
2115	94.95	95.96	98.83	99.80	97.37
2116	93.99	95.16	98.59	99.78	96.84
2117	95.29	96.94	98.14	99.82	97.54
2118	95.62	96.64	98.84	99.79	97.73
2119	97.17	97.95	99.17	99.87	98.55
2120	90.30	91.80	97.94	99.66	94.77
2121	91.46	93.39	97.54	99.43	95.42
2122	93.52	94.34	98.99	99.73	96.61
2123	92.60	93.58	98.76	99.77	96.10
2124	94.42	98.69	95.54	99.55	97.09
2125	95.96	96.53	99.36	99.66	97.93
2126	95.71	96.74	98.83	99.76	97.77
2127	89.30	90.73	98.14	98.79	94.29
2128	96.70	98.18	98.43	99.47	98.30
2129	95.48	97.00	98.32	99.31	97.66
2130	90.36	94.90	94.56	98.82	94.73
2131	90.20	94.96	94.38	98.16	94.67
2132	93.88	95.14	98.54	99.26	96.81
2133	91.94	92.98	98.64	99.68	95.73
2134	87.17	88.55	98.22	98.42	93.13
2135	93.01	95.20	97.42	99.12	96.30
2136	95.20	97.75	97.23	99.48	97.49
2137	95.29	96.33	98.83	99.46	97.57
2138	93.53	95.72	97.45	99.36	96.58
2139	95.89	96.57	99.22	99.87	97.88
2140	91.31	93.70	97.12	98.29	95.38
2141	96.31	98.94	97.27	99.87	98.10
2142	94.01	95.53	98.19	99.69	96.85
2143	97.50	98.22	99.23	99.86	98.72
2144	96.24	96.92	99.25	99.65	98.07
2145	96.72	98.68	97.93	99.88	98.31
2146	95.82	96.65	99.07	99.71	97.85
2147	96.40	98.56	97.72	99.77	98.14
2148	95.63	98.17	97.29	99.72	97.73
2149	95.23	97.47	97.55	99.51	97.51
2150	97.14	98.38	98.69	99.93	98.53
2151	93.82	94.66	98.99	99.54	96.78
2152	93.93	94.75	99.01	99.71	96.83
2153	90.13	94.10	95.01	99.59	94.55
2154	87.26	87.71	99.40	99.51	93.19
2155	91.00	92.38	98.18	99.53	95.19
2156	91.15	92.41	98.33	99.58	95.28
2157	94.41	95.25	99.00	99.72	97.09
2158	94.86	96.09	98.60	99.53	97.32
2159	94.38	95.33	98.88	99.62	97.07
2160	96.95	98.15	98.72	99.74	98.43
2161	90.57	92.65	97.27	99.33	94.90
2162	97.39	99.13	98.21	99.65	98.67
2163	93.31	95.73	97.18	99.31	96.45
2164	90.26	96.54	92.95	98.95	94.71
2165	91.86	93.82	97.56	99.13	95.66
2166	93.03	93.56	99.38	99.51	96.38
2167	96.76	97.54	99.16	99.66	98.34
2168	97.14	98.57	98.49	99.67	98.53
2169	94.41	98.73	95.49	99.69	97.08

2170	85.52	87.80	96.47	98.73	91.93
2171	95.53	96.48	98.91	99.83	97.68
2172	94.14	95.88	98.01	99.13	96.93
2173	94.37	96.41	97.66	99.71	97.03
2174	97.70	99.54	98.13	99.95	98.83
2175	97.24	98.02	99.16	99.84	98.59
2176	97.21	98.38	98.75	99.90	98.57
2177	93.42	96.00	97.00	99.56	96.50
2178	90.95	92.73	97.76	98.85	95.18
2179	94.66	95.53	99.02	99.26	97.24
2180	96.28	98.66	97.51	99.59	98.08
2181	96.93	98.89	97.96	99.75	98.42
2182	96.55	98.18	98.25	99.74	98.22
2183	96.06	98.34	97.60	99.32	97.97
2184	96.02	96.21	99.80	99.85	97.97
2185	91.96	92.70	99.04	99.69	95.76
2186	93.66	94.83	98.57	99.80	96.67
2187	96.33	97.93	98.27	99.80	98.10
2188	95.57	97.85	97.53	99.70	97.69
2189	87.51	94.09	91.76	99.61	92.91
2190	96.47	97.84	98.51	99.79	98.18
2191	97.15	98.43	98.65	99.68	98.54
2192	96.42	96.86	99.52	99.71	98.17
2193	85.73	86.69	98.57	99.16	92.25
2194	85.55	92.74	90.54	99.36	91.63
2195	92.58	96.13	95.91	99.30	96.02
2196	96.23	98.53	97.59	99.48	98.06
2197	96.06	98.83	97.12	99.52	97.97
2198	93.72	94.89	98.63	99.30	96.73
2199	94.74	97.07	97.40	99.64	97.24
2200	86.53	89.67	95.27	99.31	92.38
2201	89.54	90.36	98.84	99.63	94.41
2202	89.19	89.73	99.26	99.58	94.26
2203	83.53	90.49	89.90	99.49	90.20
2204	93.15	96.15	96.55	99.32	96.35
2205	95.62	96.82	98.65	99.68	97.73
2206	94.66	96.94	97.48	99.08	97.21
2207	89.80	91.93	97.38	98.01	94.58
2208	94.76	97.74	96.77	99.45	97.25
2209	93.10	97.15	95.53	99.26	96.33
2210	89.35	97.12	91.51	98.82	94.23
2211	94.17	98.28	95.64	99.57	96.94
2212	94.69	96.84	97.61	99.17	97.22
2213	95.57	97.85	97.56	99.14	97.71
2214	95.81	97.44	98.22	99.24	97.83
2215	81.61	83.46	96.63	98.75	89.56
2216	94.56	96.37	97.93	99.64	97.14
2217	93.53	96.16	96.98	99.20	96.57
2218	94.30	98.12	95.94	99.28	97.02
2219	95.48	97.11	98.20	99.47	97.65
2220	92.28	93.15	99.01	98.95	95.99
2221	94.35	96.71	97.38	98.92	97.04
2222	94.58	97.42	96.92	98.85	97.17
2223	90.69	95.87	94.07	98.47	94.96
2224	94.40	96.94	97.20	98.88	97.07
2225	94.95	97.38	97.35	98.97	97.37
2226	93.66	96.11	97.24	98.68	96.67
2227	90.61	92.17	97.91	99.49	94.95
Average	92.56	94.82	97.28	99.34	95.98
Standard c	4.54	4.39	2.02	0.62	2.72
Confidence	0.19	0.18	0.08	0.03	0.11
