# Supplementary material for: In Situ Root Dataset Expansion Strategy Based on an Improved CycleGAN Generator
Source: Plant Phenomics. 2024 Feb 12;6:0148. doi: 10.34133/plantphenomics.0148 (PMC11020132; doi:10.34133/plantphenomics.0148)
Supplement: Supplementary 1 — The network and corresponding weights can be viewed on GitHub (https://github.com/jiwd123/improved_cyclegan) and Zenodo (https://doi.org/10.5281/zenodo.10460303). [file plantphenomics.0148.f1.zip › generalization evaluation_Ws.pdf]

No.	IOU	Recall	Precision	Accuracy	F1
1	94.63	97.35	97.02	99.40	97.18
2	85.95	96.96	87.99	98.60	92.26
3	75.64	81.24	88.92	96.23	84.91
4	6.09	51.22	50.93	11.50	51.07
5	74.12	82.80	83.23	97.64	83.01
6	92.74	97.90	94.47	99.35	96.15
7	86.42	96.22	88.95	98.83	92.44
8	81.85	83.79	97.04	97.82	89.93
9	72.51	80.15	85.13	93.62	82.56
10	8.07	49.45	48.76	15.16	49.10
11	65.27	70.22	80.06	97.80	74.82
12	3.16	46.63	47.65	6.13	47.13
13	79.43	97.36	81.08	97.95	88.48
14	49.22	49.57	49.81	98.34	49.69
15	82.95	98.47	84.24	97.87	90.80
16	17.34	52.27	57.18	31.18	54.62
17	82.89	96.05	85.04	99.24	90.21
18	93.56	98.06	95.19	99.56	96.60
19	55.47	62.82	59.38	98.27	61.05
20	92.63	97.06	95.08	99.37	96.06
21	88.54	96.77	90.90	98.79	93.74
22	64.27	70.23	77.61	96.83	73.74
23	51.93	55.54	64.69	93.83	59.76
24	91.11	99.48	91.50	99.76	95.32
25	75.47	80.99	89.15	95.87	84.87
26	83.62	96.54	85.96	97.67	90.94
27	96.32	97.78	98.43	99.59	98.10
28	3.28	50.03	50.00	6.48	50.01
29	5.69	50.53	50.34	10.80	50.44
30	76.40	86.03	84.31	96.54	85.16
31	73.56	92.96	76.91	96.24	84.18
32	77.81	88.53	84.41	96.27	86.42
33	93.16	96.61	96.09	99.47	96.35
34	95.89	98.37	97.38	99.56	97.87
35	94.94	99.19	95.66	99.45	97.40
36	95.11	97.93	96.97	99.46	97.45
37	94.09	99.27	94.74	99.33	96.95
38	85.52	96.82	87.75	97.99	92.06
39	3.70	50.87	50.14	7.27	50.51
40	3.46	53.06	50.40	6.83	51.70
41	78.09	83.44	90.43	96.33	86.80
42	95.75	98.15	97.43	99.59	97.79
43	75.53	81.22	87.99	97.60	84.47
44	58.57	71.11	62.87	97.22	66.74
45	73.21	78.41	87.54	97.30	82.72
46	57.45	62.97	82.93	90.33	71.59
47	85.67	97.64	87.26	98.75	92.15
48	61.12	77.57	64.66	97.95	70.53
49	1.85	36.52	41.17	3.63	38.70
50	67.25	82.92	72.18	97.40	77.18
51	78.30	81.69	93.07	98.11	87.01
52	82.13	91.16	87.58	98.69	89.33
53	68.06	74.75	81.21	96.30	77.85
54	52.71	67.31	54.21	97.84	60.05
55	62.46	73.67	68.12	98.52	70.79
56	57.71	64.15	65.47	97.07	64.80
57	1.15	1.15	50.00	2.31	2.26
58	64.52	74.25	72.40	97.68	73.32
59	64.77	74.34	73.12	97.27	73.72
60	1.58	42.60	48.51	3.14	45.36
61	89.58	98.88	90.40	99.50	94.45

62	96.80	97.81	98.90	99.66	98.36
63	83.77	86.62	95.65	97.73	90.91
64	94.76	97.47	97.05	99.39	97.26
65	96.41	98.87	97.45	99.51	98.15
66	90.64	94.77	94.97	99.41	94.87
67	86.30	88.96	96.24	97.97	92.46
68	81.89	85.53	94.11	97.25	89.62
69	94.33	99.22	95.02	99.30	97.08
70	84.23	87.61	94.77	98.10	91.05
71	1.62	21.61	49.86	3.23	30.16
72	62.45	81.35	65.98	97.25	72.86
73	1.01	23.23	49.81	2.02	31.69
74	67.67	81.11	73.27	98.31	76.99
75	48.93	49.38	49.54	97.86	49.46
76	67.89	70.69	93.30	95.97	80.43
77	1.85	43.37	49.58	3.69	46.27
78	61.54	63.95	93.86	95.79	76.07
79	50.48	55.58	74.87	89.88	63.80
80	82.28	86.81	92.32	98.88	89.48
81	49.02	49.02	50.00	98.04	49.50
82	49.02	49.02	50.00	98.04	49.50
83	47.17	49.36	47.88	94.28	48.61
84	44.67	51.67	66.08	85.45	57.99
85	50.19	95.64	51.10	98.17	66.61
86	2.22	51.33	50.11	4.42	50.71
87	89.00	94.44	93.29	99.47	93.86
88	1.00	23.64	41.43	1.99	30.11
89	80.64	84.48	92.55	99.37	88.33
90	57.12	99.09	58.03	98.18	73.19
91	59.30	71.63	63.64	97.79	67.40
92	83.08	96.95	84.89	98.72	90.52
93	0.84	14.25	48.67	1.68	22.04
94	73.71	74.78	97.91	98.51	84.80
95	49.23	49.23	50.00	98.45	49.61
96	66.61	71.22	83.26	97.18	76.77
97	70.19	73.06	90.45	98.16	80.83
98	65.97	92.90	67.74	98.04	78.35
99	1.66	42.95	49.92	3.32	46.17
100	2.10	50.80	50.18	4.15	50.49
101	1.32	51.31	50.01	2.63	50.65
102	56.12	62.89	64.46	94.65	63.66
103	88.14	95.09	91.73	99.09	93.38
104	81.61	83.94	95.93	98.29	89.53
105	8.55	50.95	57.30	16.55	53.94
106	52.17	55.34	58.76	96.23	57.00
107	49.69	52.03	50.52	98.25	51.26
108	0.60	0.60	41.87	1.19	1.18
109	91.93	99.36	92.42	99.79	95.76
110	1.80	49.21	49.99	3.60	49.60
111	55.80	58.68	82.62	95.13	68.62
112	75.12	84.63	83.66	96.23	84.14
113	81.84	98.13	83.31	97.74	90.12
114	82.51	99.34	83.11	98.91	90.50
115	56.53	59.90	90.69	93.67	72.15
116	77.80	94.63	80.08	99.09	86.75
117	50.55	56.05	52.11	97.34	54.01
118	85.70	99.15	86.45	98.74	92.37
119	88.29	97.81	89.97	98.45	93.72
120	49.24	59.32	83.91	80.46	69.51
121	85.19	97.35	86.99	98.42	91.88
122	75.99	79.19	93.64	96.84	85.81
123	92.68	97.68	94.59	99.29	96.11

124	93.24	97.22	95.59	99.56	96.40
125	0.87	0.87	50.00	1.75	1.72
126	55.88	58.83	70.25	97.03	64.04
127	67.50	98.98	68.29	98.50	80.82
128	49.65	51.01	50.38	98.44	50.70
129	83.46	95.85	85.82	99.15	90.56
130	68.04	75.25	79.47	97.63	77.30
131	0.77	0.77	50.00	1.54	1.51
132	95.71	98.84	96.74	99.61	97.78
133	90.74	96.11	93.83	99.26	94.96
134	96.52	97.66	98.77	99.57	98.21
135	62.36	68.49	91.20	88.89	78.23
136	37.50	56.04	74.70	63.91	64.04
137	90.51	98.69	91.57	98.96	95.00
138	95.70	98.14	97.40	99.39	97.77
139	6.34	48.29	48.94	11.97	48.61
140	94.59	97.40	96.94	99.04	97.17
141	3.99	45.18	49.28	7.85	47.14
142	0.64	17.40	27.21	1.26	21.23
143	85.90	97.68	87.38	99.18	92.24
144	66.47	80.44	72.00	97.69	75.98
145	89.83	98.17	91.17	99.41	94.54
146	86.04	98.69	86.99	98.97	92.47
147	81.70	98.27	83.25	97.31	90.14
148	94.56	97.24	97.07	99.12	97.15
149	63.59	69.10	92.73	89.95	79.19
150	86.50	90.49	94.24	99.05	92.33
151	81.04	84.69	93.60	97.89	88.92
152	65.00	70.57	88.40	91.14	78.49
153	88.55	94.84	92.49	98.60	93.65
154	3.55	51.60	50.14	7.04	50.86
155	51.03	55.74	61.99	91.60	58.70
156	83.50	88.72	92.37	96.91	90.51
157	96.25	97.59	98.54	99.36	98.07
158	94.57	96.28	98.05	99.31	97.16
159	80.20	84.64	92.36	97.11	88.33
160	94.55	97.84	96.46	99.24	97.15
161	79.21	90.28	84.66	97.55	87.38
162	2.56	44.76	47.33	5.02	46.01
163	73.95	78.47	90.24	96.08	83.94
164	84.15	90.25	91.19	98.71	90.72
165	87.89	92.74	93.67	98.81	93.20
166	88.72	91.80	95.75	99.41	93.73
167	86.99	98.41	88.15	98.86	93.00
168	68.13	73.13	85.28	95.85	78.74
169	80.43	90.37	86.35	96.96	88.32
170	89.22	96.16	92.17	98.42	94.12
171	90.44	97.92	92.10	98.68	94.92
172	93.93	98.86	94.92	99.29	96.85
173	80.19	84.23	92.41	98.53	88.13
174	70.59	78.59	81.75	96.23	80.14
175	5.78	48.82	49.36	11.00	49.09
176	84.88	93.84	88.99	98.31	91.35
177	43.68	53.34	80.27	80.64	64.09
178	1.09	18.60	47.08	2.18	26.66
179	65.81	69.97	87.38	94.94	77.71
180	58.32	97.78	59.86	97.00	74.25
181	3.23	50.11	50.05	6.28	50.08
182	91.77	98.84	92.70	99.39	95.67
183	49.11	49.11	50.00	98.21	49.55
184	83.78	90.61	90.30	98.72	90.46
185	58.13	62.85	87.57	91.49	73.18

186	0.95	0.95	50.00	1.90	1.86
187	67.55	71.46	88.05	95.99	78.89
188	83.13	98.76	83.84	99.47	90.69
189	84.71	97.66	86.25	98.66	91.60
190	49.68	51.83	54.94	95.74	53.34
191	48.83	54.57	74.64	88.37	63.05
192	86.07	93.15	91.00	98.41	92.06
193	69.79	75.00	87.15	94.86	80.62
194	62.88	66.18	84.56	96.61	74.25
195	1.73	47.19	49.82	3.45	48.47
196	92.44	96.63	95.28	99.28	95.95
197	1.15	1.15	50.00	2.29	2.24
198	57.75	62.29	82.27	92.48	70.90
199	67.40	89.44	70.14	97.79	78.62
200	72.23	76.60	88.69	97.18	82.20
201	2.67	50.70	50.08	5.28	50.39
202	51.69	60.32	87.02	83.39	71.25
203	68.46	75.76	79.31	98.37	77.49
204	47.63	56.25	78.62	82.74	65.58
205	1.46	1.46	50.00	2.93	2.84
206	71.95	75.66	89.37	98.00	81.94
207	64.35	74.66	71.23	98.51	72.91
208	59.15	62.71	79.31	95.75	70.04
209	81.89	86.97	91.92	97.33	89.38
210	1.32	39.42	48.39	2.64	43.45
211	2.87	49.24	49.71	5.61	49.47
212	56.57	63.71	62.16	97.51	62.93
213	82.16	85.35	94.26	98.87	89.59
214	75.73	81.37	88.07	97.83	84.59
215	2.23	42.40	47.20	4.39	44.67
216	91.02	97.11	93.22	99.75	95.12
217	2.70	48.30	49.44	5.30	48.86
218	2.76	45.54	48.16	5.40	46.81
219	84.39	95.93	86.93	98.60	91.21
220	87.66	92.21	93.93	98.77	93.06
221	48.38	53.66	60.04	88.86	56.67
222	45.50	50.81	55.36	88.39	52.99
223	66.60	73.00	79.90	96.61	76.29
224	65.92	73.80	77.26	95.99	75.49
225	55.31	61.53	62.00	95.99	61.76
226	2.40	52.39	50.00	4.79	51.17
227	63.74	69.45	82.59	92.93	75.46
228	70.81	79.69	79.74	98.66	79.71
229	72.44	94.02	74.49	98.43	83.13
230	63.42	69.78	79.64	93.23	74.38
231	85.61	92.88	90.56	99.04	91.70
232	2.07	44.51	45.22	4.05	44.86
233	69.13	89.02	72.35	97.80	79.82
234	68.76	97.42	69.55	99.06	81.16
235	85.10	96.82	87.12	98.80	91.72
236	92.28	96.68	95.04	99.37	95.86
237	65.22	71.44	77.85	97.40	74.51
238	1.96	1.96	50.00	3.93	3.78
239	89.30	93.97	94.16	99.16	94.06
240	66.31	90.63	68.72	97.56	78.16
241	60.50	65.73	75.49	95.27	70.27
242	90.67	95.83	93.99	99.34	94.90
243	68.69	74.24	86.69	93.71	79.98
244	65.61	98.53	66.18	99.05	79.18
245	46.99	47.02	49.96	93.98	48.45
246	1.02	41.35	45.85	2.02	43.49
247	61.27	65.35	78.54	96.57	71.34

248	68.98	75.00	83.25	96.39	78.91
249	72.39	88.64	76.21	98.84	81.96
250	64.25	75.11	70.85	98.30	72.92
251	49.03	49.13	49.89	98.06	49.51
252	86.68	89.01	96.38	99.39	92.55
253	66.88	75.93	75.39	98.62	75.66
254	46.32	54.56	80.77	83.48	65.13
255	0.69	0.69	50.00	1.39	1.37
256	63.17	69.79	75.13	96.51	72.36
257	75.71	95.22	77.67	98.61	85.55
258	48.40	49.07	49.86	96.66	49.46
259	88.63	92.74	94.55	99.48	93.63
260	54.36	95.57	56.12	96.55	70.72
261	55.39	59.28	89.39	92.61	71.29
262	0.50	1.77	40.51	1.00	3.40
263	49.25	58.02	74.19	82.40	65.12
264	72.73	80.80	84.00	95.01	82.37
265	64.59	73.28	74.37	96.35	73.82
266	81.50	87.27	90.55	98.53	88.88
267	70.19	75.58	85.13	96.97	80.07
268	5.67	51.14	51.36	10.73	51.25
269	61.27	66.41	74.77	96.84	70.35
270	50.58	55.29	82.13	90.66	66.09
271	65.84	76.53	72.78	98.59	74.61
272	56.98	60.70	86.86	93.34	71.46
273	3.58	51.55	50.90	6.93	51.23
274	59.79	64.69	77.28	94.30	70.43
275	53.20	57.90	57.31	97.43	57.60
276	54.49	67.97	56.36	98.26	61.62
277	87.27	99.17	88.00	98.82	93.25
278	55.55	59.97	64.04	96.96	61.94
279	82.73	92.62	87.11	99.13	89.78
280	62.80	71.43	72.26	96.02	71.85
281	43.43	54.76	69.94	76.51	61.43
282	64.32	69.75	78.53	97.07	73.88
283	43.47	53.10	75.97	80.50	62.51
284	60.08	71.00	65.17	98.11	67.96
285	75.70	93.61	78.14	98.98	85.18
286	0.93	37.63	46.55	1.86	41.62
287	62.28	67.00	78.86	96.04	72.45
288	45.48	56.49	74.84	77.73	64.38
289	68.18	81.42	75.23	95.39	78.20
290	62.99	69.20	79.07	93.55	73.80
291	56.48	94.30	57.19	98.78	71.20
292	60.73	65.68	73.68	97.28	69.45
293	1.69	26.69	49.98	3.39	34.80
294	17.38	52.44	60.77	31.83	56.30
295	64.54	71.67	76.38	96.30	73.95
296	65.36	70.00	84.03	95.52	76.38
297	77.82	88.63	83.65	98.68	86.07
298	89.98	98.85	90.79	99.69	94.65
299	78.52	86.82	86.38	98.18	86.60
300	49.71	55.17	50.73	97.91	52.86
301	49.26	49.26	50.00	98.52	49.63
302	43.70	51.42	63.02	83.84	56.63
303	51.92	55.34	69.61	93.73	61.66
304	74.62	76.77	94.36	98.60	84.66
305	2.72	52.10	50.53	5.35	51.30
306	1.87	1.87	49.98	3.75	3.61
307	1.08	1.08	50.00	2.15	2.11
308	56.05	61.04	68.42	94.40	64.52
309	80.62	83.38	94.53	99.02	88.60

310	75.33	91.95	78.43	98.87	84.65
311	1.86	51.09	50.14	3.69	50.61
312	64.29	78.83	70.49	95.20	74.43
313	88.59	94.06	93.21	98.99	93.63
314	37.52	50.70	57.71	72.51	53.98
315	85.63	88.57	95.45	98.98	91.88
316	89.69	94.47	94.18	98.81	94.33
317	73.56	78.66	88.47	96.53	83.28
318	88.29	96.13	91.08	98.96	93.54
319	86.73	93.62	91.38	98.57	92.49
320	64.82	69.13	86.81	94.43	76.97
321	68.77	74.82	85.84	93.28	79.96
322	66.15	79.59	71.86	97.79	75.53
323	2.67	48.14	49.12	5.23	48.63
324	5.46	50.35	50.31	10.36	50.33
325	75.74	94.30	77.92	98.98	85.33
326	76.21	92.70	79.37	98.22	85.52
327	56.30	63.02	87.51	87.36	73.27
328	82.94	94.51	86.28	98.18	90.20
329	74.18	83.03	83.80	95.83	83.42
330	5.77	52.73	51.40	10.97	52.06
331	86.43	98.29	87.86	97.94	92.78
332	5.71	51.74	50.62	10.96	51.17
333	59.34	73.29	62.74	98.54	67.60
334	62.27	98.25	63.90	96.79	77.44
335	69.57	74.96	88.31	93.28	81.09
336	87.22	93.06	92.63	97.75	92.84
337	65.26	71.87	79.04	95.33	75.28
338	84.05	94.98	87.41	97.39	91.04
339	46.26	61.51	78.91	71.29	69.13
340	57.33	65.12	85.38	85.68	73.89
341	5.38	51.46	50.69	10.28	51.07
342	84.64	92.78	89.65	97.71	91.19
343	4.43	52.11	50.54	8.62	51.31
344	77.77	97.22	79.25	98.38	87.32
345	94.30	97.40	96.62	99.09	97.01
346	80.10	92.34	84.65	96.69	88.33
347	82.74	87.64	92.47	97.22	89.99
348	92.04	94.24	97.36	98.74	95.77
349	65.73	71.69	88.16	90.60	79.08
350	71.30	97.70	73.59	95.62	83.95
351	87.86	92.01	94.58	97.90	93.27
352	67.02	72.48	88.59	91.90	79.73
353	79.51	97.29	81.26	97.73	88.55
354	62.27	73.63	67.69	98.69	70.53
355	69.35	72.34	90.70	97.31	80.49
356	52.06	96.48	53.84	96.47	69.11
357	70.88	91.15	73.79	97.87	81.55
358	65.96	70.26	83.40	97.20	76.27
359	61.78	93.92	63.62	96.97	75.86
360	86.96	99.10	87.68	99.03	93.04
361	67.57	73.31	83.43	95.36	78.04
362	4.84	49.02	49.31	9.25	49.16
363	82.63	91.56	87.94	98.56	89.71
364	68.62	87.26	72.94	96.19	79.46
365	80.31	95.24	83.17	97.19	88.79
366	1.32	43.82	48.27	2.61	45.94
367	86.42	97.87	88.13	97.76	92.74
368	79.32	97.19	81.58	96.34	88.70
369	84.76	97.20	86.70	98.08	91.65
370	79.58	94.62	82.55	97.58	88.17
371	85.29	95.34	88.57	97.40	91.83

372	11.87	54.18	53.55	21.24	53.86
373	91.59	96.55	94.47	98.52	95.50
374	83.86	92.84	88.59	97.94	90.66
375	86.70	94.81	90.42	98.11	92.56
376	43.69	59.71	76.28	69.19	66.99
377	59.09	63.76	84.91	92.20	72.83
378	5.02	43.90	48.29	9.75	45.99
379	5.37	50.62	50.15	10.41	50.38
380	75.60	97.44	77.99	95.60	86.64
381	5.48	48.15	49.26	10.50	48.69
382	86.68	96.10	89.53	97.70	92.70
383	5.19	48.08	49.60	10.11	48.83
384	70.57	75.88	89.64	92.90	82.19
385	88.81	96.23	91.66	98.42	93.89
386	70.02	74.51	92.00	93.18	82.33
387	73.61	79.48	87.86	95.27	83.46
388	83.66	98.50	84.87	98.16	91.18
389	87.33	92.66	93.06	98.48	92.86
390	77.62	97.86	79.35	97.04	87.64
391	72.98	80.11	85.82	94.37	82.87
392	5.65	51.39	50.42	10.89	50.90
393	78.74	96.91	80.98	96.64	88.23
394	81.27	86.19	91.97	97.22	88.98
395	78.70	85.47	88.42	97.27	86.92
396	87.50	97.10	89.58	98.54	93.19
397	3.01	43.69	49.11	5.96	46.24
398	55.32	58.63	79.92	94.39	67.64
399	77.56	81.71	92.38	96.31	86.72
400	59.75	77.80	62.38	98.42	69.24
401	2.39	51.52	50.71	4.68	51.11
402	3.28	49.39	49.66	6.37	49.52
403	1.12	46.55	47.39	2.22	46.97
404	65.05	69.45	86.40	94.55	77.00
405	93.03	97.03	95.56	99.19	96.29
406	54.08	59.18	80.48	90.48	68.21
407	79.98	88.54	86.99	98.11	87.76
408	80.09	93.20	83.99	97.20	88.35
409	91.15	94.16	96.34	98.59	95.24
410	86.58	99.31	87.27	98.70	92.90
411	7.41	51.85	51.63	13.79	51.74
412	92.72	96.33	95.91	98.92	96.12
413	93.73	96.61	96.77	99.04	96.69
414	86.34	91.36	93.29	97.49	92.31
415	86.64	97.46	88.70	97.34	92.87
416	32.28	56.38	69.85	53.95	62.40
417	1.74	38.50	49.72	3.47	43.40
418	60.15	66.30	81.86	90.29	73.26
419	94.74	98.70	95.90	99.21	97.28
420	70.82	78.43	81.88	97.32	80.12
421	86.81	97.72	88.33	99.07	92.79
422	84.39	91.69	90.09	98.84	90.88
423	89.20	95.97	92.26	98.75	94.08
424	1.89	44.86	49.31	3.75	46.98
425	65.55	70.46	81.48	96.98	75.57
426	68.84	97.14	69.91	98.51	81.31
427	55.13	77.03	56.30	98.70	65.06
428	52.95	55.81	61.83	96.62	58.67
429	69.09	93.80	70.54	99.15	80.52
430	69.41	81.90	76.80	95.59	79.27
431	92.67	95.48	96.71	98.96	96.09
432	81.43	83.90	95.66	98.25	89.39
433	60.62	90.92	61.89	98.48	73.65

434	72.65	84.98	78.59	98.77	81.66
435	90.11	95.36	93.76	99.46	94.55
436	52.46	60.66	55.06	96.78	57.72
437	63.80	92.99	65.39	97.97	76.79
438	1.38	1.38	50.00	2.76	2.69
439	68.14	77.31	77.59	97.00	77.45
440	91.45	96.63	94.15	99.38	95.37
441	58.04	61.11	77.91	96.40	68.50
442	89.69	91.05	98.17	99.19	94.48
443	63.85	67.62	94.85	92.99	78.95
444	52.99	95.51	54.07	97.89	69.05
445	59.89	67.36	72.97	92.23	70.05
446	91.40	95.94	94.74	99.32	95.33
447	51.05	70.56	52.38	97.48	60.12
448	55.12	82.03	56.83	97.28	67.14
449	55.56	58.52	77.20	95.45	66.58
450	69.60	75.77	81.86	98.77	78.70
451	55.56	60.43	62.34	97.61	61.37
452	1.44	33.66	49.00	2.88	39.91
453	80.28	84.99	91.68	97.85	88.21
454	76.27	81.25	89.30	98.13	85.08
455	62.03	66.45	85.60	93.53	74.82
456	91.18	98.01	92.78	98.89	95.32
457	82.58	91.35	88.25	97.57	89.77
458	56.53	60.76	79.71	93.05	68.95
459	87.13	94.40	91.08	99.28	92.71
460	46.28	51.13	53.91	88.87	52.48
461	74.84	77.53	93.66	97.85	84.83
462	53.17	56.69	76.34	93.59	65.07
463	57.21	65.97	64.42	94.67	65.18
464	47.33	56.38	90.04	82.35	69.34
465	47.80	51.21	56.03	92.57	53.51
466	92.08	93.88	97.72	99.66	95.76
467	60.28	68.56	71.19	93.25	69.85
468	70.61	75.22	88.40	95.74	81.28
469	73.17	77.30	89.29	97.84	82.86
470	59.75	64.74	73.03	96.39	68.64
471	62.20	83.27	64.94	97.89	72.97
472	48.75	62.66	51.00	95.37	56.23
473	63.16	68.00	81.63	94.91	74.20
474	51.20	55.31	77.91	91.95	64.69
475	67.04	98.33	68.35	97.52	80.64
476	6.28	44.92	39.28	11.97	41.91
477	76.66	94.41	78.89	99.04	85.96
478	49.28	52.46	50.16	98.16	51.29
479	49.26	49.31	49.95	98.51	49.62
480	1.29	1.29	50.00	2.58	2.52
481	88.59	91.66	95.71	99.60	93.64
482	72.11	84.87	78.32	97.51	81.46
483	57.22	63.76	77.50	89.25	69.96
484	69.43	72.51	89.84	97.71	80.25
485	50.26	56.36	52.59	95.79	54.41
486	66.30	90.89	69.12	96.52	78.52
487	1.06	35.25	49.58	2.12	41.21
488	49.03	49.03	50.00	98.06	49.51
489	1.50	1.50	50.00	3.00	2.91
490	50.78	57.68	84.62	86.45	68.60
491	56.70	59.88	75.73	95.88	66.88
492	1.33	40.97	49.84	2.66	44.97
493	1.57	47.44	45.48	3.10	46.44
494	58.60	62.93	88.15	92.26	73.44
495	84.74	93.28	89.20	98.64	91.20

496	58.98	83.43	61.58	96.59	70.86
497	87.63	96.19	90.26	99.20	93.13
498	51.69	55.90	74.41	91.91	63.84
499	72.50	81.16	81.79	98.03	81.47
500	91.49	94.98	95.77	99.49	95.37
501	88.65	91.18	96.42	99.42	93.73
502	61.04	70.33	70.88	93.53	70.60
503	90.88	93.93	96.20	99.02	95.05
504	76.01	80.53	89.43	99.02	84.75
505	1.22	40.53	49.62	2.44	44.62
506	72.63	95.07	74.34	98.63	83.44
507	58.73	63.31	89.15	91.68	74.04
508	3.48	49.13	49.42	6.73	49.27
509	90.72	98.47	91.88	99.37	95.07
510	90.03	98.75	90.96	99.46	94.69
511	89.10	93.32	94.57	99.15	93.94
512	89.68	99.48	90.18	99.11	94.60
513	93.84	97.22	96.27	99.31	96.74
514	81.23	86.64	90.68	99.18	88.61
515	1.88	51.23	50.56	3.71	50.89
516	79.01	83.88	90.61	98.63	87.11
517	52.36	56.87	65.75	92.37	60.99
518	57.28	70.19	62.00	95.45	65.84
519	62.48	86.32	64.41	98.67	73.78
520	85.82	96.89	87.72	99.41	92.08
521	65.71	69.98	91.46	93.01	79.29
522	81.89	95.45	84.57	98.04	89.68
523	9.09	51.59	55.14	17.10	53.30
524	0.77	0.77	50.00	1.55	1.52
525	58.14	62.91	69.90	96.62	66.22
526	2.05	42.10	49.72	4.10	45.59
527	90.06	93.77	95.30	99.26	94.53
528	75.75	78.41	93.06	99.02	85.11
529	64.34	66.40	92.92	97.07	77.45
530	65.05	69.36	82.21	97.23	75.24
531	48.91	48.93	49.97	97.81	49.45
532	88.63	97.88	90.34	98.07	93.96
533	93.05	97.70	94.97	99.29	96.32
534	74.66	82.63	84.77	96.67	83.69
535	50.04	54.15	50.92	98.31	52.48
536	69.36	75.31	83.36	96.87	79.13
537	82.96	93.73	86.59	99.15	90.02
538	2.90	48.69	49.36	5.65	49.03
539	65.22	68.99	89.19	94.78	77.80
540	89.63	93.36	95.14	99.71	94.24
541	57.03	98.47	58.44	97.22	73.34
542	76.35	78.59	94.84	98.39	85.95
543	8.36	53.80	52.21	15.53	53.00
544	48.98	48.98	50.00	97.95	49.48
545	74.48	97.40	75.60	98.82	85.12
546	62.93	84.96	65.46	98.11	73.94
547	49.39	99.28	50.11	98.56	66.60
548	87.09	90.71	94.77	99.33	92.70
549	2.03	52.01	50.02	4.06	51.00
550	76.88	84.45	86.31	97.68	85.37
551	66.41	69.94	88.08	96.28	77.97
552	66.62	69.92	89.35	96.23	78.45
553	68.24	75.78	79.30	97.52	77.50
554	77.05	85.57	86.14	95.42	85.85
555	92.71	96.13	96.03	99.69	96.08
556	87.72	91.86	94.33	99.27	93.08
557	58.27	62.25	74.94	95.74	68.01

558	58.69	84.41	60.37	98.15	70.39
559	91.52	98.49	92.79	98.79	95.55
560	94.71	96.89	97.57	99.28	97.23
561	86.92	97.61	88.62	98.57	92.90
562	85.86	93.12	90.84	97.95	91.97
563	76.82	97.55	78.67	96.96	87.10
564	6.94	50.10	50.11	12.99	50.10
565	7.39	50.99	50.69	13.79	50.84
566	82.12	94.91	85.52	96.54	89.97
567	83.01	98.34	84.63	97.02	90.97
568	65.96	72.46	84.93	91.13	78.20
569	80.97	90.33	86.69	98.67	88.47
570	77.15	81.93	91.10	96.18	86.27
571	83.19	98.13	84.69	97.72	90.92
572	78.53	83.38	91.56	96.05	87.28
573	87.06	92.80	92.55	98.64	92.67
574	59.95	62.95	89.66	94.98	73.97
575	69.29	80.34	76.26	98.84	78.25
576	86.42	96.37	88.81	99.00	92.43
577	81.33	92.13	85.73	98.99	88.81
578	69.06	98.73	70.25	97.68	82.09
579	81.48	93.72	84.98	98.52	89.13
580	59.55	64.48	70.39	98.04	67.31
581	71.66	77.32	86.54	95.94	81.67
582	86.77	98.57	87.85	98.80	92.90
583	51.86	56.73	87.17	90.44	68.73
584	58.35	99.07	59.27	98.15	74.17
585	1.43	51.34	50.09	2.86	50.71
586	83.37	96.61	86.10	96.10	91.05
587	83.17	91.74	89.07	95.70	90.38
588	4.91	49.71	49.76	9.37	49.74
589	88.34	98.28	89.77	98.14	93.84
590	82.51	93.03	86.82	97.83	89.82
591	89.89	95.20	93.64	99.59	94.41
592	81.69	96.34	83.84	98.24	89.65
593	78.21	92.85	82.31	96.05	87.27
594	75.59	96.90	78.41	94.93	86.68
595	92.37	97.55	94.45	98.53	95.97
596	72.06	77.98	88.30	93.32	82.82
597	89.48	95.93	92.67	98.24	94.27
598	7.78	51.59	51.45	14.44	51.52
599	79.36	97.82	81.14	97.01	88.71
600	86.94	98.94	87.93	98.32	93.11
601	95.25	98.49	96.60	99.46	97.53
602	75.85	87.96	81.48	98.27	84.60
603	51.83	54.75	55.32	97.74	55.03
604	60.12	62.36	89.60	96.59	73.53
605	62.33	97.28	62.95	99.06	76.44
606	72.36	83.08	80.27	96.82	81.65
607	89.85	95.25	93.69	98.30	94.46
608	66.48	82.65	72.20	95.09	77.07
609	2.66	49.01	49.92	5.29	49.46
610	70.29	90.51	74.42	95.03	81.68
611	75.79	90.27	80.82	95.78	85.28
612	57.44	64.24	74.91	89.68	69.17
613	1.68	44.08	48.36	3.33	46.12
614	90.60	97.42	92.66	98.61	94.98
615	87.21	96.83	89.74	97.12	93.15
616	85.22	93.67	89.89	96.31	91.75
617	77.83	86.89	85.99	95.63	86.44
618	61.91	96.90	63.57	96.92	76.77
619	48.74	54.71	86.35	88.11	66.98

620	47.58	52.76	70.87	89.34	60.49
621	54.06	61.10	57.50	97.60	59.25
622	61.22	91.14	62.48	98.58	74.14
623	1.03	48.01	49.04	2.03	48.52
624	69.98	74.65	90.58	93.58	81.84
625	64.16	68.08	82.36	97.24	74.54
626	64.88	77.89	70.59	97.86	74.06
627	85.53	93.12	90.20	99.28	91.64
628	68.60	96.14	69.99	98.14	81.01
629	3.13	48.49	49.44	6.11	48.96
630	54.11	57.44	68.03	95.42	62.29
631	0.70	32.51	49.79	1.40	39.34
632	84.15	94.29	87.97	97.44	91.02
633	89.02	96.39	91.84	97.95	94.06
634	69.97	76.77	84.02	94.60	80.23
635	77.08	82.79	90.02	95.03	86.26
636	55.95	97.24	58.41	95.15	72.98
637	82.33	94.77	85.50	97.82	89.89
638	93.09	96.39	96.27	98.99	96.33
639	65.98	73.19	80.61	93.25	76.72
640	91.21	94.16	96.32	99.37	95.23
641	58.40	71.92	61.62	98.45	66.37
642	48.81	49.39	49.41	97.62	49.40
643	80.06	94.01	82.90	99.42	88.11
644	59.34	97.87	60.52	97.73	74.79
645	70.45	90.25	73.18	98.78	80.82
646	55.52	97.58	57.86	95.37	72.64
647	59.65	63.47	78.13	95.97	70.04
648	71.38	89.24	74.72	98.70	81.33
649	66.87	87.85	69.52	98.64	77.62
650	62.19	85.52	64.02	99.01	73.22
651	60.80	75.77	65.00	97.36	69.97
652	51.77	56.00	66.18	92.42	60.67
653	87.92	97.59	89.60	99.05	93.43
654	78.20	85.38	87.96	96.36	86.65
655	69.46	87.73	73.30	97.50	79.87
656	86.88	98.32	87.97	99.39	92.86
657	48.27	54.23	76.17	87.92	63.36
658	83.61	93.68	87.55	98.45	90.51
659	91.27	94.02	96.57	99.25	95.28
660	88.19	92.42	94.30	99.48	93.35
661	86.80	98.02	88.07	99.38	92.78
662	49.22	54.79	73.31	88.75	62.71
663	92.22	95.63	95.98	99.38	95.81
664	1.58	1.58	50.00	3.17	3.07
665	46.49	53.22	71.98	86.15	61.20
666	50.46	53.38	54.30	95.86	53.83
667	63.91	81.62	67.97	97.18	74.17
668	82.24	89.38	89.18	99.36	89.28
669	84.07	91.68	89.78	98.25	90.72
670	72.90	81.49	82.18	98.06	81.84
671	78.97	84.61	89.59	98.43	87.03
672	81.43	86.85	91.57	96.51	89.14
673	68.98	76.72	81.13	95.37	78.86
674	91.52	96.01	94.79	99.47	95.40
675	88.80	93.80	93.76	98.66	93.78
676	76.15	80.83	90.54	96.69	85.41
677	49.20	49.21	49.98	98.39	49.60
678	49.44	58.15	81.06	82.83	67.72
679	1.15	1.15	50.00	2.29	2.24
680	87.39	95.64	90.35	99.43	92.92
681	80.74	90.73	86.14	98.38	88.38

682	80.97	86.26	90.93	98.49	88.53
683	1.19	51.09	50.10	2.37	50.59
684	54.22	57.22	67.60	96.16	61.98
685	66.69	72.01	82.96	95.90	77.10
686	84.46	93.21	88.85	98.94	90.98
687	50.16	66.35	51.02	98.30	57.68
688	49.23	49.23	50.00	98.45	49.61
689	54.39	57.98	87.80	93.16	69.84
690	90.90	96.12	94.01	99.21	95.05
691	94.27	98.43	95.63	99.28	97.01
692	78.17	98.23	79.87	96.80	88.11
693	49.64	51.90	52.44	96.06	52.17
694	54.92	78.82	56.91	96.91	66.10
695	80.64	94.03	83.95	98.01	88.70
696	61.22	65.82	81.38	94.22	72.78
697	68.38	76.99	77.35	98.97	77.17
698	68.35	77.96	76.82	98.10	77.38
699	61.97	67.36	76.40	96.04	71.59
700	73.39	91.52	76.53	98.15	83.36
701	48.77	48.77	50.00	97.54	49.38
702	0.60	23.93	45.37	1.19	31.33
703	49.36	49.37	50.00	98.73	49.68
704	48.70	49.13	49.56	97.41	49.34
705	54.67	58.87	62.55	96.76	60.66
706	87.52	96.51	89.98	98.68	93.13
707	59.73	66.35	85.25	88.41	74.62
708	84.56	96.39	86.85	98.51	91.37
709	92.60	95.43	96.68	99.14	96.05
710	70.98	81.09	78.88	98.42	79.97
711	56.28	82.57	57.71	98.10	67.94
712	62.98	65.99	85.24	97.03	74.39
713	79.25	90.19	84.50	98.56	87.26
714	51.92	56.07	54.57	97.71	55.31
715	63.49	80.62	67.27	97.98	73.34
716	1.18	1.18	50.00	2.37	2.31
717	70.99	95.99	72.20	98.99	82.41
718	75.18	80.13	88.71	98.16	84.20
719	59.46	78.76	62.27	97.56	69.55
720	85.85	90.57	93.26	98.81	91.89
721	83.05	93.14	87.10	99.19	90.02
722	62.81	65.43	85.81	97.55	74.25
723	2.19	47.84	48.72	4.30	48.28
724	1.60	51.59	50.01	3.20	50.79
725	62.80	70.74	72.25	97.08	71.49
726	42.82	50.73	55.20	82.82	52.87
727	34.22	52.10	73.26	64.32	60.89
728	48.87	49.06	49.80	97.74	49.43
729	49.50	51.04	51.10	97.19	51.07
730	67.65	71.21	89.77	95.82	79.42
731	69.14	73.70	89.52	94.23	80.84
732	4.02	48.77	49.44	7.78	49.10
733	77.99	81.59	92.74	97.80	86.81
734	58.09	89.98	59.02	98.82	71.28
735	89.35	94.31	93.86	99.28	94.08
736	1.51	30.16	45.47	2.99	36.27
737	66.76	71.19	85.20	96.22	77.56
738	70.30	79.21	80.20	96.65	79.70
739	91.18	97.10	93.46	99.25	95.25
740	82.67	94.78	85.58	99.08	89.94
741	59.24	98.64	59.89	98.75	74.53
742	69.36	81.33	76.61	96.74	78.90
743	73.31	79.58	86.50	96.06	82.89

744	78.68	87.62	85.81	98.44	86.71
745	80.73	84.83	92.41	98.80	88.46
746	82.65	89.22	90.15	98.48	89.68
747	77.49	80.61	93.09	98.52	86.40
748	87.42	90.61	95.38	99.36	92.93
749	54.99	66.37	57.51	98.00	61.63
750	55.04	97.07	55.84	98.46	70.90
751	76.33	80.18	91.25	98.32	85.35
752	77.27	79.40	95.58	98.14	86.74
753	84.46	87.40	95.26	98.79	91.16
754	63.31	83.26	66.32	98.05	73.83
755	82.63	95.12	85.41	98.73	90.00
756	93.81	95.83	97.65	99.32	96.73
757	53.03	56.26	76.31	94.11	64.77
758	60.69	65.25	87.58	92.33	74.78
759	84.82	92.00	90.42	98.50	91.21
760	78.54	97.87	79.95	97.99	88.01
761	62.04	71.23	70.46	96.26	70.84
762	80.67	89.21	87.12	99.08	88.16
763	71.83	76.81	86.43	98.09	81.33
764	85.91	88.89	95.34	99.35	92.00
765	49.29	49.29	50.00	98.59	49.64
766	71.61	74.44	90.80	98.69	81.81
767	62.46	66.63	77.35	98.25	71.59
768	1.41	43.31	49.41	2.80	46.16
769	48.95	48.95	50.00	97.91	49.47
770	65.20	91.30	67.38	97.50	77.54
771	0.98	17.98	46.67	1.95	25.96
772	66.13	71.21	83.04	95.89	76.67
773	82.56	91.90	87.64	98.23	89.72
774	65.37	72.60	76.61	97.17	74.55
775	75.54	90.47	79.27	99.29	84.50
776	73.01	88.21	77.26	98.77	82.37
777	77.06	81.06	91.85	97.37	86.12
778	81.96	86.00	93.34	97.61	89.52
779	55.75	64.07	59.56	98.06	61.73
780	85.11	94.69	88.56	98.67	91.52
781	67.83	78.64	75.43	97.71	77.01
782	68.76	73.08	87.15	96.35	79.50
783	1.49	48.70	49.99	2.98	49.34
784	63.18	67.25	80.36	97.27	73.22
785	76.40	83.45	86.66	97.55	85.03
786	2.40	48.73	49.79	4.75	49.25
787	81.62	83.00	97.91	98.32	89.84
788	84.40	91.08	90.65	98.95	90.87
789	73.74	83.92	81.79	96.94	82.84
790	59.44	62.57	80.16	96.51	70.28
791	80.23	91.22	85.22	97.93	88.12
792	76.56	80.66	91.13	97.90	85.57
793	77.09	86.07	84.68	98.68	85.37
794	63.91	68.29	82.05	96.23	74.54
795	69.15	73.60	86.18	97.19	79.39
796	66.32	72.75	78.36	98.01	75.45
797	50.22	53.86	58.92	93.19	56.28
798	72.78	84.16	79.29	98.95	81.65
799	60.36	70.07	66.25	98.17	68.11
800	0.73	50.71	50.02	1.47	50.36
801	69.45	93.97	71.12	98.64	80.96
802	76.67	83.27	87.70	96.83	85.43
803	74.59	80.56	88.33	95.09	84.27
804	74.60	79.73	90.11	94.91	84.60
805	83.94	90.91	90.35	98.15	90.63

806	74.75	92.83	77.39	98.83	84.41
807	52.18	55.05	64.57	95.34	59.43
808	0.59	28.88	47.45	1.17	35.91
809	81.75	91.07	87.02	99.24	89.00
810	91.39	98.50	92.54	99.47	95.43
811	90.96	98.65	92.07	98.94	95.25
812	50.23	97.70	52.53	95.42	68.32
813	5.66	49.25	49.85	11.01	49.55
814	90.39	94.97	94.56	98.57	94.77
815	1.51	1.51	50.00	3.03	2.94
816	85.54	95.65	88.46	98.26	91.92
817	86.53	96.60	88.86	98.53	92.57
818	82.77	96.69	84.68	98.76	90.29
819	2.83	46.48	49.38	5.59	47.89
820	67.85	95.75	69.83	96.86	80.76
821	81.71	91.81	86.53	98.57	89.09
822	55.37	59.65	69.09	94.59	64.02
823	62.19	65.75	87.95	94.69	75.25
824	86.88	93.17	92.01	98.31	92.59
825	80.63	88.95	87.66	97.66	88.30
826	64.12	74.78	73.09	94.35	73.93
827	88.64	93.73	93.57	99.22	93.65
828	0.53	17.97	37.90	1.06	24.38
829	68.01	71.41	88.44	97.15	79.02
830	2.68	45.95	49.10	5.28	47.47
831	2.56	46.95	49.19	5.04	48.05
832	89.49	95.85	92.60	99.48	94.20
833	64.33	72.00	73.71	98.43	72.85
834	49.40	51.46	51.57	96.28	51.51
835	2.03	43.49	49.88	4.05	46.46
836	63.95	93.61	65.23	98.51	76.89
837	66.79	68.79	92.66	97.78	78.96
838	49.39	49.39	50.00	98.78	49.69
839	50.58	54.34	52.08	97.76	53.19
840	0.84	0.84	50.00	1.69	1.66
841	79.67	89.35	85.53	99.33	87.40
842	0.85	50.85	50.00	1.70	50.42
843	69.56	96.91	70.52	98.96	81.63
844	62.16	86.14	64.63	97.49	73.85
845	2.55	50.78	50.24	5.00	50.51
846	64.99	78.06	70.93	97.36	74.33
847	79.96	97.68	81.17	98.97	88.66
848	1.95	45.13	49.38	3.87	47.16
849	59.33	93.46	60.41	98.34	73.38
850	51.92	56.99	81.67	90.11	67.13
851	83.58	91.91	89.09	97.49	90.48
852	83.66	91.48	89.60	97.28	90.53
853	83.91	94.79	87.18	98.26	90.83
854	92.29	96.38	95.35	99.24	95.86
855	79.91	85.25	90.84	97.29	87.95
856	92.10	96.73	94.85	98.85	95.78
857	70.37	74.08	87.87	98.37	80.38
858	71.74	77.27	89.66	92.83	83.01
859	65.39	86.86	69.82	94.41	77.41
860	63.09	69.58	81.49	91.43	75.07
861	69.38	76.63	83.04	94.14	79.71
862	71.91	77.60	87.95	94.25	82.45
863	66.05	72.61	81.65	93.70	76.87
864	83.23	96.00	85.61	98.60	90.50
865	59.11	70.34	63.56	98.18	66.78
866	80.09	84.56	91.56	98.95	87.92
867	4.35	47.87	49.59	8.52	48.71

868	72.87	79.41	86.35	95.03	82.73
869	68.88	78.88	79.49	93.06	79.18
870	77.60	90.14	82.73	97.24	86.28
871	79.62	95.07	81.94	99.08	88.02
872	81.53	93.28	85.23	98.84	89.07
873	86.57	92.24	92.43	98.89	92.33
874	67.62	72.08	84.16	97.67	77.65
875	49.22	49.22	50.00	98.44	49.61
876	66.94	85.13	70.26	98.92	76.98
877	50.78	54.20	52.39	97.91	53.28
878	80.49	97.95	81.72	98.65	89.10
879	51.60	54.71	54.63	97.64	54.67
880	4.89	47.68	49.45	9.51	48.55
881	72.03	87.81	77.66	94.67	82.42
882	79.52	95.33	81.93	98.31	88.12
883	0.84	0.84	50.00	1.68	1.65
884	76.73	85.52	84.57	98.84	85.04
885	73.06	98.14	74.13	98.35	84.46
886	74.44	80.47	87.43	96.38	83.81
887	72.27	96.52	74.63	96.05	84.18
888	73.13	86.58	79.41	95.65	82.84
889	89.42	96.70	91.94	98.59	94.26
890	39.39	52.82	72.45	72.79	61.10
891	85.82	97.76	87.33	98.79	92.25
892	80.88	99.31	81.41	99.14	89.47
893	82.61	98.38	83.82	98.27	90.52
894	67.38	87.62	72.15	94.14	79.13
895	4.89	49.30	49.91	9.60	49.60
896	55.60	93.23	59.54	92.36	72.67
897	54.23	64.21	77.94	81.33	70.41
898	56.04	63.18	75.86	87.80	68.94
899	58.36	72.03	63.72	94.67	67.62
900	63.81	91.22	66.71	95.62	77.06
901	59.24	71.44	63.12	98.60	67.03
902	76.95	96.13	79.03	97.63	86.75
903	93.05	97.42	95.22	99.40	96.31
904	74.00	85.13	81.07	97.38	83.05
905	1.77	1.77	49.94	3.54	3.41
906	41.96	55.64	75.48	72.66	64.06
907	2.38	44.92	47.18	4.65	46.03
908	3.91	46.82	44.61	7.55	45.69
909	69.28	75.88	83.38	95.16	79.45
910	33.82	52.27	69.81	62.97	59.78
911	1.78	45.81	49.74	3.56	47.69
912	72.83	88.79	77.16	97.65	82.56
913	1.70	51.70	50.00	3.40	50.84
914	76.47	82.13	88.49	97.78	85.19
915	94.78	97.27	97.28	99.19	97.27
916	66.02	73.75	81.58	91.04	77.47
917	83.46	91.94	88.83	97.82	90.36
918	74.65	96.74	76.01	98.69	85.13
919	82.32	94.99	85.17	98.56	89.81
920	90.56	95.02	94.71	98.63	94.87
921	4.08	47.71	48.23	7.85	47.97
922	65.85	71.30	81.26	96.30	75.96
923	92.48	97.03	94.95	99.25	95.98
924	2.84	45.15	47.71	5.56	46.39
925	85.64	96.62	87.71	99.38	91.95
926	61.30	66.73	75.79	95.61	70.98
927	90.63	97.96	92.23	98.93	95.01
928	4.76	49.67	49.82	9.12	49.75
929	76.57	88.41	82.63	96.77	85.42

930	74.65	91.06	78.93	96.16	84.56
931	76.18	95.21	79.22	95.72	86.48
932	71.53	94.91	74.79	94.73	83.66
933	68.19	97.54	70.20	96.22	81.64
934	84.55	89.88	92.09	99.04	90.97
935	81.88	96.84	83.89	97.90	89.90
936	62.66	76.55	67.84	97.17	71.93
937	76.40	88.28	82.80	95.75	85.46
938	79.04	96.35	81.83	95.71	88.50
939	82.96	97.38	84.88	97.52	90.70
940	93.18	97.95	94.90	99.23	96.40
941	2.32	43.32	49.07	4.60	46.02
942	82.63	97.49	84.22	98.42	90.37
943	71.16	80.06	81.35	95.89	80.70
944	70.16	81.30	77.50	97.88	79.36
945	79.73	83.44	92.50	99.16	87.73
946	61.09	66.54	73.70	96.85	69.94
947	64.72	66.61	92.26	97.72	77.36
948	1.39	45.41	49.84	2.77	47.52
949	62.01	65.98	84.03	94.96	73.92
950	54.68	98.48	56.21	96.96	71.57
951	74.84	82.97	84.31	97.76	83.63
952	61.36	66.33	74.69	97.31	70.26
953	76.88	87.44	83.34	98.25	85.34
954	84.15	88.66	93.19	98.02	90.87
955	80.14	89.78	86.19	97.91	87.95
956	48.56	49.09	49.46	97.13	49.27
957	90.96	98.55	92.06	99.54	95.19
958	2.32	47.52	49.31	4.57	48.40
959	76.47	82.64	88.72	95.64	85.57
960	61.61	66.82	80.68	93.50	73.10
961	1.30	1.30	50.00	2.59	2.53
962	89.74	96.57	92.44	98.29	94.46
963	76.33	89.97	81.51	96.28	85.53
964	49.25	49.25	50.00	98.50	49.62
965	86.29	95.70	89.05	99.45	92.26
966	5.83	49.48	49.59	11.03	49.54
967	79.57	87.16	88.06	96.88	87.60
968	59.11	62.92	88.27	93.40	73.47
969	91.30	96.93	93.73	99.24	95.31
970	77.70	88.08	84.16	97.80	86.08
971	78.43	83.93	90.63	95.65	87.15
972	81.75	90.38	88.13	96.88	89.24
973	1.89	38.25	45.66	3.74	41.63
974	93.96	97.18	96.43	99.45	96.81
975	59.38	66.12	79.87	89.32	72.35
976	7.82	52.65	52.63	14.51	52.64
977	5.15	50.03	50.03	9.79	50.03
978	78.26	84.79	87.89	99.00	86.31
979	40.88	51.52	64.79	77.95	57.40
980	67.47	78.76	74.79	97.51	76.72
981	51.39	57.60	52.56	98.50	54.96
982	76.73	88.73	82.11	98.54	85.29
983	3.17	46.01	46.86	6.15	46.43
984	57.59	61.42	71.72	96.63	66.17
985	54.36	59.84	58.75	97.87	59.29
986	67.56	77.33	76.47	96.85	76.90
987	85.89	91.78	92.15	97.91	91.97
988	64.00	93.69	66.34	96.25	77.68
989	73.90	83.05	82.34	98.26	82.69
990	57.94	69.23	62.03	97.83	65.43
991	65.68	71.54	82.72	93.99	76.72

992	83.81	93.24	88.18	98.20	90.64
993	54.55	57.83	76.39	94.52	65.83
994	77.45	90.32	81.99	98.78	85.95
995	61.09	66.68	72.02	98.03	69.25
996	69.39	75.08	83.04	97.98	78.86
997	60.88	67.31	78.26	91.69	72.37
998	69.13	80.38	76.74	97.11	78.52
999	50.63	56.58	89.11	88.29	69.22
1000	85.33	97.85	86.79	98.71	91.99
1001	72.13	94.13	74.25	98.09	83.02
1002	75.31	85.39	83.19	96.52	84.28
1003	72.00	78.25	85.82	95.71	81.86
1004	58.24	61.88	83.00	94.31	70.90
1005	62.03	65.75	83.88	95.54	73.72
1006	90.55	95.32	94.37	98.99	94.84
1007	86.77	94.68	90.48	98.91	92.53
1008	69.91	75.61	82.90	98.82	79.09
1009	62.52	72.51	69.84	97.19	71.15
1010	70.70	82.10	78.25	96.86	80.13
1011	61.19	64.31	87.81	95.36	74.25
1012	1.31	13.79	47.01	2.62	21.32
1013	0.62	0.62	50.00	1.25	1.23
1014	71.51	73.56	93.39	98.63	82.30
1015	71.73	75.14	91.08	97.03	82.34
1016	90.94	96.90	93.37	99.22	95.10
1017	2.18	45.72	49.48	4.33	47.53
1018	67.85	96.12	69.56	97.34	80.71
1019	65.81	74.25	76.13	96.41	75.18
1020	69.22	91.88	71.94	97.18	80.70
1021	54.80	60.41	80.47	89.69	69.01
1022	2.79	50.57	50.10	5.50	50.34
1023	89.21	98.59	90.21	99.49	94.21
1024	72.71	96.15	75.15	96.11	84.36
1025	71.30	89.90	75.03	97.02	81.79
1026	59.63	63.25	84.40	94.57	72.31
1027	80.49	99.73	80.76	99.47	89.25
1028	88.76	98.43	89.88	99.32	93.96
1029	73.21	88.75	77.73	97.49	82.87
1030	50.73	55.04	66.39	91.68	60.18
1031	73.11	94.75	75.01	98.46	83.73
1032	72.06	96.03	74.40	96.33	83.84
1033	2.39	40.04	49.34	4.76	44.20
1034	90.42	98.27	91.72	99.39	94.88
1035	2.29	51.89	50.17	4.55	51.02
1036	75.04	95.51	77.40	97.08	85.51
1037	1.99	51.81	50.17	3.95	50.98
1038	68.99	90.45	71.87	97.57	80.09
1039	2.52	43.71	48.16	4.97	45.83
1040	49.85	54.89	63.38	89.99	58.83
1041	64.79	73.20	74.70	96.68	73.94
1042	82.95	91.47	88.36	98.87	89.89
1043	81.00	86.81	90.56	97.62	88.64
1044	1.33	43.57	49.22	2.65	46.23
1045	80.09	95.46	82.69	97.66	88.61
1046	65.53	71.46	83.35	93.22	76.95
1047	84.00	97.45	85.43	99.40	91.05
1048	56.95	60.29	86.85	94.09	71.17
1049	59.06	63.68	71.31	97.22	67.28
1050	67.50	71.39	86.18	97.26	78.09
1051	49.72	53.36	68.79	92.68	60.10
1052	63.60	68.54	83.96	93.79	75.47
1053	89.39	93.42	94.90	98.67	94.16

1054	61.63	71.77	69.52	95.59	70.63
1055	1.11	1.11	49.90	2.22	2.17
1056	62.05	99.25	62.80	98.51	76.92
1057	85.32	96.65	87.73	97.60	91.97
1058	72.44	76.39	91.61	95.47	83.31
1059	91.10	96.42	94.05	98.36	95.22
1060	10.31	54.73	52.83	18.82	53.77
1061	95.09	98.65	96.33	98.89	97.47
1062	95.16	98.97	96.11	99.01	97.52
1063	85.41	88.63	95.73	96.57	92.05
1064	90.40	92.59	97.32	98.12	94.90
1065	70.41	75.43	91.69	92.22	82.77
1066	95.99	97.32	98.55	99.13	97.93
1067	94.59	98.12	96.27	98.82	97.19
1068	84.38	95.52	87.02	99.32	91.07
1069	75.86	86.01	84.31	93.99	85.15
1070	83.33	95.34	86.62	96.51	90.77
1071	81.30	97.40	83.36	96.84	89.83
1072	78.35	92.87	82.45	96.14	87.35
1073	78.41	87.77	85.90	96.21	86.82
1074	82.64	91.55	88.30	96.97	89.89
1075	81.23	92.32	86.44	95.00	89.28
1076	37.60	59.84	72.79	58.93	65.68
1077	2.54	52.09	50.11	5.06	51.08
1078	84.33	91.77	90.10	97.94	90.93
1079	56.14	90.58	57.27	98.11	70.17
1080	81.87	89.18	89.59	96.10	89.39
1081	80.73	95.27	84.16	95.43	89.37
1082	57.29	67.10	84.23	82.19	74.69
1083	70.79	79.16	83.73	91.93	81.38
1084	13.55	50.44	50.56	23.94	50.50
1085	58.89	62.55	78.22	95.70	69.52
1086	79.00	81.33	94.94	98.99	87.61
1087	83.82	87.95	93.25	99.30	90.52
1088	94.53	96.60	97.66	99.55	97.13
1089	65.34	70.30	83.35	95.24	76.27
1090	84.62	94.53	88.15	98.38	91.23
1091	48.95	49.13	49.82	97.91	49.47
1092	51.82	57.31	91.00	89.23	70.33
1093	85.72	94.06	89.77	98.68	91.86
1094	70.48	96.62	72.13	97.55	82.60
1095	1.59	1.59	50.00	3.18	3.08
1096	67.06	71.63	85.25	96.07	77.85
1097	48.86	48.87	49.99	97.72	49.42
1098	90.84	97.41	92.80	99.55	95.05
1099	7.20	50.18	50.20	13.44	50.19
1100	6.96	52.21	50.79	13.23	51.49
1101	80.66	96.12	83.00	97.63	89.08
1102	52.83	64.28	84.03	78.75	72.84
1103	90.38	98.11	91.89	98.70	94.90
1104	51.29	61.26	81.31	80.79	69.88
1105	89.05	94.72	93.34	97.42	94.03
1106	9.52	54.37	50.98	18.08	52.62
1107	92.37	98.15	94.03	98.04	96.05
1108	95.12	98.31	96.66	98.91	97.48
1109	82.93	86.63	95.02	95.54	90.63
1110	93.66	99.13	94.50	98.66	96.76
1111	6.61	50.43	50.15	12.60	50.29
1112	53.58	57.66	61.77	95.69	59.64
1113	89.66	95.26	93.51	97.79	94.38
1114	88.96	94.79	93.15	97.65	93.96
1115	77.85	84.83	88.81	94.19	86.77

1116	80.32	94.55	83.96	95.74	88.94
1117	91.70	96.09	95.02	98.49	95.55
1118	78.09	86.11	87.19	95.63	86.65
1119	89.15	93.97	94.05	98.45	94.01
1120	3.59	52.31	50.30	7.09	51.29
1121	85.75	93.78	90.34	96.88	92.03
1122	9.62	51.41	51.03	17.60	51.22
1123	56.07	78.29	57.35	98.77	66.20
1124	84.99	89.98	92.77	98.35	91.35
1125	71.17	84.02	78.09	95.77	80.95
1126	43.95	61.59	77.32	67.31	68.56
1127	69.68	84.22	77.92	90.27	80.95
1128	37.94	59.93	70.63	58.80	64.84
1129	90.00	96.45	92.83	98.18	94.61
1130	82.53	95.75	85.26	97.47	90.20
1131	56.75	60.03	81.86	94.70	69.27
1132	70.77	85.79	75.23	99.11	80.16
1133	60.47	64.92	83.93	93.31	73.21
1134	59.47	63.83	80.74	93.91	71.30
1135	79.35	97.52	81.11	97.39	88.56
1136	91.59	94.95	96.00	98.78	95.47
1137	82.11	85.79	93.73	98.22	89.58
1138	68.72	96.60	70.06	98.10	81.22
1139	76.16	92.09	79.58	98.25	85.38
1140	78.71	88.96	84.52	99.19	86.68
1141	77.47	98.39	78.73	97.86	87.47
1142	90.67	94.47	95.42	98.39	94.94
1143	92.66	96.81	95.41	98.61	96.11
1144	86.62	94.00	91.00	98.04	92.48
1145	1.33	1.33	50.00	2.65	2.58
1146	2.56	49.41	49.95	5.09	49.68
1147	84.25	90.24	91.55	97.71	90.89
1148	79.72	96.81	81.69	97.66	88.61
1149	80.23	94.57	83.05	98.52	88.43
1150	84.22	89.91	91.53	99.33	90.71
1151	67.87	71.51	88.59	96.42	79.14
1152	2.09	52.08	50.01	4.18	51.02
1153	69.81	81.16	77.01	97.84	79.03
1154	91.97	96.96	94.45	99.41	95.68
1155	48.54	48.54	50.00	97.07	49.26
1156	52.14	55.49	58.70	95.97	57.05
1157	82.30	93.51	86.22	97.98	89.72
1158	57.99	68.59	62.32	97.92	65.30
1159	48.13	53.27	70.52	89.45	60.70
1160	48.66	48.97	49.68	97.33	49.32
1161	2.31	43.60	49.75	4.60	46.47
1162	1.44	29.22	49.90	2.88	36.85
1163	91.42	95.84	94.89	98.98	95.36
1164	80.62	96.61	83.16	96.30	89.38
1165	81.40	97.20	83.47	97.07	89.82
1166	89.74	94.42	94.35	98.27	94.39
1167	85.51	95.26	88.67	98.35	91.85
1168	54.04	59.67	61.86	94.38	60.75
1169	57.25	60.89	77.92	94.84	68.36
1170	67.57	74.25	79.32	98.19	76.70
1171	65.17	75.16	72.56	98.49	73.83
1172	32.04	50.18	51.76	61.89	50.96
1173	78.95	89.00	85.31	97.38	87.12
1174	76.27	83.14	87.20	96.60	85.12
1175	67.72	73.06	81.49	98.53	77.04
1176	49.57	70.26	50.54	98.06	58.79
1177	7.02	48.51	43.87	13.54	46.08

1178	65.05	91.73	67.20	97.38	77.57
1179	64.29	90.12	66.25	98.10	76.37
1180	69.79	84.24	75.11	97.82	79.41
1181	60.18	63.49	79.29	96.90	70.52
1182	58.85	84.88	60.94	97.31	70.94
1183	70.40	75.60	85.40	97.34	80.20
1184	0.66	0.66	50.00	1.32	1.30
1185	49.16	55.49	50.19	97.88	52.71
1186	0.82	0.82	49.91	1.64	1.62
1187	62.68	67.32	76.75	98.04	71.72
1188	80.10	92.48	83.94	99.06	88.01
1189	73.87	78.02	89.01	98.76	83.15
1190	90.97	93.81	96.43	99.24	95.10
1191	51.53	56.66	53.72	97.41	55.15
1192	77.26	96.59	78.86	98.59	86.83
1193	74.13	85.47	81.10	97.11	83.23
1194	61.49	83.69	64.63	96.53	72.94
1195	73.59	89.86	77.71	97.45	83.34
1196	70.72	79.96	82.10	92.70	81.02
1197	79.99	96.56	82.52	96.33	88.99
1198	50.02	54.95	78.41	90.15	64.62
1199	65.41	68.30	93.22	95.37	78.84
1200	74.56	85.72	81.88	96.19	83.75
1201	69.43	76.69	83.52	93.53	79.96
1202	63.95	92.28	66.14	96.89	77.06
1203	86.23	88.19	96.92	99.25	92.35
1204	74.71	79.62	88.51	98.14	83.83
1205	58.26	62.18	77.54	95.01	69.01
1206	63.38	68.45	78.36	96.81	73.07
1207	61.46	66.55	75.05	96.96	70.54
1208	76.22	95.72	78.20	98.17	86.08
1209	66.94	74.77	78.51	95.97	76.60
1210	57.82	66.19	67.03	93.26	66.61
1211	88.81	92.91	94.63	99.20	93.76
1212	48.18	58.27	77.41	79.94	66.49
1213	65.48	68.30	86.76	98.07	76.43
1214	45.63	54.72	73.50	81.41	62.73
1215	60.91	74.41	66.63	95.59	70.31
1216	62.69	86.35	64.84	98.30	74.07
1217	87.49	93.94	91.87	99.62	92.89
1218	72.70	80.82	82.64	97.79	81.72
1219	1.42	48.87	49.93	2.84	49.40
1220	70.90	73.62	91.91	97.75	81.76
1221	87.16	90.69	94.97	98.97	92.78
1222	75.08	90.90	79.09	97.43	84.58
1223	92.74	98.31	94.09	99.71	96.15
1224	66.71	92.75	69.88	95.15	79.71
1225	8.24	49.58	49.74	15.30	49.66
1226	81.49	95.69	84.32	96.90	89.65
1227	1.82	44.14	49.18	3.62	46.53
1228	79.27	85.62	88.91	98.21	87.24
1229	66.48	78.76	73.76	96.01	76.18
1230	58.12	60.52	88.05	96.07	71.73
1231	79.05	94.85	81.63	98.29	87.75
1232	4.41	48.79	48.33	8.46	48.56
1233	68.05	73.38	82.58	97.73	77.71
1234	69.20	76.71	80.62	97.07	78.62
1235	57.87	66.12	63.85	97.29	64.96
1236	57.45	62.12	90.30	91.24	73.61
1237	49.03	52.35	51.24	95.29	51.79
1238	87.67	91.32	95.01	98.54	93.13
1239	70.11	92.81	72.98	96.54	81.71

1240	0.88	48.77	49.92	1.75	49.34
1241	49.06	49.06	50.00	98.12	49.53
1242	22.50	49.99	49.93	42.89	49.96
1243	65.00	68.75	82.79	97.99	75.12
1244	54.32	57.51	70.70	95.31	63.43
1245	49.70	52.34	52.61	95.71	52.48
1246	64.65	75.24	72.26	97.06	73.72
1247	77.54	91.43	81.62	98.21	86.25
1248	60.06	82.89	62.11	98.26	71.01
1249	67.50	76.47	76.82	97.52	76.64
1250	10.65	52.14	55.52	19.68	53.78
1251	74.03	79.70	88.66	94.98	83.94
1252	72.95	86.31	79.47	95.22	82.75
1253	63.98	94.62	67.68	93.18	78.91
1254	80.15	96.42	82.66	96.60	89.01
1255	69.08	75.02	85.86	93.77	80.07
1256	48.14	52.88	71.74	90.31	60.88
1257	92.67	97.33	94.91	99.12	96.10
1258	93.43	96.74	96.29	99.17	96.51
1259	75.35	83.91	84.93	95.78	84.41
1260	51.48	87.84	53.14	96.74	66.22
1261	69.23	84.08	74.65	97.11	79.09
1262	48.44	49.96	49.92	96.02	49.94
1263	59.60	75.47	62.66	98.39	68.47
1264	60.52	62.64	88.16	97.16	73.24
1265	54.67	58.02	88.35	93.64	70.05
1266	51.13	61.51	52.99	96.77	56.93
1267	47.96	50.17	50.56	94.67	50.36
1268	82.33	92.30	86.98	98.47	89.56
1269	70.06	95.47	71.62	98.25	81.84
1270	87.17	90.90	94.79	98.60	92.81
1271	62.25	69.97	73.04	95.44	71.47
1272	52.47	56.08	64.98	94.20	60.20
1273	43.61	54.68	76.39	77.68	63.74
1274	52.80	57.13	83.42	91.66	67.81
1275	69.46	77.77	79.28	98.14	78.52
1276	74.10	95.73	76.05	97.81	84.76
1277	4.46	46.39	48.04	8.58	47.20
1278	56.57	72.41	59.70	96.68	65.45
1279	0.76	0.76	50.00	1.53	1.50
1280	48.65	49.24	49.39	97.30	49.32
1281	65.37	73.57	75.03	97.64	74.29
1282	67.74	78.83	76.13	95.64	77.46
1283	7.20	51.10	51.08	13.43	51.09
1284	72.11	80.47	83.24	94.87	81.83
1285	73.68	85.51	80.61	96.48	82.99
1286	70.71	89.70	75.54	94.08	82.02
1287	9.62	50.35	50.35	17.55	50.35
1288	3.59	47.74	49.32	7.00	48.52
1289	74.29	92.65	78.01	95.80	84.70
1290	40.40	53.98	77.22	72.91	63.54
1291	79.09	86.03	88.98	95.43	87.48
1292	6.61	50.83	50.38	12.53	50.60
1293	59.68	69.93	64.55	98.68	67.13
1294	36.80	51.21	64.81	70.56	57.21
1295	66.19	98.15	66.76	99.17	79.47
1296	0.94	0.94	50.00	1.88	1.84
1297	43.56	49.07	44.28	87.11	46.56
1298	1.39	1.39	50.00	2.78	2.70
1299	61.68	64.72	93.40	94.58	76.45
1300	67.22	88.19	69.96	98.41	78.03
1301	90.26	94.21	95.12	99.00	94.67

1302	73.35	94.70	75.65	97.52	84.11
1303	62.51	68.36	77.82	94.71	72.78
1304	1.64	51.63	50.00	3.28	50.81
1305	57.62	60.81	85.22	94.71	70.98
1306	89.43	91.60	96.97	99.59	94.21
1307	89.18	95.15	92.91	99.02	94.01
1308	52.43	57.93	80.01	89.40	67.20
1309	64.26	69.52	80.80	95.50	74.74
1310	77.47	95.05	80.23	96.95	87.01
1311	72.35	83.56	79.58	97.56	81.52
1312	72.12	77.01	88.58	95.80	82.39
1313	11.38	51.15	58.37	21.81	54.52
1314	74.32	85.05	81.82	96.82	83.40
1315	60.97	68.19	80.72	88.90	73.93
1316	72.24	83.15	81.07	94.02	82.09
1317	75.32	90.42	80.12	95.91	84.96
1318	83.97	93.10	88.44	98.39	90.71
1319	71.44	89.51	76.08	95.09	82.25
1320	86.49	93.71	91.21	97.31	92.44
1321	72.98	85.90	79.22	96.78	82.43
1322	91.24	95.62	94.89	98.89	95.26
1323	78.56	95.68	80.46	99.12	87.41
1324	59.59	68.52	71.86	90.32	70.15
1325	67.84	95.02	70.23	96.22	80.76
1326	0.86	0.86	50.00	1.72	1.69
1327	85.90	88.24	96.27	99.30	92.08
1328	83.97	88.31	93.11	98.94	90.65
1329	54.31	64.23	57.18	97.40	60.50
1330	58.12	70.75	61.30	98.74	65.69
1331	64.51	74.27	71.71	98.80	72.97
1332	55.13	87.12	56.09	98.47	68.24
1333	58.73	68.72	64.55	96.74	66.57
1334	58.27	65.67	67.50	94.87	66.57
1335	48.85	98.45	50.39	96.91	66.66
1336	2.33	49.40	49.76	4.57	49.58
1337	73.62	96.25	75.44	97.68	84.59
1338	67.34	71.59	84.17	97.90	77.37
1339	94.93	97.57	97.11	99.53	97.34
1340	86.69	98.86	87.68	98.46	92.94
1341	75.41	85.97	82.64	97.21	84.27
1342	3.89	47.51	49.63	7.67	48.55
1343	65.82	75.31	77.30	92.58	76.29
1344	90.16	96.39	93.03	98.41	94.68
1345	2.60	46.30	48.23	5.09	47.24
1346	73.61	91.88	77.50	95.82	84.08
1347	75.78	88.16	81.96	96.02	84.95
1348	59.85	70.08	70.30	90.64	70.19
1349	6.14	49.31	49.79	11.80	49.55
1350	74.82	80.53	89.08	94.88	84.59
1351	44.33	57.96	74.36	72.80	65.15
1352	47.69	96.05	51.64	92.12	67.17
1353	74.39	86.14	80.95	97.26	83.47
1354	49.41	49.41	50.00	98.82	49.70
1355	87.11	91.64	93.70	99.47	92.66
1356	87.46	98.49	88.41	99.70	93.18
1357	69.40	78.88	78.35	97.46	78.61
1358	60.96	73.01	66.39	97.25	69.54
1359	72.73	81.90	81.11	98.86	81.50
1360	58.94	67.49	64.82	98.11	66.12
1361	1.46	1.46	50.00	2.92	2.84
1362	82.83	90.90	88.72	98.73	89.80
1363	57.14	62.37	69.21	94.98	65.61

1364	88.34	89.49	98.28	99.58	93.68
1365	0.73	50.73	50.00	1.46	50.36
1366	91.89	97.23	94.13	99.16	95.66
1367	53.10	79.48	55.78	95.06	65.56
1368	71.00	75.68	87.76	96.46	81.27
1369	51.04	57.21	88.98	87.87	69.65
1370	3.17	46.95	49.57	6.26	48.22
1371	91.26	94.32	96.27	98.86	95.28
1372	66.91	95.18	69.11	96.48	80.08
1373	63.32	80.72	69.07	93.67	74.44
1374	65.98	73.08	81.82	92.22	77.20
1375	75.25	98.89	76.22	98.20	86.09
1376	2.11	8.36	48.41	4.23	14.25
1377	90.22	93.43	95.95	98.79	94.67
1378	78.83	96.46	81.15	96.91	88.14
1379	75.60	86.28	82.10	98.93	84.14
1380	84.67	92.62	89.78	97.98	91.18
1381	90.33	93.75	95.72	98.93	94.72
1382	78.66	85.87	87.79	97.56	86.82
1383	90.44	95.47	94.13	98.69	94.79
1384	63.34	70.78	77.06	93.21	73.78
1385	39.10	50.58	56.89	75.99	53.55
1386	86.84	94.40	90.73	99.31	92.53
1387	89.67	96.27	92.45	99.45	94.32
1388	67.41	74.56	79.71	96.42	77.05
1389	85.33	93.12	90.03	98.88	91.55
1390	63.65	68.72	86.28	92.53	76.51
1391	49.12	49.17	49.95	98.24	49.56
1392	62.87	99.19	63.67	98.39	77.56
1393	82.80	88.63	90.91	98.95	89.76
1394	89.03	91.94	96.00	99.51	93.93
1395	49.80	99.38	50.43	98.75	66.90
1396	76.30	79.21	92.71	99.03	85.43
1397	48.15	54.27	93.88	87.89	68.78
1398	48.47	49.80	49.93	96.36	49.86
1399	78.99	95.75	81.26	97.99	87.91
1400	90.33	97.35	92.32	99.38	94.77
1401	87.72	97.45	89.40	99.51	93.25
1402	0.91	0.91	50.00	1.82	1.79
1403	83.83	95.67	86.62	97.96	90.92
1404	74.80	97.72	76.44	97.25	85.78
1405	82.04	86.91	92.03	98.07	89.40
1406	74.99	90.13	79.58	96.74	84.52
1407	65.41	91.84	67.35	97.89	77.71
1408	83.28	99.61	83.58	99.55	90.89
1409	55.18	60.51	72.20	91.63	65.84
1410	52.84	98.24	54.32	97.03	69.96
1411	80.20	97.82	81.21	99.46	88.75
1412	1.86	47.44	49.12	3.66	48.26
1413	80.47	95.15	83.15	98.03	88.74
1414	65.28	77.09	73.64	94.23	75.33
1415	89.80	93.26	95.48	99.60	94.36
1416	61.32	77.97	64.77	98.10	70.76
1417	51.30	54.05	61.86	95.24	57.69
1418	86.89	94.72	90.49	99.57	92.55
1419	80.72	85.36	91.50	99.13	88.32
1420	75.81	97.54	77.39	97.60	86.31
1421	92.06	96.32	95.18	98.91	95.74
1422	61.28	66.62	84.36	91.69	74.45
1423	56.40	59.89	72.89	95.77	65.76
1424	65.33	71.48	78.71	96.75	74.92
1425	56.45	61.48	66.67	95.82	63.97

1426	0.85	0.85	50.00	1.70	1.67
1427	48.41	54.32	87.18	88.24	66.94
1428	57.05	97.57	57.64	98.92	72.47
1429	76.11	78.68	93.81	98.51	85.58
1430	44.94	56.03	85.33	78.31	67.64
1431	66.96	70.47	87.63	96.84	78.12
1432	90.57	99.23	91.17	99.48	95.03
1433	58.35	64.12	84.08	89.91	72.76
1434	53.83	59.00	59.42	96.41	59.21
1435	70.83	92.72	73.29	97.83	81.87
1436	64.04	91.04	65.75	98.29	76.36
1437	68.49	82.10	74.08	98.24	77.88
1438	61.61	67.77	75.38	94.93	71.37
1439	1.58	1.58	50.00	3.16	3.06
1440	65.02	91.64	67.03	97.72	77.43
1441	0.84	38.42	47.18	1.68	42.35
1442	0.96	0.96	50.00	1.92	1.88
1443	70.42	77.64	82.25	96.94	79.88
1444	48.44	48.44	50.00	96.89	49.21
1445	3.10	49.12	49.70	6.06	49.41
1446	67.43	71.58	89.74	94.39	79.64
1447	93.81	95.38	98.14	99.53	96.74
1448	83.36	90.21	90.10	98.75	90.15
1449	85.57	94.15	89.40	99.28	91.71
1450	79.92	87.12	88.08	98.90	87.59
1451	71.83	95.75	73.96	96.99	83.46
1452	76.83	81.94	89.48	97.92	85.54
1453	63.23	68.21	86.28	92.56	76.19
1454	83.06	83.81	98.84	99.16	90.71
1455	54.73	59.06	94.59	91.54	72.71
1456	1.05	1.05	50.00	2.10	2.06
1457	83.57	96.66	85.61	98.56	90.80
1458	85.53	94.19	89.33	99.23	91.70
1459	59.78	65.44	84.51	90.48	73.76
1460	46.72	54.76	81.36	83.89	65.46
1461	0.89	0.89	50.00	1.78	1.75
1462	76.66	81.75	89.86	97.06	85.61
1463	89.00	93.72	94.07	98.93	93.89
1464	83.59	96.36	86.07	97.51	90.92
1465	64.36	74.67	73.75	94.28	74.21
1466	68.60	98.72	69.68	97.92	81.70
1467	69.54	73.03	89.02	97.39	80.23
1468	86.63	99.33	87.11	99.48	92.82
1469	90.99	93.86	96.48	98.49	95.15
1470	12.72	54.29	54.41	22.57	54.35
1471	90.85	97.20	93.12	98.46	95.12
1472	88.18	98.05	89.55	99.17	93.61
1473	3.24	46.44	46.49	6.28	46.46
1474	60.91	65.59	75.49	96.75	70.20
1475	26.54	51.28	66.03	50.64	57.73
1476	58.56	64.71	66.19	98.30	65.44
1477	53.22	56.86	65.28	94.71	60.78
1478	60.77	99.50	61.27	99.00	75.84
1479	57.11	62.98	75.27	91.13	68.58
1480	10.82	51.95	52.77	19.59	52.36
1481	87.26	98.39	88.57	98.30	93.22
1482	76.21	86.34	83.79	96.45	85.04
1483	4.50	46.19	48.06	8.66	47.11
1484	92.75	95.05	97.26	99.09	96.14
1485	87.45	91.57	94.51	97.94	93.02
1486	82.13	97.16	84.35	96.75	90.30
1487	0.97	0.97	50.00	1.93	1.90

1488	79.21	83.68	91.23	98.83	87.29
1489	66.36	75.46	74.59	98.84	75.02
1490	58.68	61.21	81.73	96.94	70.00
1491	1.17	40.41	47.11	2.32	43.51
1492	89.93	96.91	92.25	99.12	94.53
1493	84.50	92.39	89.86	97.43	91.11
1494	81.82	95.76	84.21	98.43	89.62
1495	74.60	81.83	85.25	97.60	83.50
1496	83.60	97.86	84.91	98.93	90.93
1497	88.01	97.28	89.98	98.67	93.48
1498	86.37	92.06	92.46	98.27	92.26
1499	2.36	44.00	46.93	4.63	45.42
1500	49.17	49.31	49.86	98.35	49.58
1501	87.46	96.11	90.20	98.73	93.06
1502	90.04	94.88	94.16	99.23	94.52
1503	54.35	76.32	55.50	98.53	64.26
1504	49.23	49.81	49.96	98.28	49.88
1505	49.41	49.41	50.00	98.83	49.70
1506	71.70	94.39	74.00	97.35	82.96
1507	74.46	82.61	85.32	94.49	83.94
1508	82.49	95.40	85.54	96.98	90.20
1509	62.86	78.88	66.66	98.39	72.26
1510	78.61	96.50	80.37	98.53	87.70
1511	2.81	48.76	49.77	5.54	49.26
1512	92.85	97.81	94.66	99.32	96.21
1513	89.26	94.73	93.38	99.06	94.05
1514	0.57	18.02	35.87	1.14	23.99
1515	53.81	86.02	54.88	98.11	67.01
1516	81.17	96.92	82.91	98.57	89.37
1517	87.27	99.51	87.61	99.71	93.18
1518	88.44	99.79	88.65	99.59	93.89
1519	55.74	74.76	57.50	98.15	65.00
1520	70.46	77.13	82.19	98.40	79.58
1521	84.02	99.34	84.55	99.19	91.35
1522	62.90	94.01	65.65	95.17	77.31
1523	91.88	96.08	95.16	99.30	95.62
1524	72.77	78.37	86.15	97.86	82.08
1525	69.35	95.87	71.41	96.78	81.85
1526	80.74	89.15	87.42	98.56	88.27
1527	58.85	62.69	81.51	94.46	70.87
1528	87.16	94.15	91.34	99.19	92.72
1529	72.53	76.45	89.82	97.31	82.60
1530	53.92	75.76	54.88	98.78	63.65
1531	66.90	70.56	84.90	98.27	77.07
1532	0.62	50.62	50.00	1.25	50.31
1533	49.06	49.28	49.76	98.11	49.52
1534	56.69	61.35	75.38	93.14	67.65
1535	62.46	71.55	71.54	95.69	71.54
1536	63.36	69.21	79.65	94.24	74.06
1537	86.25	95.61	89.08	99.39	92.23
1538	53.02	60.53	55.16	98.13	57.72
1539	68.32	71.17	92.67	96.21	80.51
1540	2.22	46.65	48.84	4.37	47.72
1541	78.17	83.55	89.57	98.38	86.45
1542	55.92	59.19	78.34	94.88	67.43
1543	57.51	61.65	70.34	96.63	65.71
1544	1.26	37.20	49.54	2.53	42.49
1545	36.50	56.21	78.95	62.53	65.66
1546	81.32	89.46	88.08	98.02	88.77
1547	64.60	68.30	89.59	94.59	77.51
1548	1.58	1.58	50.00	3.15	3.06
1549	53.70	78.81	54.62	98.63	64.53

1550	52.93	55.84	67.31	95.43	61.04
1551	5.98	50.08	50.48	11.66	50.28
1552	49.95	53.18	55.91	94.14	54.51
1553	76.04	80.82	89.41	98.29	84.90
1554	47.93	53.36	63.67	88.57	58.06
1555	52.32	56.51	79.49	91.96	66.05
1556	77.49	94.76	79.70	98.99	86.58
1557	1.09	1.09	50.00	2.17	2.13
1558	0.76	3.09	42.18	1.53	5.76
1559	59.97	72.55	64.56	97.67	68.32
1560	81.32	91.05	86.66	98.33	88.80
1561	77.50	93.09	80.73	98.14	86.47
1562	91.84	95.78	95.40	99.29	95.59
1563	1.07	10.85	44.53	2.13	17.45
1564	88.19	96.54	90.56	99.53	93.45
1565	86.57	91.73	93.03	98.43	92.38
1566	76.28	96.46	78.29	97.41	86.43
1567	78.75	89.17	84.78	97.88	86.92
1568	84.85	99.25	85.58	98.63	91.91
1569	43.06	51.16	59.83	82.84	55.16
1570	1.10	26.10	49.97	2.20	34.29
1571	1.12	1.12	50.00	2.24	2.19
1572	77.33	81.34	91.74	97.69	86.23
1573	1.04	1.04	50.00	2.08	2.03
1574	49.81	52.81	51.88	96.39	52.34
1575	61.89	66.11	79.93	96.11	72.36
1576	49.51	98.94	50.57	97.88	66.93
1577	75.47	80.74	88.15	98.53	84.28
1578	69.24	73.35	89.51	95.35	80.63
1579	6.69	51.82	51.24	12.56	51.53
1580	82.90	87.62	92.58	97.88	90.03
1581	81.10	88.85	88.40	97.84	88.62
1582	69.44	90.23	72.73	96.92	80.54
1583	74.49	88.42	79.28	98.39	83.60
1584	52.74	57.63	85.59	90.49	68.88
1585	44.67	53.35	73.74	82.26	61.91
1586	63.34	85.64	65.80	98.20	74.42
1587	50.27	54.02	59.38	93.00	56.57
1588	49.98	98.78	51.20	97.57	67.44
1589	1.21	1.21	50.00	2.43	2.37
1590	57.62	60.18	86.04	95.86	70.83
1591	58.94	63.17	87.38	92.63	73.33
1592	52.61	63.14	58.09	91.71	60.51
1593	71.46	77.47	86.81	94.43	81.88
1594	70.86	95.11	72.35	98.75	82.18
1595	6.39	49.99	49.99	12.02	49.99
1596	72.14	80.42	82.37	96.97	81.38
1597	92.17	96.83	94.82	98.96	95.81
1598	71.64	95.95	73.40	97.74	83.17
1599	56.56	93.40	57.43	98.53	71.12
1600	83.92	96.64	85.88	99.07	90.94
1601	87.66	97.82	89.26	98.62	93.35
1602	91.71	97.63	93.64	98.92	95.59
1603	4.21	49.61	49.75	8.10	49.68
1604	30.96	53.00	68.17	56.14	59.64
1605	86.67	91.20	93.80	98.16	92.48
1606	49.27	49.27	50.00	98.54	49.63
1607	84.92	89.18	93.40	99.39	91.24
1608	62.58	90.70	65.06	96.38	75.77
1609	4.76	53.55	50.34	9.38	51.90
1610	61.41	69.59	75.54	90.66	72.45
1611	3.30	49.83	49.92	6.42	49.88

1612	1.53	46.91	49.65	3.04	48.24
1613	76.25	84.51	84.80	98.72	84.66
1614	81.04	90.97	86.29	98.64	88.57
1615	89.35	96.23	92.15	99.19	94.15
1616	65.97	80.12	71.23	97.96	75.41
1617	85.13	99.07	85.71	99.57	91.90
1618	72.24	78.02	85.69	97.50	81.67
1619	60.24	68.85	66.98	97.96	67.90
1620	82.87	86.00	94.38	99.34	90.00
1621	80.26	96.77	82.19	97.95	88.89
1622	87.34	96.39	90.07	97.61	93.12
1623	70.10	96.95	73.02	94.47	83.30
1624	78.62	97.19	80.35	97.79	87.97
1625	89.70	96.34	92.48	99.02	94.37
1626	89.42	96.60	92.00	98.66	94.25
1627	91.43	98.56	92.60	99.09	95.48
1628	70.72	98.47	71.33	99.19	82.73
1629	74.92	80.76	88.83	94.93	84.60
1630	5.54	49.82	49.94	10.65	49.88
1631	92.29	97.83	94.06	99.21	95.91
1632	77.05	99.33	77.64	98.90	87.16
1633	76.92	88.54	82.74	97.76	85.54
1634	73.97	79.82	88.53	94.60	83.95
1635	71.90	85.84	78.33	95.09	81.91
1636	86.81	94.82	90.45	98.65	92.58
1637	90.80	98.09	92.38	98.50	95.15
1638	74.36	82.75	84.23	96.33	83.48
1639	61.16	70.59	67.83	97.90	69.18
1640	87.08	95.89	89.82	99.41	92.76
1641	0.61	0.61	49.87	1.21	1.20
1642	0.84	19.74	47.97	1.68	27.97
1643	67.22	74.32	79.35	96.71	76.75
1644	65.07	75.20	75.09	93.70	75.15
1645	77.03	84.08	87.15	97.28	85.59
1646	71.79	77.93	84.17	98.39	80.93
1647	49.28	49.28	50.00	98.56	49.64
1648	81.65	95.47	83.92	99.42	89.33
1649	90.56	97.57	92.39	99.33	94.91
1650	45.12	53.85	84.13	82.60	65.67
1651	77.68	97.09	79.21	98.37	87.24
1652	89.76	93.01	95.76	99.35	94.36
1653	60.74	70.37	67.43	97.37	68.87
1654	72.74	86.70	78.06	97.72	82.15
1655	88.54	95.82	91.67	98.59	93.69
1656	83.59	96.48	85.96	97.65	90.92
1657	57.27	96.83	60.35	93.94	74.35
1658	74.86	83.54	84.12	96.74	83.83
1659	71.34	95.95	73.84	95.93	83.45
1660	78.16	88.15	85.34	95.75	86.72
1661	47.55	54.39	64.13	85.52	58.86
1662	86.17	95.87	89.06	98.03	92.34
1663	77.53	98.02	79.36	96.63	87.71
1664	2.56	44.59	47.76	5.01	46.12
1665	56.56	62.31	78.38	90.29	69.43
1666	77.36	88.93	83.75	95.38	86.26
1667	91.34	96.94	93.89	98.29	95.39
1668	48.93	49.20	49.72	97.85	49.46
1669	74.89	82.10	85.01	98.71	83.53
1670	3.19	49.54	49.80	6.22	49.67
1671	87.52	94.12	91.80	99.30	92.94
1672	69.46	75.05	84.17	96.89	79.35
1673	2.91	46.79	46.32	5.65	46.55

1674	86.24	96.92	88.53	97.68	92.54
1675	94.44	97.43	96.77	98.61	97.10
1676	89.06	94.79	93.23	97.82	94.01
1677	47.83	56.89	76.70	81.81	65.32
1678	55.60	60.52	86.36	90.78	71.17
1679	40.26	52.62	80.56	75.37	63.66
1680	74.85	87.63	80.21	98.50	83.75
1681	78.51	92.54	82.04	98.76	86.97
1682	73.45	77.63	89.74	97.35	83.24
1683	41.16	51.56	73.89	78.99	60.74
1684	69.63	74.22	88.09	95.46	80.57
1685	94.64	96.81	97.55	99.59	97.18
1686	1.24	1.24	49.39	2.48	2.42
1687	74.85	90.19	78.61	99.04	84.00
1688	89.27	98.99	90.20	98.53	94.39
1689	7.71	50.84	50.62	14.35	50.73
1690	7.03	49.94	49.95	13.15	49.94
1691	5.17	49.61	49.88	9.99	49.75
1692	81.44	92.32	86.34	96.43	89.23
1693	90.43	97.99	92.07	98.48	94.93
1694	75.57	81.70	86.92	98.56	84.23
1695	5.35	48.63	48.51	10.16	48.57
1696	69.47	82.41	76.38	96.06	79.28
1697	83.94	95.88	86.73	97.44	91.08
1698	3.42	41.60	49.15	6.78	45.06
1699	48.76	49.45	49.56	97.35	49.50
1700	0.93	0.93	50.00	1.85	1.82
1701	74.59	98.18	75.71	98.26	85.49
1702	47.71	59.49	81.24	77.17	68.69
1703	55.70	79.97	56.81	98.77	66.43
1704	85.59	93.85	89.78	98.62	91.77
1705	3.81	49.11	49.56	7.37	49.33
1706	88.42	98.25	89.69	99.06	93.77
1707	73.89	98.36	74.54	99.34	84.81
1708	49.36	49.36	50.00	98.72	49.68
1709	1.94	44.92	46.32	3.80	45.61
1710	87.63	92.60	93.56	98.27	93.07
1711	6.85	52.51	50.88	13.03	51.68
1712	9.23	52.98	52.96	16.90	52.97
1713	70.06	77.91	81.58	96.04	79.70
1714	70.63	78.02	81.88	97.54	79.90
1715	69.93	72.57	91.24	98.05	80.84
1716	71.71	84.35	77.69	98.40	80.89
1717	88.27	93.41	93.38	99.47	93.39
1718	64.84	70.34	79.20	96.95	74.51
1719	92.34	98.53	93.49	99.74	95.94
1720	0.92	50.03	50.02	1.82	50.02
1721	79.09	86.62	87.74	97.27	87.18
1722	81.70	90.05	88.44	96.53	89.23
1723	84.11	96.78	86.47	97.20	91.33
1724	5.79	50.58	50.41	10.97	50.50
1725	4.10	51.44	50.19	8.08	50.81
1726	87.49	95.79	90.70	97.38	93.18
1727	72.21	80.67	83.78	93.60	82.20
1728	78.16	83.89	88.74	99.24	86.24
1729	62.97	73.94	72.21	93.02	73.06
1730	90.56	95.30	94.47	98.26	94.88
1731	8.47	51.12	50.61	15.74	50.87
1732	68.36	80.01	76.69	94.89	78.31
1733	75.69	92.04	79.00	98.35	85.03
1734	80.15	94.60	83.12	97.86	88.49
1735	74.97	94.07	77.82	97.00	85.18

1736	73.57	83.96	81.97	95.56	82.95
1737	56.27	88.22	57.38	98.30	69.54
1738	81.93	99.07	82.67	98.85	90.13
1739	0.61	0.61	45.79	1.23	1.21
1740	91.00	95.31	94.94	98.66	95.12
1741	79.06	86.72	87.67	97.04	87.19
1742	0.58	0.58	50.00	1.16	1.14
1743	1.73	37.26	22.28	3.42	27.88
1744	90.21	96.77	92.66	99.28	94.67
1745	63.82	70.63	81.35	91.37	75.61
1746	52.43	59.35	61.69	90.11	60.50
1747	0.91	48.49	48.99	1.80	48.74
1748	76.34	81.73	89.85	95.81	85.60
1749	85.54	94.99	89.09	97.41	91.95
1750	59.65	64.93	69.77	98.16	67.26
1751	89.12	97.51	90.92	99.20	94.10
1752	89.59	99.31	90.17	99.21	94.52
1753	72.75	78.02	89.57	94.06	83.40
1754	66.46	71.99	85.47	93.27	78.15
1755	86.40	94.65	90.08	98.83	92.31
1756	4.55	50.79	50.38	8.76	50.58
1757	88.55	93.27	94.02	98.38	93.64
1758	2.64	42.85	44.92	5.16	43.86
1759	54.08	59.33	75.10	90.67	66.29
1760	73.95	87.85	79.92	95.29	83.70
1761	78.35	89.12	83.82	99.41	86.39
1762	60.91	73.86	69.55	90.75	71.64
1763	5.50	49.67	49.81	10.48	49.74
1764	57.62	62.40	72.42	94.68	67.03
1765	1.74	51.25	50.36	3.44	50.80
1766	90.84	95.98	94.02	99.54	94.99
1767	85.21	91.80	91.10	98.61	91.45
1768	83.93	98.21	85.32	98.06	91.31
1769	94.89	98.89	95.87	99.34	97.36
1770	74.21	79.17	89.05	96.62	83.82
1771	53.94	71.36	55.88	97.31	62.68
1772	1.62	42.93	49.71	3.24	46.07
1773	82.66	98.92	83.27	99.55	90.42
1774	1.05	1.05	50.00	2.10	2.06
1775	62.34	66.77	80.87	95.54	73.15
1776	85.97	95.32	89.14	98.44	92.13
1777	67.69	74.85	83.52	92.00	78.95
1778	85.12	95.58	88.21	97.44	91.75
1779	92.53	95.86	96.14	99.11	96.00
1780	53.18	58.38	75.64	90.35	65.90
1781	66.26	68.44	90.26	98.23	77.85
1782	74.36	91.46	77.47	98.88	83.89
1783	69.85	73.82	90.00	95.62	81.11
1784	75.02	77.27	94.26	98.53	84.92
1785	65.78	78.91	71.48	98.06	75.01
1786	59.00	62.82	78.59	95.36	69.83
1787	87.34	91.51	94.23	99.09	92.85
1788	75.56	89.93	80.40	96.70	84.90
1789	69.25	96.28	70.84	97.70	81.62
1790	0.80	0.80	50.00	1.59	1.57
1791	30.15	52.09	67.94	56.19	58.97
1792	11.03	52.69	54.07	19.98	53.37
1793	63.12	89.65	67.59	92.58	77.08
1794	94.14	97.26	96.56	99.27	96.91
1795	83.74	94.11	87.41	98.47	90.64
1796	78.00	83.79	88.77	98.73	86.21
1797	67.93	80.63	74.43	97.32	77.41

1798	49.03	49.06	49.98	98.07	49.51
1799	83.07	97.05	84.94	98.28	90.59
1800	85.60	94.10	89.66	98.24	91.83
1801	74.80	77.78	93.25	97.44	84.81
1802	45.02	54.34	85.17	81.52	66.35
1803	49.25	49.25	50.00	98.49	49.62
1804	46.26	53.02	70.01	86.04	60.34
1805	91.42	95.69	95.02	99.04	95.36
1806	74.20	78.14	92.52	95.29	84.73
1807	61.66	65.77	85.01	94.21	74.16
1808	89.15	99.11	89.82	99.39	94.24
1809	49.35	49.35	50.00	98.69	49.67
1810	80.56	96.66	82.12	99.41	88.80
1811	73.42	82.54	82.53	96.92	82.53
1812	89.34	95.60	92.77	98.36	94.17
1813	75.42	97.53	77.82	95.52	86.56
1814	75.67	98.26	77.29	96.95	86.52
1815	0.90	0.90	50.00	1.80	1.77
1816	64.89	70.81	79.74	95.64	75.01
1817	69.75	72.77	89.23	98.47	80.16
1818	68.00	78.01	77.21	95.90	77.61
1819	82.87	93.69	86.72	98.22	90.07
1820	60.42	67.32	70.82	96.01	69.03
1821	67.30	70.04	90.59	97.17	79.00
1822	49.31	99.13	50.18	98.27	66.63
1823	56.18	59.66	86.32	93.72	70.56
1824	0.98	0.98	50.00	1.96	1.92
1825	64.68	91.41	67.30	96.38	77.52
1826	17.07	54.10	57.56	29.68	55.77
1827	70.74	81.50	79.68	94.74	80.58
1828	72.56	77.76	87.58	96.61	82.38
1829	49.09	93.26	51.07	96.04	66.00
1830	85.75	92.80	90.96	98.13	91.87
1831	10.37	51.58	50.91	18.95	51.24
1832	63.07	90.93	69.09	89.23	78.52
1833	7.71	37.75	47.49	14.97	42.06
1834	77.25	83.47	87.66	99.02	85.51
1835	58.48	67.18	64.07	97.95	65.59
1836	50.41	57.53	68.74	85.95	62.64
1837	53.32	61.63	76.97	84.25	68.45
1838	61.23	78.85	64.78	97.41	71.12
1839	78.69	92.81	82.33	97.98	87.26
1840	53.55	57.47	76.26	92.94	65.54
1841	81.60	93.62	85.11	98.78	89.16
1842	67.83	71.96	90.37	94.25	80.12
1843	73.93	76.90	93.19	97.17	84.27
1844	2.32	48.45	49.39	4.56	48.91
1845	62.76	70.30	84.91	87.55	76.92
1846	91.13	96.51	94.12	97.40	95.30
1847	88.17	95.81	91.61	96.36	93.67
1848	96.36	97.92	98.34	99.13	98.13
1849	93.39	98.28	94.93	98.48	96.57
1850	80.07	99.23	80.50	99.55	88.89
1851	86.95	94.09	91.68	95.70	92.87
1852	90.26	97.58	92.52	96.76	94.98
1853	10.20	54.31	51.16	19.18	52.68
1854	92.87	97.54	95.00	98.41	96.25
1855	6.04	55.98	50.06	12.06	52.86
1856	90.34	94.15	95.45	97.58	94.80
1857	74.55	79.94	91.28	92.79	85.23
1858	85.15	87.91	96.41	96.70	91.96
1859	88.89	91.64	96.55	97.54	94.03

1860	91.86	99.04	92.80	98.38	95.82
1861	49.13	49.31	49.82	98.27	49.56
1862	90.91	98.33	92.35	98.31	95.24
1863	79.53	84.47	92.14	95.26	88.14
1864	74.21	80.48	88.78	93.15	84.43
1865	89.55	95.87	92.85	97.92	94.33
1866	65.37	72.19	89.65	88.32	79.98
1867	77.43	85.58	87.13	94.25	86.35
1868	84.80	97.15	87.19	96.50	91.90
1869	90.68	96.04	93.97	97.92	94.99
1870	76.22	91.09	81.40	94.33	85.97
1871	66.10	74.64	83.00	87.94	78.60
1872	49.33	49.33	50.00	98.66	49.66
1873	76.05	84.29	86.87	92.53	85.56
1874	88.07	94.86	92.20	96.60	93.51
1875	68.74	93.37	74.44	89.95	82.84
1876	51.48	56.84	76.71	89.53	65.30
1877	71.59	97.91	73.26	96.96	83.81
1878	91.44	97.05	93.86	98.60	95.43
1879	91.24	97.88	92.91	99.08	95.33
1880	83.75	94.12	87.21	99.40	90.53
1881	58.00	62.67	78.65	93.08	69.76
1882	1.61	1.61	50.00	3.22	3.12
1883	51.08	54.09	62.94	94.54	58.18
1884	1.37	12.96	47.70	2.73	20.38
1885	36.53	49.43	42.85	72.42	45.91
1886	89.96	95.89	93.18	98.81	94.52
1887	93.27	97.45	95.50	98.52	96.47
1888	81.10	87.65	90.74	93.88	89.17
1889	8.77	55.60	50.97	16.85	53.19
1890	96.57	98.04	98.44	99.21	98.24
1891	94.42	98.22	96.05	98.43	97.12
1892	80.46	94.57	84.69	93.96	89.36
1893	11.07	55.22	51.59	20.61	53.34
1894	50.36	54.34	72.89	92.10	62.26
1895	93.15	98.71	94.35	98.39	96.48
1896	81.63	97.87	83.76	96.09	90.27
1897	93.68	98.69	94.89	98.45	96.75
1898	93.33	98.74	94.48	98.61	96.56
1899	96.71	98.00	98.63	99.28	98.31
1900	82.79	86.35	95.62	94.94	90.75
1901	93.83	98.98	94.79	98.65	96.84
1902	90.35	98.50	91.71	98.03	94.98
1903	87.23	91.08	94.88	97.48	92.94
1904	89.51	93.63	94.91	98.03	94.27
1905	54.97	57.78	73.30	95.99	64.62
1906	5.11	54.22	50.69	9.98	52.40
1907	97.83	98.55	99.24	99.67	98.90
1908	92.15	98.28	93.64	98.49	95.90
1909	95.03	97.80	97.02	99.13	97.41
1910	93.65	98.64	94.82	99.18	96.69
1911	7.88	51.38	50.60	14.81	50.98
1912	87.34	97.79	89.34	96.96	93.37
1913	70.29	95.98	73.90	93.38	83.50
1914	90.12	98.09	91.85	97.57	94.87
1915	90.06	97.45	92.20	97.73	94.75
1916	76.36	94.96	78.75	97.82	86.10
1917	76.03	82.80	87.19	96.60	84.94
1918	67.57	72.39	83.43	97.50	77.52
1919	80.74	94.31	83.88	98.09	88.79
1920	71.25	87.55	76.75	94.75	81.79
1921	78.80	96.68	80.99	97.03	88.14

1922	83.69	89.35	91.35	99.40	90.34
1923	57.91	67.30	62.74	97.93	64.94
1924	80.97	86.13	91.06	98.59	88.53
1925	67.34	76.88	75.34	98.86	76.10
1926	58.82	64.67	67.83	97.63	66.21
1927	62.40	68.07	74.52	97.63	71.15
1928	88.51	98.86	89.47	98.68	93.93
1929	55.33	59.13	75.01	94.05	66.13
1930	89.05	95.44	92.45	99.46	93.92
1931	67.89	79.97	74.43	98.09	77.10
1932	6.77	51.92	53.09	12.72	52.50
1933	80.27	90.40	85.93	97.72	88.11
1934	84.27	95.28	87.10	99.06	91.01
1935	53.88	99.06	54.82	98.11	70.58
1936	2.38	51.65	50.72	4.66	51.19
1937	53.40	97.23	54.22	98.37	69.62
1938	87.19	90.72	95.05	98.64	92.83
1939	52.15	58.36	83.39	87.91	68.67
1940	88.89	94.82	92.88	98.92	93.84
1941	0.62	0.62	50.00	1.25	1.23
1942	51.42	54.14	63.51	95.22	58.45
1943	66.75	88.27	69.24	98.70	77.61
1944	71.54	78.02	83.42	98.46	80.63
1945	1.79	49.03	49.96	3.57	49.49
1946	11.79	52.53	52.89	21.10	52.71
1947	86.44	92.14	92.64	97.16	92.39
1948	6.31	49.47	49.75	11.98	49.61
1949	73.45	79.10	89.81	93.16	84.11
1950	84.68	97.53	86.19	99.08	91.51
1951	73.11	76.99	89.41	98.35	82.74
1952	2.89	50.80	50.44	5.62	50.62
1953	1.82	42.31	47.73	3.60	44.86
1954	1.39	32.37	44.28	2.76	37.40
1955	53.15	56.26	62.78	96.16	59.34
1956	68.81	77.68	78.06	98.05	77.87
1957	55.35	60.84	65.16	94.48	62.93
1958	65.16	77.18	71.80	97.25	74.39
1959	65.34	98.47	66.66	97.43	79.50
1960	0.86	0.86	50.00	1.72	1.69
1961	62.46	67.16	79.16	96.05	72.67
1962	0.71	0.71	49.84	1.42	1.40
1963	60.09	87.93	63.13	95.20	73.50
1964	47.13	58.01	75.25	78.17	65.52
1965	57.59	62.51	74.37	93.60	67.93
1966	85.70	99.00	86.43	99.13	92.29
1967	76.93	81.84	89.65	98.34	85.57
1968	92.49	96.87	95.07	99.66	95.96
1969	1.07	23.98	47.71	2.14	31.92
1970	84.70	92.36	89.80	99.29	91.06
1971	75.53	78.67	92.48	98.19	85.02
1972	69.19	85.51	73.75	97.59	79.20
1973	1.15	1.15	50.00	2.30	2.24
1974	47.86	54.34	65.14	86.42	59.25
1975	92.06	95.31	96.19	98.78	95.75
1976	87.33	97.39	89.51	97.13	93.29
1977	88.29	94.29	92.72	98.30	93.50
1978	85.46	97.37	87.27	98.37	92.04
1979	80.44	90.65	85.61	99.14	88.06
1980	95.47	97.39	97.88	99.66	97.63
1981	57.38	60.87	88.49	93.70	72.13
1982	63.65	86.54	66.48	97.23	75.20
1983	49.08	49.25	49.82	98.16	49.53

1984	3.38	49.46	49.86	6.62	49.66
1985	68.18	80.52	75.53	96.00	77.94
1986	51.76	86.97	54.12	95.37	66.72
1987	64.08	77.13	69.81	97.46	73.29
1988	89.28	92.36	95.89	99.19	94.09
1989	3.57	51.80	50.13	7.08	50.95
1990	76.29	95.45	78.63	97.45	86.22
1991	62.30	66.56	78.47	97.18	72.03
1992	87.75	90.20	96.37	99.51	93.18
1993	57.22	64.65	64.41	96.06	64.53
1994	48.96	49.96	49.96	97.26	49.96
1995	2.87	46.18	35.52	5.65	40.16
1996	53.50	58.85	75.60	90.19	66.18
1997	75.96	90.18	80.63	97.18	85.14
1998	74.46	82.64	84.74	95.84	83.67
1999	11.30	52.74	52.20	20.34	52.47
2000	88.41	97.37	90.65	97.14	93.89
2001	87.50	96.06	90.40	98.06	93.14
2002	91.90	96.62	94.69	99.18	95.64
2003	84.99	96.33	87.41	98.27	91.65
2004	58.01	62.54	69.96	96.80	66.04
2005	1.10	1.10	50.00	2.19	2.14
2006	4.87	47.90	45.89	9.34	46.87
2007	83.77	97.49	85.56	97.89	91.14
2008	79.96	98.42	81.34	97.59	89.07
2009	55.26	59.00	87.95	92.97	70.62
2010	34.15	51.99	68.64	64.02	59.17
2011	61.51	87.74	64.32	96.12	74.23
2012	24.20	51.80	62.98	45.03	56.84
2013	75.29	82.21	86.34	97.05	84.22
2014	72.01	84.09	79.11	96.39	81.52
2015	74.63	97.91	76.10	97.54	85.64
2016	49.49	54.96	71.77	88.96	62.25
2017	91.23	95.37	95.13	98.82	95.25
2018	66.32	73.30	79.51	95.42	76.28
2019	51.91	54.40	62.92	96.03	58.35
2020	0.49	11.08	40.59	0.98	17.41
2021	49.56	52.65	50.62	97.77	51.61
2022	71.27	90.31	74.50	97.90	81.65
2023	77.71	90.08	83.16	96.39	86.48
2024	73.49	81.34	85.21	94.18	83.23
2025	5.32	51.81	51.15	10.14	51.48
2026	11.06	52.99	52.65	19.93	52.82
2027	48.77	48.77	50.00	97.54	49.38
2028	71.45	83.14	78.28	97.94	80.64
2029	13.43	52.56	52.54	23.68	52.55
2030	93.32	97.72	95.28	98.81	96.49
2031	83.20	91.27	89.29	96.81	90.27
2032	71.00	97.13	73.73	94.86	83.83
2033	50.56	68.84	51.35	98.48	58.82
2034	62.76	75.97	68.07	97.51	71.80
2035	78.98	90.64	83.89	98.36	87.14
2036	5.15	50.16	50.11	9.81	50.14
2037	72.34	97.08	73.97	97.51	83.96
2038	71.12	82.71	78.08	97.88	80.33
2039	70.80	78.49	80.96	98.79	79.71
2040	63.80	98.70	64.73	98.21	78.18
2041	51.83	56.79	78.02	90.38	65.73
2042	63.65	70.17	80.43	92.51	74.95
2043	68.69	87.34	73.60	94.71	79.88
2044	4.05	50.04	50.01	7.95	50.02
2045	1.45	51.03	50.10	2.89	50.56

2046	88.65	92.65	94.67	99.48	93.65
2047	67.15	69.26	91.15	98.30	78.71
2048	1.44	1.44	50.00	2.88	2.80
2049	85.18	89.05	94.06	98.98	91.49
2050	53.12	57.60	77.78	91.62	66.18
2051	2.56	49.17	49.82	5.05	49.49
2052	79.85	88.59	87.13	96.64	87.85
2053	87.63	94.01	92.24	97.81	93.12
2054	59.81	90.51	63.70	93.03	74.78
2055	77.56	95.01	80.14	97.57	86.94
2056	91.12	96.66	93.89	98.23	95.25
2057	79.14	88.57	86.66	94.60	87.60
2058	79.38	93.96	82.93	96.61	88.10
2059	77.31	87.75	84.45	95.77	86.07
2060	81.12	94.62	84.59	96.44	89.32
2061	75.96	81.00	89.60	97.06	85.08
2062	47.35	50.24	50.73	93.03	50.48
2063	78.01	92.11	81.80	98.27	86.65
2064	71.63	98.87	72.60	98.17	83.72
2065	0.59	50.59	50.01	1.19	50.30
2066	62.39	69.15	72.23	98.14	70.66
2067	77.35	85.91	85.16	98.99	85.53
2068	58.52	61.77	75.38	97.14	67.90
2069	73.02	75.09	95.00	97.82	83.88
2070	65.34	71.61	77.66	97.66	74.51
2071	1.51	51.47	50.03	3.01	50.74
2072	72.88	92.11	76.34	96.48	83.49
2073	64.34	68.65	84.73	95.20	75.85
2074	1.46	44.31	49.98	2.91	46.97
2075	0.69	0.69	49.94	1.38	1.36
2076	35.17	52.06	68.07	65.79	58.99
2077	1.39	39.84	49.92	2.77	44.32
2078	93.49	95.66	97.44	99.51	96.55
2079	93.69	95.93	97.40	99.50	96.66
2080	56.33	61.51	63.38	97.84	62.43
2081	2.25	47.00	49.68	4.48	48.30
2082	3.43	49.89	49.99	6.83	49.94
2083	51.56	56.24	75.22	90.94	64.36
2084	5.81	51.18	50.50	11.09	50.84
2085	76.84	94.12	80.54	95.14	86.80
2086	10.07	51.80	51.48	18.33	51.64
2087	89.54	94.83	93.76	97.84	94.30
2088	7.82	47.86	48.77	14.62	48.31
2089	88.87	93.28	94.42	98.45	93.85
2090	50.96	91.46	53.84	94.28	67.78
2091	6.44	53.09	51.59	12.18	52.33
2092	69.56	82.82	75.50	97.89	78.99
2093	1.48	51.46	50.02	2.97	50.73
2094	48.45	48.85	49.59	96.90	49.21
2095	48.76	49.13	49.62	97.53	49.37
2096	50.90	54.60	68.20	92.85	60.65
2097	79.13	88.74	85.33	99.07	87.00
2098	0.59	0.59	50.00	1.17	1.16
2099	66.46	75.12	76.74	96.26	75.92
2100	60.48	66.21	74.25	95.14	70.00
2101	69.11	80.60	75.92	98.50	78.19
2102	72.92	97.76	74.40	97.52	84.50
2103	3.09	51.14	50.18	6.09	50.65
2104	86.53	96.22	89.07	98.84	92.50
2105	79.48	86.66	87.75	99.25	87.21
2106	89.49	96.89	91.84	98.78	94.30
2107	73.96	96.52	76.64	95.41	85.44

2108	79.37	90.28	85.33	95.79	87.74
2109	81.64	87.43	91.30	96.00	89.32
2110	49.10	89.82	53.44	91.35	67.01
2111	89.43	93.87	94.57	97.97	94.22
2112	70.52	81.69	79.43	93.99	80.55
2113	81.37	91.77	86.47	97.17	89.04
2114	84.83	92.16	90.61	96.76	91.38
2115	48.92	49.09	49.83	97.85	49.46
2116	88.83	91.58	96.15	99.57	93.81
2117	56.67	95.38	57.54	98.43	71.78
2118	6.35	50.86	53.40	12.22	52.10
2119	1.17	1.17	50.00	2.34	2.28
2120	83.76	88.39	92.58	99.40	90.44
2121	63.73	86.89	66.33	97.65	75.23
2122	53.82	99.14	54.68	98.28	70.48
2123	49.30	49.30	50.00	98.60	49.65
2124	86.95	91.24	94.04	98.80	92.62
2125	67.66	81.80	73.55	96.86	77.46
2126	1.36	41.35	49.95	2.72	45.25
2127	66.62	74.75	78.20	95.27	76.44
2128	14.54	51.04	52.14	25.97	51.58
2129	81.82	97.67	83.62	97.33	90.10
2130	60.14	93.69	62.68	95.40	75.11
2131	75.42	92.93	79.30	95.46	85.58
2132	68.62	84.41	74.14	95.88	78.94
2133	0.87	17.53	49.14	1.74	25.84
2134	2.55	41.44	49.98	5.11	45.31
2135	8.10	52.26	53.30	15.04	52.78
2136	90.65	94.32	95.50	98.93	94.91
2137	57.93	64.40	83.85	88.48	72.85
2138	82.06	92.64	86.47	98.19	89.45
2139	49.53	51.74	50.33	98.27	51.03
2140	75.62	93.26	79.41	95.46	85.78
2141	91.94	96.40	94.89	99.70	95.64
2142	69.65	73.08	89.43	97.27	80.43
2143	54.99	60.99	62.12	95.47	61.55
2144	71.83	77.70	86.08	96.18	81.68
2145	72.59	96.69	73.71	99.03	83.65
2146	84.50	95.05	87.55	98.91	91.15
2147	86.71	90.25	94.86	98.99	92.50
2148	91.53	95.80	95.01	99.43	95.40
2149	82.04	96.55	84.12	98.18	89.91
2150	70.15	88.48	73.24	99.21	80.14
2151	64.25	67.96	89.92	94.40	77.41
2152	72.75	79.78	83.77	98.30	81.73
2153	53.32	98.66	54.19	98.27	69.96
2154	46.40	51.55	59.83	88.97	55.38
2155	1.16	1.16	50.00	2.31	2.26
2156	51.03	99.02	52.01	98.03	68.20
2157	1.17	1.17	50.00	2.34	2.28
2158	78.11	86.37	86.31	97.71	86.34
2159	67.83	73.09	83.66	96.52	78.02
2160	83.38	96.11	85.71	98.58	90.61
2161	80.70	90.41	86.27	98.64	88.29
2162	93.43	98.85	94.43	99.11	96.59
2163	2.88	45.35	49.12	5.67	47.16
2164	77.10	84.05	87.45	96.91	85.72
2165	66.41	79.63	73.17	95.92	76.26
2166	42.51	53.62	71.22	77.19	61.18
2167	66.23	71.12	87.67	93.34	78.53
2168	87.75	98.06	89.21	98.60	93.42
2169	80.38	87.40	88.58	98.67	87.98

2170	52.02	61.59	54.82	95.78	58.01
2171	0.90	0.90	50.00	1.80	1.77
2172	6.29	51.69	51.12	11.87	51.40
2173	66.77	71.96	82.00	97.18	76.65
2174	35.78	51.09	72.26	69.27	59.86
2175	64.39	98.75	65.40	98.05	78.69
2176	0.88	24.66	48.59	1.75	32.72
2177	80.28	88.52	87.33	98.53	87.92
2178	3.22	45.32	49.40	6.38	47.27
2179	63.04	97.58	65.46	95.26	78.35
2180	92.55	98.75	93.59	99.18	96.10
2181	89.87	96.02	92.93	99.15	94.45
2182	82.82	86.93	93.23	98.39	89.97
2183	90.66	98.00	92.31	98.39	95.07
2184	0.99	50.89	50.10	1.97	50.49
2185	49.19	50.65	50.54	97.02	50.60
2186	66.20	68.95	88.35	97.81	77.45
2187	92.85	94.48	97.99	99.58	96.21
2188	90.21	94.21	95.03	99.30	94.61
2189	0.71	50.71	50.00	1.42	50.35
2190	80.69	94.47	83.54	98.84	88.67
2191	75.88	95.73	78.16	97.25	86.06
2192	2.07	43.01	49.55	4.12	46.05
2193	1.13	32.35	49.31	2.26	39.07
2194	78.93	85.11	88.71	98.91	86.87
2195	77.92	92.35	81.69	97.87	86.69
2196	83.42	89.82	90.88	97.37	90.35
2197	93.93	98.29	95.39	99.25	96.82
2198	2.98	48.24	49.77	5.89	48.99
2199	80.65	83.94	93.95	98.26	88.66
2200	54.11	59.40	59.74	96.57	59.57
2201	66.14	68.33	91.46	97.65	78.22
2202	53.67	56.98	75.42	94.20	64.92
2203	58.89	97.16	59.51	98.91	73.81
2204	75.35	96.97	77.06	97.65	85.88
2205	66.26	69.79	91.13	94.84	79.04
2206	89.71	97.75	91.52	98.28	94.53
2207	4.80	51.60	50.07	9.54	50.82
2208	89.59	96.68	92.10	98.90	94.34
2209	74.92	92.70	78.20	97.25	84.83
2210	59.89	68.00	71.14	92.91	69.54
2211	91.97	98.22	93.38	99.41	95.74
2212	71.73	95.83	74.35	95.75	83.73
2213	86.50	97.57	88.47	97.40	92.80
2214	7.07	52.02	50.94	13.35	51.48
2215	55.63	60.05	67.73	95.16	63.66
2216	77.06	82.38	89.23	98.00	85.67
2217	76.78	90.67	81.46	96.99	85.82
2218	66.74	71.79	90.18	92.01	79.94
2219	64.71	70.57	83.47	92.71	76.48
2220	54.29	86.95	57.28	94.36	69.06
2221	85.56	95.87	88.55	97.25	92.06
2222	84.10	91.85	90.02	96.36	90.93
2223	72.83	97.21	75.17	95.77	84.78
2224	9.16	48.91	49.13	16.81	49.02
2225	81.16	92.91	85.73	96.01	89.17
2226	77.22	96.03	80.14	95.44	87.37
2227	48.27	48.81	49.43	96.53	49.12
Average	61.04	74.75	75.63	82.04	73.87
Standard c	28.29	22.93	16.36	33.16	20.72
Confidence	1.17	0.95	0.68	1.38	0.86
