# Supplementary material for: In Situ Root Dataset Expansion Strategy Based on an Improved CycleGAN Generator
Source: Plant Phenomics. 2024 Feb 12;6:0148. doi: 10.34133/plantphenomics.0148 (PMC11020132; doi:10.34133/plantphenomics.0148)
Supplement: Supplementary 1 — The network and corresponding weights can be viewed on GitHub (https://github.com/jiwd123/improved_cyclegan) and Zenodo (https://doi.org/10.5281/zenodo.10460303). [file plantphenomics.0148.f1.zip › Performance evaluation_Wn+Wgn.pdf]

No.	IOU	Recall	Precision	Accuracy	F1
1	88.86	93.30	94.25	99.43	93.77
2	88.29	92.87	94.00	99.13	93.43
3	86.25	92.26	91.98	98.95	92.12
4	85.68	95.95	88.12	99.73	91.87
5	89.12	91.54	96.63	99.42	94.02
6	90.09	95.38	93.72	99.46	94.54
7	88.09	89.43	98.02	99.40	93.53
8	89.02	92.17	95.68	99.66	93.89
9	88.74	89.73	98.56	99.54	93.94
10	87.68	92.09	94.07	98.92	93.07
11	84.68	91.20	90.98	98.61	91.09
12	85.43	91.68	91.50	98.72	91.59
13	82.24	88.37	90.47	98.42	89.41
14	88.43	91.26	95.94	99.77	93.54
15	84.01	87.33	94.56	98.87	90.80
16	85.15	89.16	93.88	98.98	91.46
17	84.30	86.80	95.84	98.97	91.10
18	86.99	93.44	91.71	99.60	92.57
19	85.97	92.66	91.28	98.69	91.96
20	89.27	92.85	95.31	99.16	94.07
21	85.67	95.64	88.45	99.12	91.90
22	86.47	88.82	96.48	98.84	92.49
23	84.94	89.51	93.14	98.90	91.29
24	81.72	86.55	91.88	98.35	89.14
25	90.75	95.63	94.27	99.30	94.94
Average	86.71	91.42	93.63	99.12	92.46
Standard c	2.37	2.74	2.69	0.40	1.53
Confidence	0.93	1.07	1.06	0.16	0.60
