# Supplementary material for: In Situ Root Dataset Expansion Strategy Based on an Improved CycleGAN Generator
Source: Plant Phenomics. 2024 Feb 12;6:0148. doi: 10.34133/plantphenomics.0148 (PMC11020132; doi:10.34133/plantphenomics.0148)
Supplement: Supplementary 1 — The network and corresponding weights can be viewed on GitHub (https://github.com/jiwd123/improved_cyclegan) and Zenodo (https://doi.org/10.5281/zenodo.10460303). [file plantphenomics.0148.f1.zip › Performance evaluation_Wn.pdf]

No.	IOU	Recall	Precision	Accuracy	F1
1	88.16	93.04	93.63	99.39	93.33
2	87.57	93.38	92.58	99.09	92.98
3	85.72	93.08	90.54	98.94	91.79
4	85.05	95.46	87.75	99.72	91.44
5	88.59	91.34	96.14	99.39	93.68
6	89.31	95.07	93.08	99.41	94.06
7	87.66	89.10	97.84	99.37	93.27
8	88.00	91.50	95.08	99.63	93.25
9	88.27	89.51	98.16	99.52	93.64
10	87.19	92.18	93.33	98.89	92.75
11	84.32	91.09	90.61	98.57	90.85
12	85.19	91.40	91.46	98.69	91.43
13	81.96	88.18	90.26	98.39	89.21
14	88.32	91.89	95.03	99.77	93.44
15	83.38	86.93	94.06	98.82	90.36
16	84.19	88.43	93.33	98.90	90.82
17	83.47	86.23	95.29	98.90	90.53
18	83.04	93.94	86.46	99.49	90.05
19	85.74	91.45	92.16	98.63	91.81
20	88.90	92.90	94.77	99.14	93.83
21	85.06	95.47	87.88	99.08	91.52
22	86.53	88.91	96.44	98.85	92.52
23	85.19	90.17	92.72	98.94	91.43
24	81.01	85.27	92.48	98.22	88.73
25	90.19	95.99	93.30	99.26	94.63
Average	86.08	91.28	92.98	99.08	92.05
Standard c	2.43	2.93	2.96	0.41	1.58
Confidence	0.95	1.15	1.16	0.16	0.62
