# Supplementary material for: In Situ Root Dataset Expansion Strategy Based on an Improved CycleGAN Generator
Source: Plant Phenomics. 2024 Feb 12;6:0148. doi: 10.34133/plantphenomics.0148 (PMC11020132; doi:10.34133/plantphenomics.0148)
Supplement: Supplementary 1 — The network and corresponding weights can be viewed on GitHub (https://github.com/jiwd123/improved_cyclegan) and Zenodo (https://doi.org/10.5281/zenodo.10460303). [file plantphenomics.0148.f1.zip › Performance evaluation_Ws+Wgs.pdf]

No.	IOU	Recall	Precision	Accuracy	F1
1	87.30	91.61	94.01	99.33	92.80
2	86.75	91.37	93.58	98.99	92.46
3	86.27	91.63	92.65	98.94	92.14
4	62.72	94.23	63.48	99.33	75.85
5	85.29	91.53	91.36	99.24	91.44
6	86.27	92.82	91.42	99.22	92.12
7	86.20	88.57	96.28	99.30	92.26
8	82.97	96.09	85.04	99.53	90.23
9	85.72	91.66	91.78	99.45	91.72
10	86.66	91.54	93.30	98.82	92.41
11	82.62	89.93	89.41	98.39	89.67
12	84.38	91.60	90.18	98.63	90.89
13	83.19	89.61	90.51	98.54	90.06
14	84.98	90.76	91.64	99.70	91.20
15	83.61	87.39	93.79	98.85	90.48
16	83.99	88.73	92.65	98.89	90.65
17	83.31	86.49	94.59	98.90	90.36
18	70.86	94.10	72.43	99.15	81.86
19	85.55	91.88	91.47	98.62	91.68
20	88.05	91.78	94.90	99.05	93.32
21	78.38	96.38	80.11	98.70	87.49
22	82.92	85.35	95.92	98.41	90.33
23	82.54	88.37	90.87	98.71	89.60
24	81.19	85.82	92.00	98.27	88.80
25	88.36	94.93	92.12	99.11	93.50
Average	83.20	90.97	89.82	98.96	90.13
Standard c	5.56	2.96	7.48	0.37	3.78
Confidence	2.18	1.16	2.93	0.15	1.48
