# Supplementary material for: In Situ Root Dataset Expansion Strategy Based on an Improved CycleGAN Generator
Source: Plant Phenomics. 2024 Feb 12;6:0148. doi: 10.34133/plantphenomics.0148 (PMC11020132; doi:10.34133/plantphenomics.0148)
Supplement: Supplementary 1 — The network and corresponding weights can be viewed on GitHub (https://github.com/jiwd123/improved_cyclegan) and Zenodo (https://doi.org/10.5281/zenodo.10460303). [file plantphenomics.0148.f1.zip › Performance evaluation_Ws.pdf]

No.	IOU	Recall	Precision	Accuracy	F1
1	82.79	85.82	94.65	98.96	90.01957
2	83.38	87.94	92.74	98.64	90.27329
3	83.67	88.49	92.49	98.65	90.44441
4	81.19	92.54	85.11	99.63	88.66729
5	80.21	82.68	95.11	98.70	88.46263
6	83.43	87.29	93.62	98.93	90.34167
7	82.70	84.31	97.13	99.01	90.2679
8	85.16	89.79	93.01	99.53	91.36764
9	85.26	87.19	96.78	99.36	91.73402
10	85.07	89.80	93.05	98.64	91.39469
11	81.31	88.01	89.49	98.20	88.74232
12	83.16	89.23	90.89	98.45	90.0527
13	82.00	87.33	91.33	98.35	89.28675
14	81.71	83.95	95.50	99.56	89.35178
15	81.55	84.88	93.96	98.62	89.19059
16	81.53	85.46	93.02	98.62	89.08033
17	80.21	82.93	94.70	98.57	88.42517
18	84.88	90.87	91.42	99.51	91.14438
19	83.79	88.27	93.04	98.32	90.59269
20	85.85	88.74	95.60	98.79	92.04305
21	85.18	92.92	89.97	99.04	91.42313
22	81.34	83.75	95.76	98.19	89.35481
23	82.47	86.23	93.56	98.62	89.7482
24	79.74	82.97	93.91	97.97	88.10415
25	88.31	92.10	94.90	99.05	93.47635
Average	83.03	87.34	93.23	98.80	90.12
Standard c	2.07	3.03	2.59	0.46	1.32
Confidence	0.81	1.19	1.01	0.18	0.52
