# Supplementary material for: In Situ Root Dataset Expansion Strategy Based on an Improved CycleGAN Generator
Source: Plant Phenomics. 2024 Feb 12;6:0148. doi: 10.34133/plantphenomics.0148 (PMC11020132; doi:10.34133/plantphenomics.0148)
Supplement: Supplementary 1 — The network and corresponding weights can be viewed on GitHub (https://github.com/jiwd123/improved_cyclegan) and Zenodo (https://doi.org/10.5281/zenodo.10460303). [file plantphenomics.0148.f1.zip › Subjective evaluation of various generators.pdf]

		model0		
Serial Number	1	2	3	4
1	1	2	2	2
2	1	2	2	2
3	1	2	3	2
4	1	2	2	2
5	1	2	2	2
6	1	2	3	2
7	1	2	2	2
8	1	3	3	2
9	1	3	2	2
10	1	2	2	2
11	1	2	2	2
12	1	2	3	2
13	1	2	2	2
14	1	2	2	2
15	1	2	2	2
16	1	3	3	2
17	1	2	2	2
18	1	2	2	2
19	1	2	3	2
20	1	3	2	2
Average score	1	2.2	2.3	2

		model1		
Serial Number	1	2	3	4
1	1	3	2	2
2	1	3	2	2
3	1	2	3	2
4	1	3	2	2
5	1	2	2	2
6	1	2	3	2
7	1	2	3	2
8	1	3	3	2
9	1	3	2	2
10	1	3	2	2
11	1	3	2	2
12	1	2	3	2
13	1	2	2	2
14	1	2	2	2
15	1	2	2	2
16	1	3	3	2
17	1	3	2	2
18	1	3	2	2
19	1	3	3	2
20	1	3	2	2
Average score	1	2.6	2.35	2

	model2			
Serial Number	1	2	3	4
1	1	2	3	2
2	1	3	3	2
3	1	2	2	2
4	1	3	2	2
5	1	2	3	2
6	1	2	2	2
7	1	2	3	2
8	1	2	3	2
9	1	3	2	2
10	1	2	2	2
11	1	3	2	2
12	1	3	3	2
13	1	3	2	2
14	1	2	2	2
15	1	2	2	2
16	1	2	3	2
17	1	2	2	2
18	1	2	3	2
19	1	3	2	2
20	1	3	2	2
Average score	1	2.4	2.4	2

	model3			
Serial Number	1	2	3	4
1	1	2	3	2
2	1	3	3	2
3	1	2	2	2
4	1	3	2	2
5	1	2	3	2
6	1	3	2	2
7	1	2	3	2
8	1	2	3	2
9	1	3	2	2
10	1	2	2	2
11	1	3	2	2
12	1	3	3	2
13	1	3	2	2
14	1	2	2	2
15	1	2	2	2
16	1	2	3	2
17	1	2	2	2
18	1	2	3	2
19	1	3	2	2
20	1	3	2	2
Average score	1	2.45	2.4	2

	model4			
Serial Number	1	2	3	4
1	1	3	3	2
2	1	3	3	2
3	1	2	2	2
4	1	3	2	2
5	1	2	2	2
6	1	2	2	2
7	1	2	3	2
8	1	3	3	2
9	1	3	2	2
10	1	2	3	2
11	1	2	2	2
12	1	3	2	2
13	1	3	2	2
14	1	2	2	2
15	1	2	3	2
16	1	2	2	2
17	1	3	2	2
18	1	2	3	2
19	1	3	2	2
20	1	3	2	2
Average score	1	2.5	2.35	2

	model5			
Serial Number	1	2	3	4
1	1	3	3	2
2	1	3	3	2
3	1	2	2	2
4	1	3	2	2
5	1	3	2	2
6	1	3	2	2
7	1	2	3	2
8	1	3	2	2
9	1	3	2	2
10	1	2	2	2
11	1	2	2	2
12	1	2	2	2
13	1	3	2	2
14	1	2	2	2
15	1	3	3	2
16	1	2	2	2
17	1	3	2	2
18	1	3	2	2
19	1	2	2	2
20	1	3	2	2
Average score	1	2.6	2.2	2

	model6			
Serial Number	1	2	3	4
1	1	2	2	2
2	1	2	2	1
3	1	2	2	1
4	1	3	2	1
5	1	2	2	2
6	1	3	2	2
7	1	2	2	2
8	1	3	2	2
9	1	3	2	1
10	1	2	2	2
11	1	3	3	1
12	1	2	3	2
13	1	3	2	2
14	1	2	2	2
15	1	3	3	2
16	1	2	2	1
17	1	3	2	2
18	1	2	2	2
19	1	2	2	2
20	1	3	2	2
Average score	1	2.45	2.15	1.7

		model7		
Serial Number	1	2	3	4
1	1	1	2	2
2	1	2	2	2
3	1	2	2	2
4	1	2	2	2
5	1	2	2	2
6	1	3	2	2
7	1	2	2	2
8	1	2	2	2
9	1	2	2	2
10	1	2	2	2
11	1	3	3	2
12	1	2	3	2
13	1	3	2	2
14	1	2	2	2
15	1	3	3	2
16	1	2	2	2
17	1	3	2	2
18	1	1	2	2
19	1	1	2	2
20	1	3	2	2
Average score	1	2.15	2.15	2

	AT_model1			
Serial Number	1	2	3	4
1	1	3	2	2
2	1	2	3	2
3	1	3	2	2
4	1	3	1	2
5	1	2	2	2
6	1	3	2	2
7	1	2	2	2
8	1	3	3	2
9	1	2	2	2
10	1	2	2	2
11	1	3	3	2
12	1	2	3	2
13	1	3	2	2
14	1	2	2	2
15	1	3	3	2
16	1	2	2	2
17	1	3	2	2
18	1	2	3	2
19	1	2	2	2
20	1	3	2	2
Average score	1	2.5	2.25	2

	AT_model3			
Serial Number	1	2	3	4
1	0	3	2	0
2	0	2	3	0
3	0	3	2	0
4	0	3	1	0
5	0	2	2	0
6	0	3	2	0
7	0	3	2	0
8	0	3	3	0
9	0	2	2	0
10	0	2	2	0
11	0	3	3	0
12	0	2	3	0
13	0	3	2	0
14	0	2	2	0
15	0	2	3	0
16	0	3	2	0
17	0	3	2	0
18	0	3	3	0
19	0	3	2	0
20	0	3	2	0
Average score	0	2.65	2.25	0

	AT_model4			
Serial Number	1	2	3	4
1	1	3	1	2
2	1	3	3	2
3	1	3	2	2
4	1	3	1	2
5	1	2	2	2
6	1	3	2	2
7	1	3	2	2
8	1	2	3	2
9	1	2	2	2
10	1	2	2	2
11	1	3	3	2
12	1	2	3	2
13	1	2	2	2
14	1	2	2	2
15	1	2	2	2
16	1	3	2	2
17	1	3	2	2
18	1	3	3	2
19	1	3	2	2
20	1	3	2	2
Average score	1	2.6	2.15	2

Serial Number	5
1	1
2	0
3	1
4	1
5	0
6	1
7	0
8	0
9	1
10	1
11	1
12	1
13	1
14	0
15	1
16	1
17	1
18	0
19	1
20	1
Average score	0.7

Serial Number	model	Score
1	model0	8.2
2	model1	8.65
3	model2	8.5
4	model3	8.55
5	model4	8.55
6	model5	8.5
7	model6	8
8	model7	8
9	AT_model1	8.45
10	AT_model3	5.6
11	AT_model4	8.45
